# Palladium-catalysed synthesis of triaryl(heteroaryl)methanes

**DOI:** 10.1038/ncomms14641

**Published:** 2017-03-14

**Authors:** Shuguang Zhang, Byeong-Seon Kim, Chen Wu, Jianyou Mao, Patrick J. Walsh

**Affiliations:** 1 Roy and Diana Vagelos Laboratories, Department of Chemistry, University of Pennsylvania, 231 South 34th Street, Philadelphia, Pennsylvania 19104-6323, USA; 2 Institute of Advanced Synthesis, School of Chemistry and Molecular Engineering, Jiangsu National Synergetic Innovation Center for Advanced Materials, Nanjing Tech University, 30 South Puzhu Road, Nanjing 211816, China

## Abstract

Tetraarylmethane derivatives are desirable for a variety of applications, but difficult to access with modern C–C bond-forming reactions. Here we report a straightforward method for palladium-catalysed arylation of aryl(heteroaryl)methanes and diaryl(heteroaryl)methanes with aryl chlorides. This reaction enables introduction of various aryl groups to construct triaryl(heteroaryl)methanes via a C–H functionalization in good to excellent yield, and represents the first step towards a general transition metal catalysed synthesis of tetraarylmethanes.

## Abstract (web summary)

Molecules with 4 aryl groups attached to a central carbon atom have proven challenging to synthesize despite their attractiveness for a variety of applications. Here the authors report a Pd-catalysed arylation of di- and triarylmethanes, giving a high-yielding route to an array of tetraarylmethanes.

Tetraarylmethanes and related derivatives are important building blocks, with uses ranging from molecular devices^1^,^2^,^3^,^4^ to porous organic frameworks^5^,^6^,^7^ and applications from protein translocation detection^8^ to drug delivery^9^. Furthermore, a recent study of 9,000 bioactive compounds by AstraZeneca to evaluate molecular space concluded that most biologically active compounds are linear or disk shaped, and very few are sphere-like molecules. They concluded that chemists should ‘… expand the current arsenal of tools to access less populated space …' and ‘… this may prove advantageous as the pharmaceutical industry ventures into new disease areas and new target classes which require different molecular shapes to bind and achieve the desired effect'^10^. Tetraarylmethanes are members of sphere-like molecules that have not been widely explored due to synthetic difficulties.

The classic methods to synthesize tetraarylmethanes are based on Friedel-Crafts arylations (Fig. 1a)^11^,^12^,^13^,^14^,^15^,^16^,^17^,^18^ and nucleophilic addition of alkyllithium or Grignard reagents to benzophenone derivatives (Fig. 1b)^19^,^20^,^21^. Each of these approaches has well-known limitations. Friedel-Crafts reactions require arenes with electron-donating groups, often afford mixtures of regioisomers, and are not suitable when *meta*-substituted products are required. Aryl organometallic reagents are typically highly reactive, sensitive to traces of air and moisture, and exhibit limited functional group tolerance.

Transition metal-catalysed cross-coupling reactions have emerged as an excellent method to construct C–C bonds. For example, they have been used with great success in the coupling of aryl halides to diarylmethanes to afford triarylmethanes^22^,^23^,^24^,^25^ (Fig. 1c)^26^. The application of transition metal catalysts to the synthesis of tetraarylmethanes, however, has proven quite challenging. This may be due to difficulties associated with the transmetallation of the bulky triarylmethyl organometallic species. Thus, there is tremendous unrealized potential in the transition metal catalysed construction of tetraarylmethane derivatives.

We are only aware of a handful of metal catalysed syntheses of tetraarylmethane derivatives. In their synthesis of triarylmethanes, Yorimitsu, Oshima and co-workers noted formation of 8% yield of a tetraarylmethane (Fig. 1c). Despite the intriguing nature of this byproduct, subsequent reports have not been forthcoming. Ghosh and co-workers reported the nickel-catalysed reaction of carbon tetrachloride and PhMgCl to form a 3:2 ratio of triphenylmethyl chloride to tetraphenylmethane (Fig. 1d)^27^. These products were not isolated. Recently, the palladium-catalysed arylation of fluorenes by the teams of Wu and Song, Xie, and Huang was reported to give diarylfluorenes (Fig. 1e)^28^,^29^. Fluorene is more acidic and less sterically demanding than other diarylmethane derivatives. In significant work, the team of Nambo and Crudden outlined the generation of triarylacetonitriles, Ar_3_C–CN, then assemble a 4th heteroaryl group by building on the nitrile (Fig. 1f)^30^.

Herein, we present a novel palladium-catalysed method for the synthesis of heteroaryl-substituted tetraarylmethane derivatives (Fig. 1g). This effort represents the first high-yielding transition metal catalysed preparation of tetraarylmethane derivatives. It enables the synthesis of a broad range of triaryl(heteroaryl)methanes, including those with four different aryl groups attached to the central carbon atom.

## Results

### Preliminary catalyst screening

**Figure 1:**
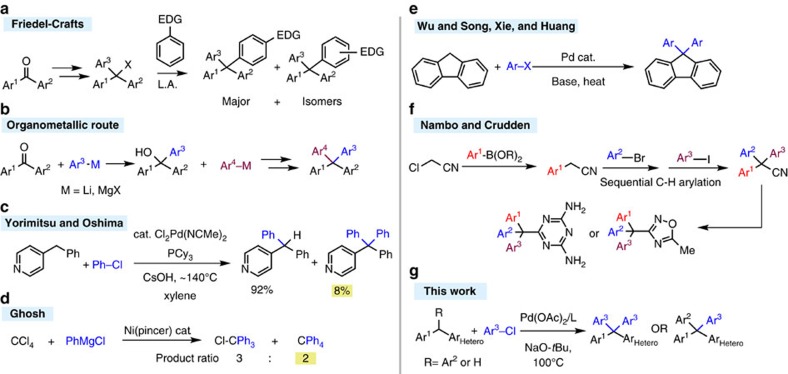
Synthesis of tetraarylmethanes. (**a**,**b**) Classic approaches to tetraarylmethanes include the Friedel-Crafts electrophilic aromatic substitution and organometallic additions to triarylmethyl cation precursors. (**c**) Formation of 8% tetraarylmethane byproduct in the synthesis of triarylmethanes. (**d**) Arylation of carbon tetrachloride resulted in up to 39% conversion to tetraphenylmethane. (**e**) Arylation of fluorene. (**f**) Sequential arylation/cycloaddition. (**g**) This work: arylation of 4-benzyl pyridine and (heteroaryl)diphenylmethanes to yield tetraarylmethane derivatives.

**Figure 2:**
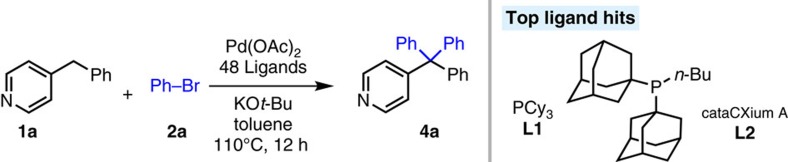
Ligands screening. Preliminary reaction screen of 48 ligands.

Given that the only example of arylation of a triarylmethane derivative we are aware of is Yorimitsu, Oshima and co-workers' 8% yield of triphenyl(4-pyridyl)methane (Fig. 1c), we chose this starting point for our reaction design. Instead of CsOH, we employed KO*t-*Bu, because of the greater solubility of this base. We reasoned that this would also allow us to lower the reaction temperature, and thus use toluene as the solvent. We initiated a search for a suitable catalyst for bis-arylation of 4-benzylpyridine (**1a**) with bromobenzene (**2a**) by examining phosphine ligands on microscale using 10 μmol of **1a** (see Supplementary Table 1). Thus, 48 electronically diverse mono- (20 mol%) and bidentate phosphine ligands (10 mol%) were tested using Pd(OAc)_2_ (10 mol%) and 4 equiv KO*t-*Bu. Reactions were conducted at 110 °C for 12 h, cooled, and quenched with water. The most promising microscale results, based on product to internal standard (4,4′-di-*tert*-butylbiphenyl) ratios, were with PCy_3_ (**L1**) and cataCXium A (**L2**, Fig. 2).

Repeating the bis-arylation reaction in Fig. 2 using 4-benzylpyridine **1a** and 4-bromotoluene **2b** with PCy_3_ (**L1**) and cataCXium A (**L2**) on laboratory scale (0.25 mmol) led to the desired product in 68 (PCy_3_) and 77% AY (cataCXium A) after 12 h (AY=assay yield, determined by ^1^HNMR analysis, see the Methods section for details) (Table 1, entries 1–2). With cataCXium A as the ligand, 4-iodotoluene **2c** reacted with 4-benzylpyridine **1a** to afford tetraarylmethane product in 76% isolated yield (entry 3). Considering that aryl chlorides are more abundant and less expensive than aryl bromides, we examined aryl chlorides. In the event, the reaction of 4-benzylpyridine **1a** with 4-chlorotoluene **2d** using the same conditions as above was conducted. Interestingly, the diarylated products were obtained in nearly identical assay yields with the aryl chlorides (66% PCy_3_ and 74% cataCXium A, entries 4–5) and aryl bromides. Although cataCXium A exhibited better AY than PCy_3,_ we chose to first optimize the reaction with the more economical and readily available PCy_3_. As outlined below, more challenging substrates gave better results with cataCXium A. To begin with, the catalyst loading was examined. Changing the loading from 10 to 5 mol% resulted in no change in AY (entry 6). Further reducing the amount of catalyst to 2.5 mol%, however, resulted in a drop to 35% (entry 7).

We next focused on the bases (LiO*t-*Bu, NaO*t-*Bu, LiN(SiMe_3_)_2_, NaN(SiMe_3_)_2_ and KN(SiMe_3_)_2_) and reaction concentration (Table 2, entries 1–8). LiO*t*-Bu and LiN(SiMe_3_)_2_ gave low yields (10–17%), probably because their stable aggregates result in less reactive bases (entries 1 and 3)^31^. NaN(SiMe_3_)_2_, and KN(SiMe_3_)_2_, are stronger bases, but resulted in some decomposition and lower yields (entries 4 and 5). The best combination from this screen was 5 mol% Pd(OAc)_2_, 10 mol% PCy_3_ and NaO*t-*Bu in toluene (0.2 M) for 12 h at 100 °C (entry 2). Solvents play an important role in deprotonative cross-coupling reactions. Therefore, four additional solvents (THF, DME, CPME and 1,4-dioxane) were examined at 100 °C. Among these, THF led to 90% AY of the diarylation products (entries 9–12). Reducing the reaction temperature to 80 °C resulted in a decrease in the AY to 37% (entry 13). The 4-benzylpyridine: 4-chlorotoluene: base ratio was next explored (entries 14–18). The use of a 1:3:3 ratio rendered 96% AY and 92% isolated yield (entry 15).

### Diarylation of aryl(4-pyridyl)methanes

Under the optimized reaction conditions (Table 2, entry 15), the deprotonative cross-coupling process (DCCP) with various aryl(4-pyridyl)methanes and aryl chlorides generally gave products in good to excellent yields (Table 3). Coupling of 4-benzylpyridine and alkyl substituted derivatives with chlorobenzene or analogues possessing alkyl substituents in the meta or para positions resulted in products **4a–4e** in 74–99% yield. Coupling with sensitive substrates, such as 4-chlorobenzonitrile and 4-chlorobenzophenone, required cataCXium A, furnishing 93 and 84% yield of **4f** and **4g**, respectively. In the absence of the palladium catalyst, 4-chlorobenzophenone gave 33% AY of the triarylmethane and *no tetraarylmethane product*. Using cataCXium A, reaction with 3-chloroanisole and 1-chloro-3,5-dimethoxybenzene provided the bis-arylated products **4h** and **4i** in 96 and 91% yield, respectively. Good yields were obtained using electron rich 4-chloro anisole (82–83%, **4k**–**l**). Using 4-chloro-*N, N*-dimethylaniline under the standard conditions provided **4j** in 85% yield (cataCXium A as the ligand). Heterocyclic compounds are present in many pharmaceuticals. The heterocyclic catechol derivative **4m** formed in 94% yield (cataCXium A). With cataCXium A, 3-pyridylchloride and 5-chloro-1-methyl-1*H*-indole underwent reaction to give products **4n**–**4p** in 57–84% yield. 4-Benzylpyridines possessing benzyl groups with 4-OMe, 4-F and 3-CF_3_ furnished products in 86–94% yield (**4q**–**4t**) (**4q** and **4r** employed cataCXium A). It is noteworthy that many of these yields are high, despite undergoing two coupling events.

### Monoarylation of diaryl(heteroaryl)methanes

The ability to synthesize tetraarylmethanes with four different aryl groups would provide greater synthetic flexibility. Based on the successful bis-arylation of 4-benzyl pyridines above, we explored the monoarylation of diaryl(4-pyridyl)methanes **5** (Table 4). Triarylmethanes are readily prepared by arylation of diphenylmethane derivatives using our prior approach (see Supplementary Methods)^32^.

Employing diaryl(4-pyridyl)methanes with two phenyl groups or one phenyl and one alkyl substituted aryl, aryl chlorides with alkyl or neutral groups furnished **6a** and **6b** in 97 and 96% yield, respectively. Aryl chlorides with 3-CN, 3-CF_3_, 4-COPh, 4-CN, 4-CF_3_, 4-F and 3-OMe reacted with 89–97% yield (**6c**–**6i**). Aryl chlorides bearing electron-donating groups 4-OMe and 4-NMe_2_, as well as catechol, underwent coupling in 78–92% yield (**6j**–**6l**). Heterocyclic aryl chlorides, including 3-pyridyl, 5-(*N*-methyl indole), and 6-quinolyl participated in the coupling with cataCXium A in 73–96% yield (**6m**–**6r**). Diaryl(4-pyridyl)methanes with a phenyl and 3,5-bis-CF_3_ aryl groups substituted aryl also participated in DCCP to produce the desired products **6s** and **6t** in 77 and 91% yield, respectively. In addition to the high yields generally observed in the synthesis of tetraarylmethane derivatives, it is noteworthy that many of the products contain chiral quaternary centres.

Due to the significance of heterocycles in drug discovery and in material science, we chose diphenyl(2-benzothiazolyl)methane (**8a**), diphenyl(2-benzoxazolyl)methane (**8b**) prepared from 2-methylthiazole (**7a**), 2-methylbenzoxazole (**7b**) using the literature approach^24^ (see General Methods A and Supplementary Methods) and diphenyl(2-pyridyl)methane (**8c**) to expand the scope of our tetraarylmethane synthesis (Table 5). Heteroaryl chlorides such as 3-chloropyridine and 6-chloroquinoline underwent coupling with diphenyl(heteroaryl)methanes (**8**), to furnish tetraarylmethane derivatives (**9a**, **9b**, **9e** and **9f**) in high isolated yields (86–94%). In addition, 5-chlorobenzothiophene and 1-(4-chlorophenyl)-1*H*-pyrrole proved to be suitable aryl chlorides, furnishing products **9c** and **9d** in 61 and 63% yield, respectively. Diphenyl(2-pyridyl)methane coupled with 4-chlorotoluene to give **9g** in 93% yield. With this catalyst system, less acidic diphenyl(3-pyridyl)methane did not afford the desired product under the optimized reaction conditions.

**Figure 3:**
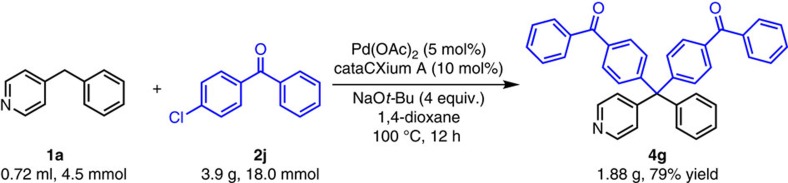
Reaction on gram scale. Diarylation of 4-benzylpyridine with 4-chlorobenzophenone on gram scale.

To demonstrate the potential utility of our method, we performed a gram scale reaction of 4-benzylpyridine **1a** with 4-chlorobenzophenone **2j**. The desired product **4g** was obtained in 79% yield (1.88 g, Fig. 3), demonstrating the reaction is scalable (see Supplementary Methods).

**Figure 4:**
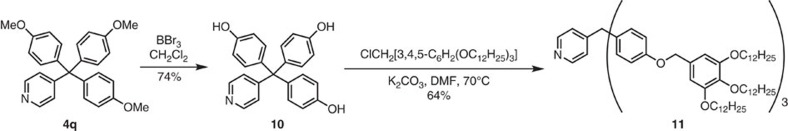
Transformation of reaction product. Synthesis of liquid crystal former **11**.

Kato and co-workers reported a liquid-crystalline bowl-shaped molecule that form columnar and micellar cubic structures, using triary(4-pyridyl)methane moieties as building blocks^18^. In order to apply our method, we synthesized the same compound (Fig. 4) from the reaction product **4q** (Table 3). It is noteworthy that Kato synthesized triary(4-pyridyl)methane was based on Friedel-Crafts arylations from diaryl(4-pyridyl)methane with 46% yield, which is less than our method (91% yield, see Supplementary Methods).

## Discussion

Our research team has been interested in the catalytic functionalization of weakly acidic sp^3^-hybridized C–H bonds through a DCCP. These reactions involve a weakly acidic C–H of the substrate (pronucleophile) that is reversibly deprotonated under the reaction conditions. Subsequently, the nucleophile undergoes catalyst promoted arylation^33^,^34^,^35^,^36^ or vinylation^37^. This method has been particularly successful with the generation of triarylmethanes^38^,^39^,^40^. Tetraarylmethane derivatives are challenging to efficiently prepare by both classical and state-of-the-art methods. The breadth of their applications has outpaced chemists' ability to prepare them in a concise fashion. Cross-coupling methods represent an appealing approach to tetraarylmethane derivatives, but to date successful reports of such processes are still lacking. Outlined herein is a palladium-catalysed DCCP for direct arylation of aryl(heteroaryl)methanes and diaryl(heteroaryl)methanes with aryl chlorides to provide triaryl(heteroaryl)methanes. Unlike traditional cross-coupling procedures, which employ prefunctionalized coupling partners, our approach relies on reversible deprotonation of the diarylmethane derivatives under the conditions used for the catalytic C–C bond forming reaction. Under our reaction conditions, a variety of triaryl(heteroaryl)methanes were prepared in good to excellent yields. This communication represents the first steps towards our goal of developing metal catalysed approaches for the construction of a wide range of tetraarylmethanes.

## Methods

### General procedure A

An oven-dried 8.0 ml reaction vial equipped with a stir bar was charged with 2-methylthiazole (**7a**, 0.50 mmol, 1.0 equiv) or 2-methylbenzoxazole (**7b**, 0.50 mmol, 1.0 equiv) and chlorobenzene (**2e**, 3.0 equiv) in a glove box under a nitrogen atmosphere at room temperature. A stock solution containing Pd(OAc)_2_ (5.6 mg, 0.025 mmol, 5 mol%) and PCy_3_ (14.0 mg, 0.05 mmol, 10 mol%) in dry *o*-xylene (2.5 ml). Then, NaO*t-*Bu (3.0 equiv) was added to the reaction mixture. The vial was capped, removed from the glove box, and heated to 130 °C for 12 h with stirring. The reaction mixture was quenched with 0.5 ml of H_2_O, diluted with 10 ml of ethyl acetate, and filtered over a pad of MgSO_4_ and silica. The pad was rinsed with ethyl acetate (10 × 2 ml) and the combined solutions were concentrated *in vacuo*. The crude material was loaded onto a deactivated silica gel column and purified by flash chromatography to afford the desired products **8a** and **8b** in 81 and 49% yield, respectively.

### General procedure B

An oven-dried 8 ml reaction vial equipped with a stir bar was charged with aryl(4-pyridyl)methanes (**1**, 0.10 mmol, 1.0 equiv) or diaryl(heteroaryl)methanes (**5**, 0.10 mmol, or **8**, 0.20 mmol, 1.0 equiv) and aryl chlorides (**2**, 2.0–4.0 equiv) in a glove box under a nitrogen atmosphere at room temperature. A stock solution containing Pd(OAc)_2_ (1.1 mg, 0.005 mmol, 5 mol%) and PCy_3_ (2.8 mg, 0.01 mmol, 10 mol%) in dry THF or cataCXium A (3.6 mg, 0.01 mmol, 10 mol%) in dry 1,4-dioxane was taken up by syringe and added to the reaction vial under nitrogen. Then, NaO*t-*Bu (2.0–4.0 equiv) was added to the reaction mixture. The vial was capped, removed from the glove box, and heated to 100 °C for 12 h with stirring. The reaction mixture was quenched with three drops of H_2_O, diluted with 3 ml of ethyl acetate, and filtered over a pad of MgSO_4_ and silica. The pad was rinsed with ethyl acetate (3 × 2 ml) and the combined solutions were concentrated *in vacuo*. The crude material was loaded onto a deactivated silica gel column and purified by flash chromatography to afford the desired products.

### Data availability

The authors declare that the data supporting the findings of this study are available within the article and its Supplementary Information files. For the experimental procedures and spectroscopic and physical data of compounds, see Supplementary Methods. For ^1^H and ^13^C{^1^H} NMR spectra of compounds, see Supplementary Figs 1–102.

## Additional information

**How to cite this article:** Zhang, S. *et al*. Palladium-catalysed synthesis of triaryl(heteroaryl)methanes. *Nat. Commun.* **8,** 14641 doi: 10.1038/ncomms14641 (2017).

**Publisher's note**: Springer Nature remains neutral with regard to jurisdictional claims in published maps and institutional affiliations.

## Supplementary Material

Supplementary Information Supplementary figures, supplementary table, supplementary methods and supplementary references.

Peer review file

## Figures and Tables

**Table 1:**
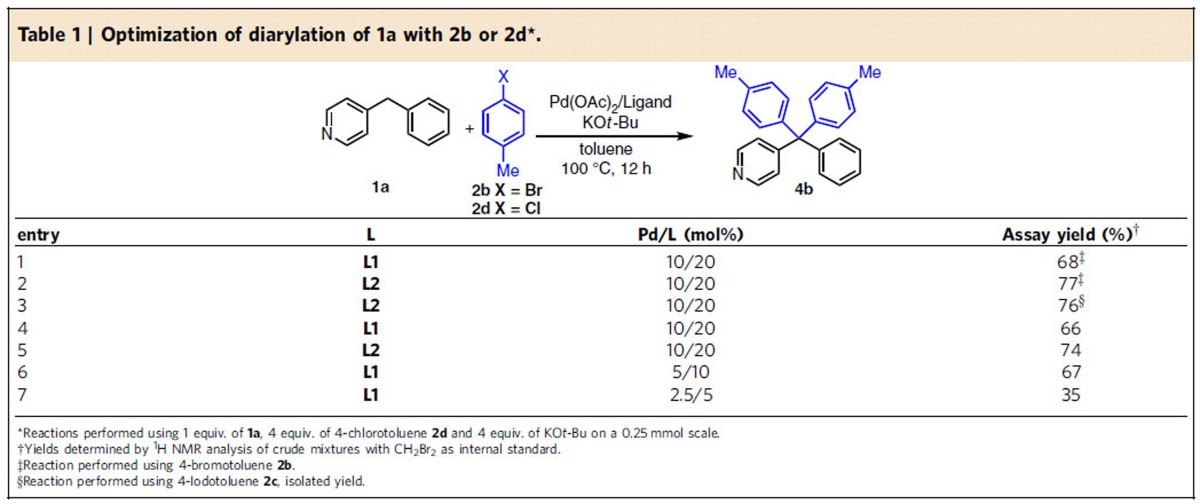
Optimization of diarylation of 1a with 2b or 2d*.

*Reaction conditions:**1**/LiHMDS/**2**/[Pd(*η*^3^-C_3_H_5_)Cl]_2_/S-IPr·HCl=200/200/100/2.5/5; 0.1 M of ketone **1**; T=30^o^C; B/L and *dr* was determined by ^1^H NMR, *dr* is the ratio of (±)-(*syn,anti*)-**3**/other diastereoisomers; Isolated yield. †T=50 ^o^C. ‡Solvent=THF. §OBoc of **2** was replaced with OP(OEt)_2_. ||The yield was determined by ^1^H NMR.

**Table 2:**
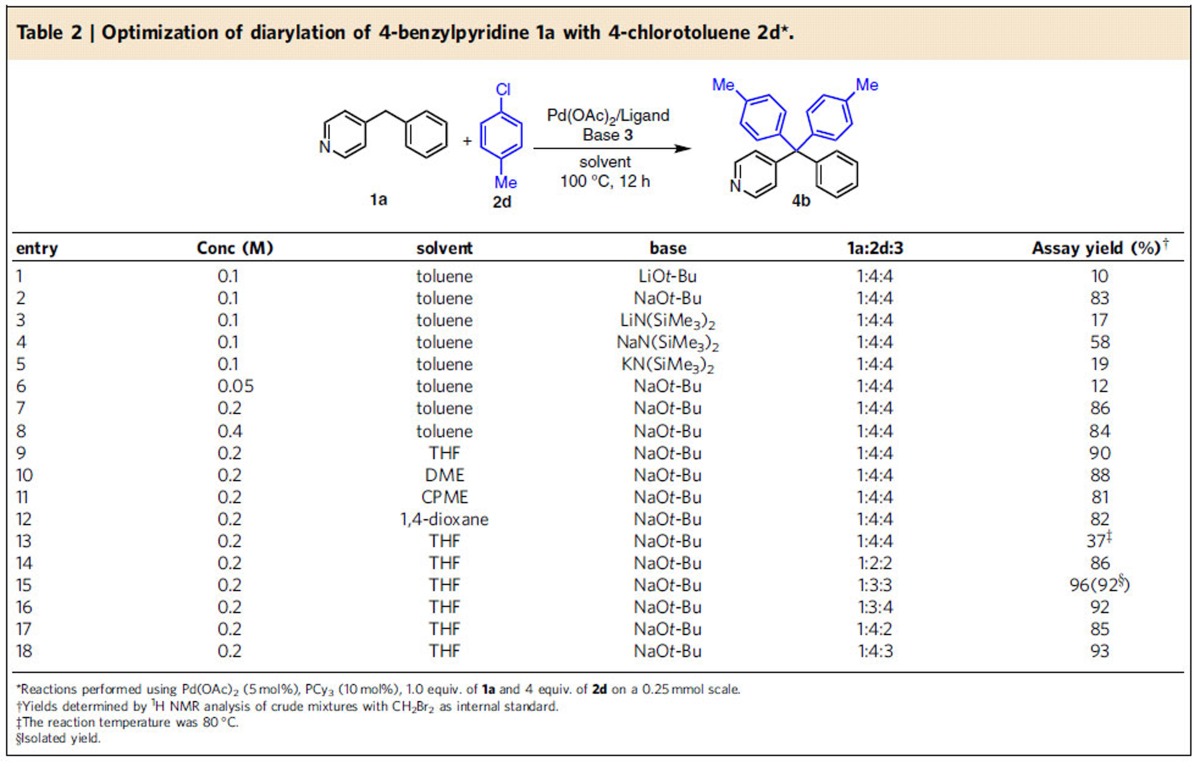
Optimization of diarylation of 4-benzylpyridine 1a with 4-chlorotoluene 2d*.

*Reaction conditions:**1**/LiHMDS/**2**/[Pd(*η*^3^-C_3_H_5_)Cl]_2_/S-IPr·HCl=200/200/100/2.5/5; 0.1 M of ketone **1**; T=30^o^C; B/L and *dr* was determined by ^1^H NMR, *dr* is the ratio of (±)-(*syn,anti*)-**3**/other diastereoisomers; Isolated yield. †T=50 ^o^C. ‡Solvent=THF. §OBoc of **2** was replaced with OP(OEt)_2_. ||The yield was determined by ^1^H NMR.

**Table 3:**
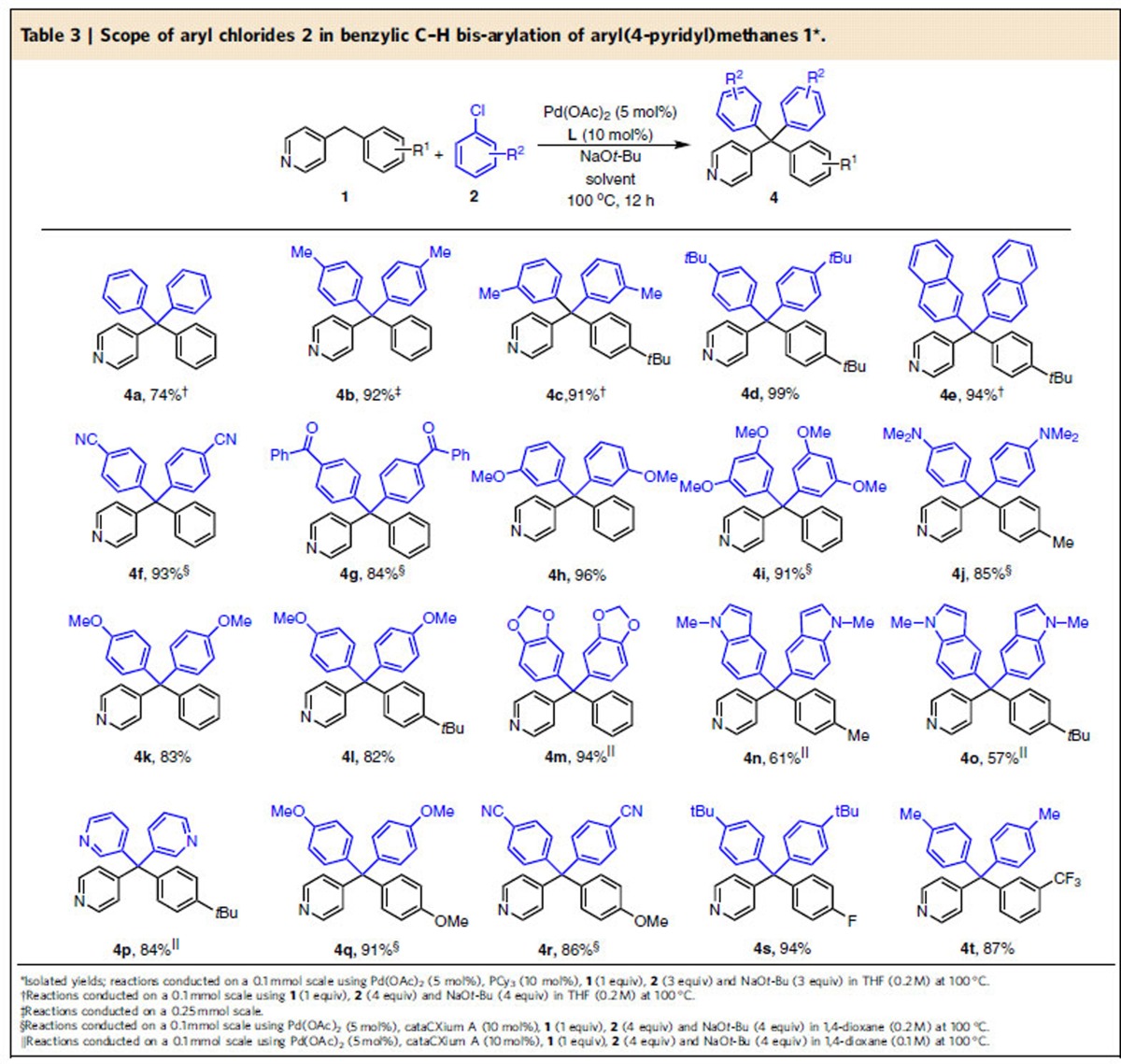
Scope of aryl chlorides 2 in benzylic C–H bis-arylation of aryl(4-pyridyl)methanes 1*.

*Reaction conditions:**1**/LiHMDS/**2**/[Pd(*η*^3^-C_3_H_5_)Cl]_2_/S-IPr·HCl=200/200/100/2.5/5; 0.1 M of ketone **1**; T=30^o^C; B/L and *dr* was determined by ^1^H NMR, *dr* is the ratio of (±)-(*syn,anti*)-**3**/other diastereoisomers; Isolated yield. †T=50 ^o^C. ‡Solvent=THF. §OBoc of **2** was replaced with OP(OEt)_2_. ||The yield was determined by ^1^H NMR.

**Table 4:**
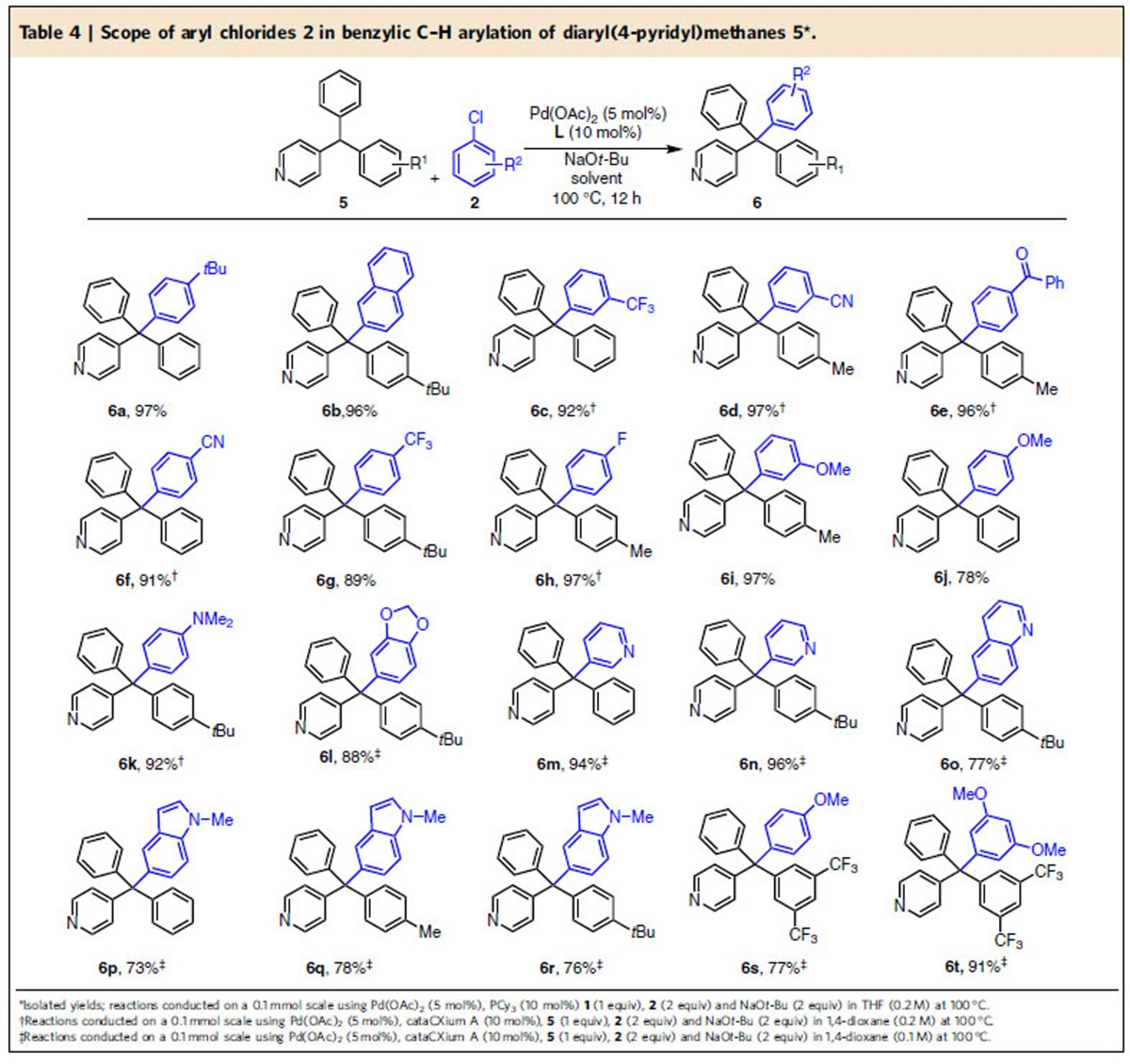
Scope of aryl chlorides 2 in benzylic C–H arylation of diaryl(4-pyridyl)methanes 5*.

*Reaction conditions:**1**/LiHMDS/**2**/[Pd(*η*^3^-C_3_H_5_)Cl]_2_/S-IPr·HCl=200/200/100/2.5/5; 0.1 M of ketone **1**; T=30^o^C; B/L and *dr* was determined by ^1^H NMR, *dr* is the ratio of (±)-(*syn,anti*)-**3**/other diastereoisomers; Isolated yield. †T=50 ^o^C. ‡Solvent=THF. §OBoc of **2** was replaced with OP(OEt)_2_. ||The yield was determined by ^1^H NMR.

**Table 5:**
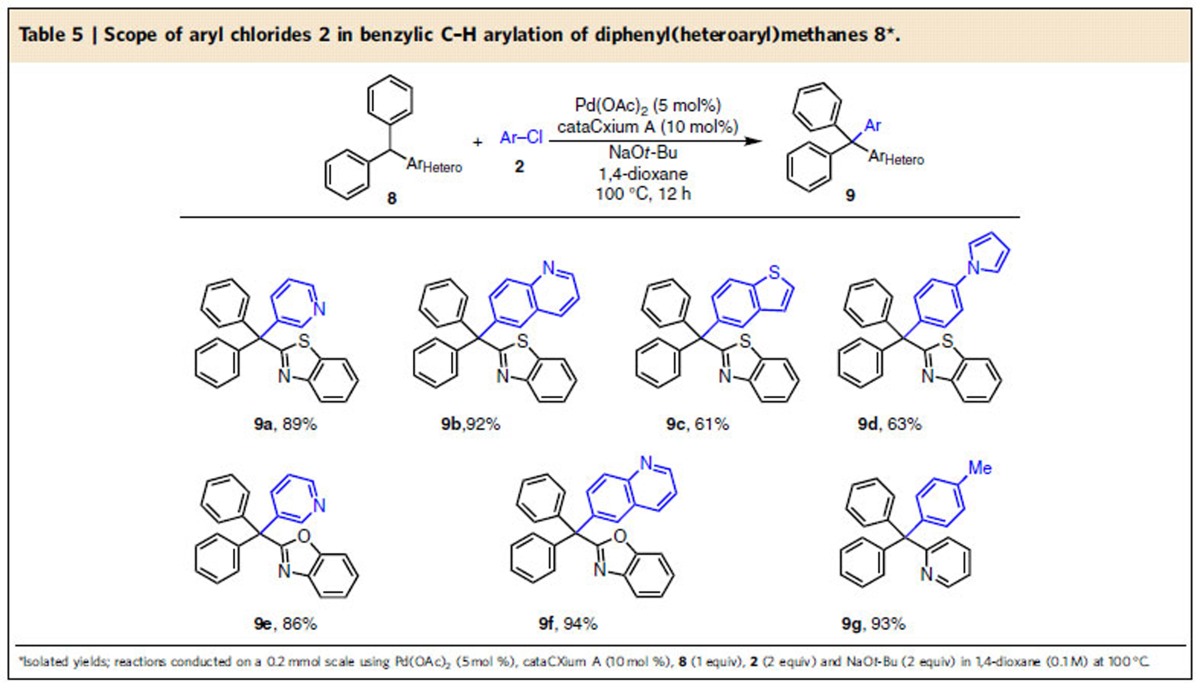
Scope of aryl chlorides 2 in benzylic C–H arylation of diphenyl(heteroaryl)methanes 8*.

*Reaction conditions:**1**/LiHMDS/**2**/[Pd(*η*^3^-C_3_H_5_)Cl]_2_/S-IPr·HCl=200/200/100/2.5/5; 0.1 M of ketone **1**; T=30^o^C; B/L and *dr* was determined by ^1^H NMR, *dr* is the ratio of (±)-(*syn,anti*)-**3**/other diastereoisomers; Isolated yield. †T=50 ^o^C. ‡Solvent=THF. §OBoc of **2** was replaced with OP(OEt)_2_. ||The yield was determined by ^1^H NMR.
