# Supplementary material for: Palladium-catalysed synthesis of triaryl(heteroaryl)methanes
Source: Nat Commun. 2017 Mar 14;8:14641. doi: 10.1038/ncomms14641 (PMC5355892; doi:10.1038/ncomms14641)
Supplement: Supplementary Information — Supplementary figures, supplementary table, supplementary methods and supplementary references. [file ncomms14641-s1.pdf]

Supplementary Figure 1. ^{1}H NMR Spectrum of 5c (500 MHz, $CDCl_3$)

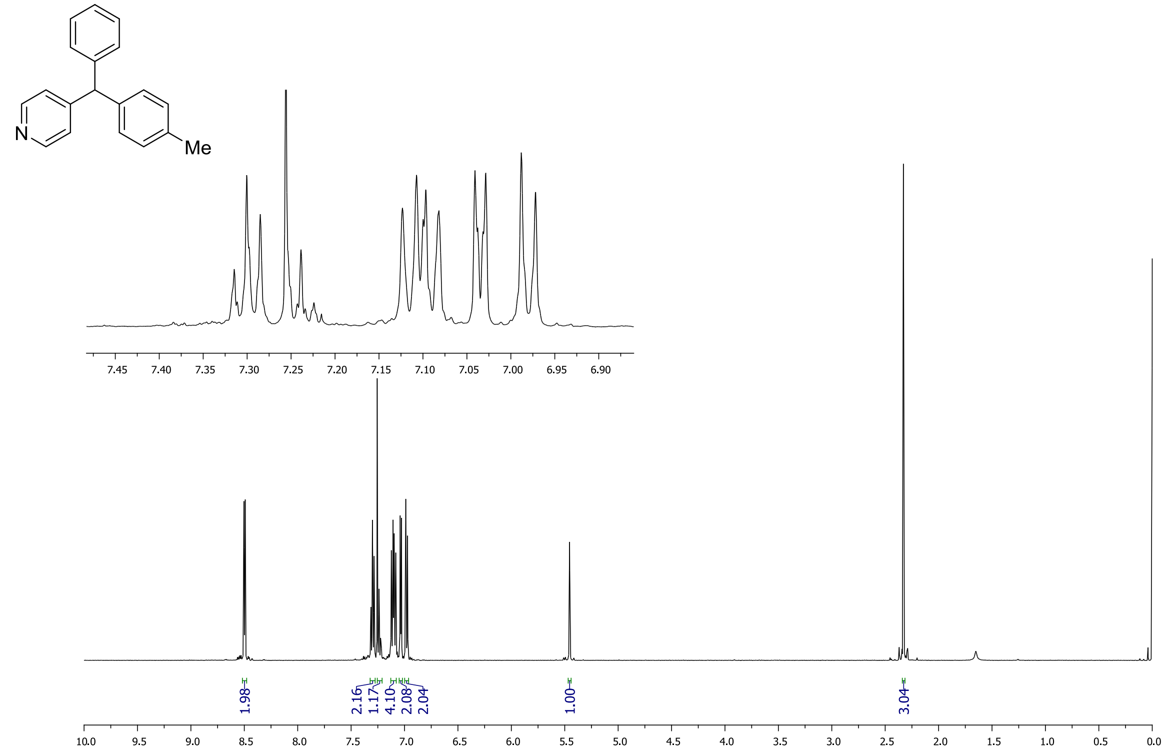

Supplementary Figure 2. ^{13}C NMR Spectrum of 5c (125 MHz, $CDCl_3$)

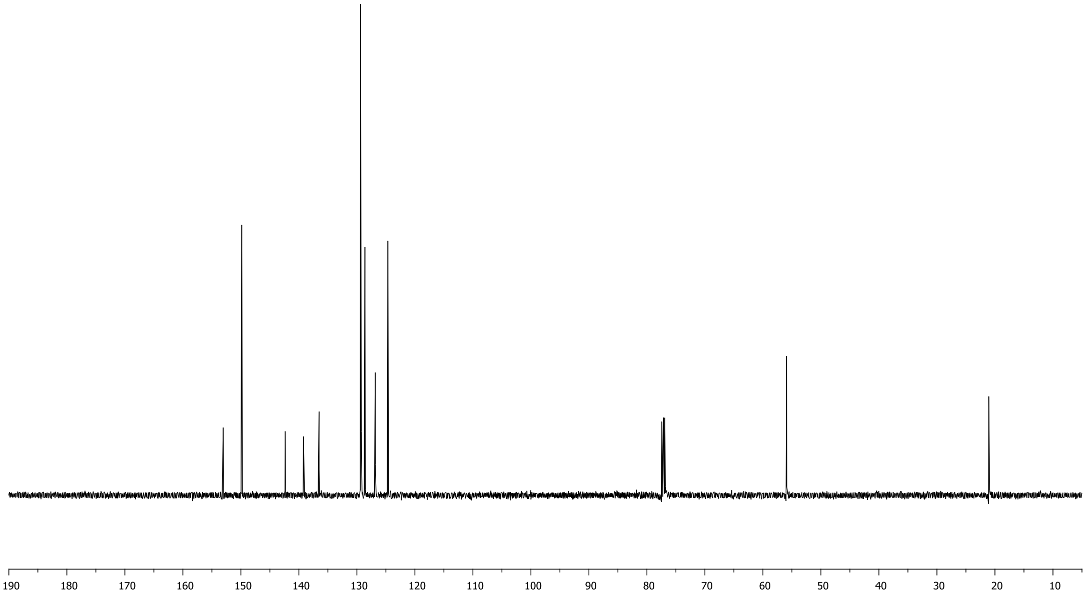

Supplementary Figure 3. ^{1}**H NMR Spectrum of 5d (500 MHz, $CDCl_3$)**

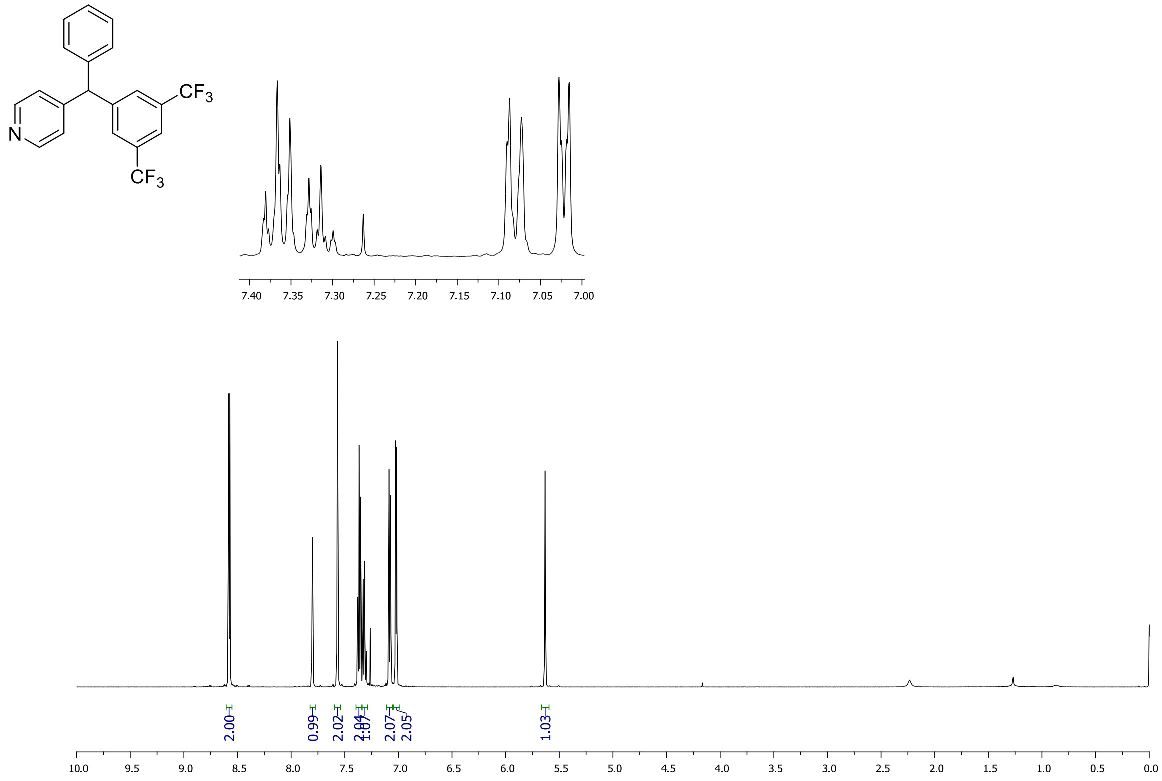

Supplementary Figure 4. ^{13}**C NMR Spectrum of 5d (125 MHz, $CDCl_3$)**

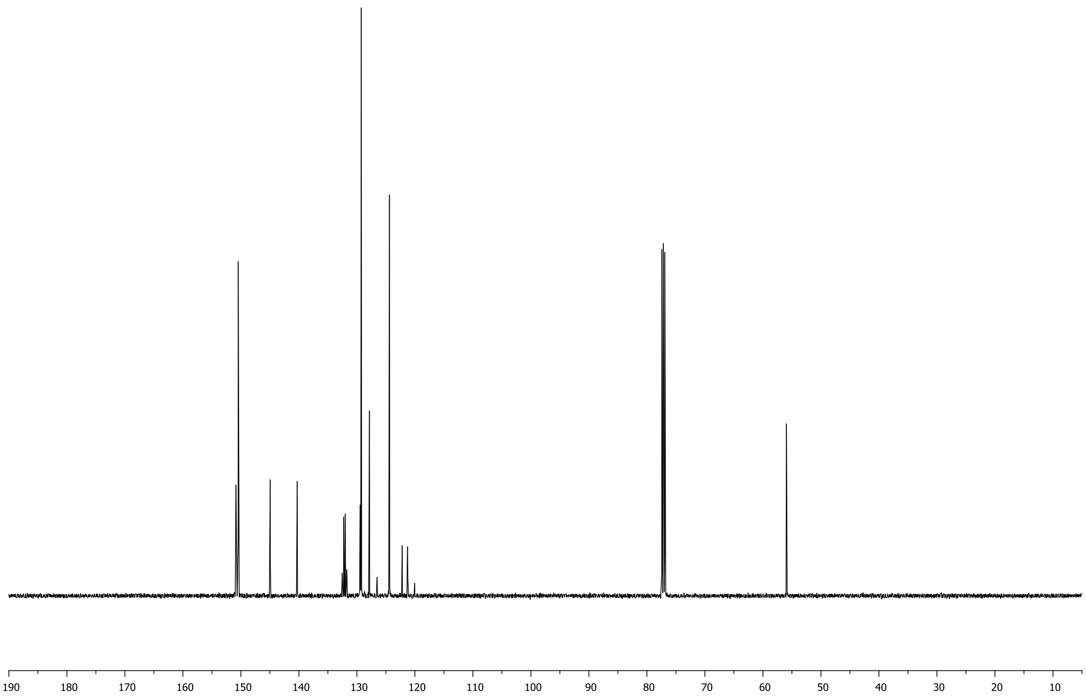

Supplementary Figure 5. ^{1}H NMR Spectrum of 4a (500 MHz, $CDCl_3$)

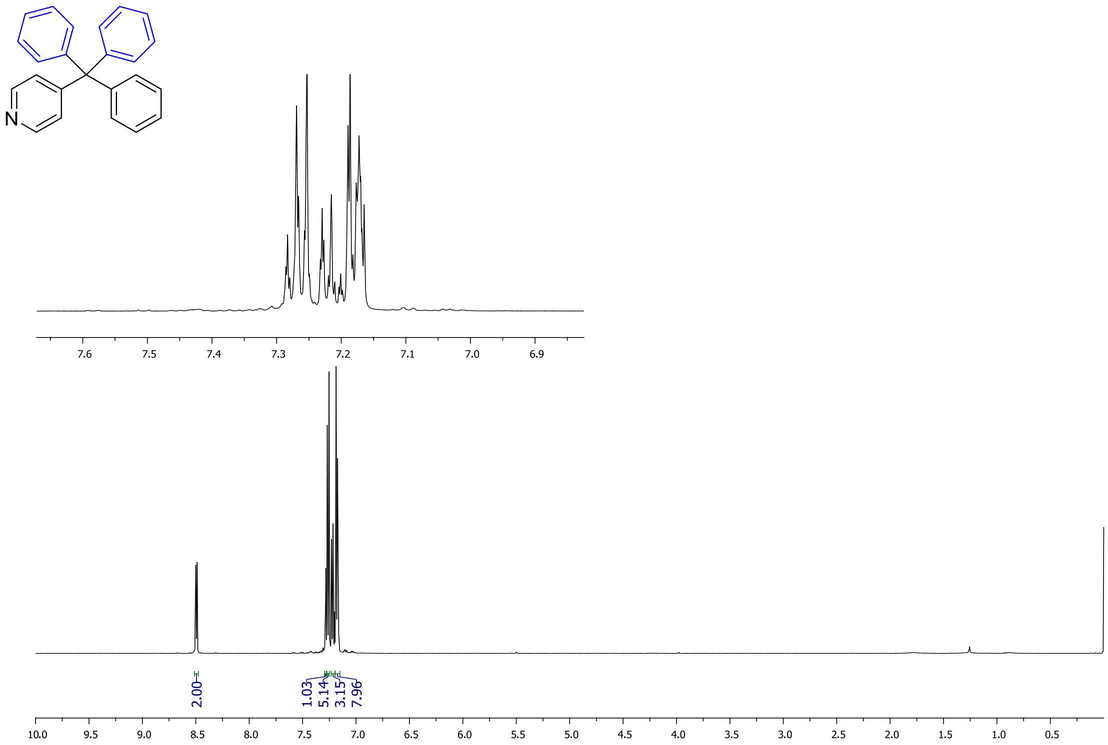

Supplementary Figure 6. ^{13}C NMR Spectrum of 4a (125 MHz, $CDCl_3$)

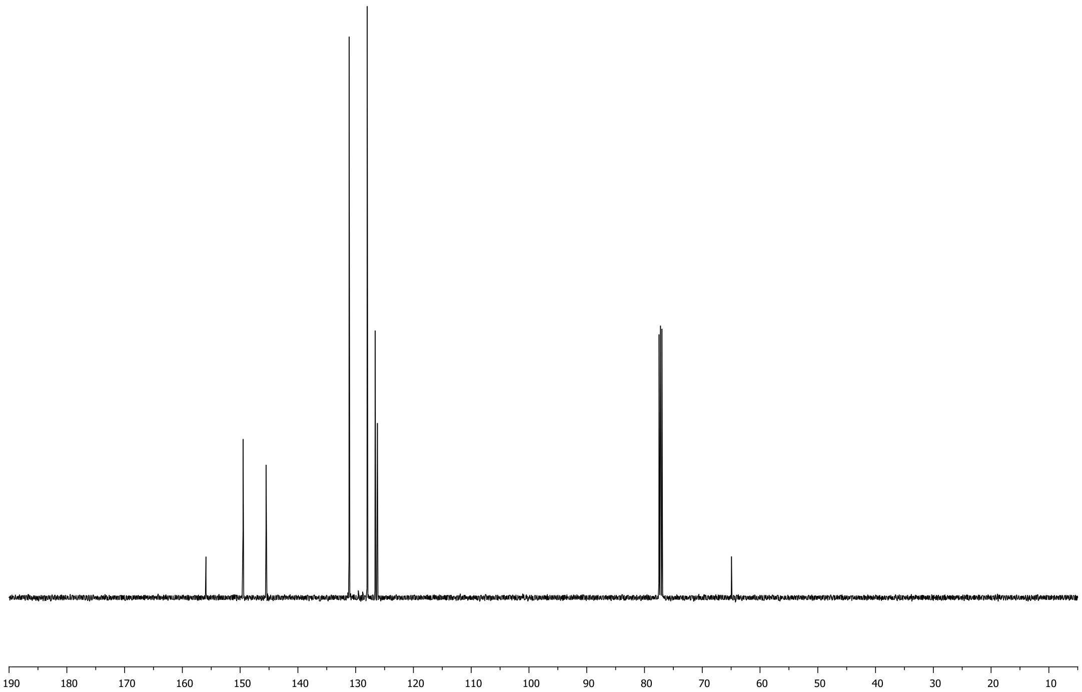

Supplementary Figure 7. ^{1}H NMR Spectrum of 4b (500 MHz, $CDCl_3$)

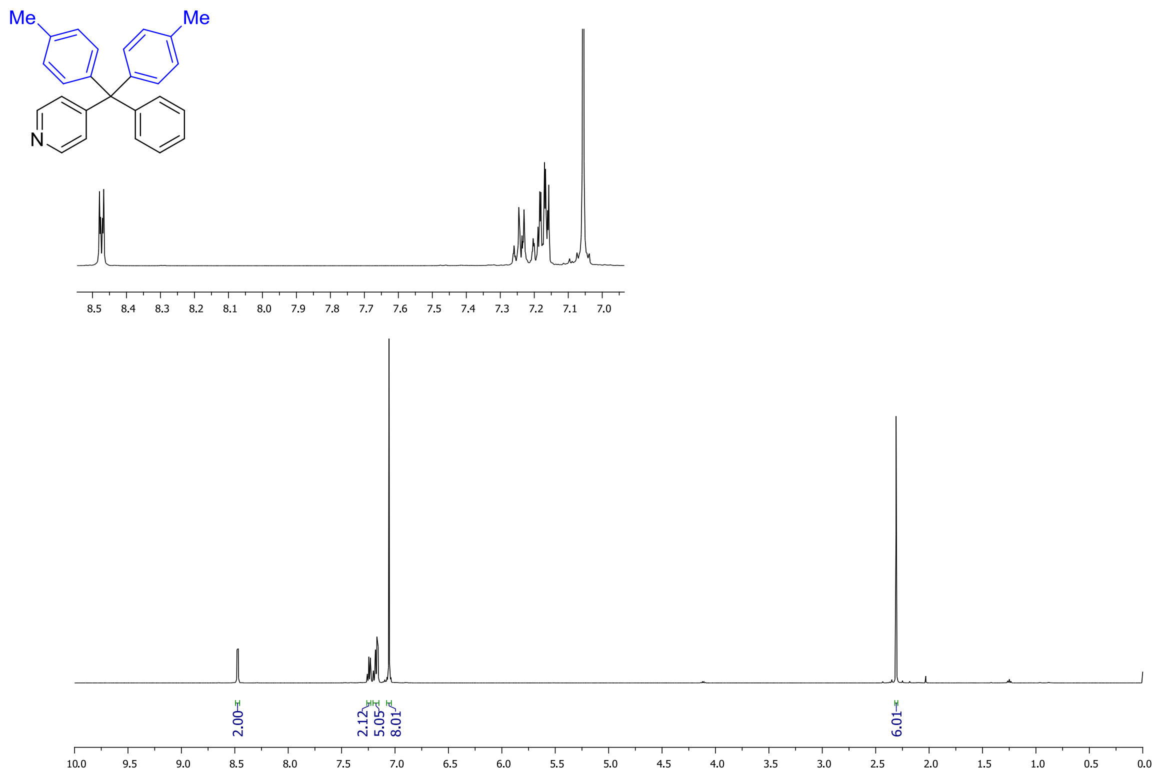

Supplementary Figure 8. ^{13}C NMR Spectrum of 4b (125 MHz, $CDCl_3$)

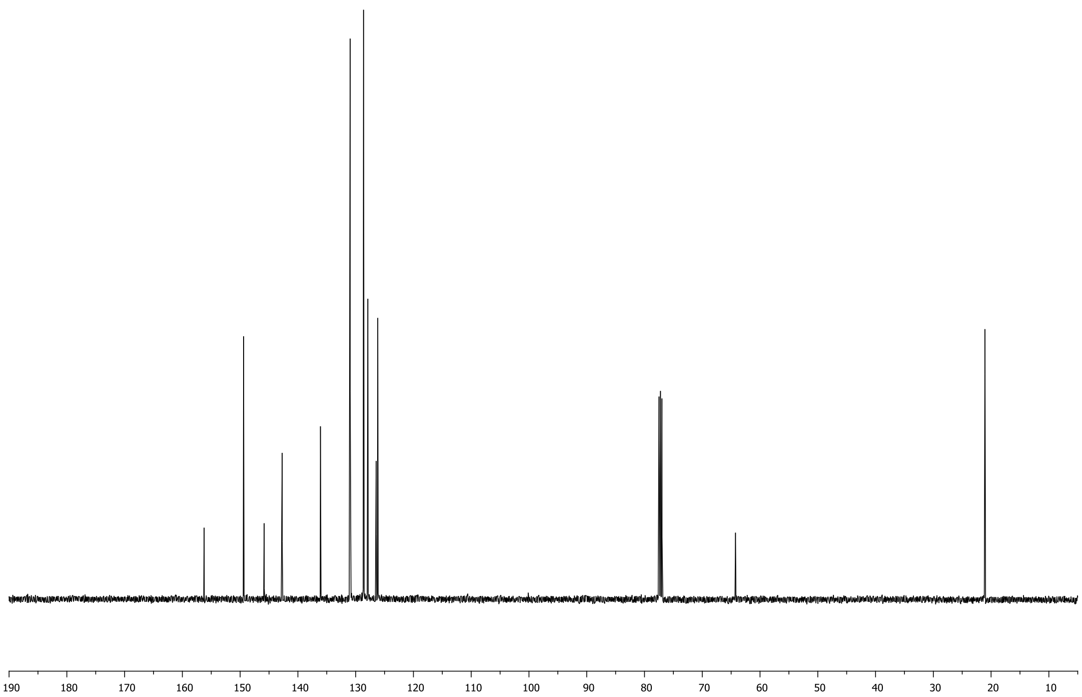

Supplementary Figure 9. ^{1}H NMR Spectrum of 4c (500 MHz, $CDCl_3$)

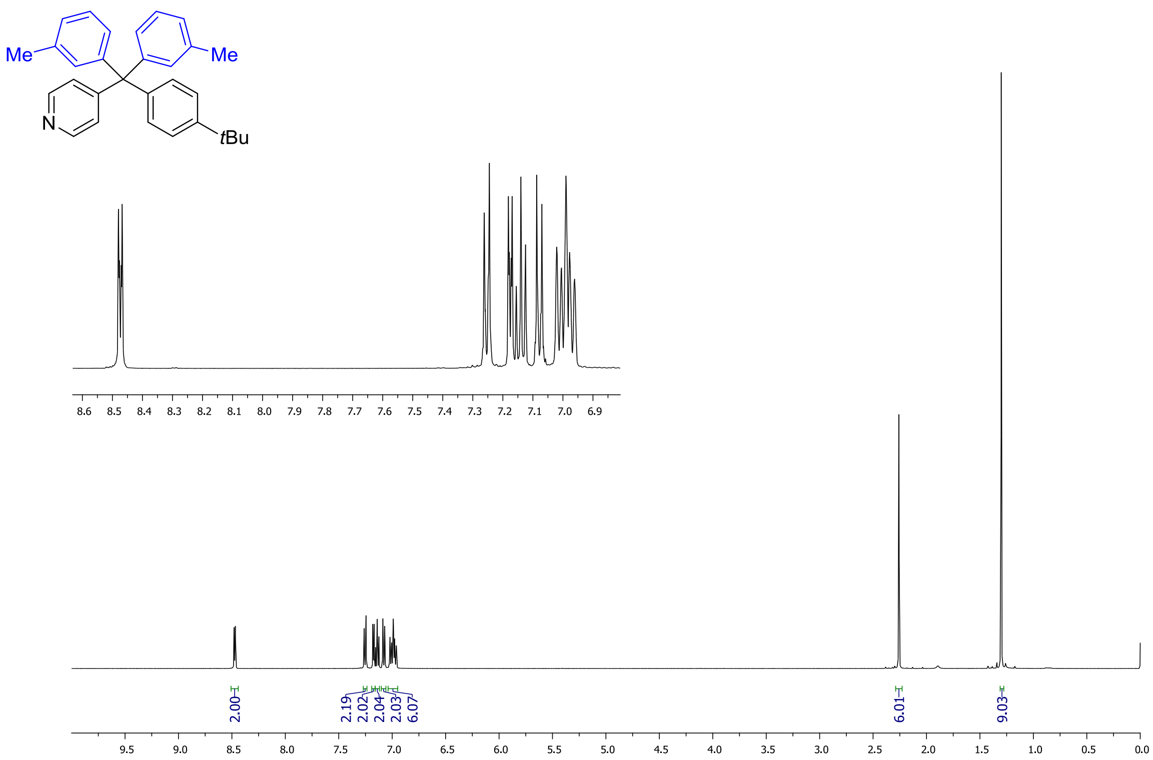

Supplementary Figure 10. ^{13}C NMR Spectrum of 4c (125 MHz, $CDCl_3$)

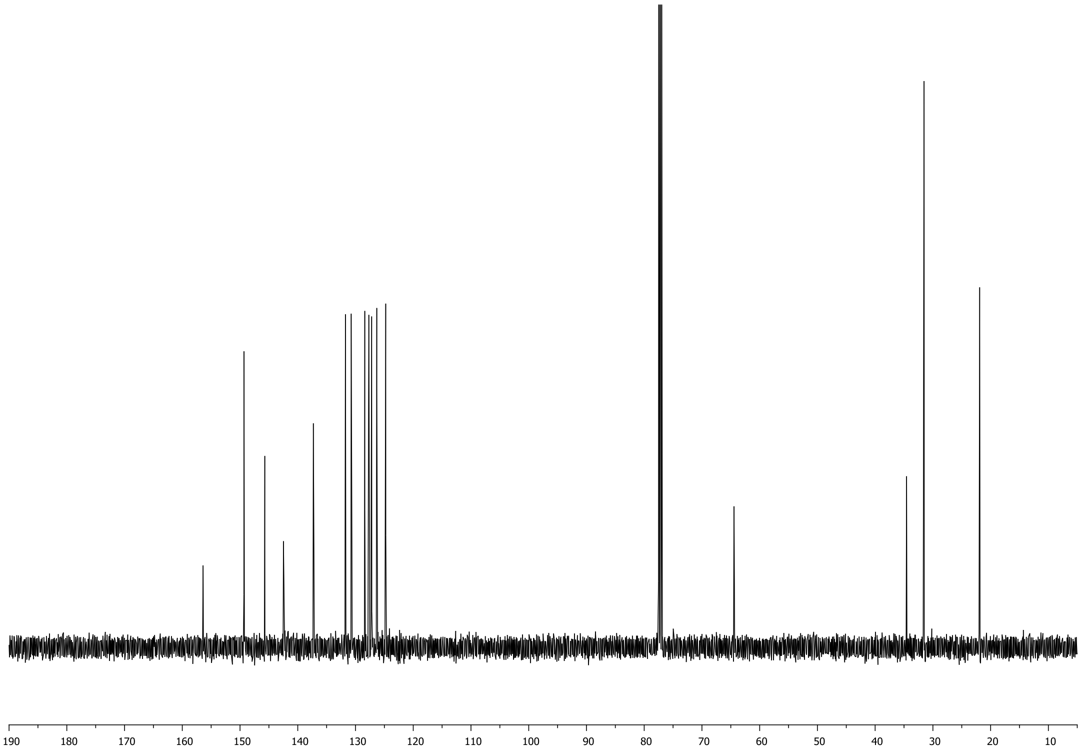

Supplementary Figure 11. ^{1}H NMR Spectrum of 4d (500 MHz, CDCl$_3$)

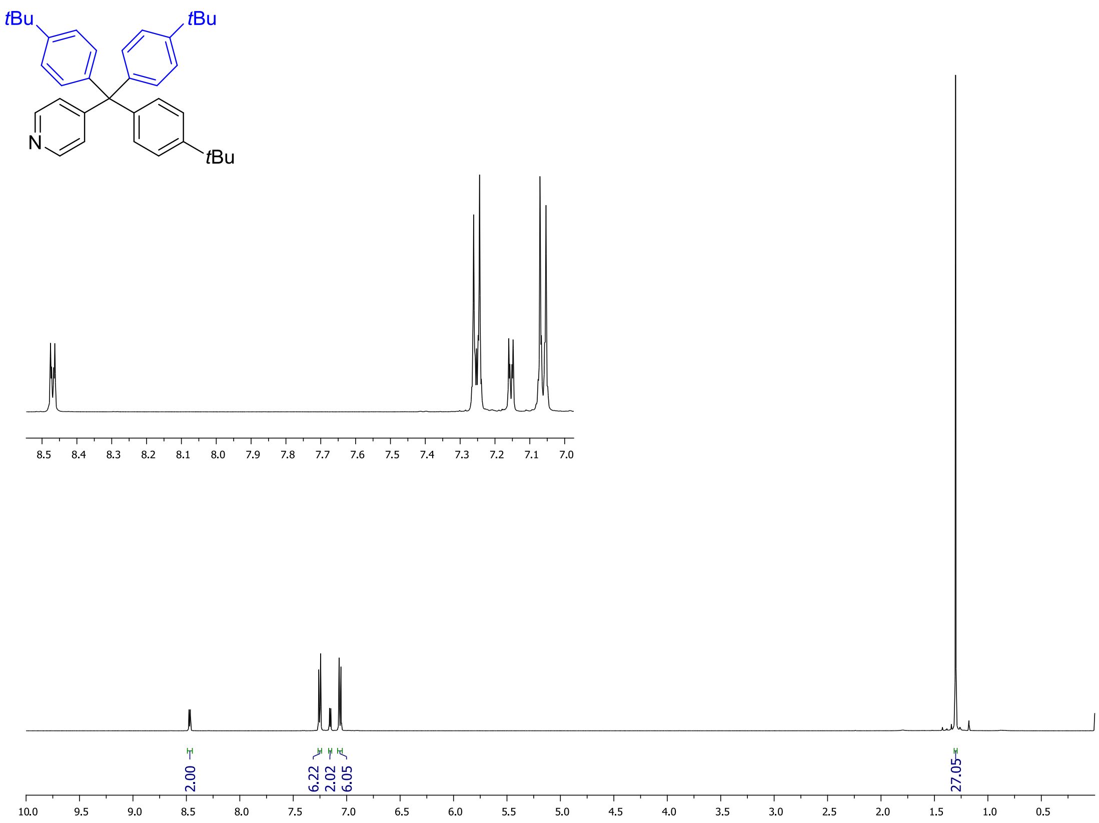

Supplementary Figure 12. ^{13}C NMR Spectrum of 4d (125 MHz, CDCl$_3$)

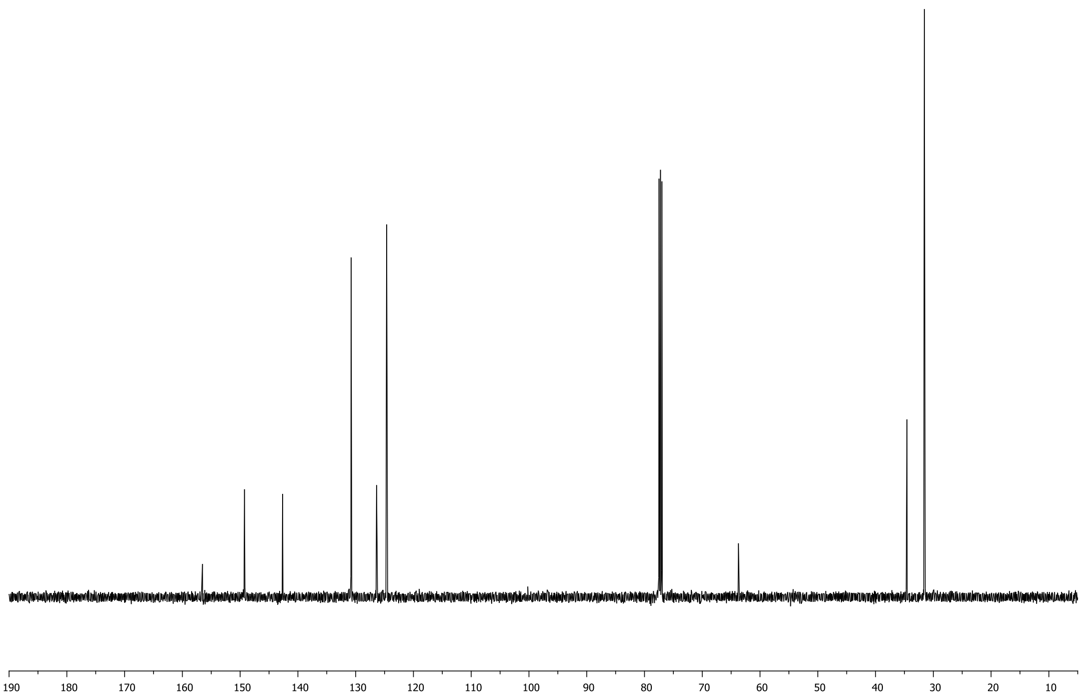

Supplementary Figure 13. ^{1}H NMR Spectrum of 4e (500 MHz, $CDCl_3$)

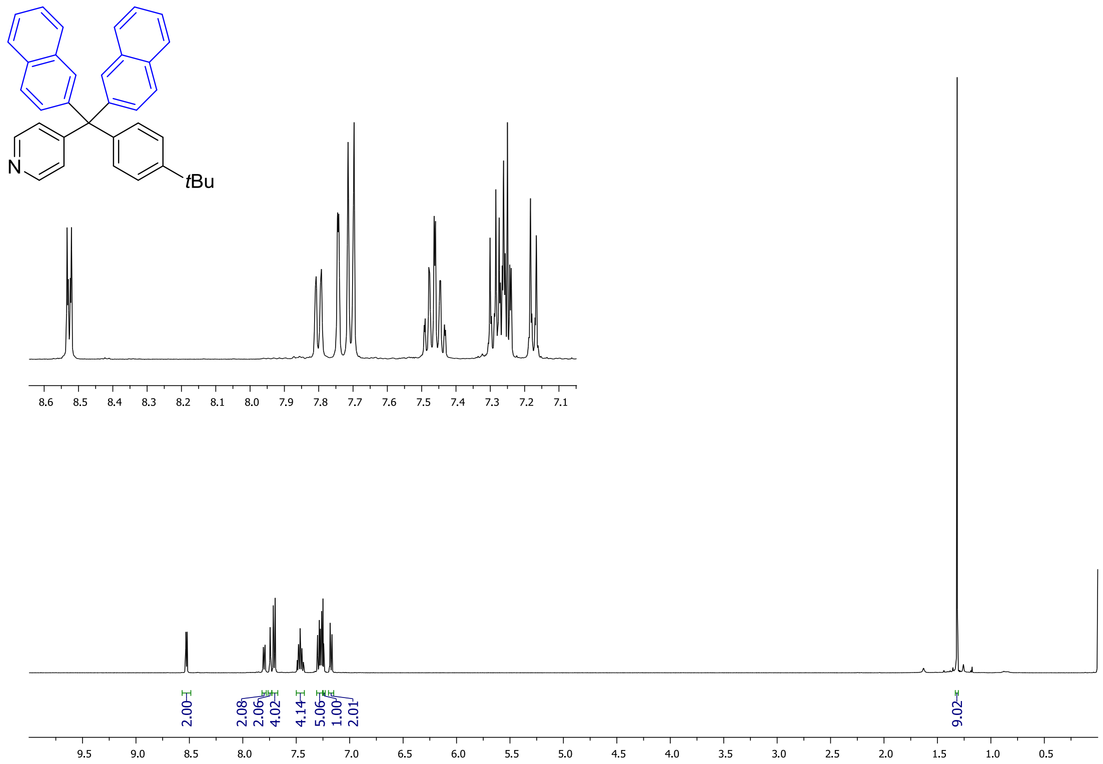

Supplementary Figure 14. ^{13}C NMR Spectrum of 4e (125 MHz, $CDCl_3$)

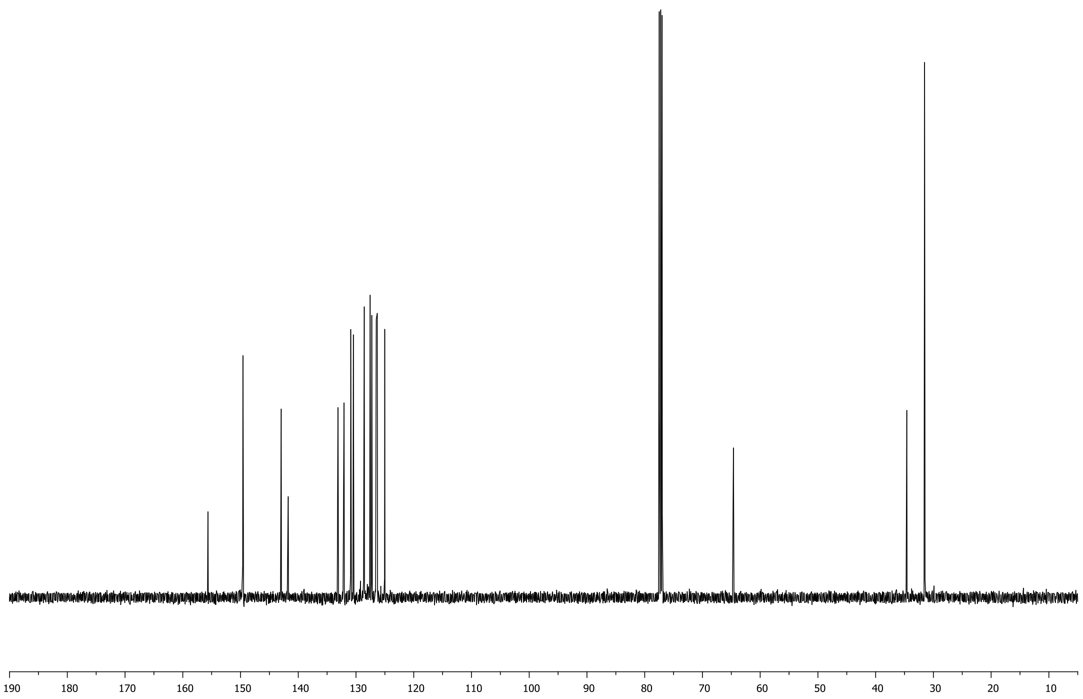

Supplementary Figure 15. ^{1}**H NMR Spectrum of 4f (500 MHz,** $CDCl_3$**)**

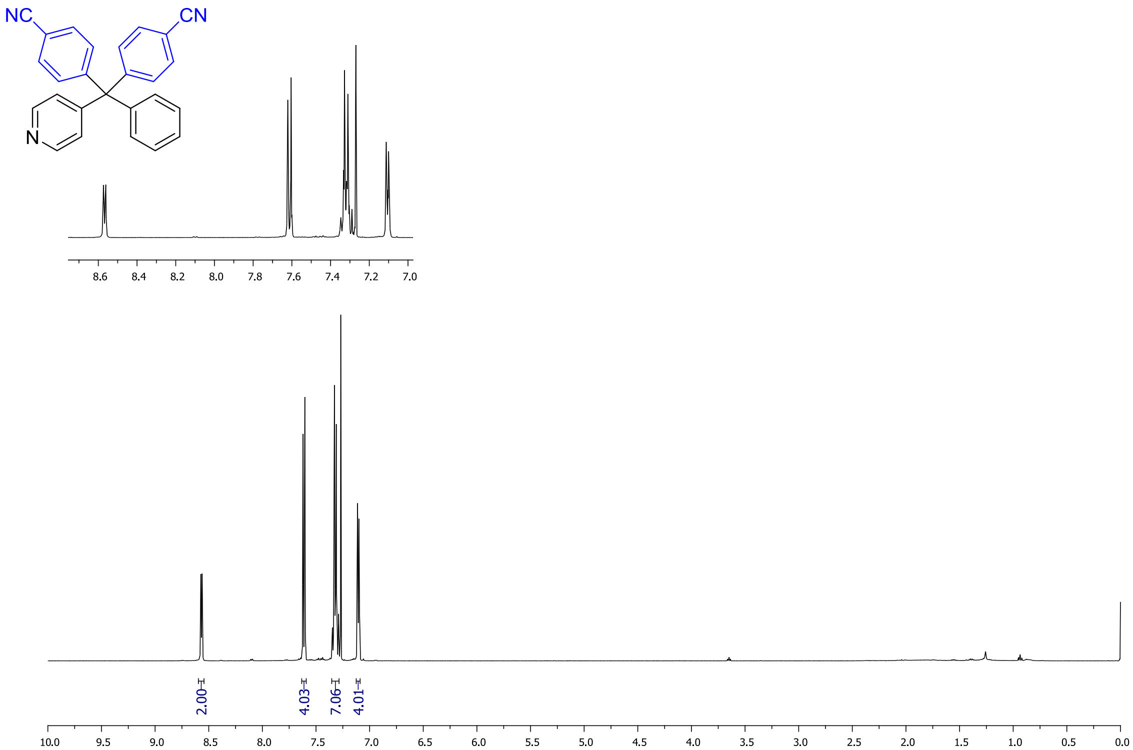

Supplementary Figure 16. ^{13}**C NMR Spectrum of 4f (125 MHz,** $CDCl_3$**)**

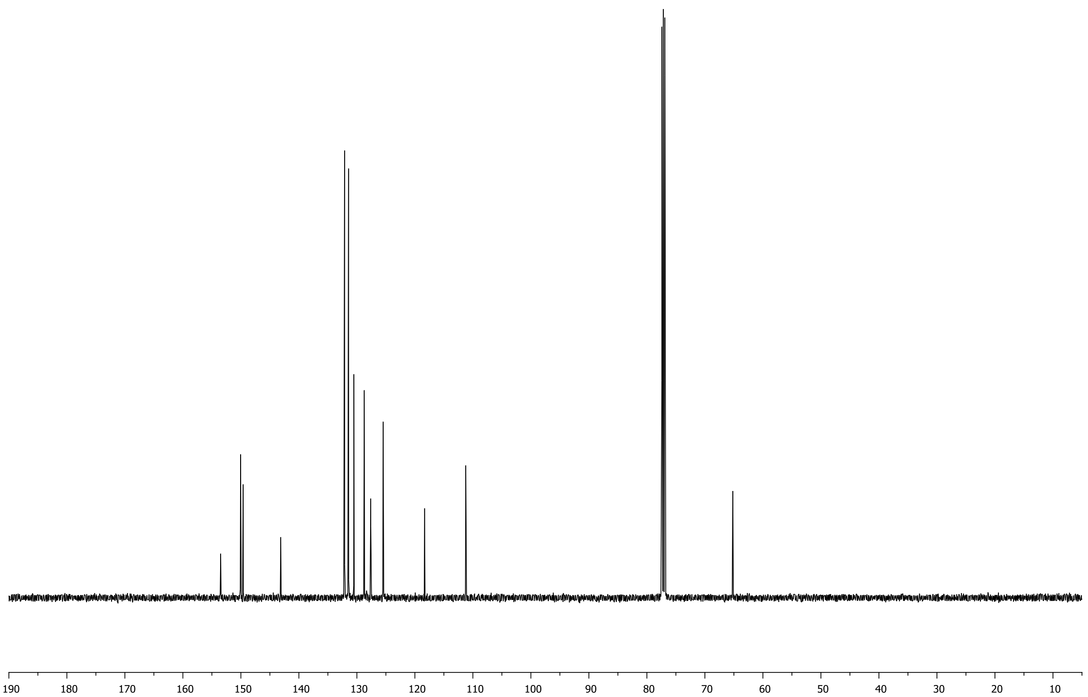

Supplementary Figure 17. ^{1}H NMR Spectrum of 4g (500 MHz, $CDCl_3$)

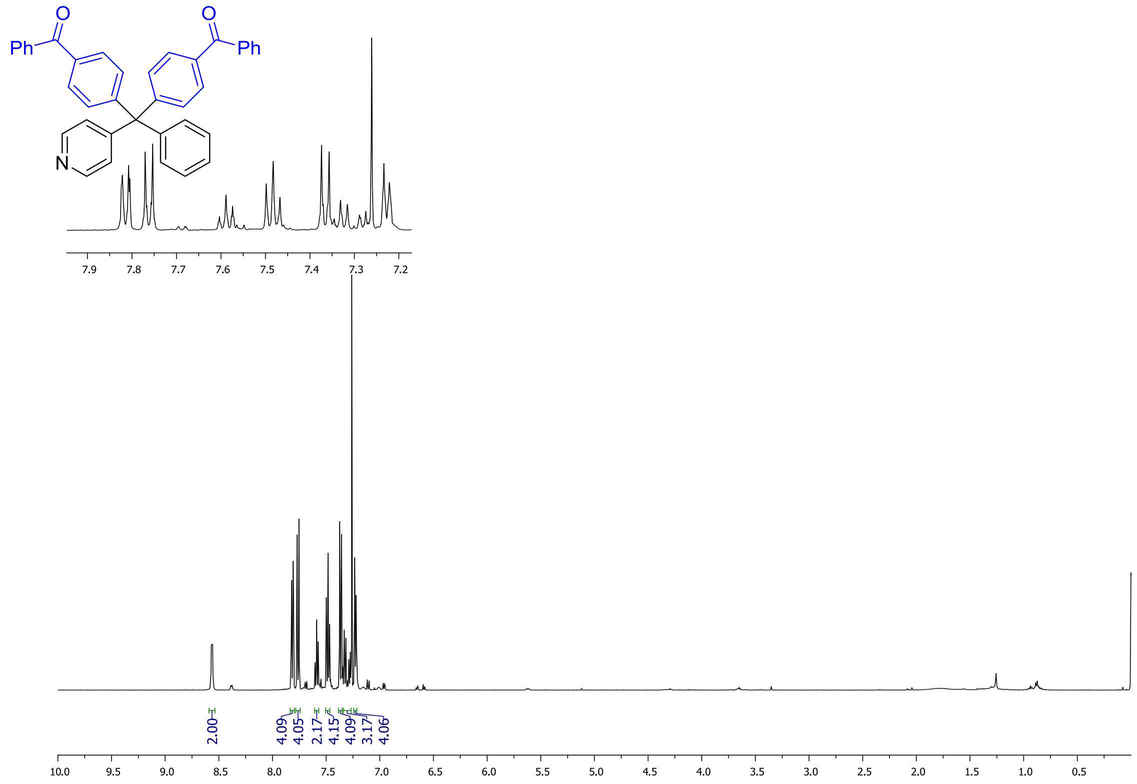

Supplementary Figure 18. ^{13}C NMR Spectrum of 4g (125 MHz, $CDCl_3$)

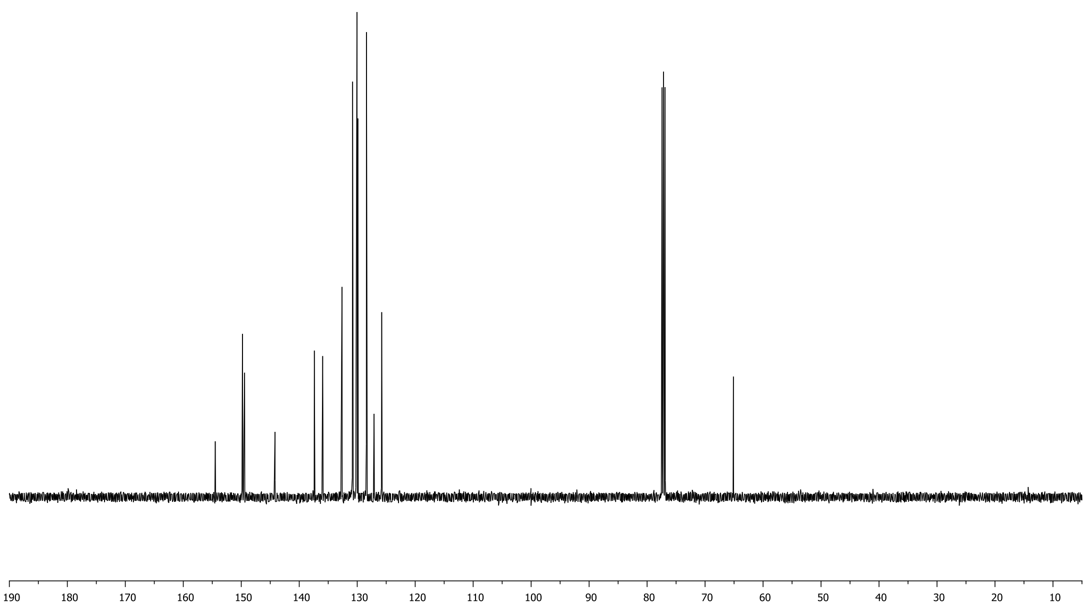

Supplementary Figure 19. ^{1}H NMR Spectrum of 4h (500 MHz, $CDCl_3$)

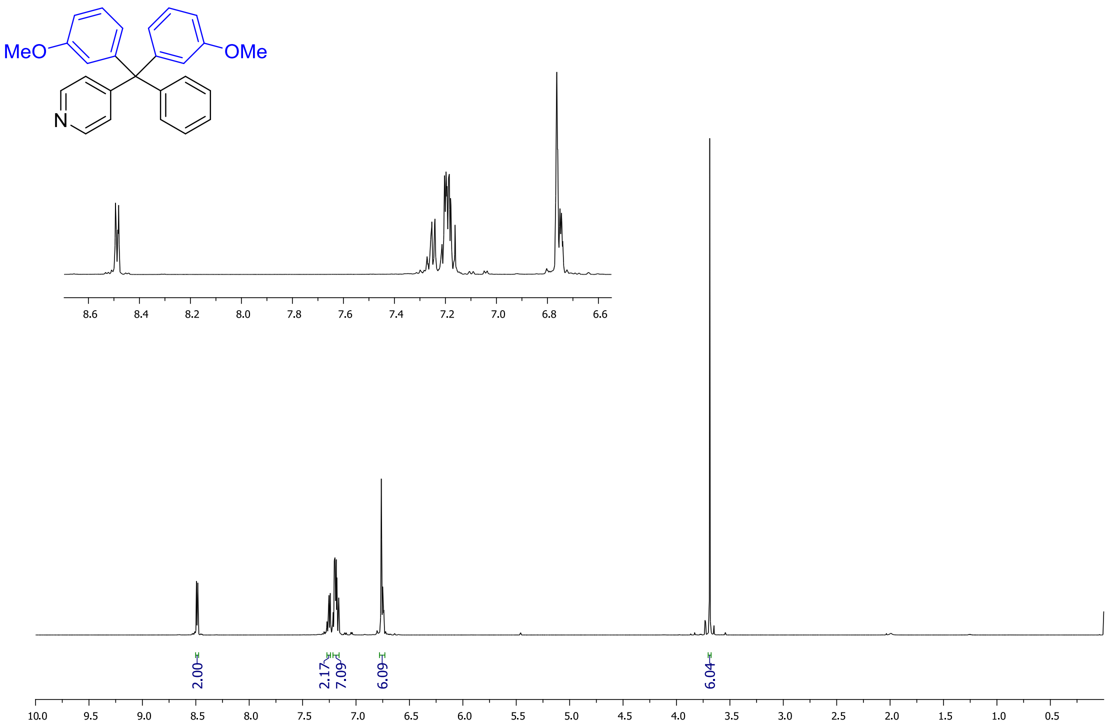

Supplementary Figure 20. ^{13}C NMR Spectrum of 4h (125 MHz, $CDCl_3$)

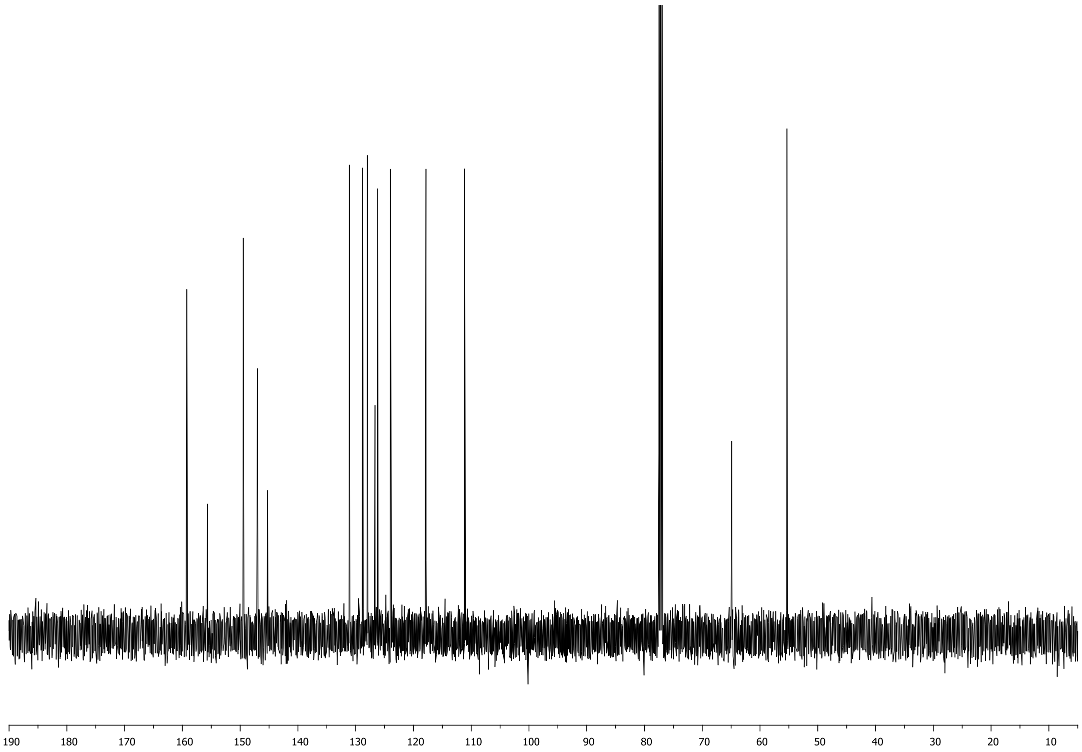

Supplementary Figure 21. ^{1}H NMR Spectrum of 4i (500 MHz, $CDCl_3$)

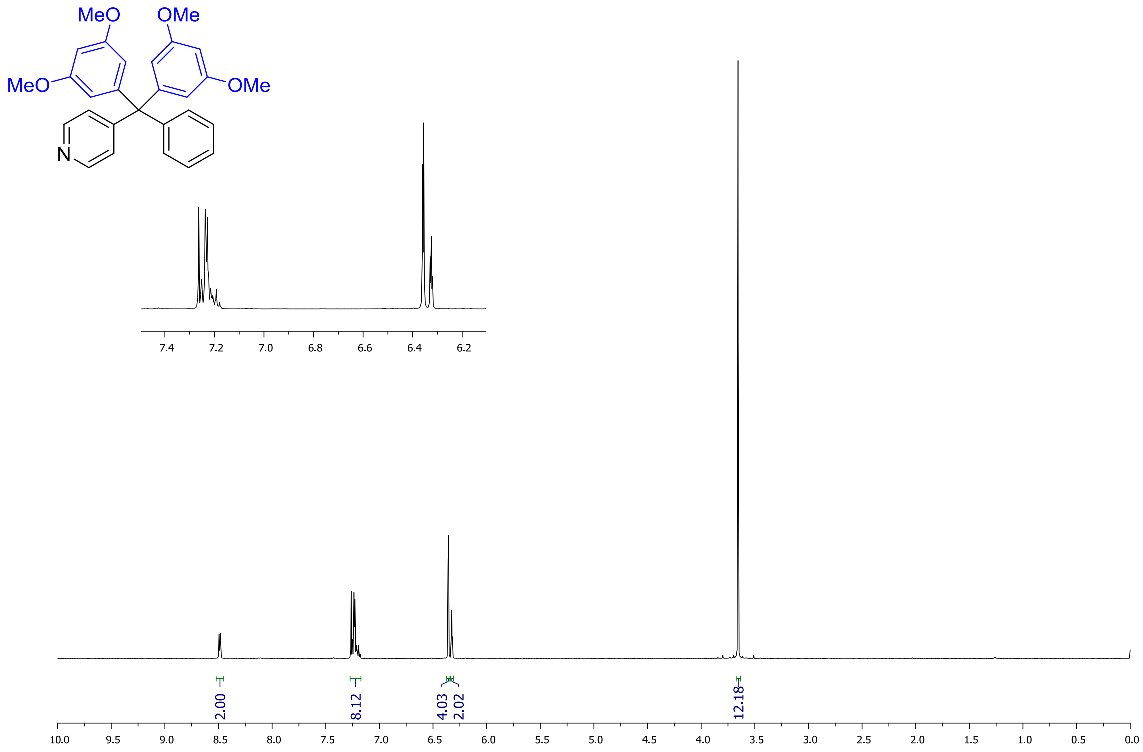

Supplementary Figure 22. ^{13}C NMR Spectrum of 4i (125 MHz, $CDCl_3$)

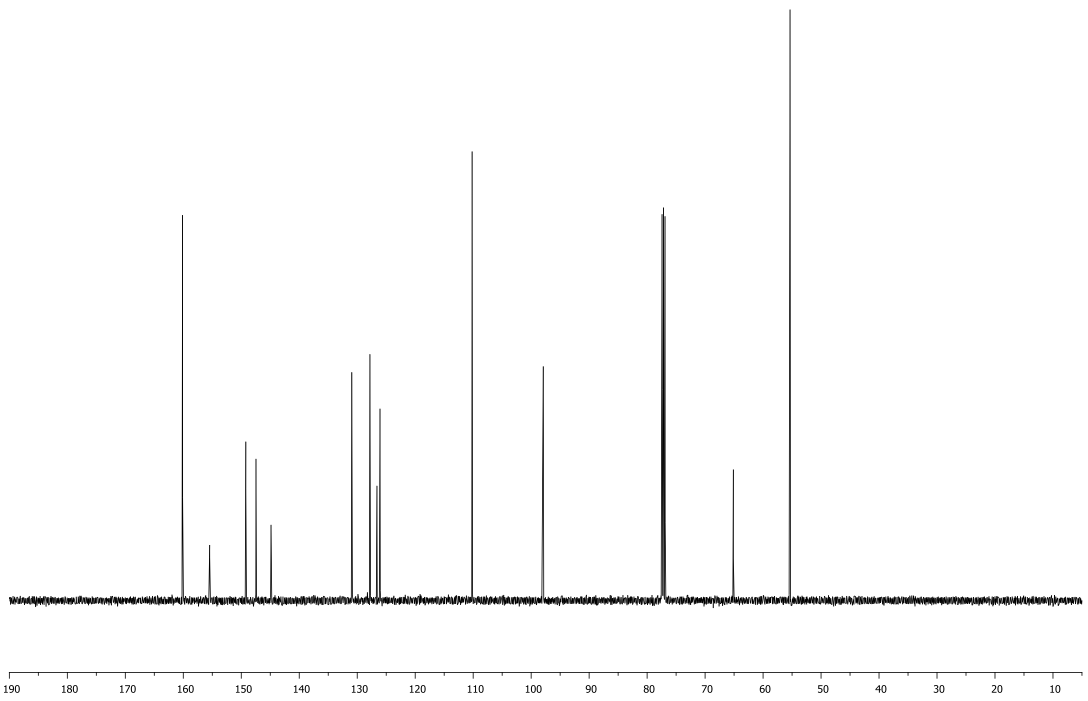

Supplementary Figure 23. ^{1}**H NMR Spectrum of 4j (500 MHz, CDCl$_3$)**

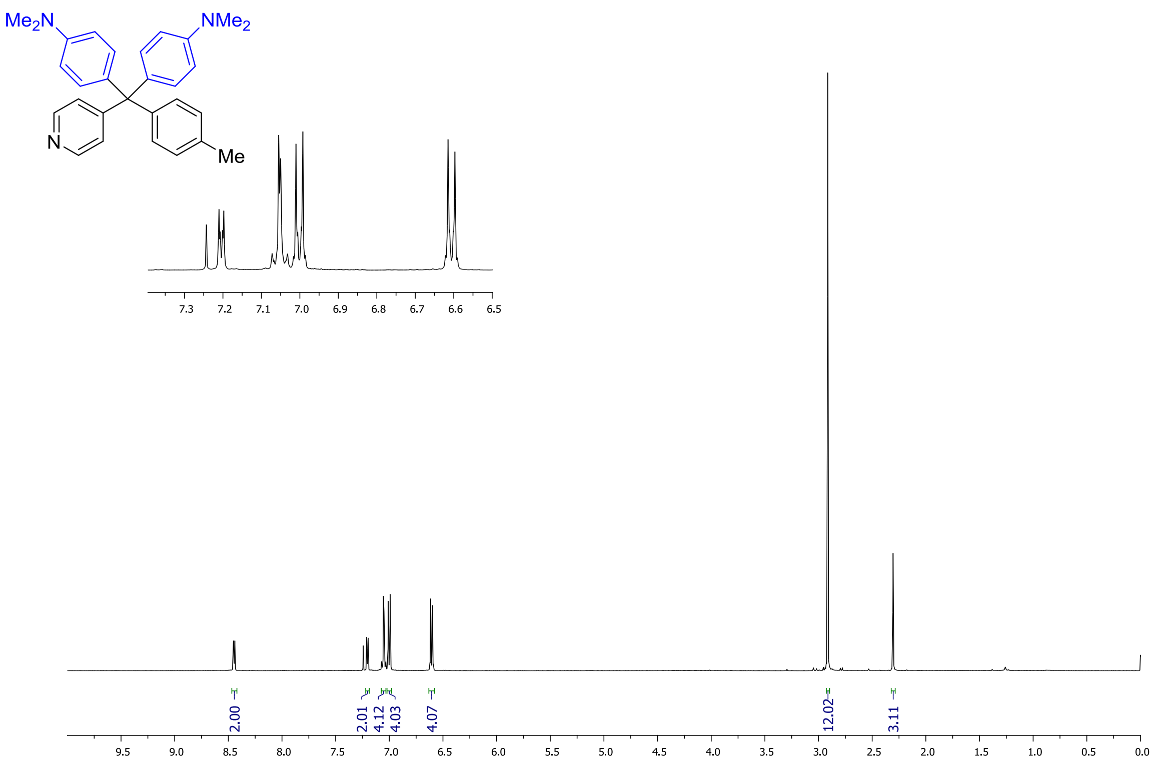

Supplementary Figure 24. ^{13}**C NMR Spectrum of 4j (125 MHz, CDCl$_3$)**

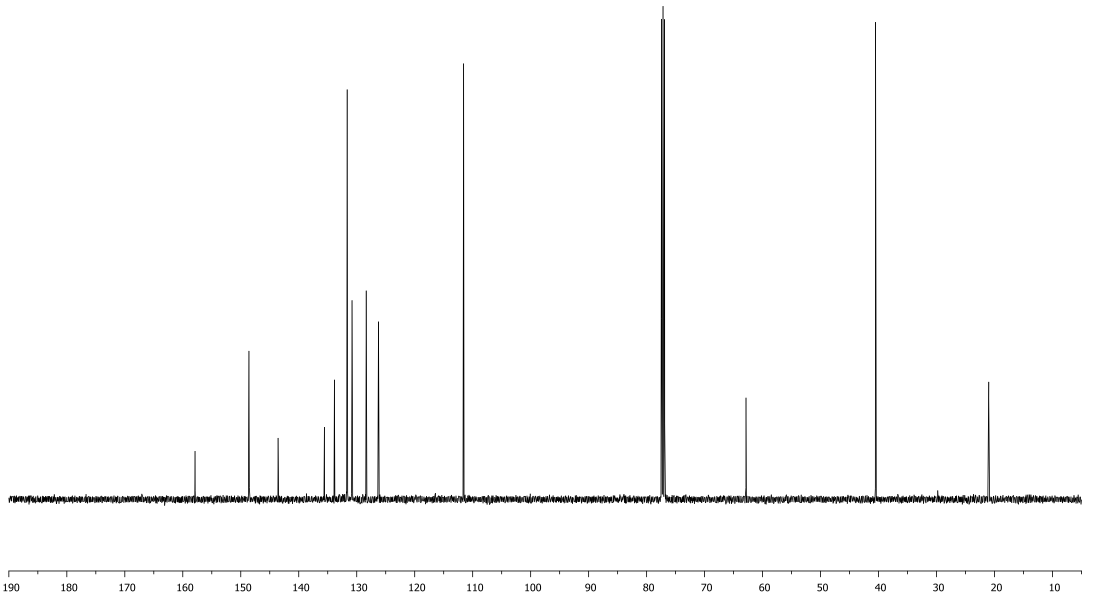

Supplementary Figure 25. ^{1}**H NMR Spectrum of 4k (500 MHz, CDCl$_3$)**

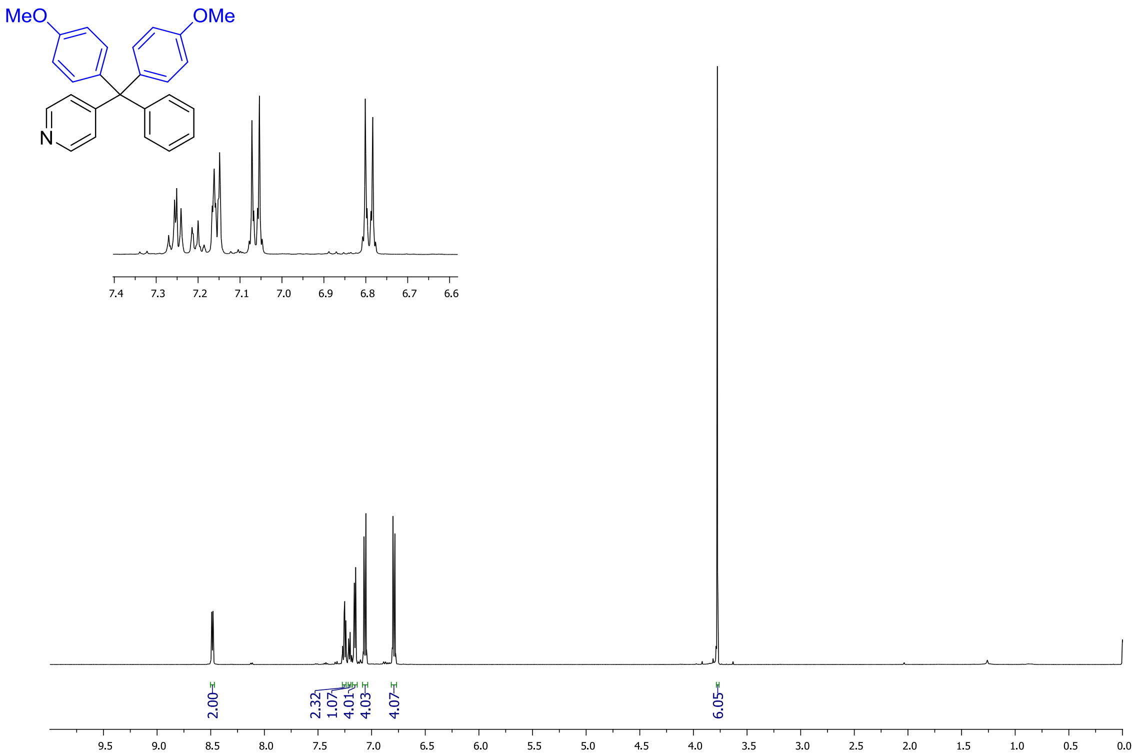

Supplementary Figure 26. ^{13}**C NMR Spectrum of 4k (125 MHz, CDCl$_3$)**

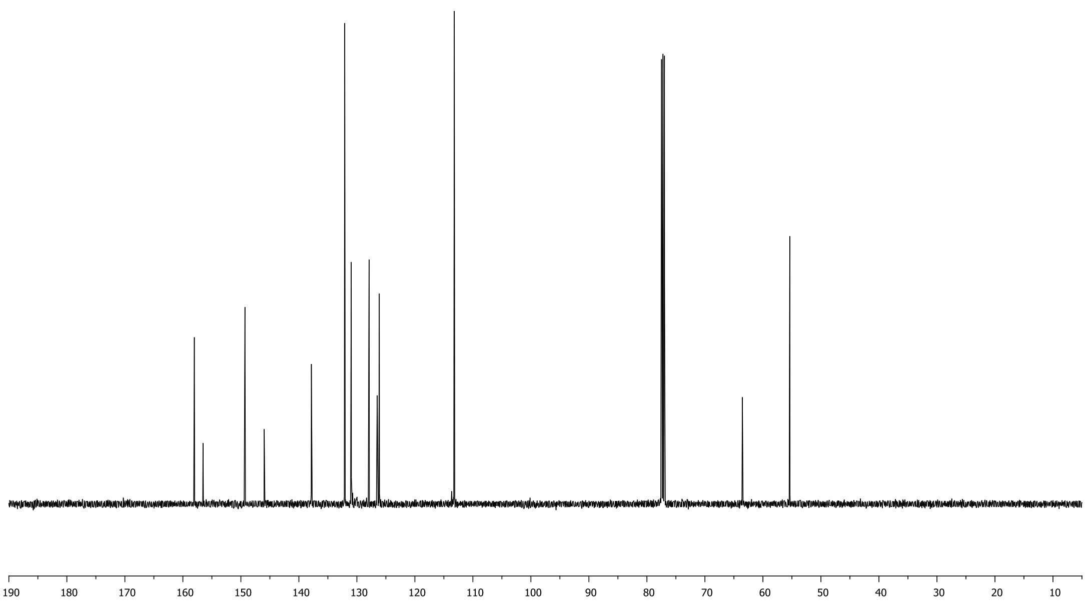

Supplementary Figure 27. ^{1}H NMR Spectrum of 4l (500 MHz, $CDCl_3$)

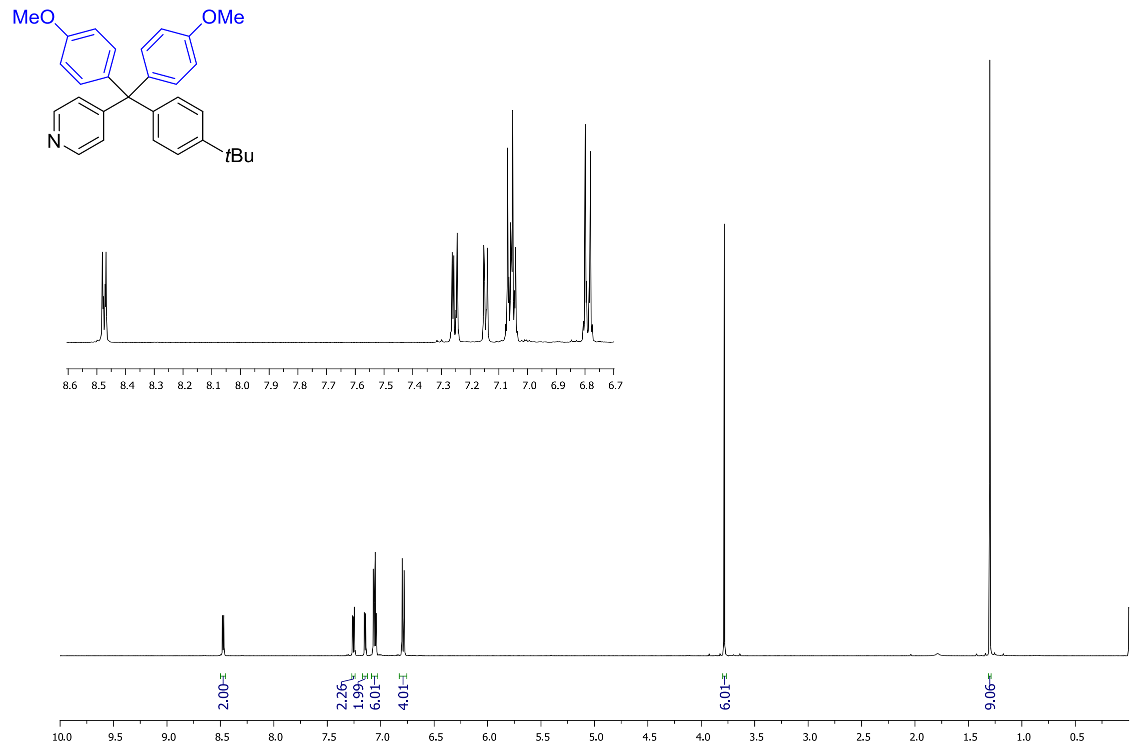

Supplementary Figure 28. ^{13}C NMR Spectrum of 4l (125 MHz, $CDCl_3$)

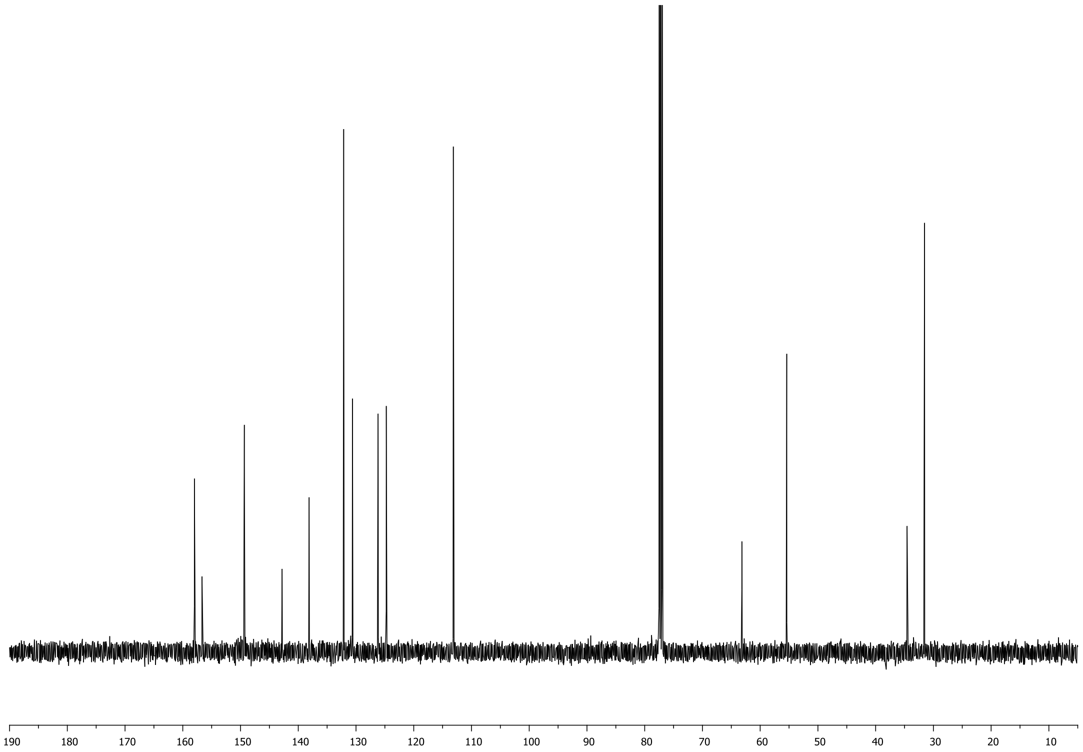

Supplementary Figure 29. ^{1}H NMR Spectrum of 4m (500 MHz, $CDCl_3$)

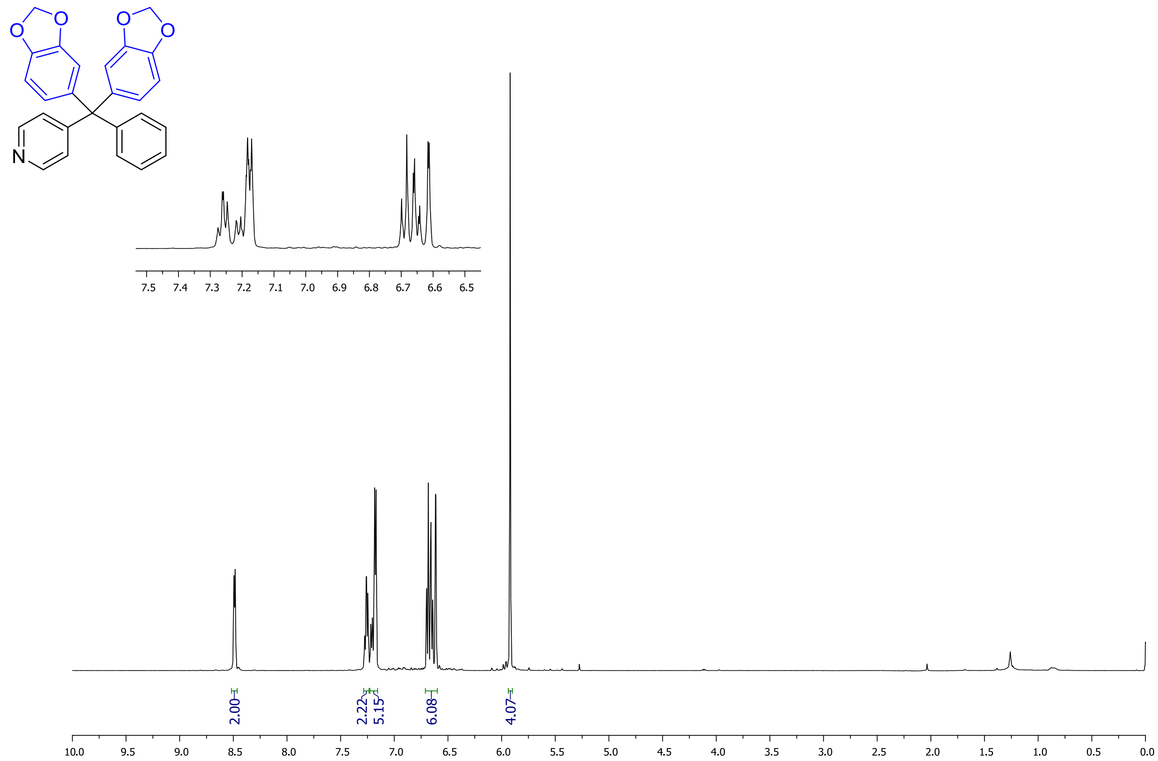

Supplementary Figure 30. ^{13}C NMR Spectrum of 4m (125 MHz, $CDCl_3$)

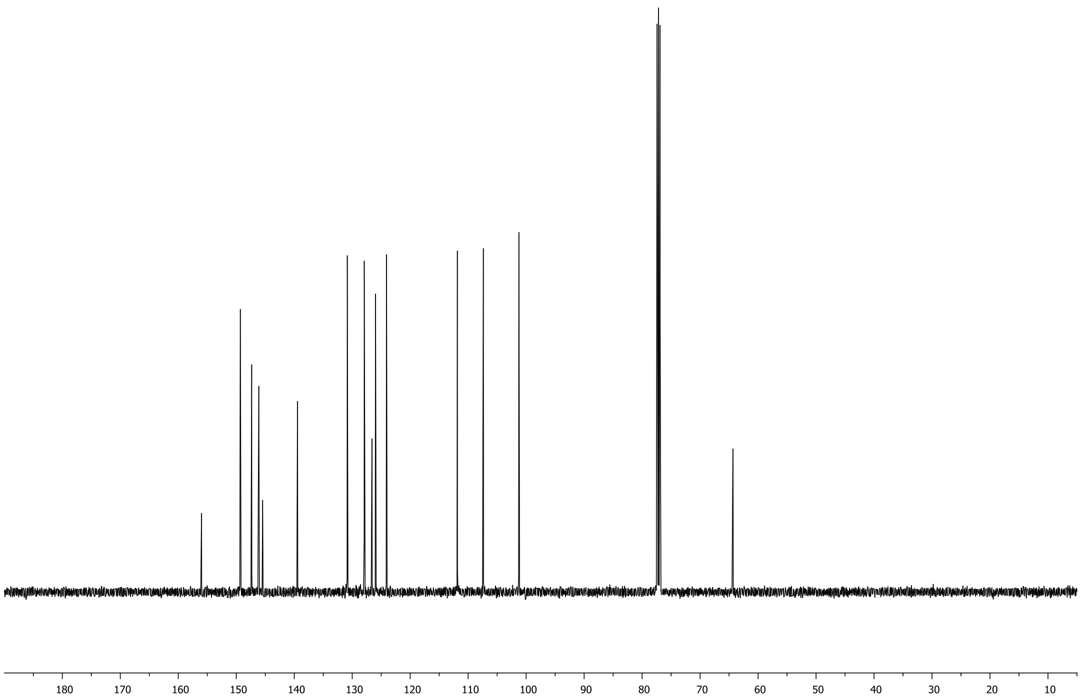

Supplementary Figure 31. ^{1}H NMR Spectrum of 4n (500 MHz, $CDCl_3$)

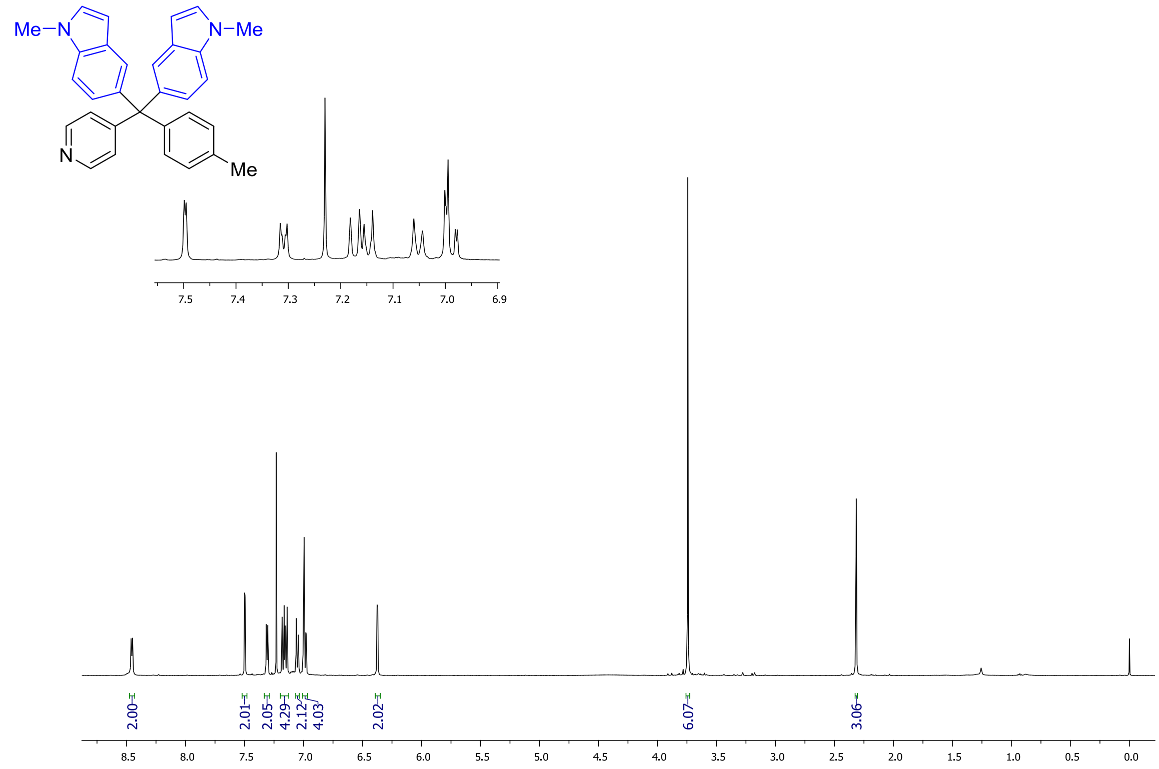

Supplementary Figure 32. ^{13}C NMR Spectrum of 4n (125 MHz, $CDCl_3$)

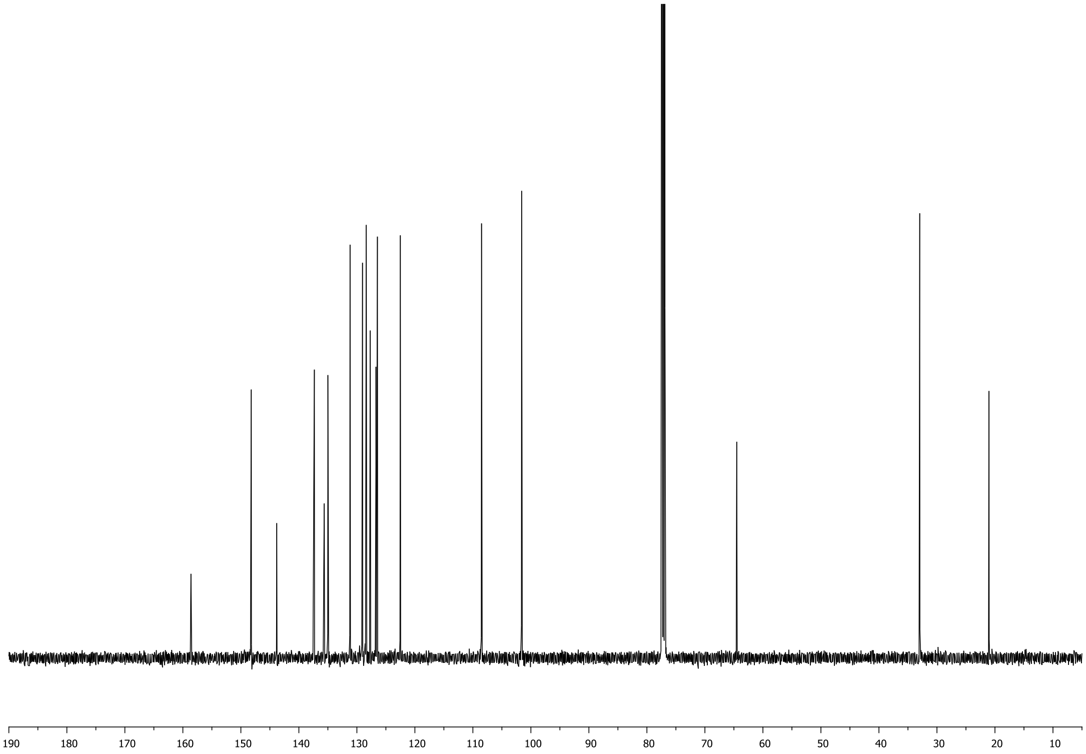

Supplementary Figure 33. ^{1}H NMR Spectrum of 4o (500 MHz, $CDCl_3$)

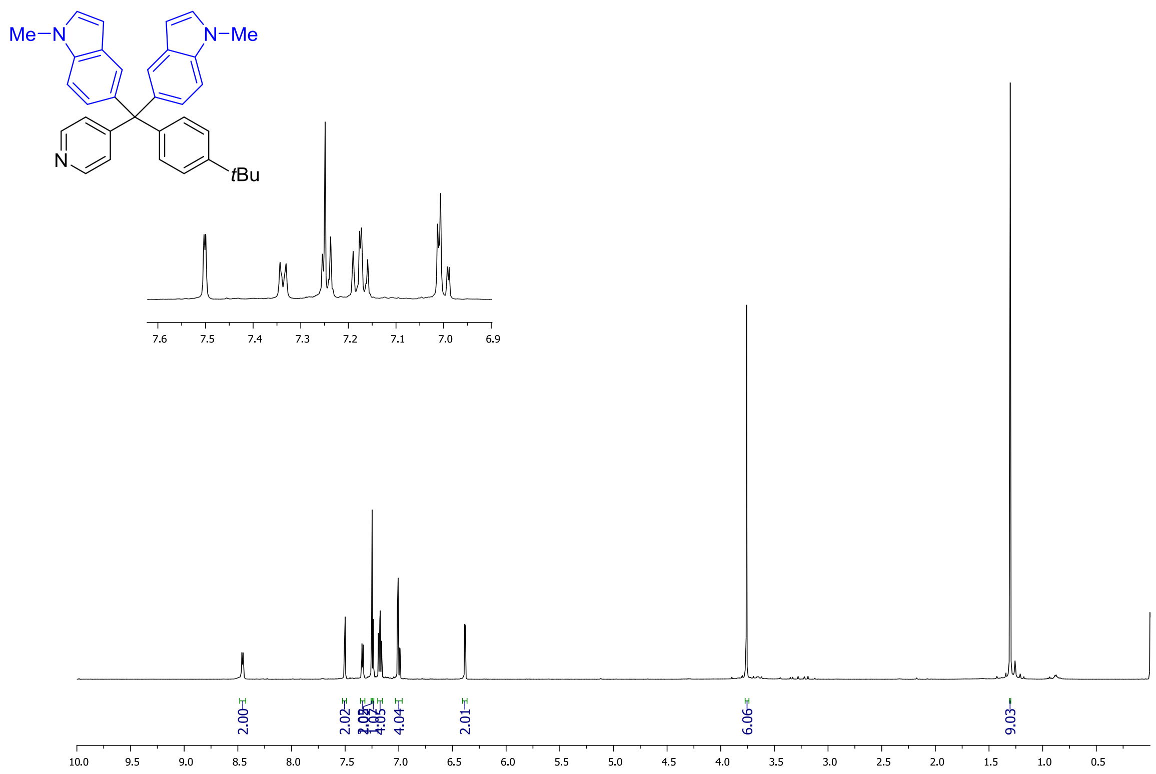

Supplementary Figure 34. ^{13}C NMR Spectrum of 4o (125 MHz, $CDCl_3$)

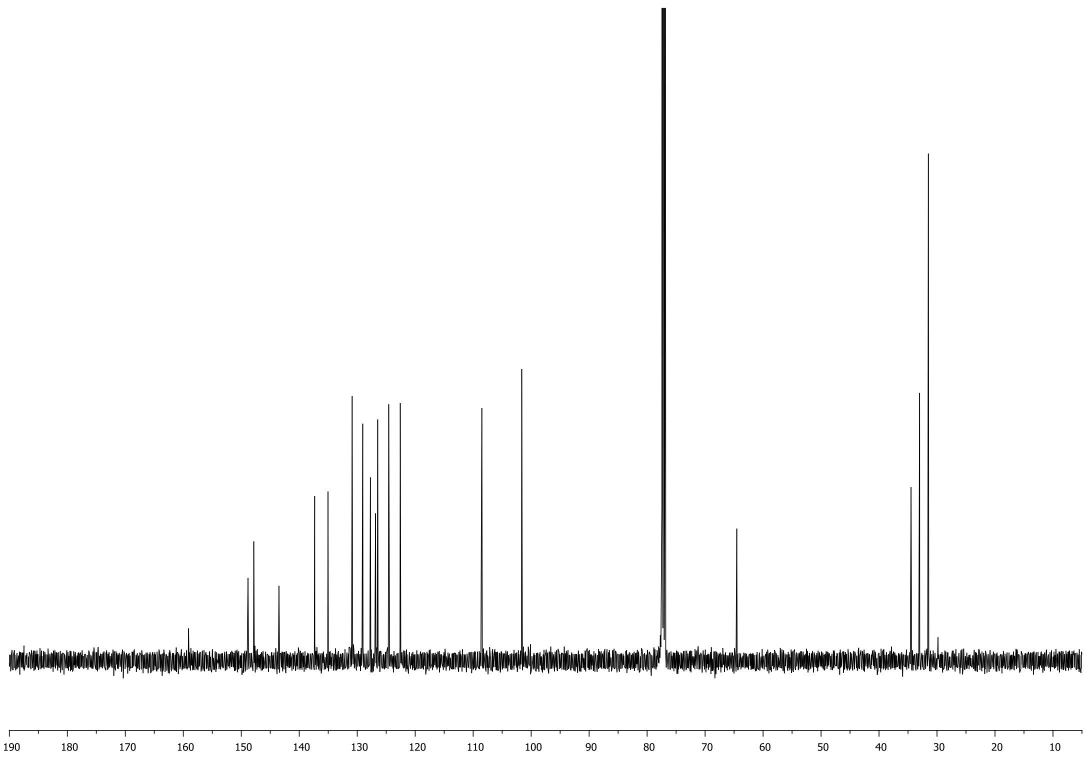

Supplementary Figure 35. ^{1}H NMR Spectrum of 4p (500 MHz, $CDCl_3$)

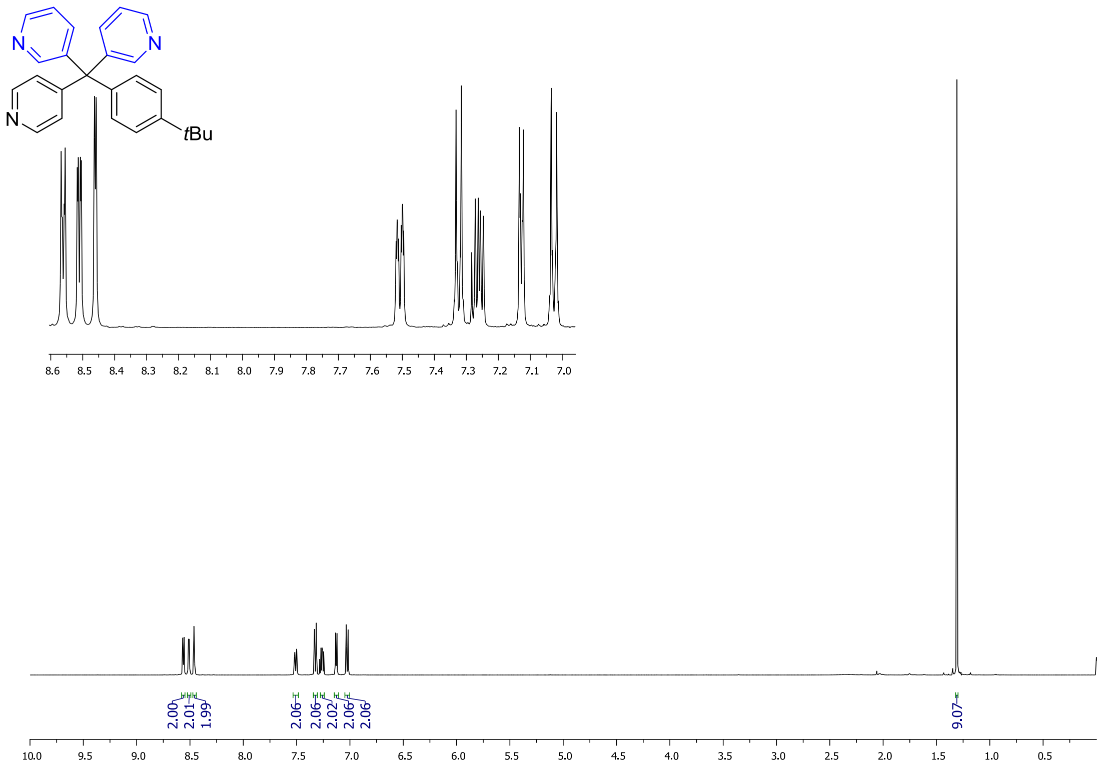

Supplementary Figure 36. ^{13}C NMR Spectrum of 4p (125 MHz, $CDCl_3$)

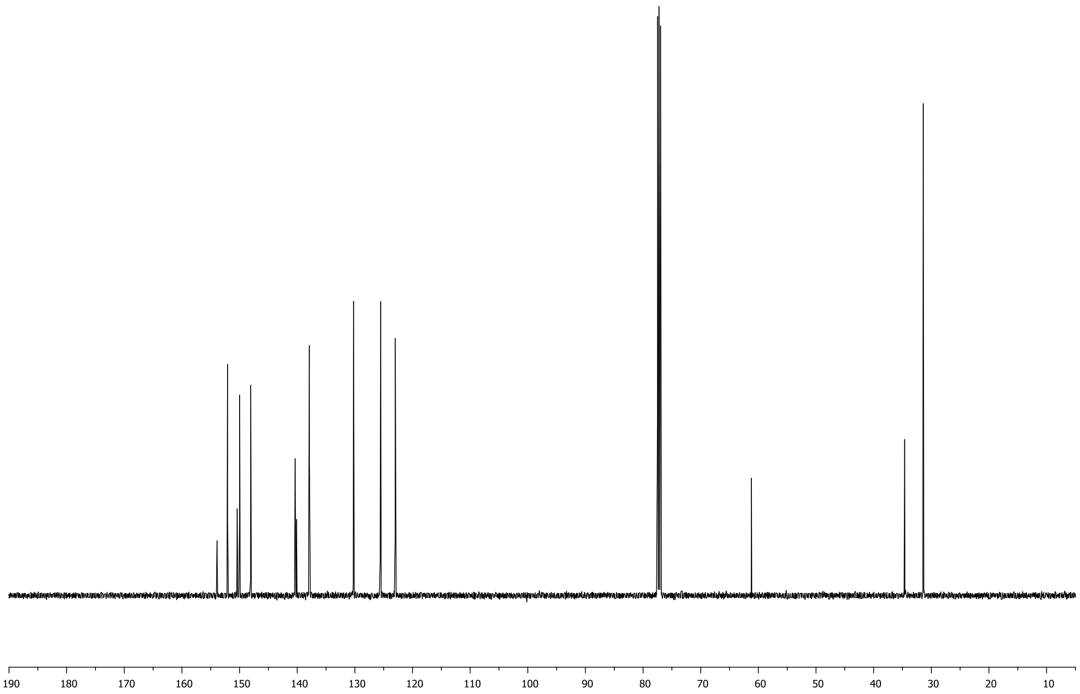

Supplementary Figure 37. ^{1}**H NMR Spectrum of 4q (500 MHz,** $CDCl_3$**)**

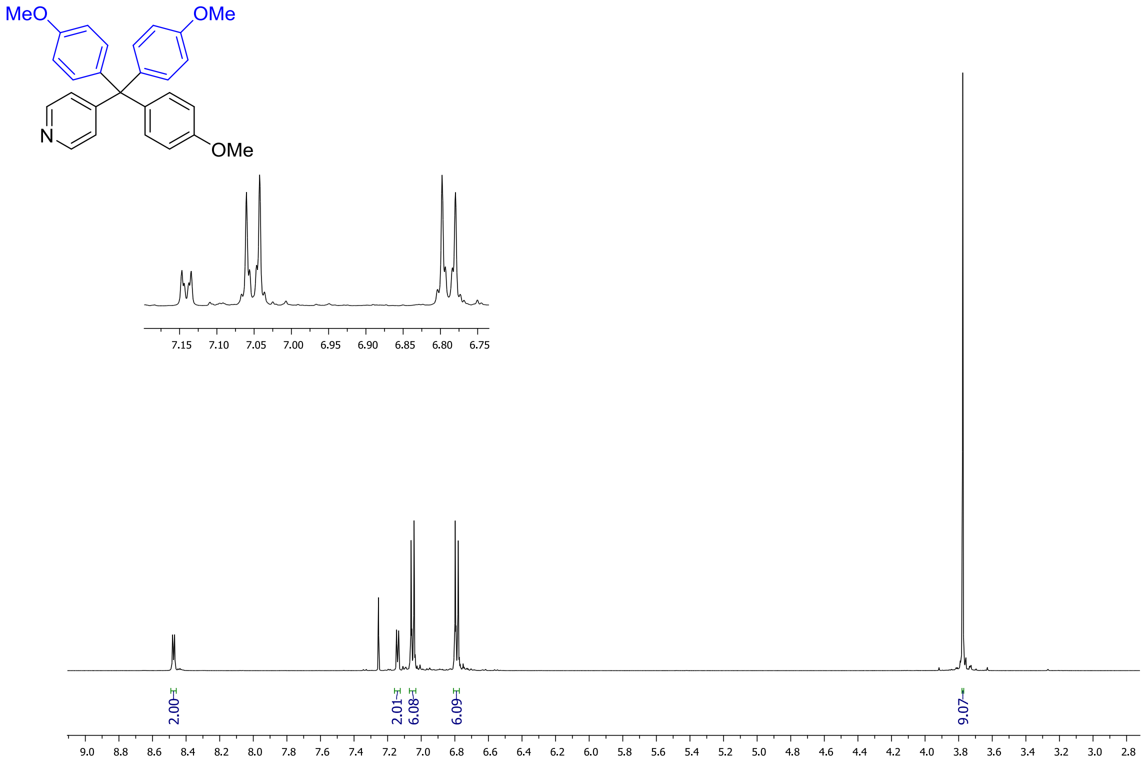

Supplementary Figure 38. ^{13}**C NMR Spectrum of 4q (125 MHz,** $CDCl_3$**)**

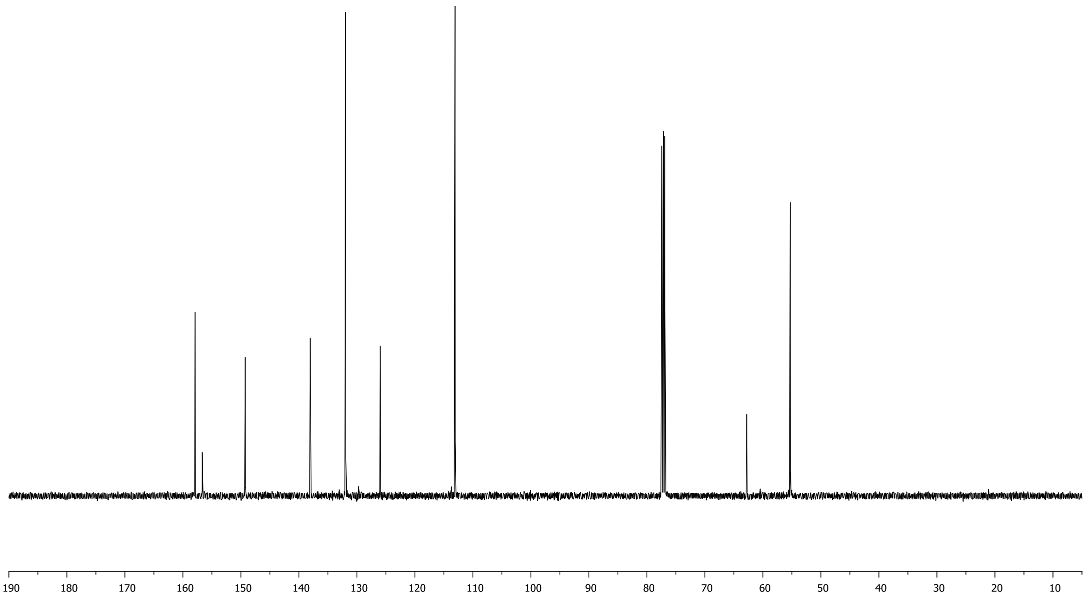

Supplementary Figure 39. ^{1}H NMR Spectrum of 4r (500 MHz, $CDCl_3$)

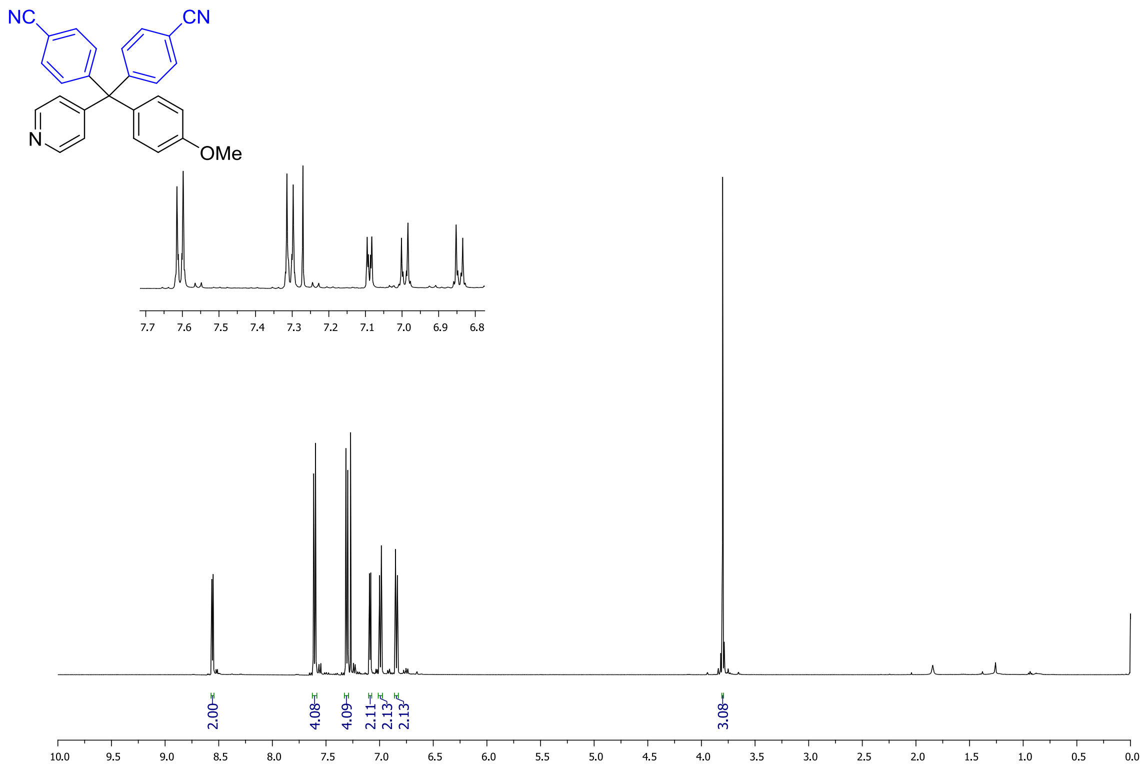

Supplementary Figure 40. ^{13}C NMR Spectrum of 4r (125 MHz, $CDCl_3$)

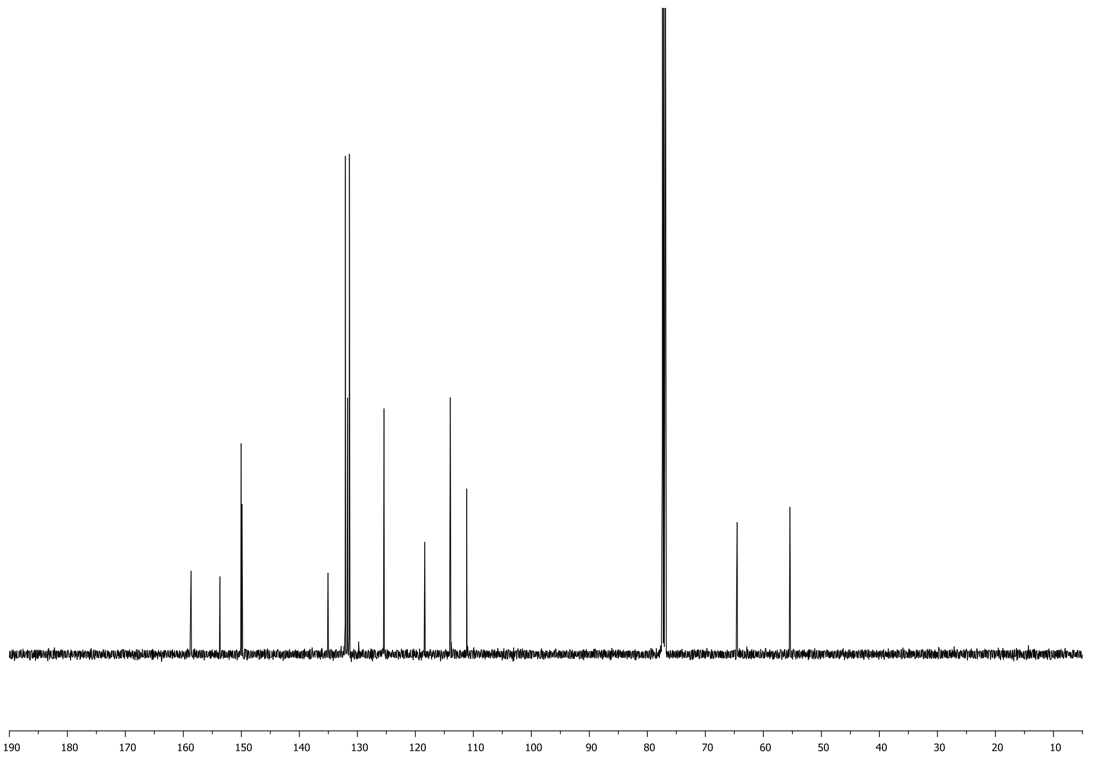

Supplementary Figure 41. ^{1}**H NMR Spectrum of 4s (500 MHz, CDCl$_3$)**

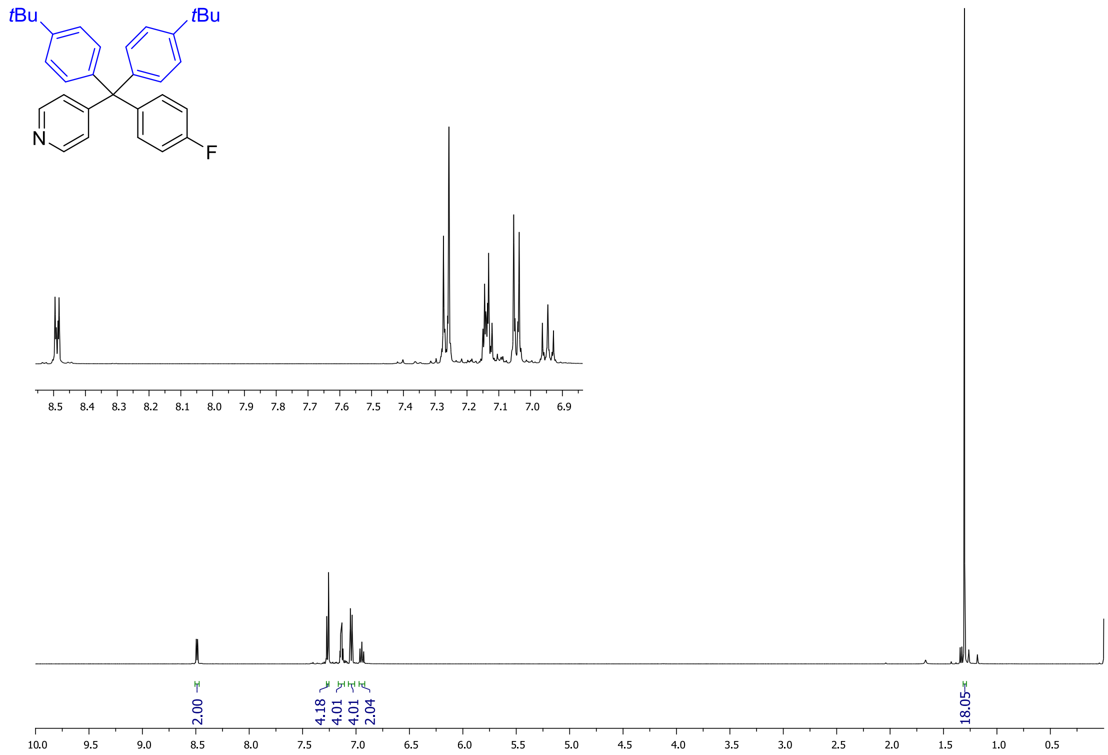

Supplementary Figure 42. ^{13}**C NMR Spectrum of 4s (125 MHz, CDCl$_3$)**

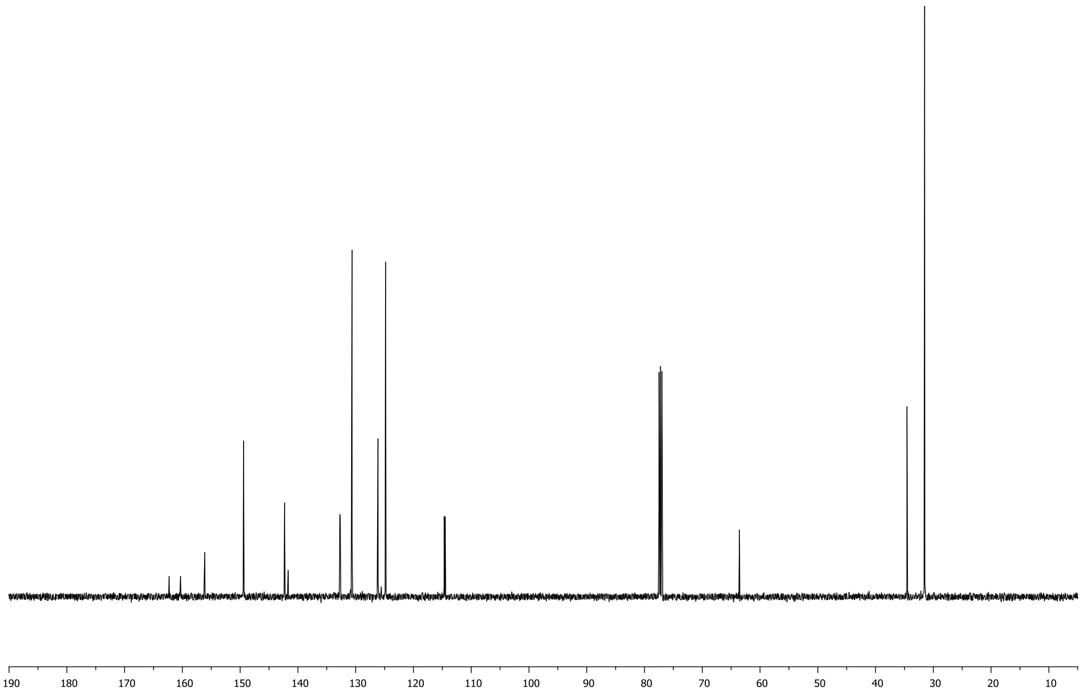

Supplementary Figure 43. ^{1}H NMR Spectrum of 4t (500 MHz, $CDCl_3$)

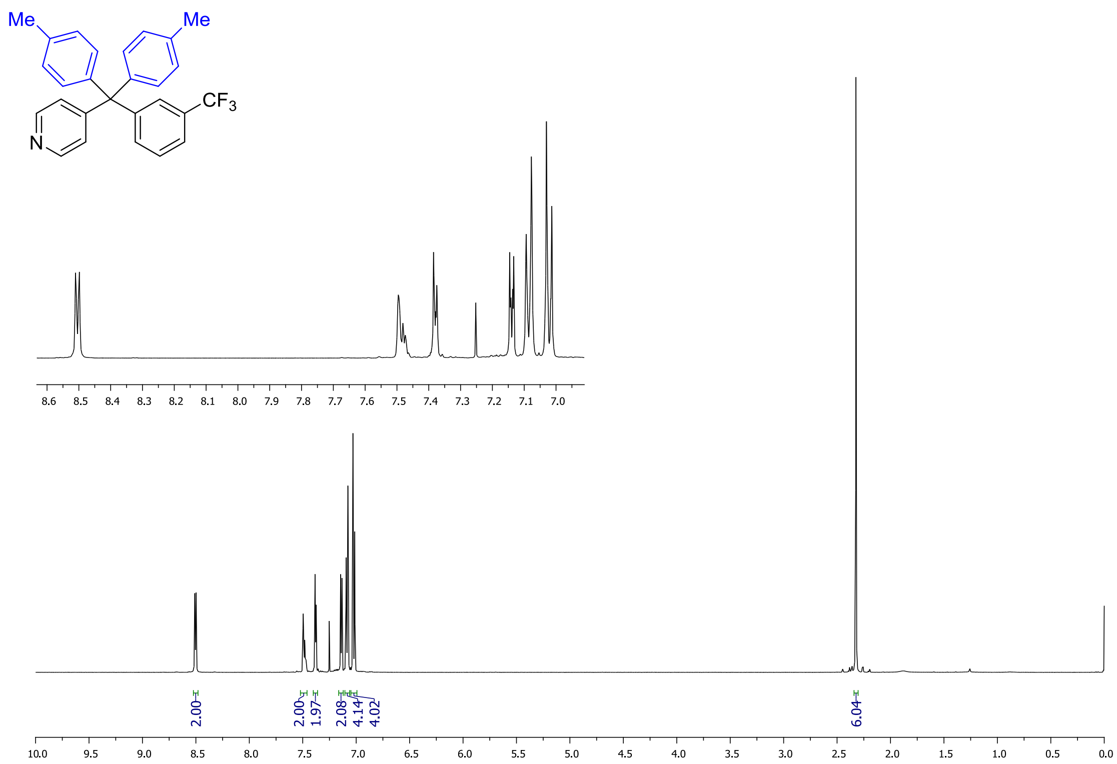

Supplementary Figure 44. ^{13}C NMR Spectrum of 4t (125 MHz, $CDCl_3$)

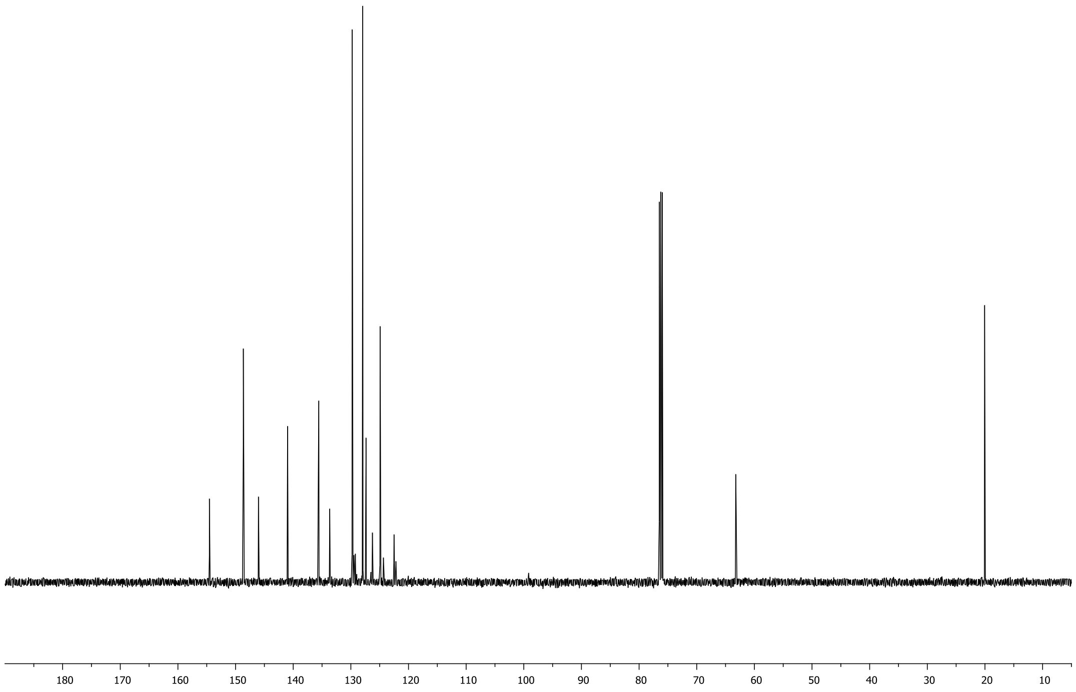

Supplementary Figure 45. ^{1}H NMR Spectrum of 6a (500 MHz, $CDCl_3$)

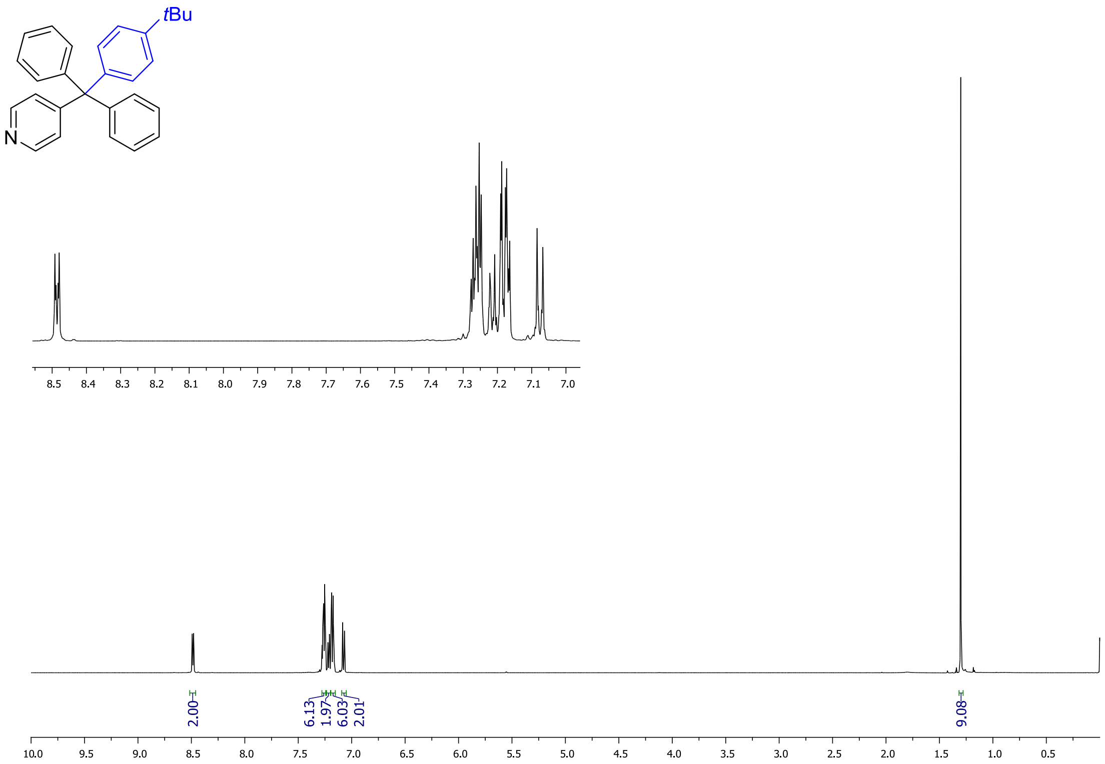

Supplementary Figure 46. ^{13}C NMR Spectrum of 6a (125 MHz, $CDCl_3$)

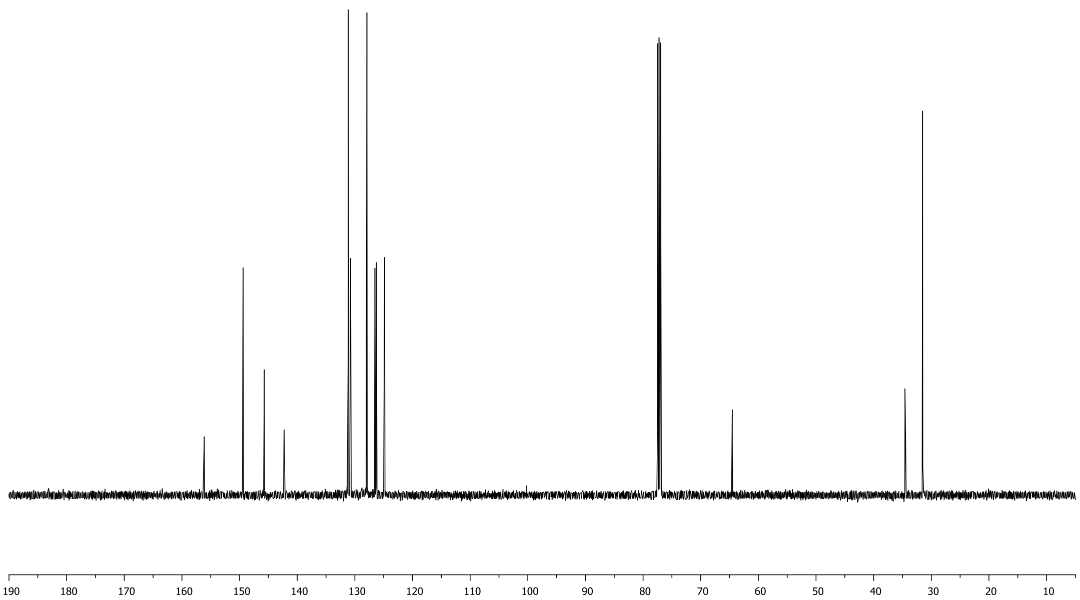

Supplementary Figure 47. ^{1}**H NMR Spectrum of 6b (500 MHz,** $CDCl_3$**)**

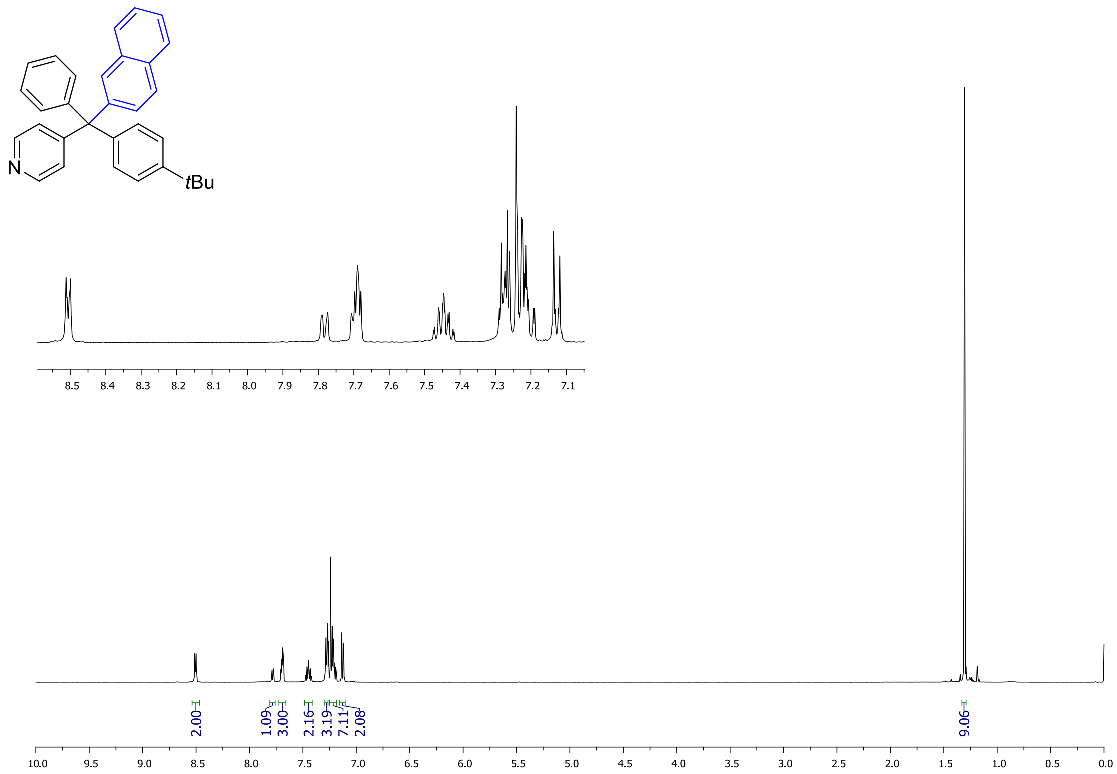

Supplementary Figure 48. ^{13}**C NMR Spectrum of 6b (125 MHz,** $CDCl_3$**)**

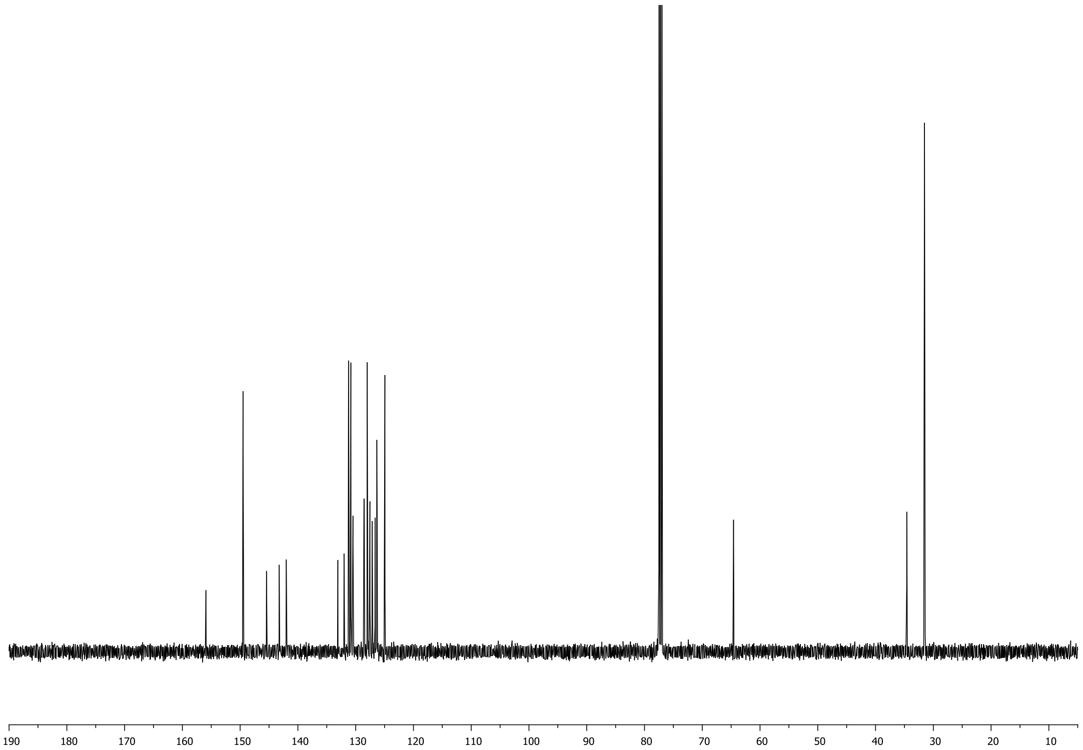

Supplementary Figure 49. ^{1}H NMR Spectrum of 6c (500 MHz, $CDCl_3$)

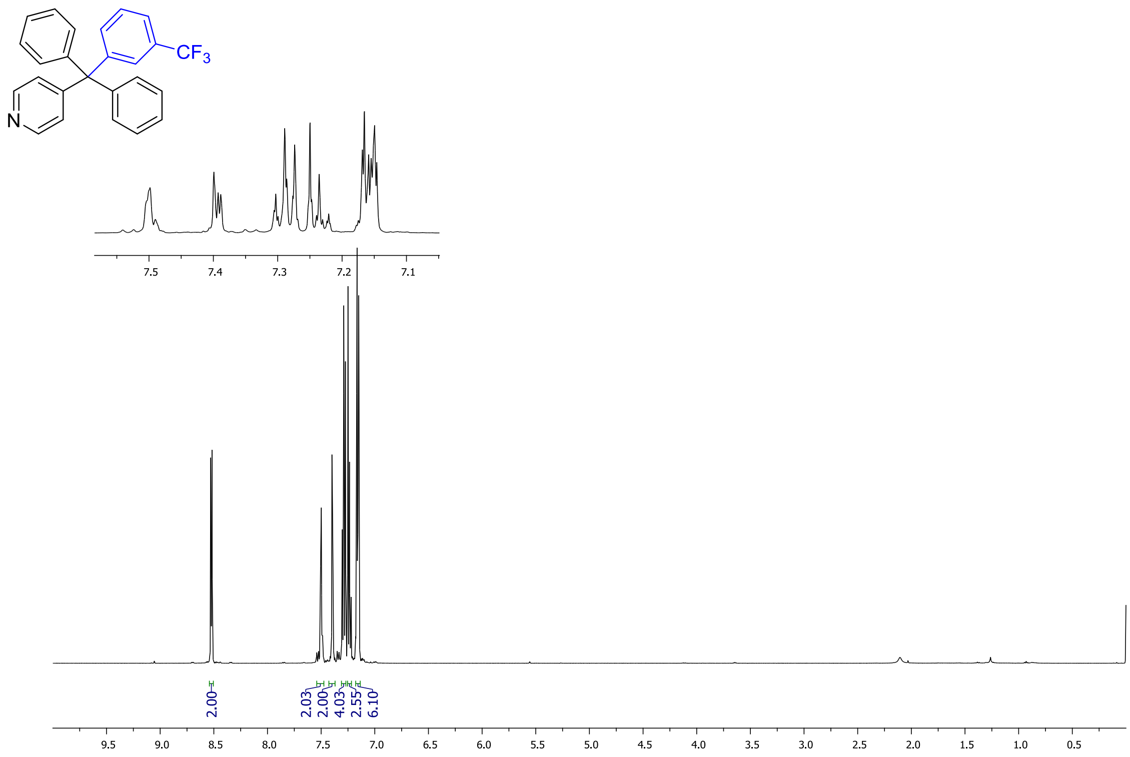

Supplementary Figure 50. ^{13}C NMR Spectrum of 6c (125 MHz, $CDCl_3$)

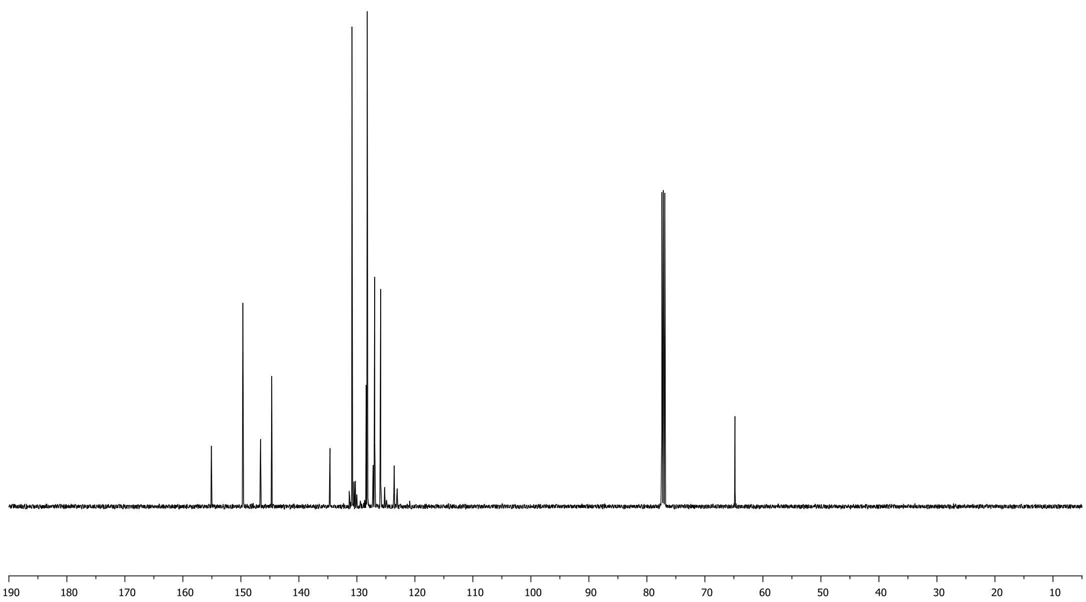

Supplementary Figure 51. ^{1}H NMR Spectrum of 6d (500 MHz, $CDCl_3$)

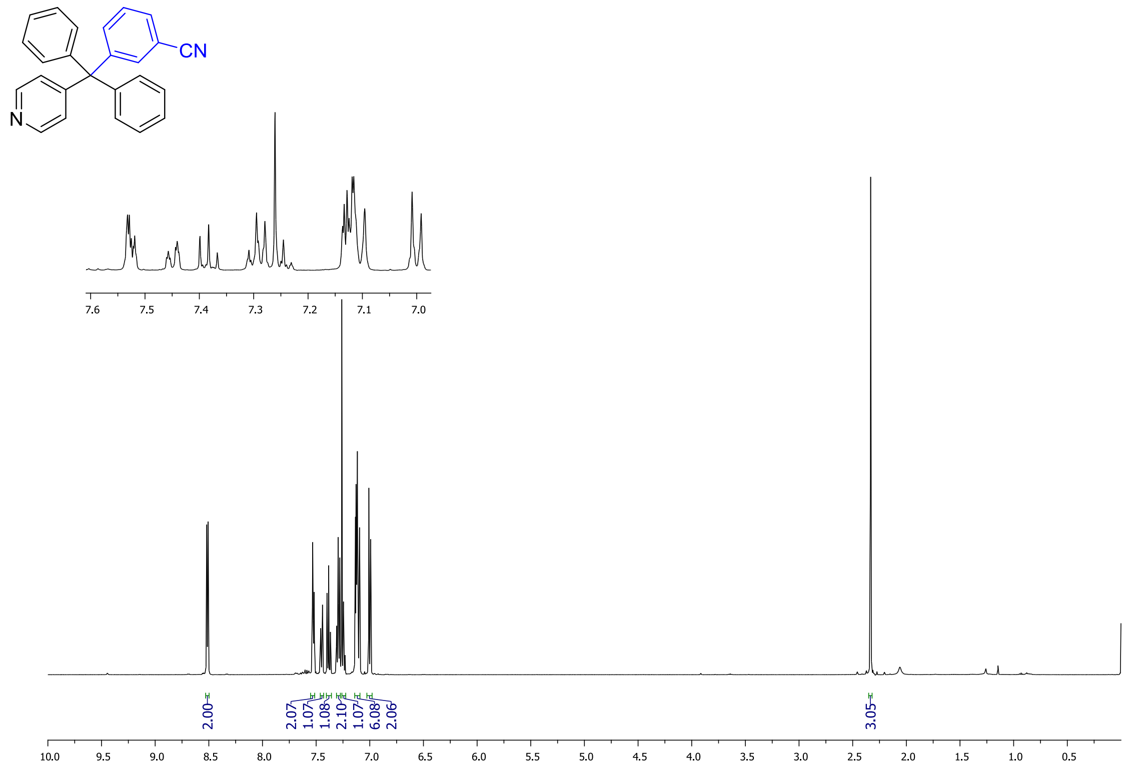

Supplementary Figure 52. ^{13}C NMR Spectrum of 6d (125 MHz, $CDCl_3$)

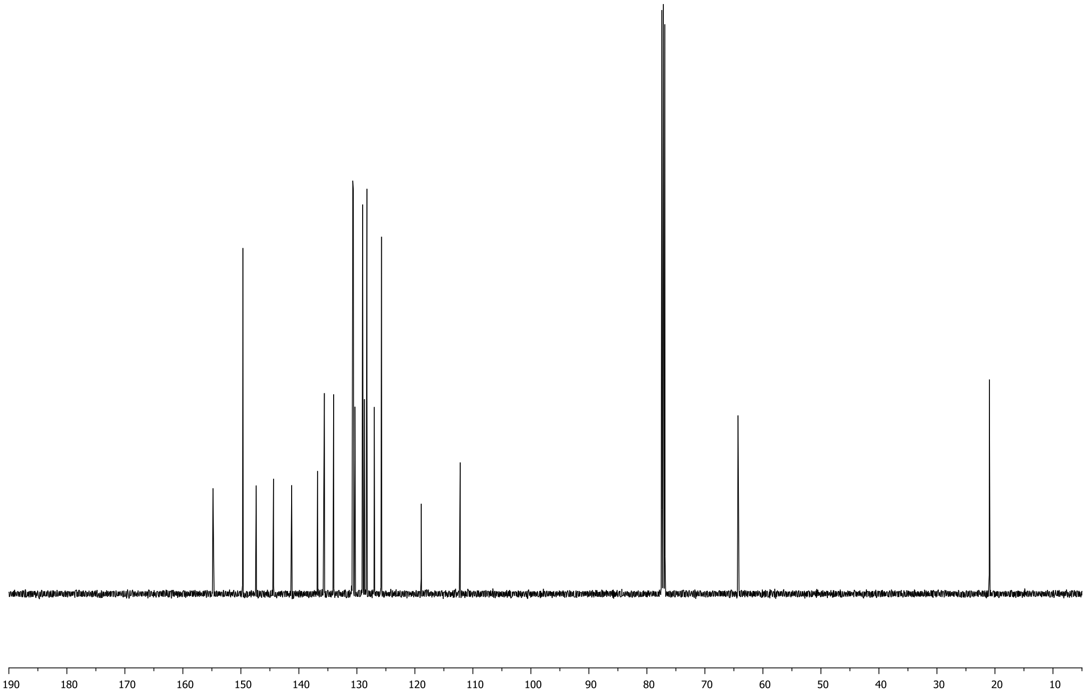

Supplementary Figure 53. ^{1}H NMR Spectrum of 6e (500 MHz, $CDCl_3$)

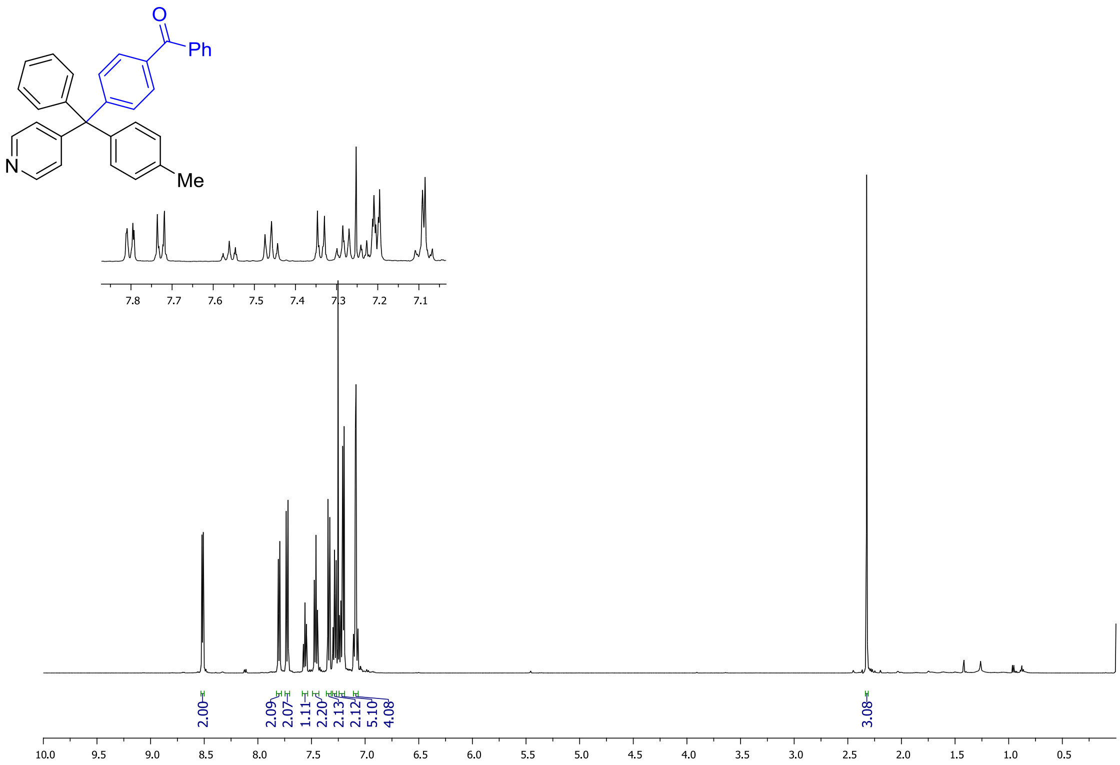

Supplementary Figure 54. ^{13}C NMR Spectrum of 6e (125 MHz, $CDCl_3$)

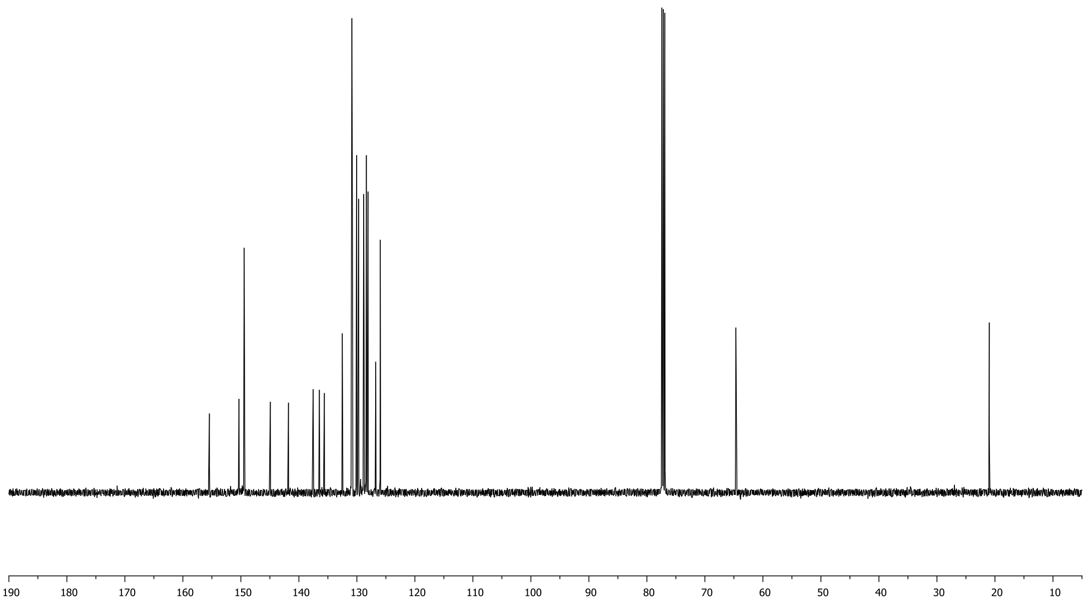

Supplementary Figure 55. ^{1}H NMR Spectrum of 6f (500 MHz, $CDCl_3$)

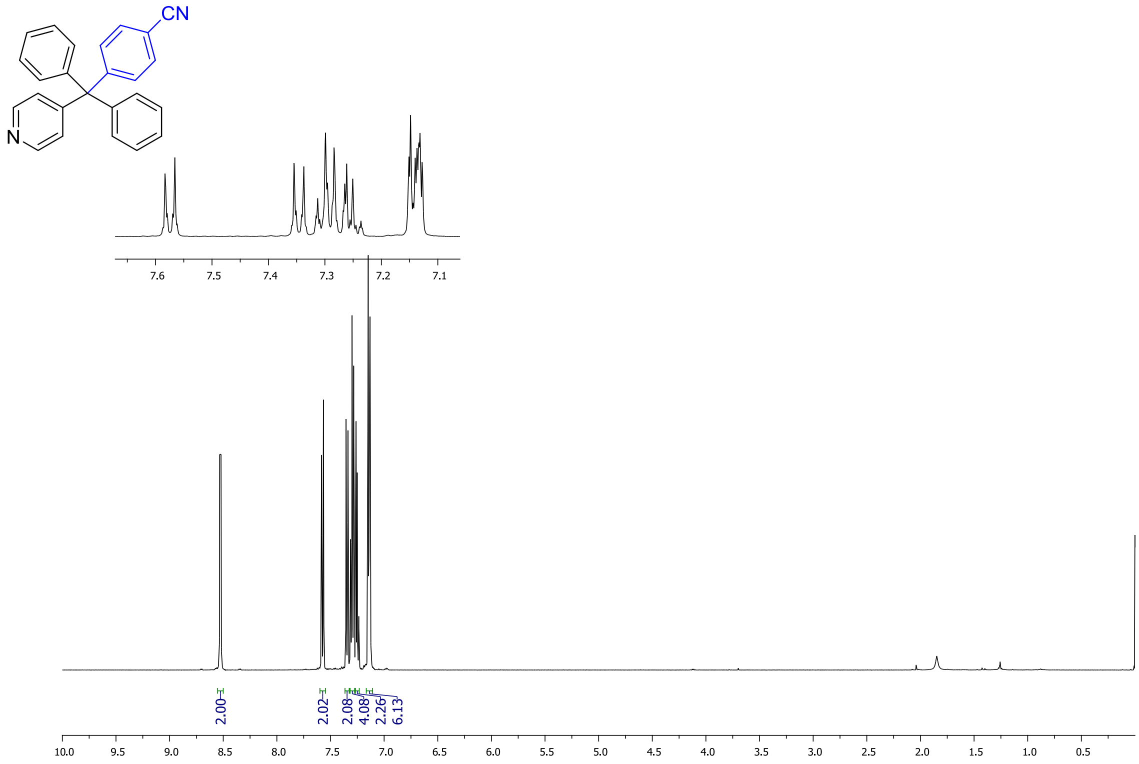

Supplementary Figure 56. ^{13}C NMR Spectrum of 6f (125 MHz, $CDCl_3$)

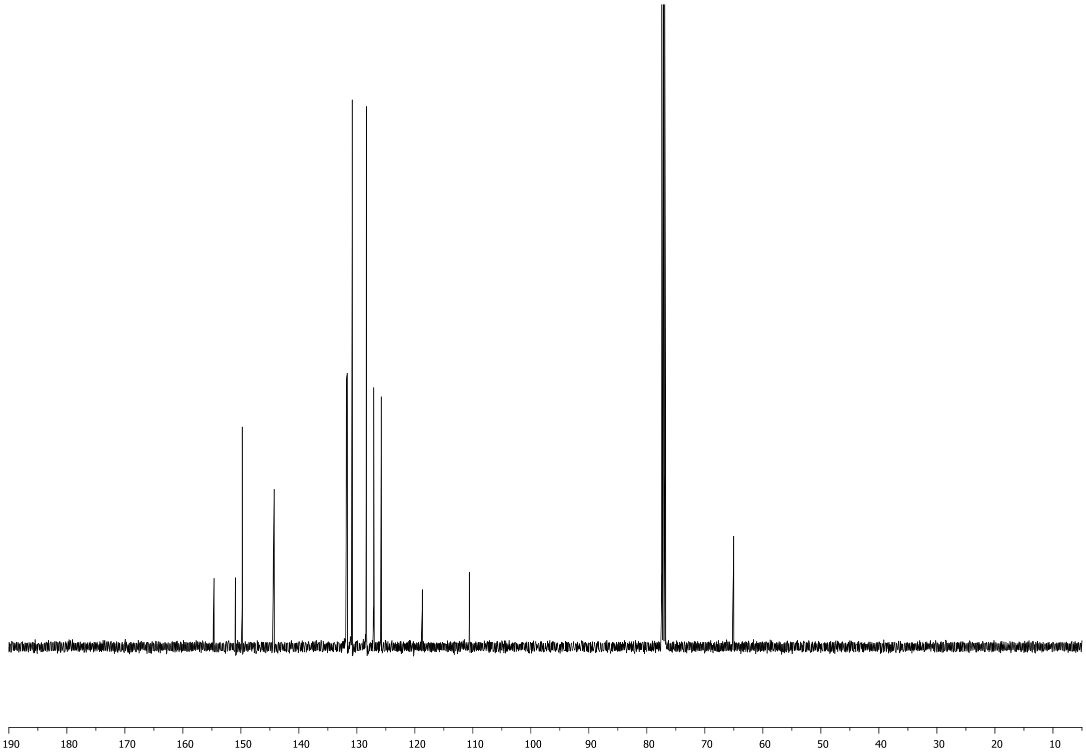

Supplementary Figure 57. ^{1}H NMR Spectrum of 6g (500 MHz, $CDCl_3$)

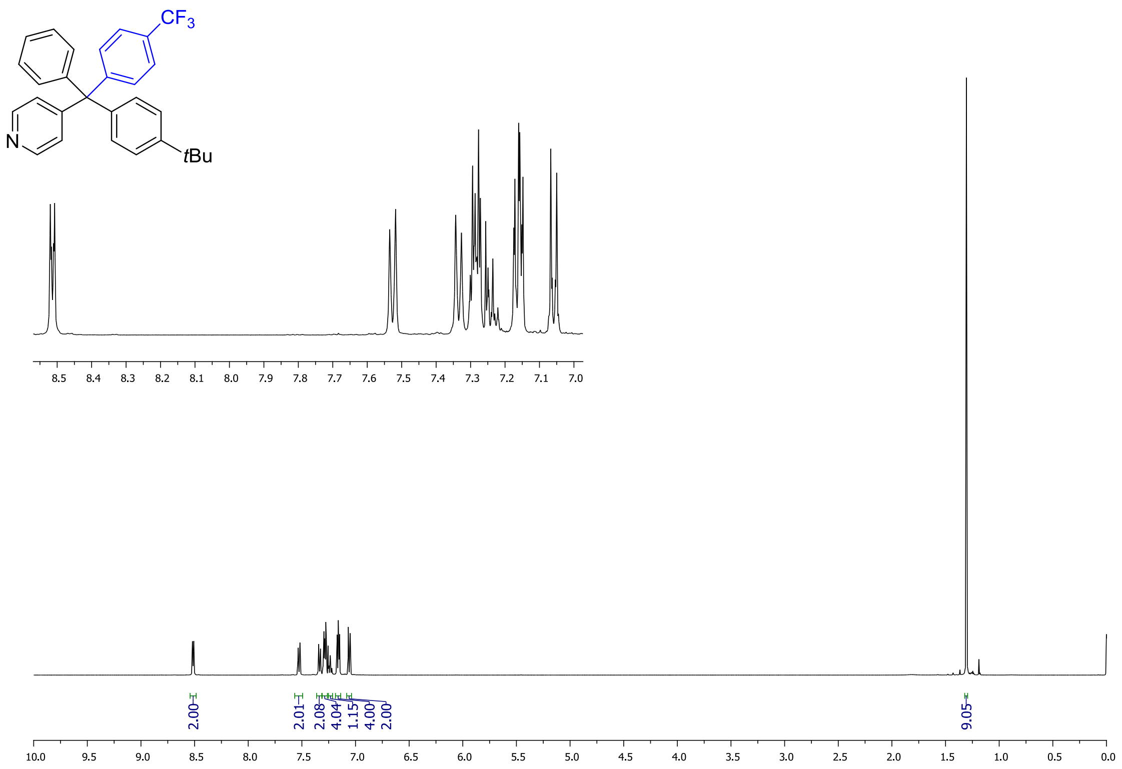

Supplementary Figure 58. ^{13}C NMR Spectrum of 6g (125 MHz, $CDCl_3$)

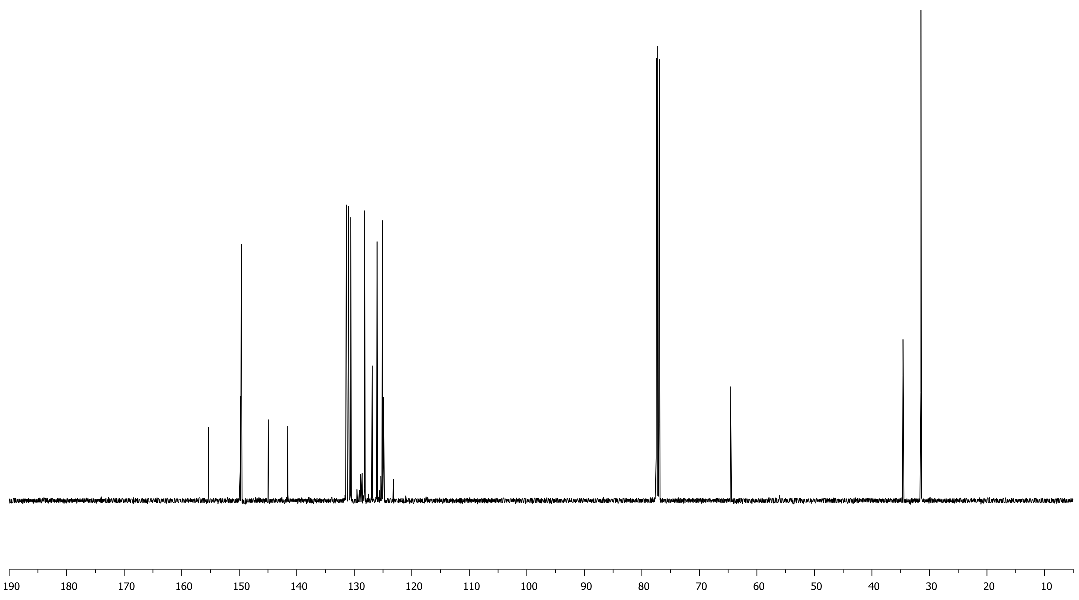

Supplementary Figure 59. ^{1}H NMR Spectrum of 6h (500 MHz, $CDCl_3$)

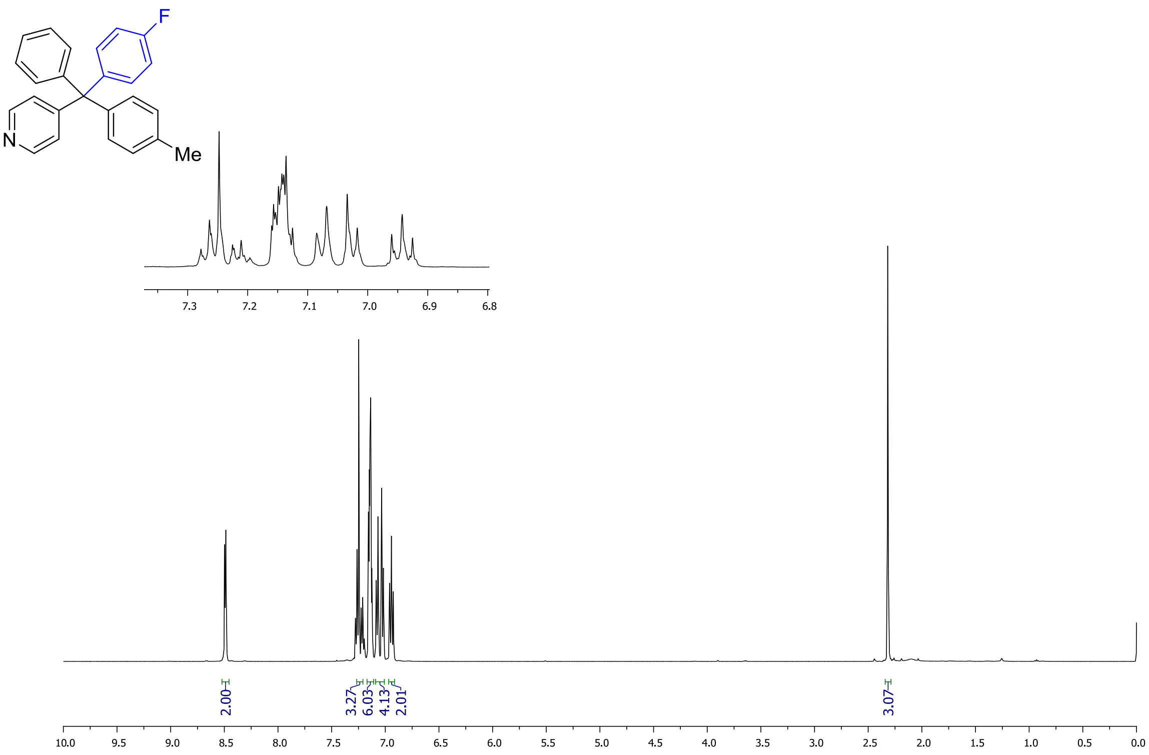

Supplementary Figure 60. ^{13}C NMR Spectrum of 6h (125 MHz, $CDCl_3$)

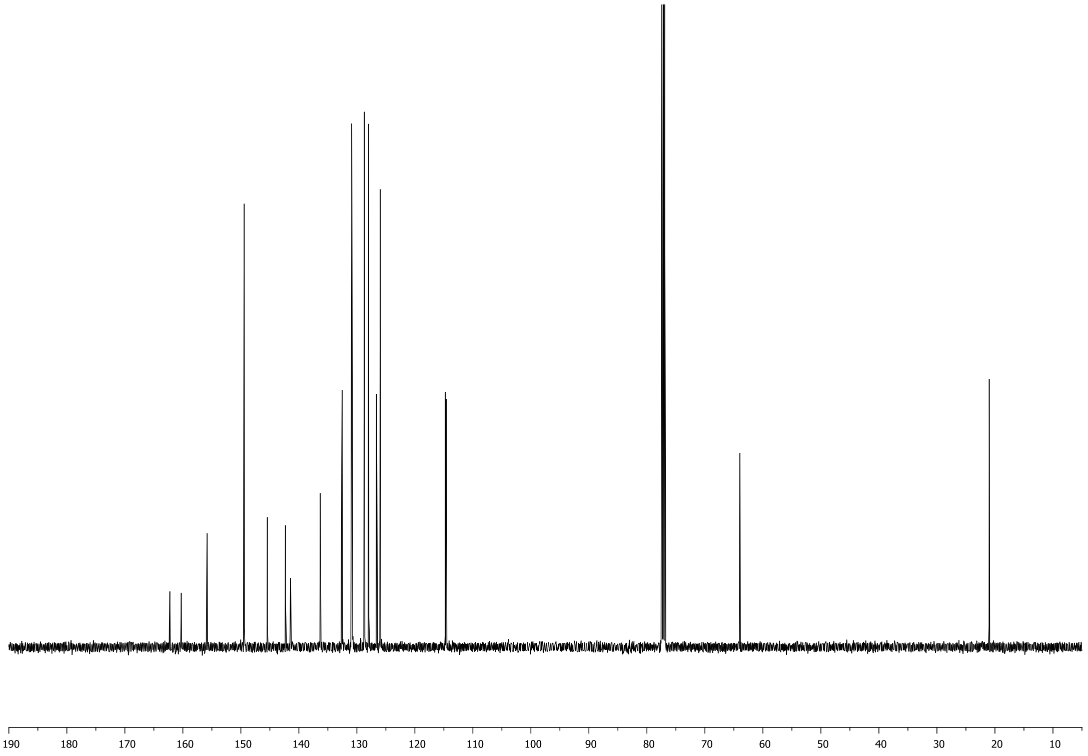

Supplementary Figure 61. ^{1}H NMR Spectrum of 6i (500 MHz, $CDCl_3$)

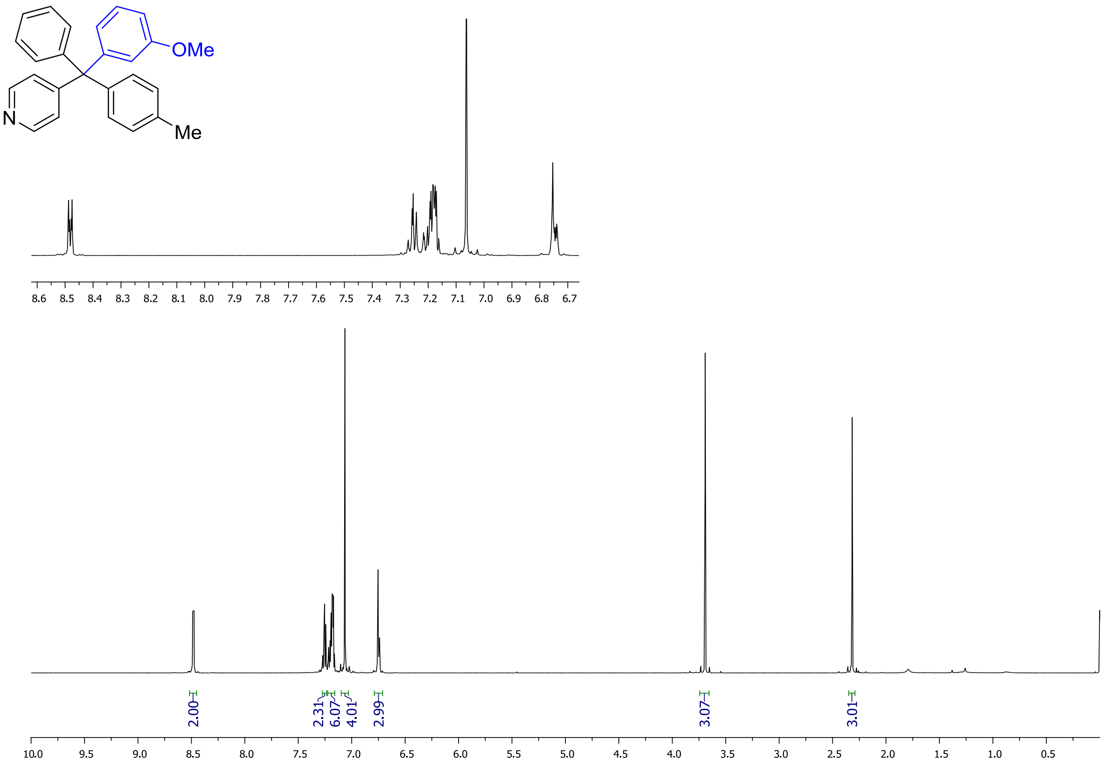

Supplementary Figure 62. ^{13}C NMR Spectrum of 6i (125 MHz, $CDCl_3$)

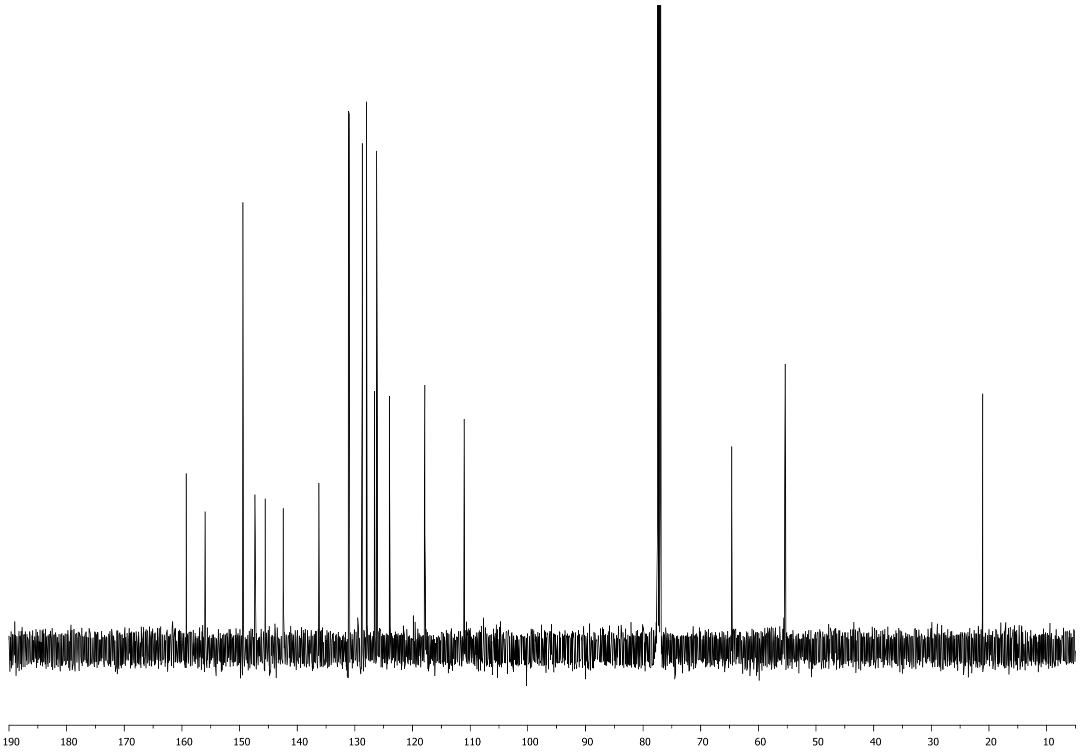

Supplementary Figure 63. ^{1}H NMR Spectrum of 6j (500 MHz, $CDCl_3$)

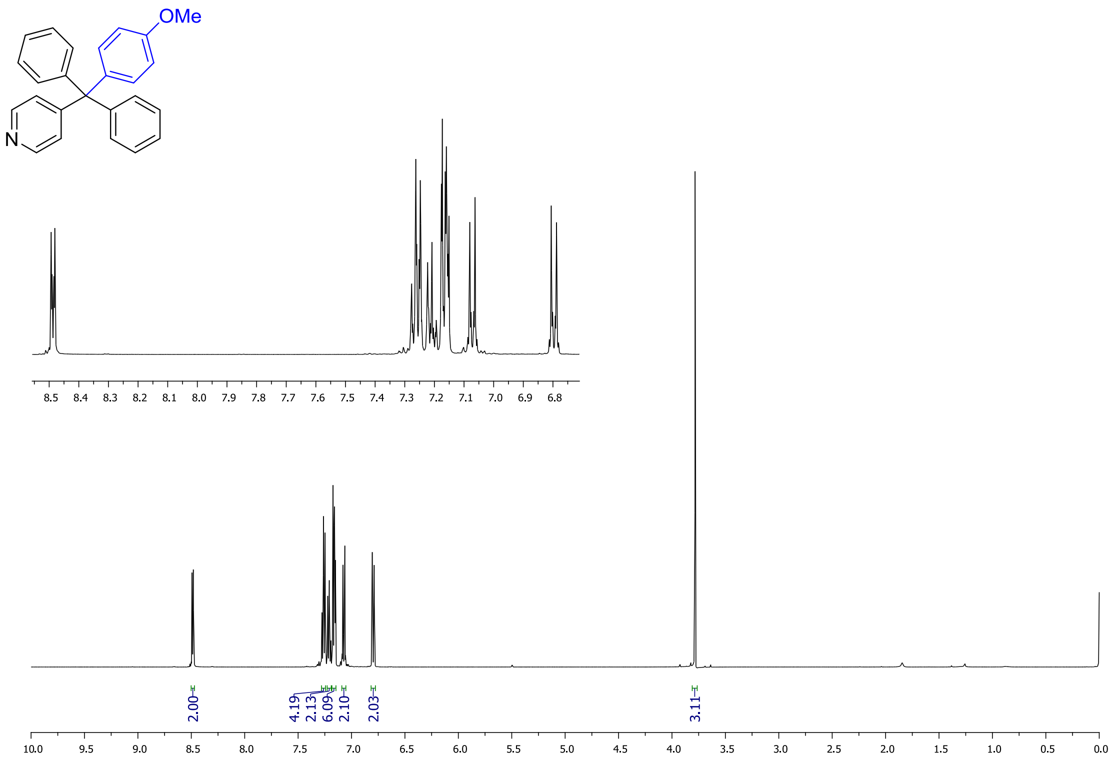

Supplementary Figure 64. ^{13}C NMR Spectrum of 6j (125 MHz, $CDCl_3$)

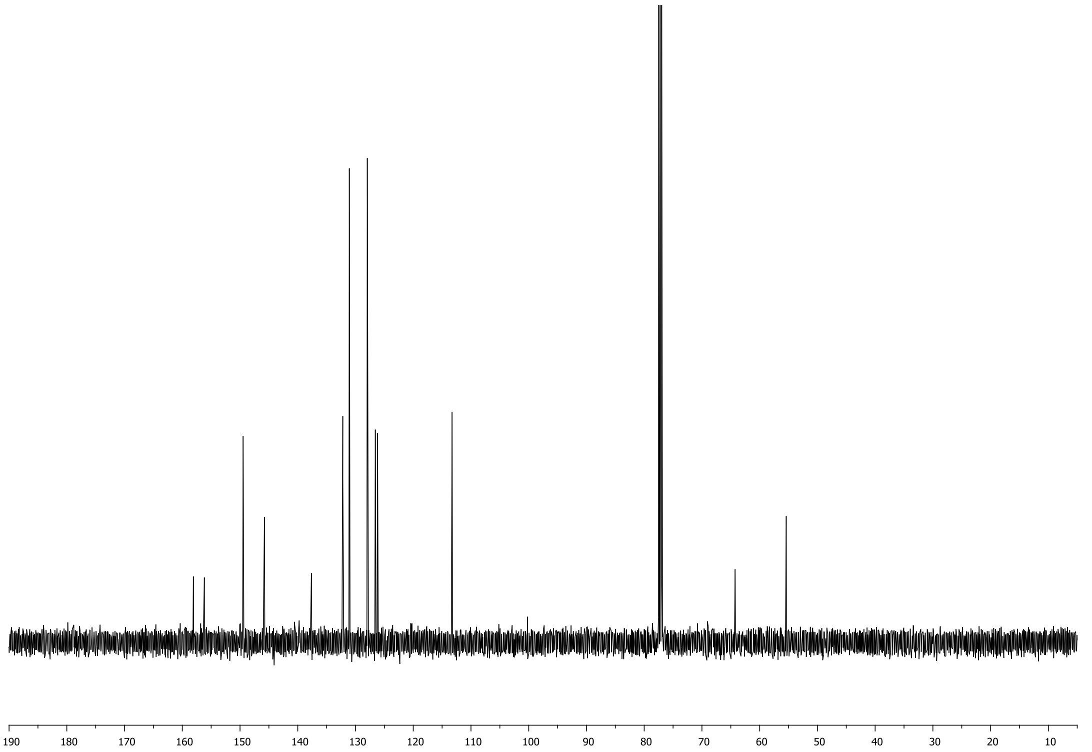

Supplementary Figure 65. ^{1}H NMR Spectrum of 6k (500 MHz, $CDCl_3$)

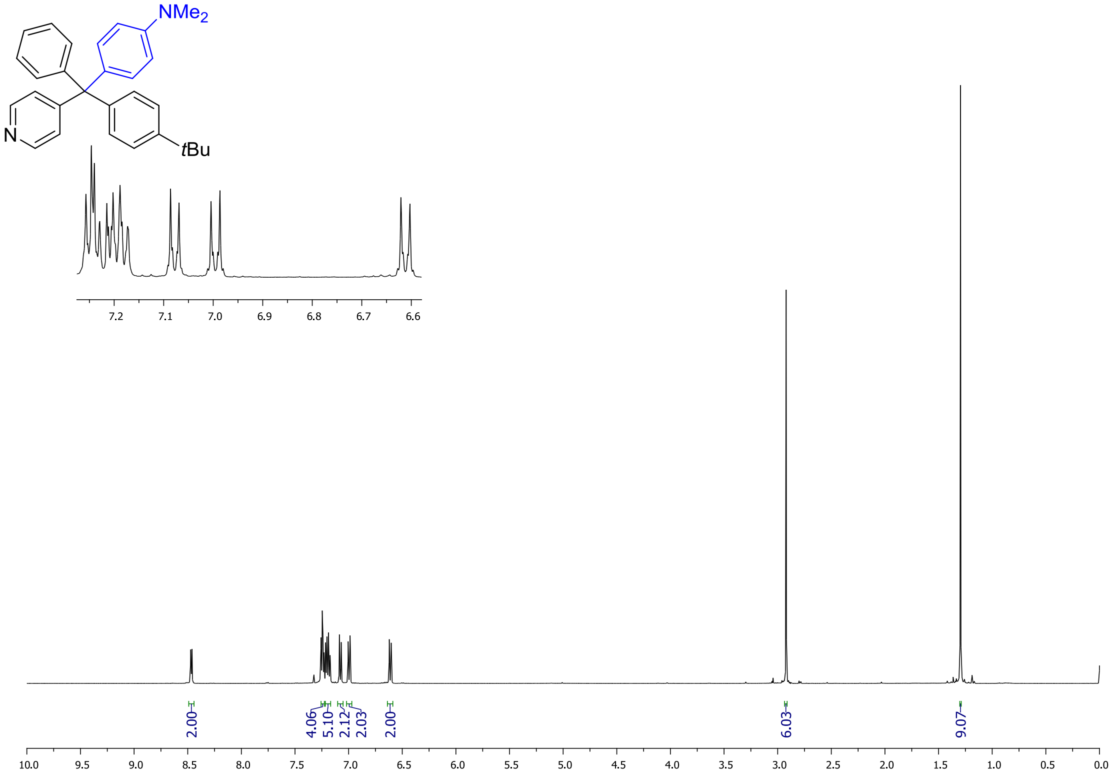

Supplementary Figure 66. ^{13}C NMR Spectrum of 6k (125 MHz, $CDCl_3$)

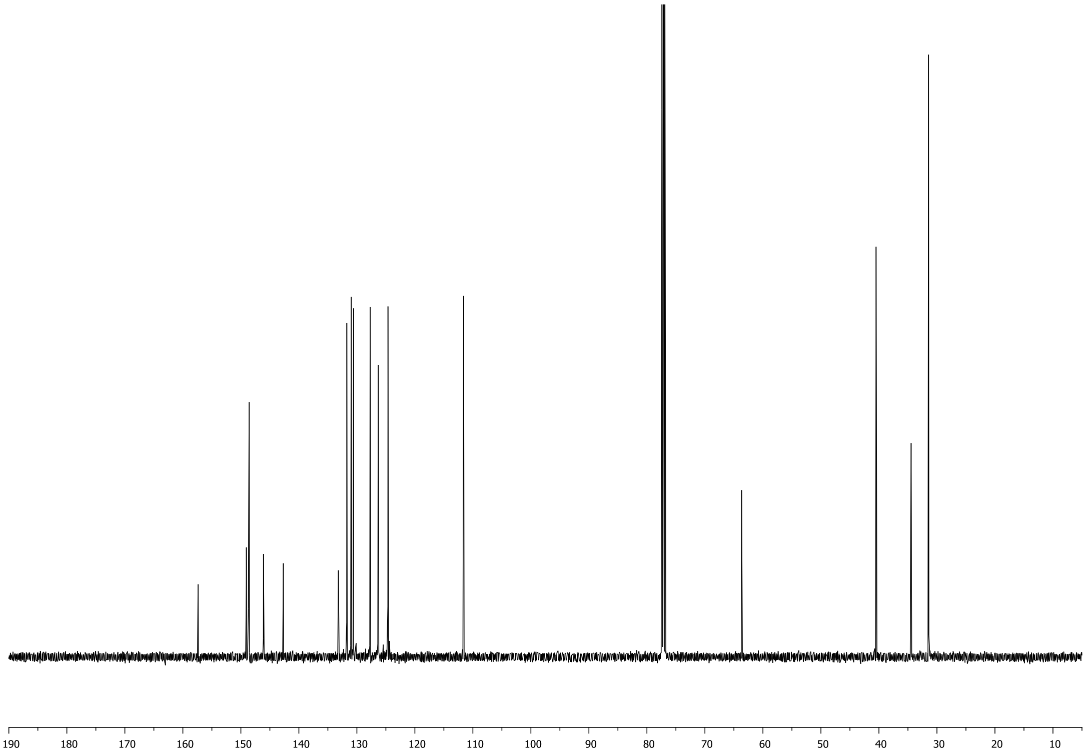

Supplementary Figure 67. ^{1}H NMR Spectrum of 6l (500 MHz, $CDCl_3$)

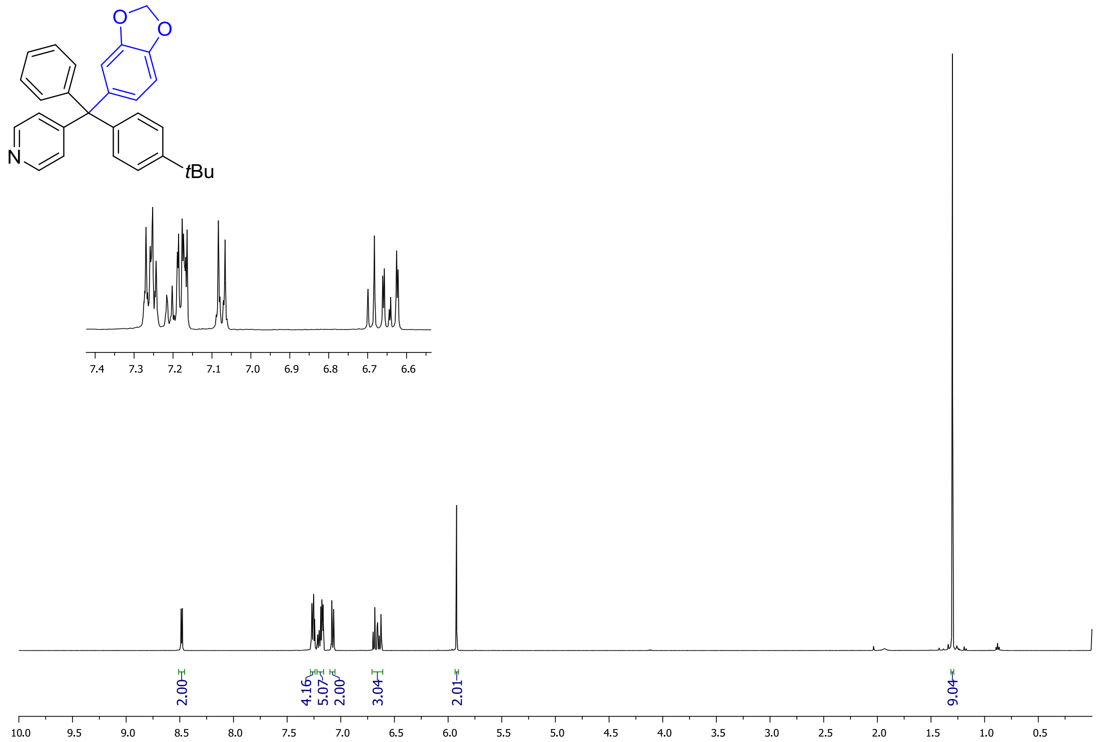

Supplementary Figure 68. ^{13}C NMR Spectrum of 6l (125 MHz, $CDCl_3$)

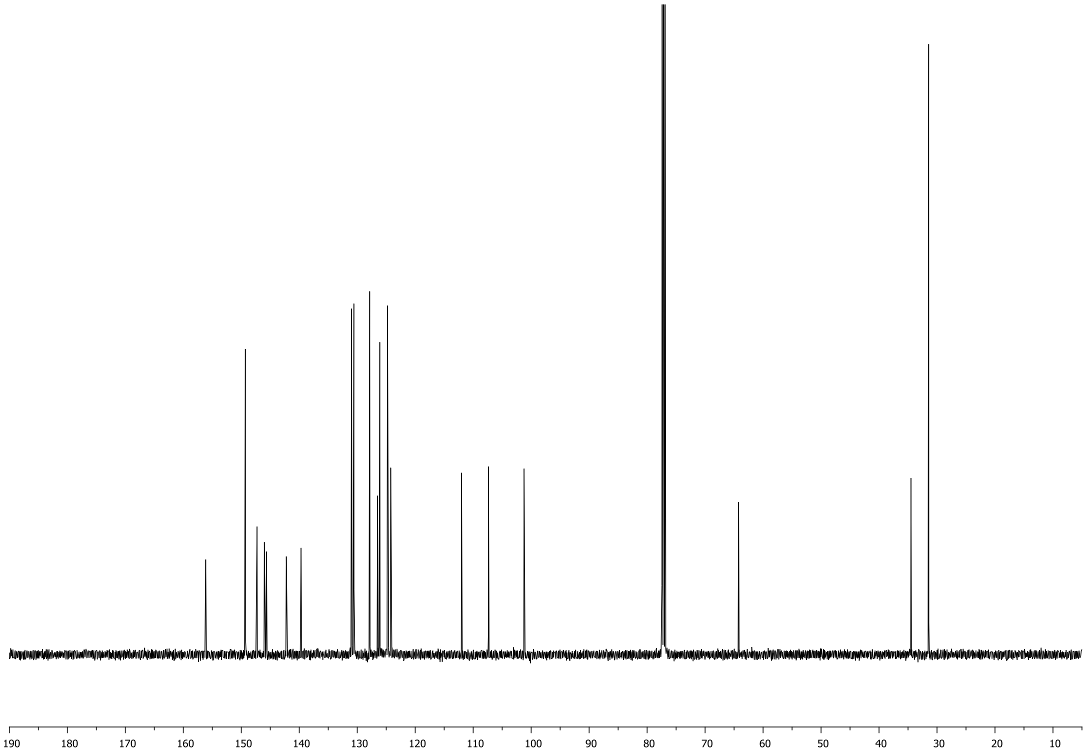

Supplementary Figure 69. ^{1}H NMR Spectrum of 6m (500 MHz, $CDCl_3$)

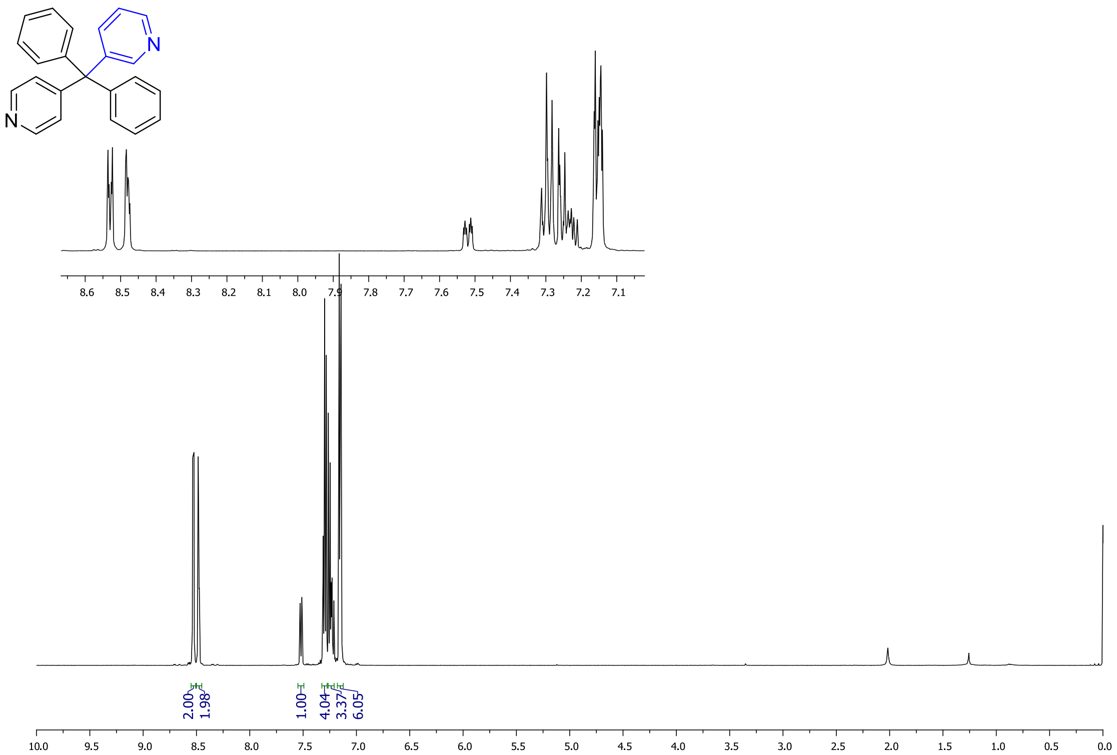

Supplementary Figure 70. ^{13}C NMR Spectrum of 6m (125 MHz, $CDCl_3$)

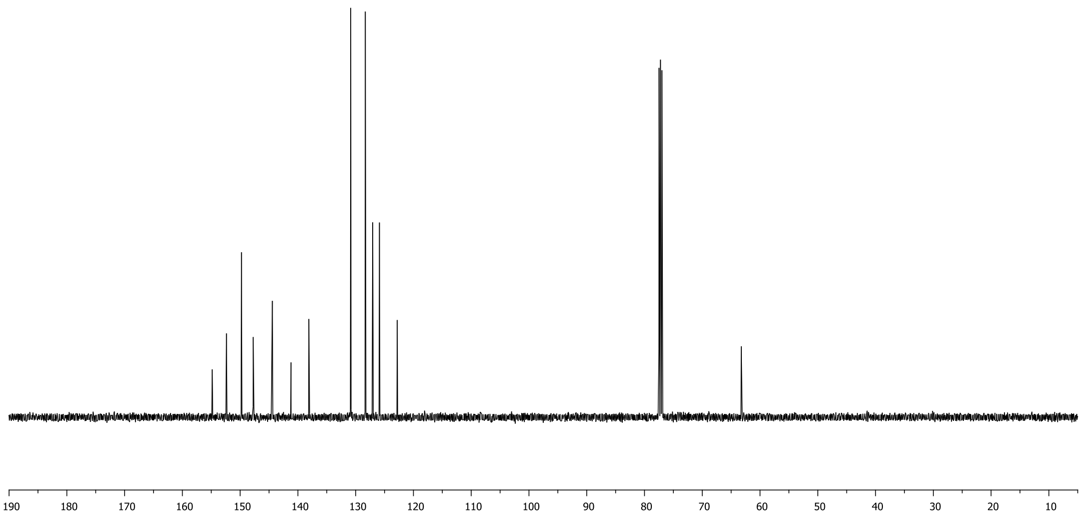

Supplementary Figure 71. ^{1}H NMR Spectrum of 6n (500 MHz, $CDCl_3$)

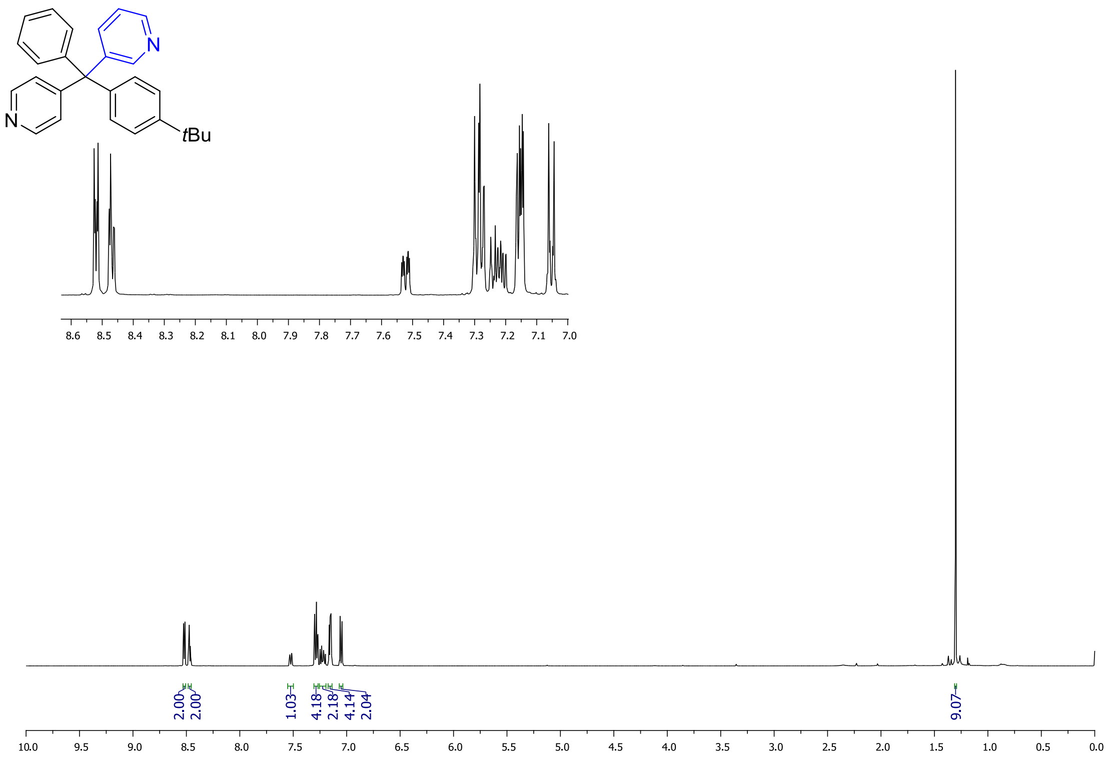

Supplementary Figure 72. ^{13}C NMR Spectrum of 6n (125 MHz, $CDCl_3$)

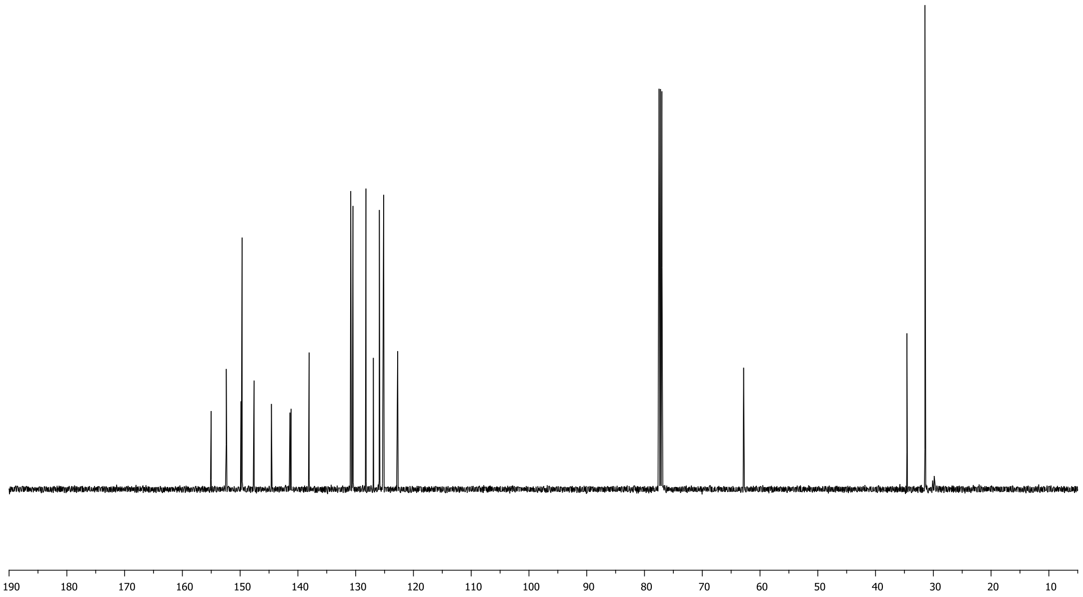

Supplementary Figure 73. ^{1}H NMR Spectrum of 6o (500 MHz, $CDCl_3$)

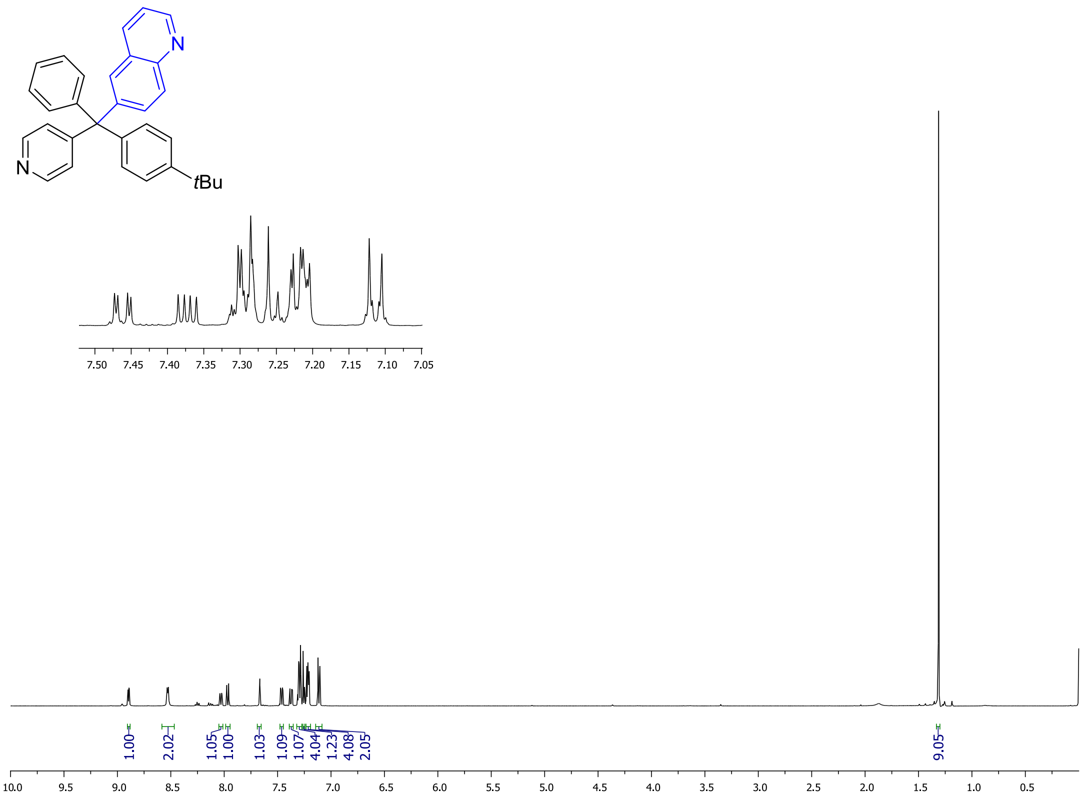

Supplementary Figure 74. ^{13}C NMR Spectrum of 6o (125 MHz, $CDCl_3$)

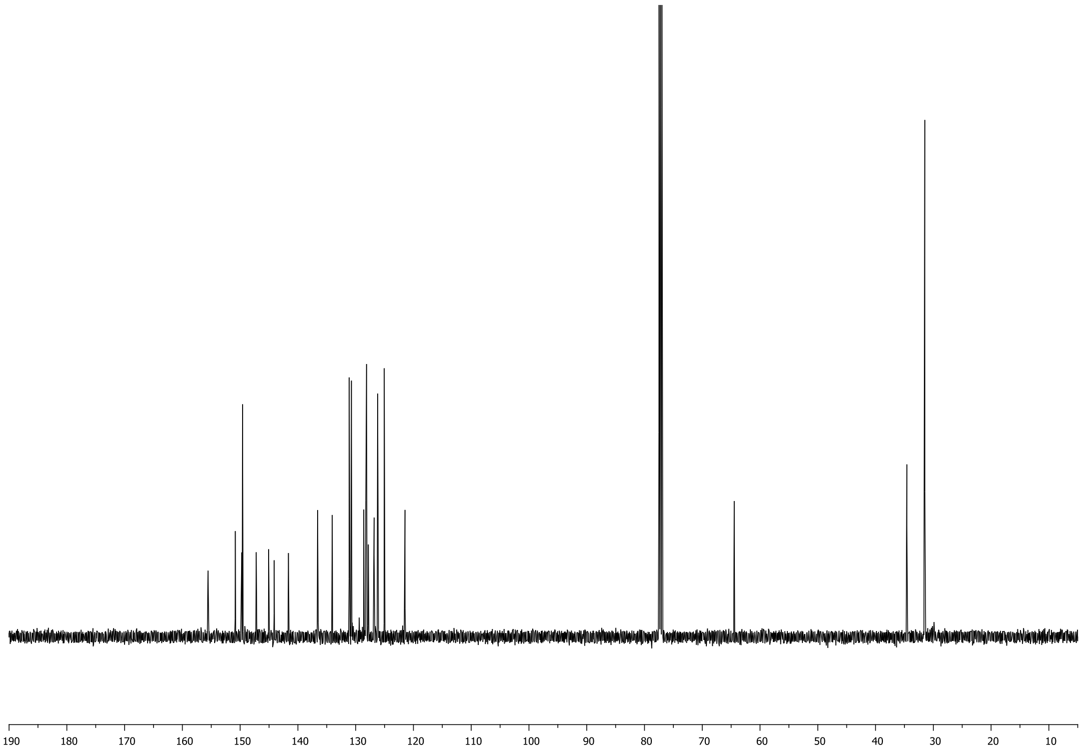

Supplementary Figure 75. ^{1}H NMR Spectrum of 6p (500 MHz, $CDCl_3$)

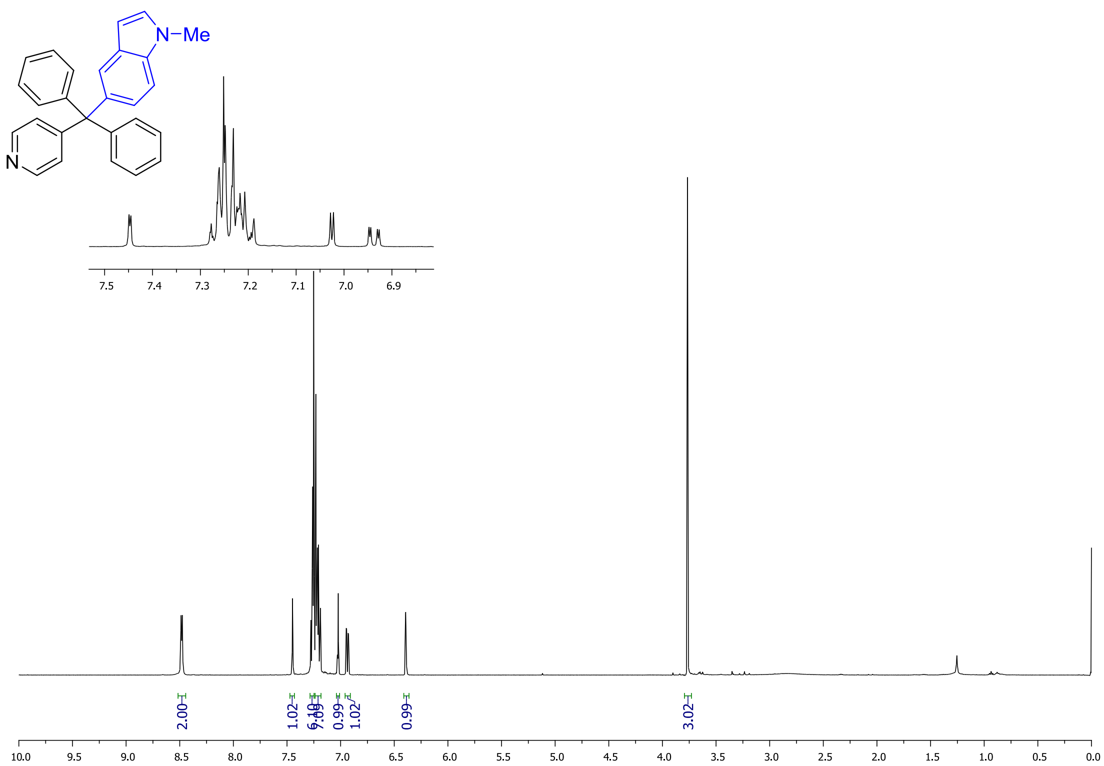

Supplementary Figure 76. ^{13}C NMR Spectrum of 6p (125 MHz, $CDCl_3$)

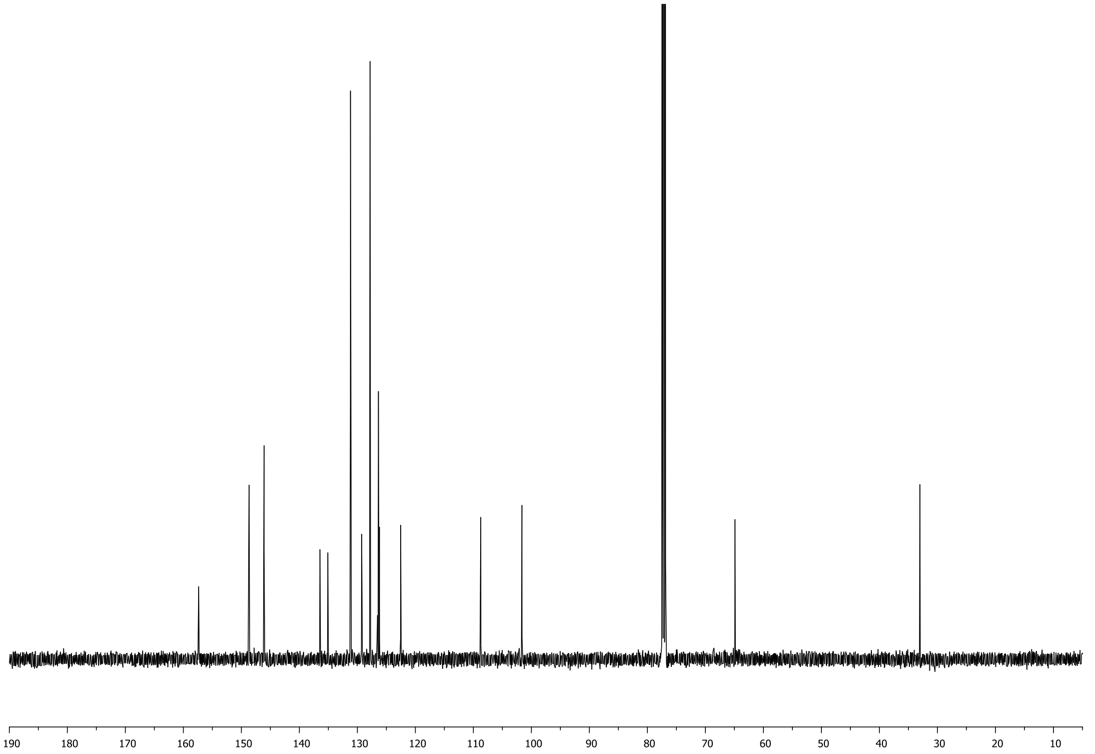

Supplementary Figure 77. ^{1}H NMR Spectrum of 6q (500 MHz, $CDCl_3$)

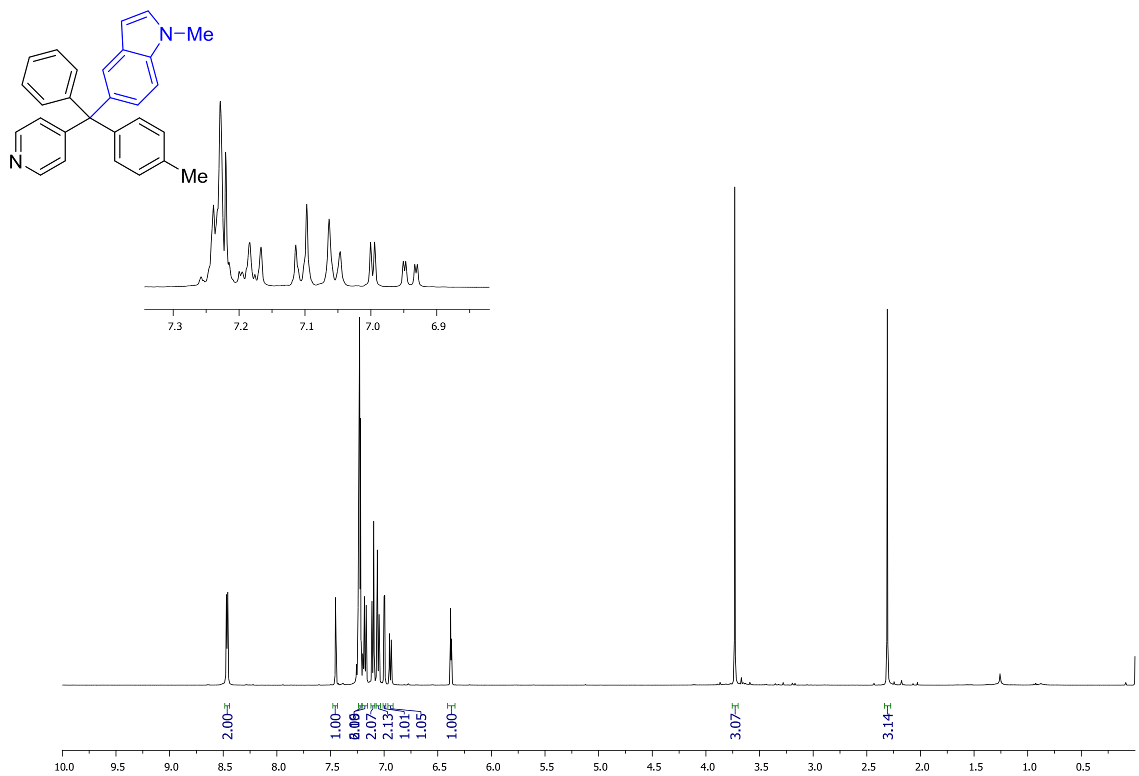

Supplementary Figure 78. ^{13}C NMR Spectrum of 6q (125 MHz, $CDCl_3$)

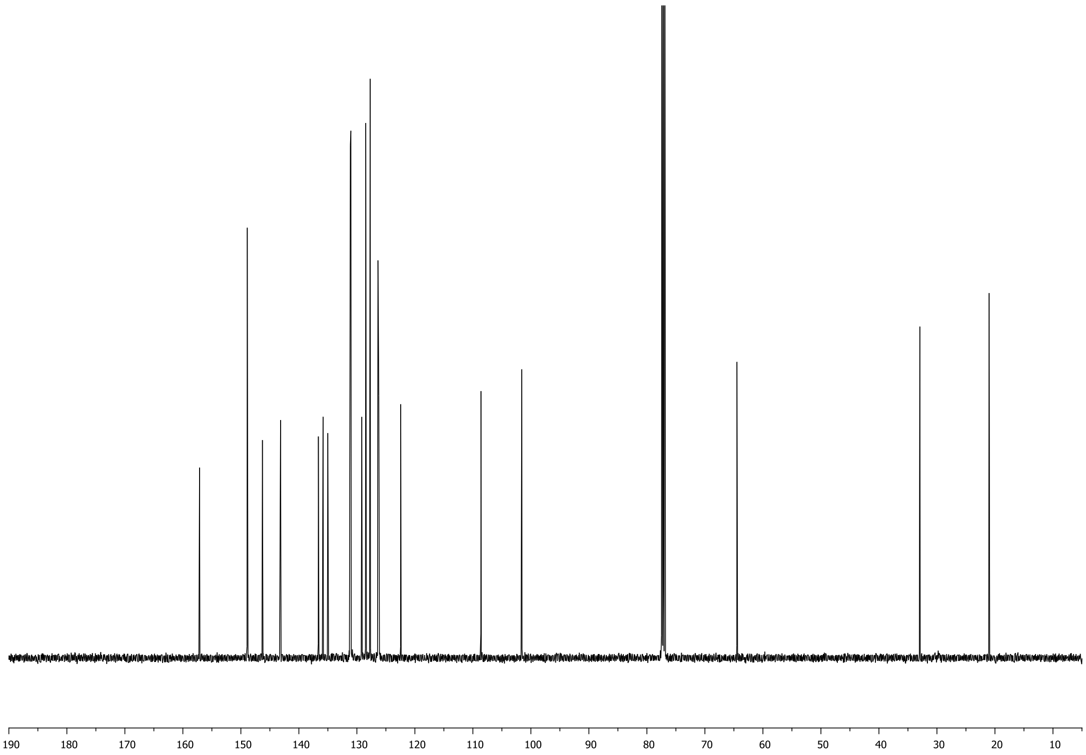

Supplementary Figure 79. ^{1}**H NMR Spectrum of 6r (500 MHz, CDCl$_3$)**

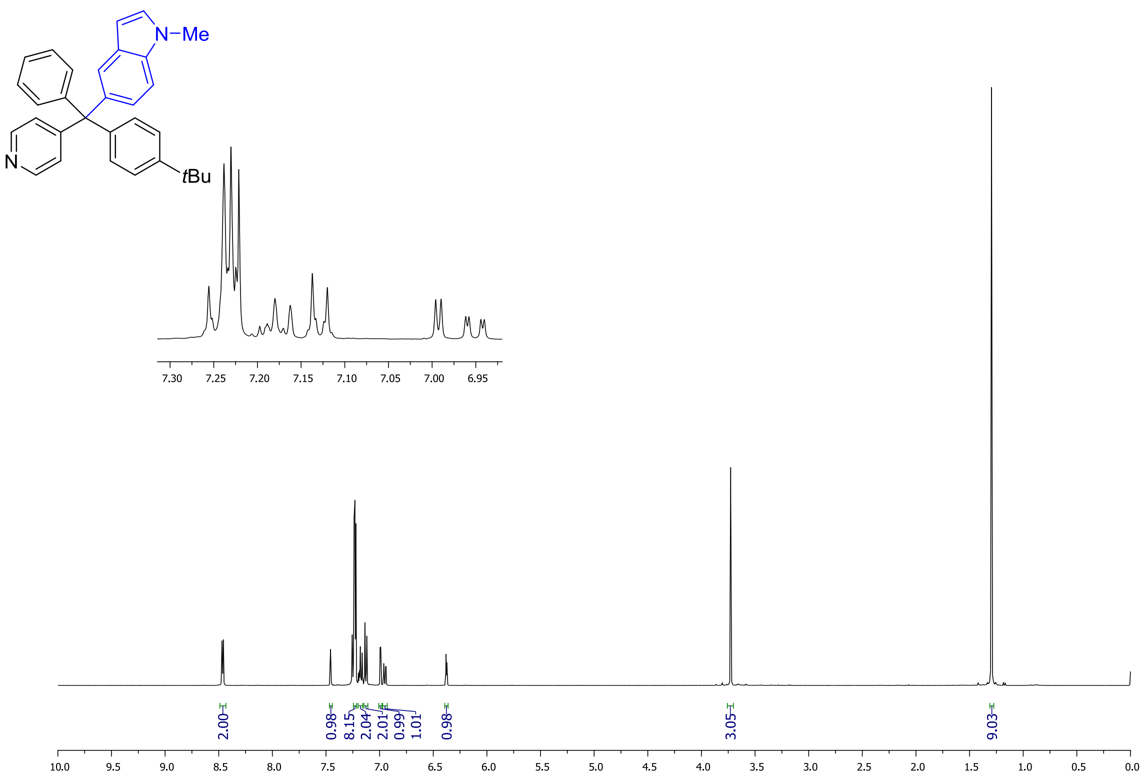

Supplementary Figure 80. ^{13}**C NMR Spectrum of 6r (125 MHz, CDCl$_3$)**

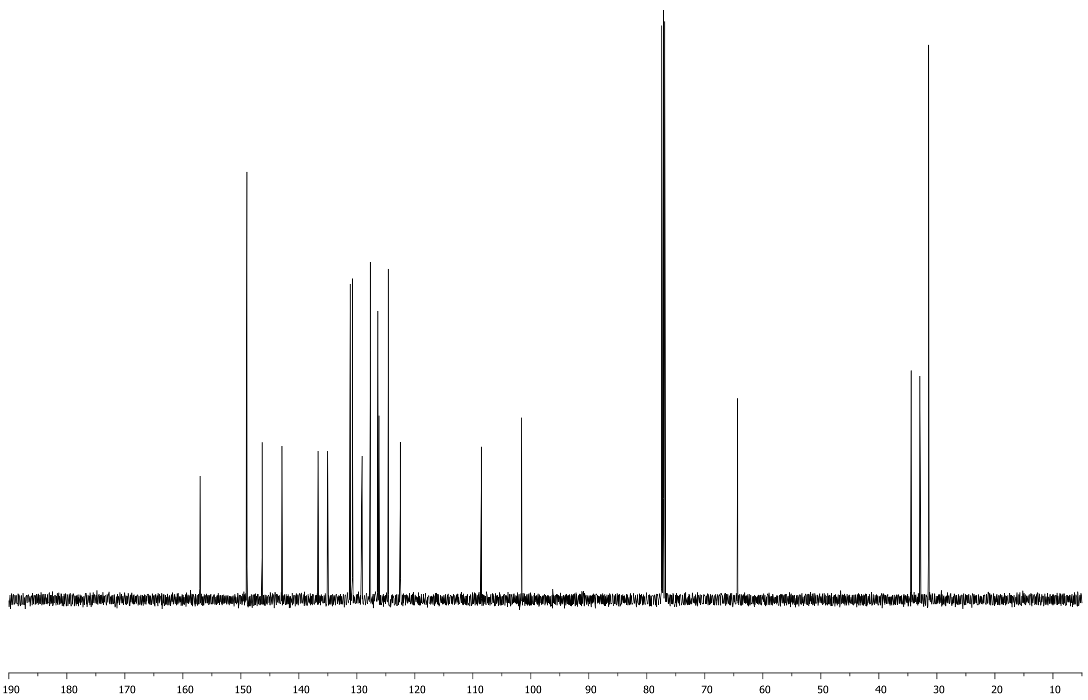

Supplementary Figure 81. ^{1}H NMR Spectrum of 6s (500 MHz, $CDCl_3$)

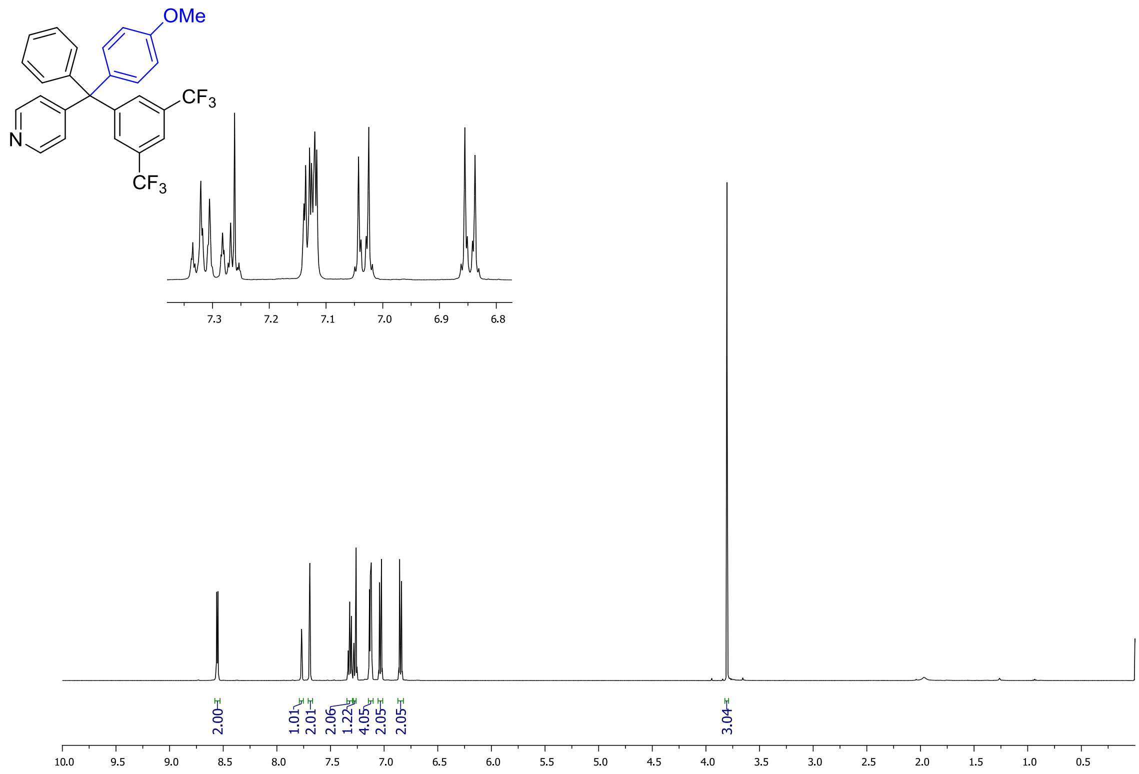

Supplementary Figure 82. ^{13}C NMR Spectrum of 6s (125 MHz, $CDCl_3$)

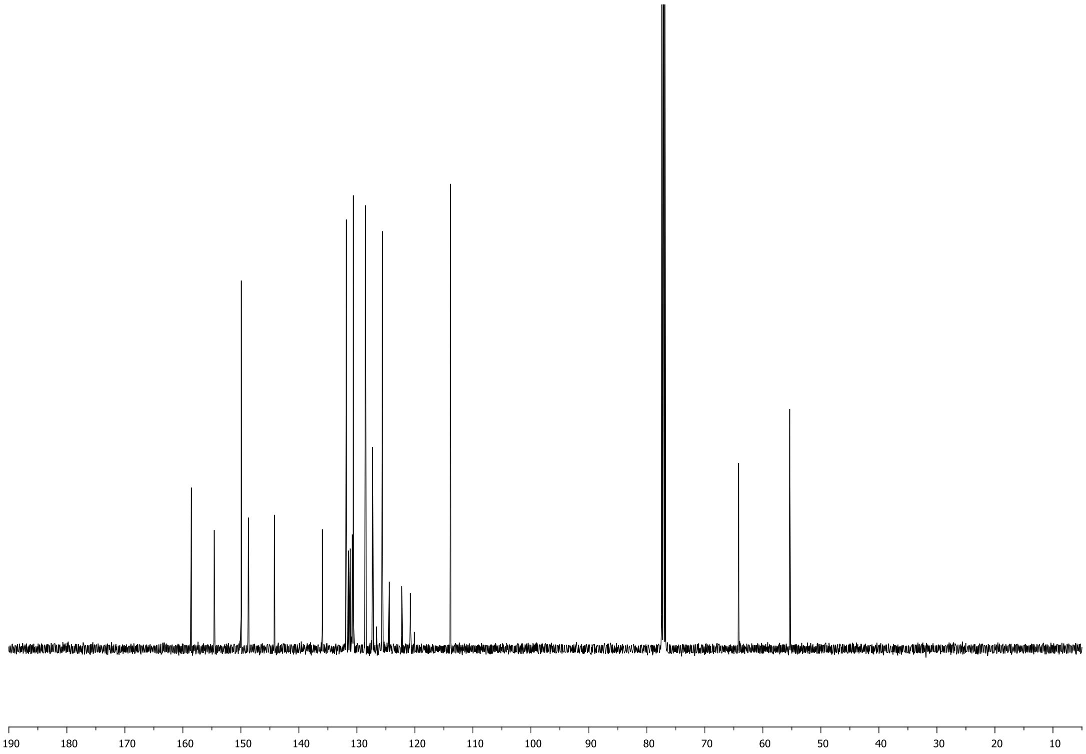

Supplementary Figure 83. ^{1}H NMR Spectrum of 6t (500 MHz, $CDCl_3$)

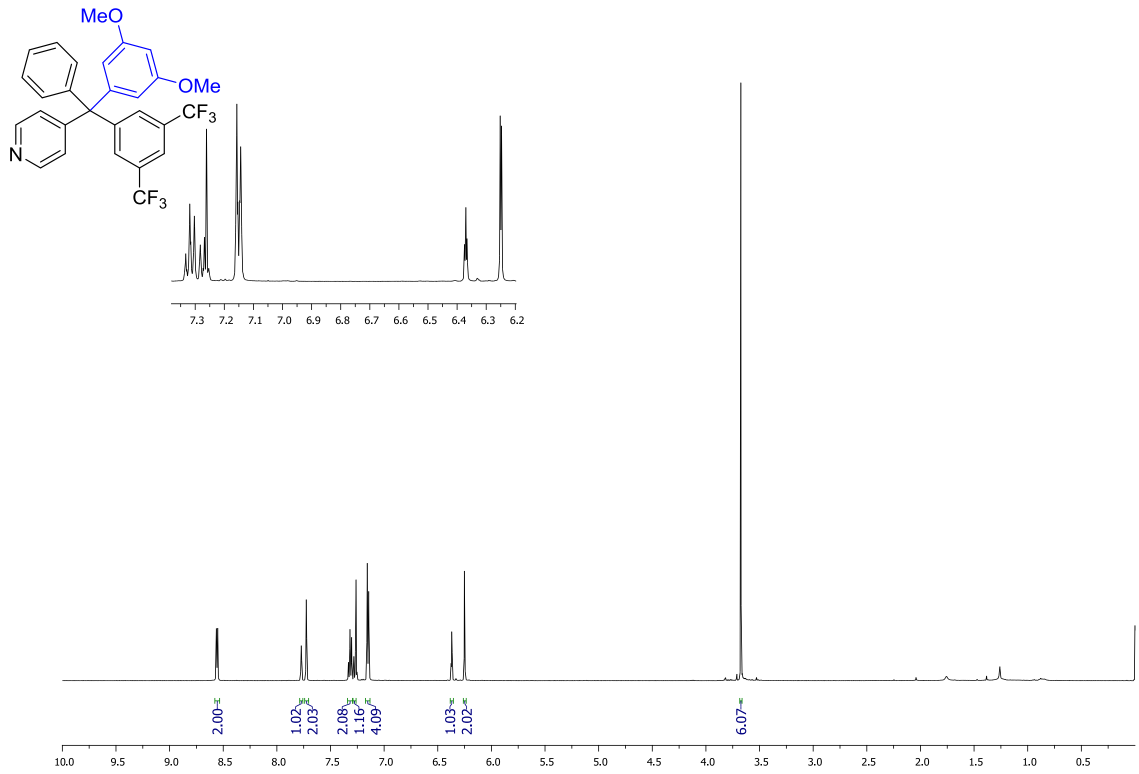

Supplementary Figure 84. ^{13}C NMR Spectrum of 6t (125 MHz, $CDCl_3$)

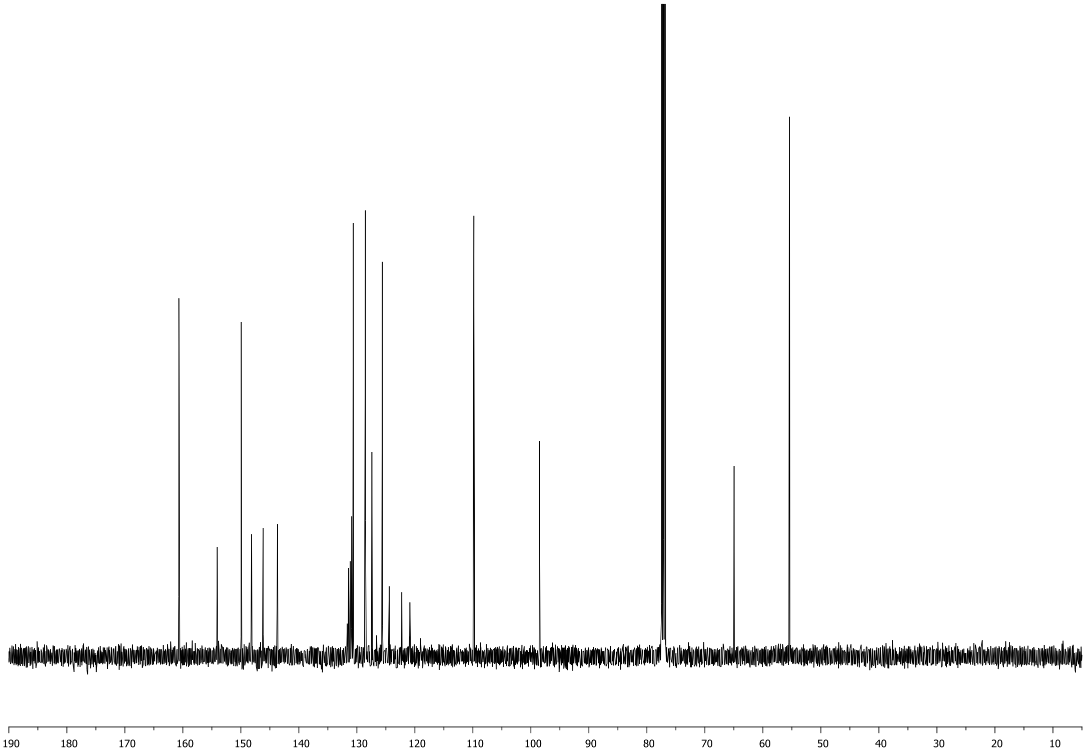

Supplementary Figure 85. ^{1}H NMR Spectrum of 9a (500 MHz, $CDCl_3$)

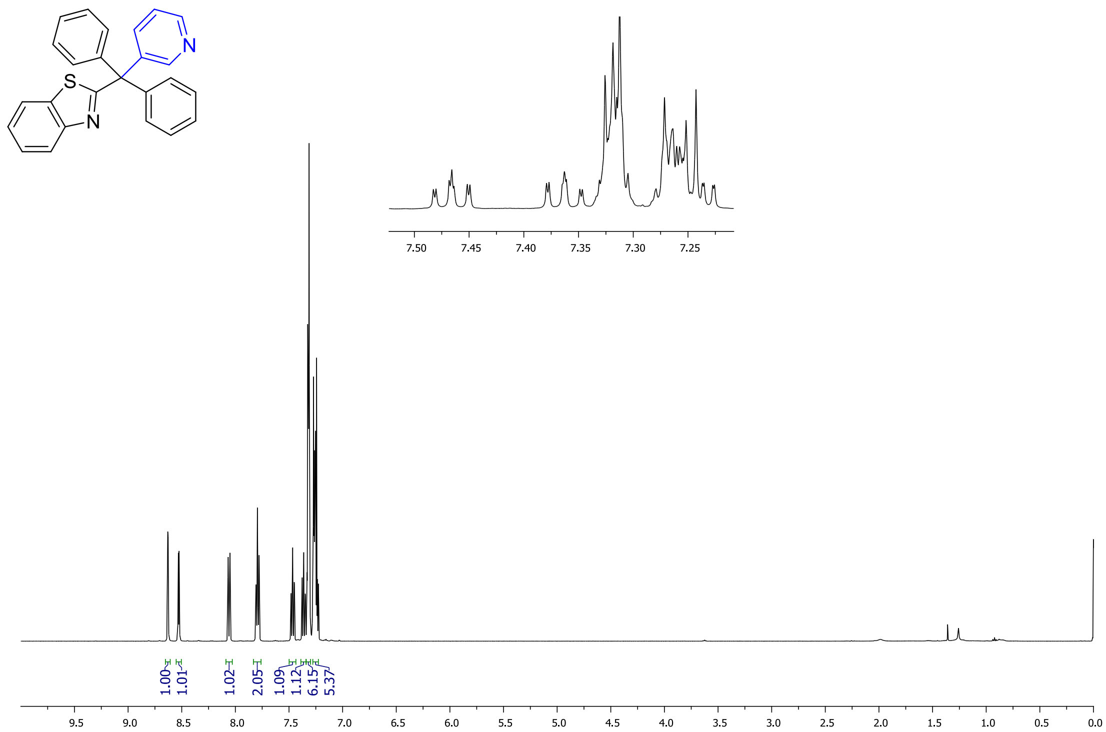

Supplementary Figure 86. ^{13}C NMR Spectrum of 9a (125 MHz, $CDCl_3$)

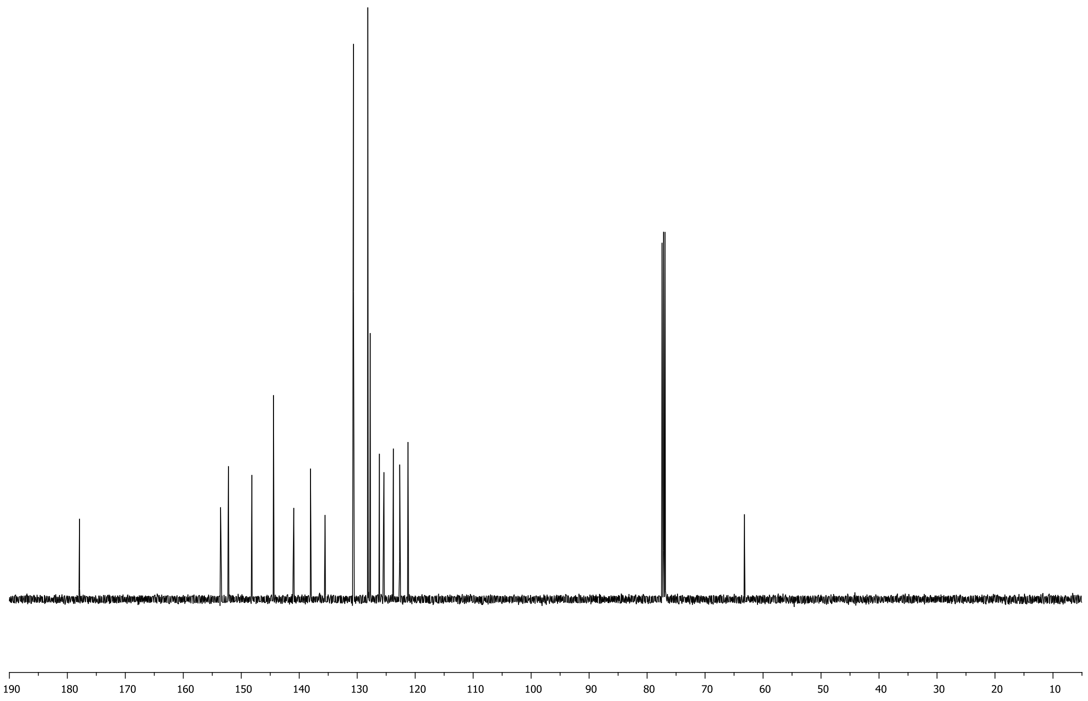

Supplementary Figure 87. ^{1}H NMR Spectrum of 9b (500 MHz, $CDCl_3$)

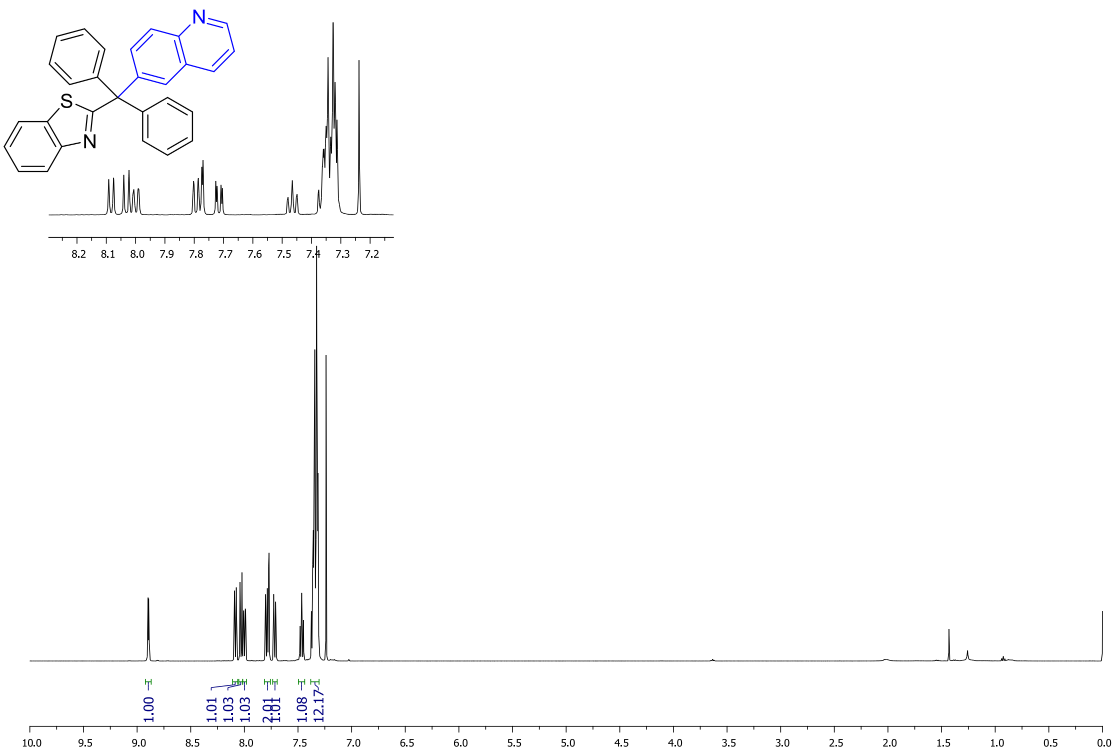

Supplementary Figure 88. ^{13}C NMR Spectrum of 9b (125 MHz, $CDCl_3$)

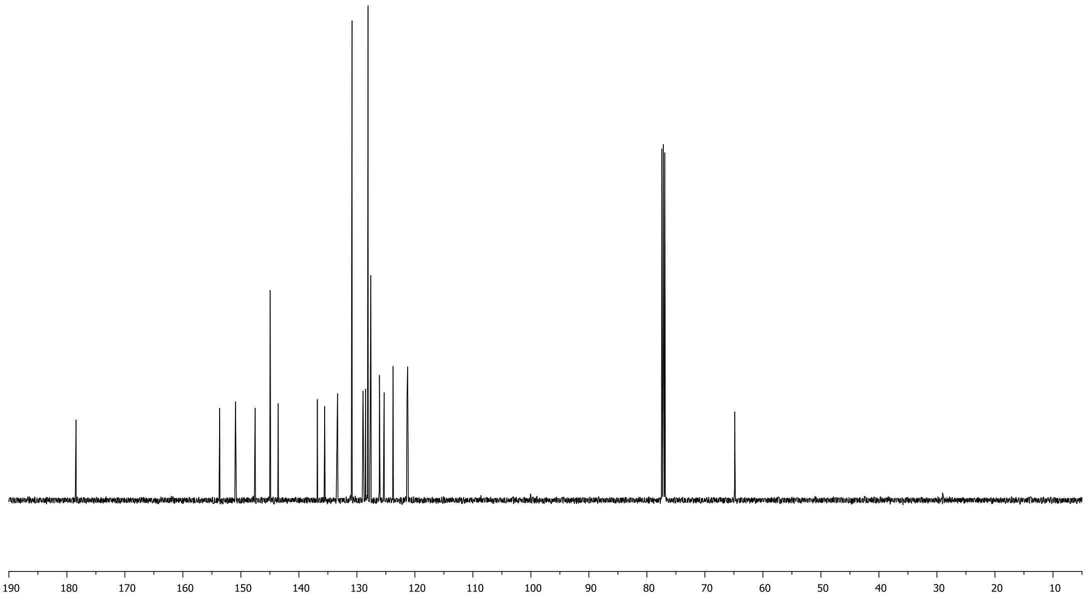

Supplementary Figure 89. ^{1}H NMR Spectrum of 9c (500 MHz, $CDCl_3$)

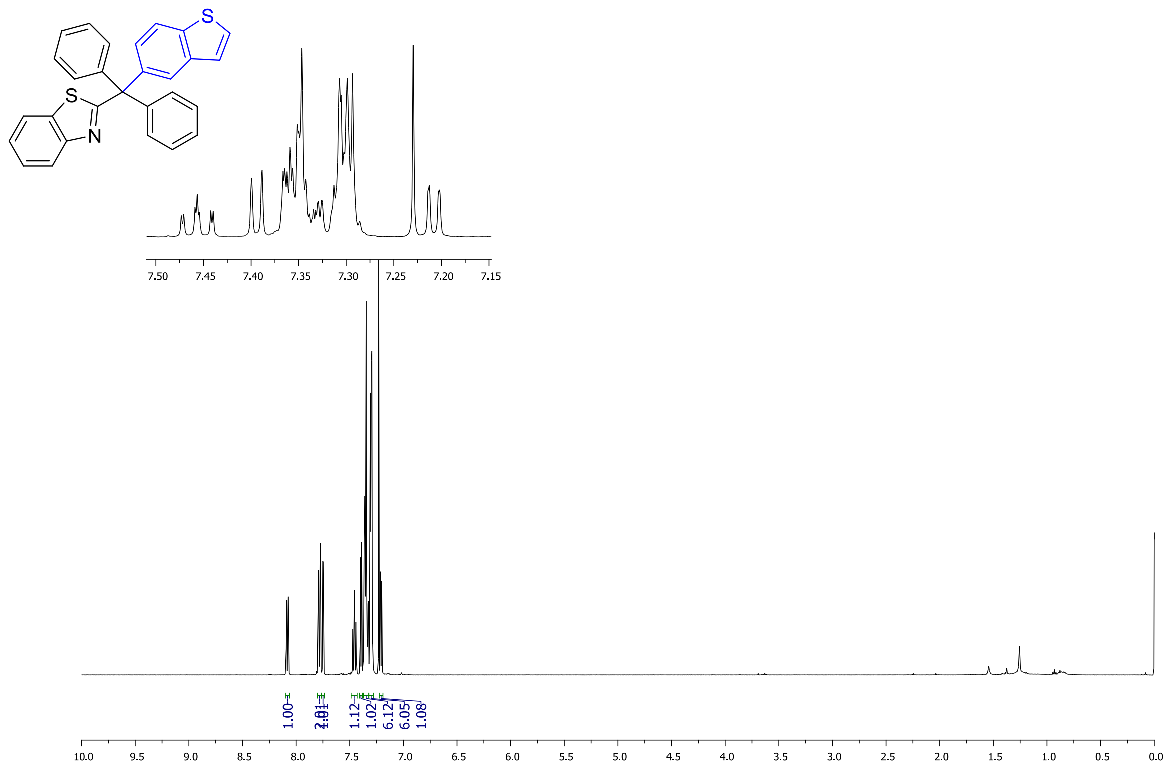

Supplementary Figure 90. ^{13}C NMR Spectrum of 9c (125 MHz, $CDCl_3$)

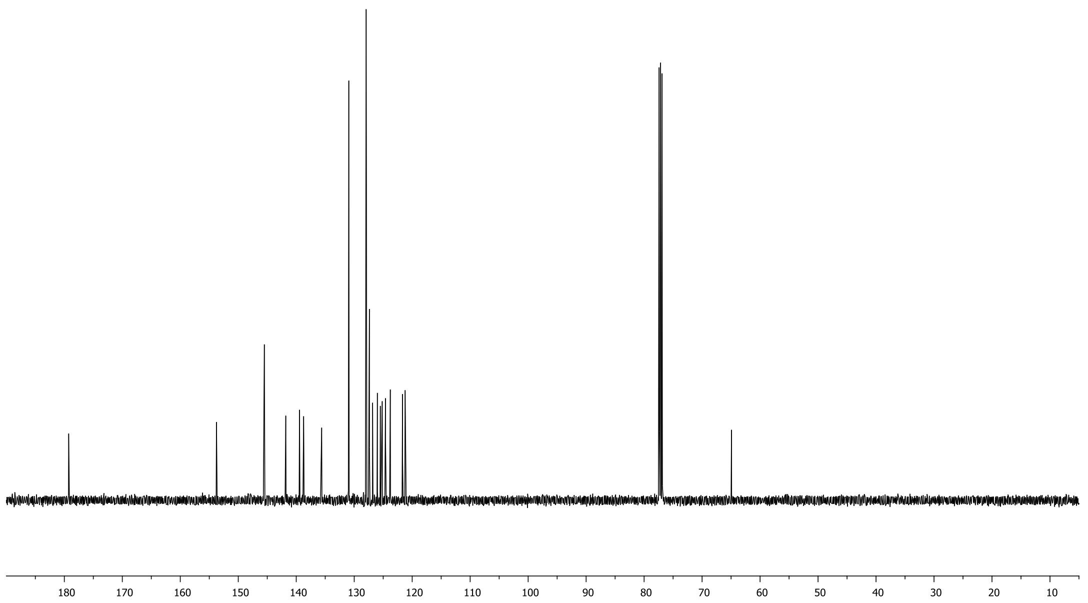

Supplementary Figure 91. ^{1}H NMR Spectrum of 9d (500 MHz, $CDCl_3$)

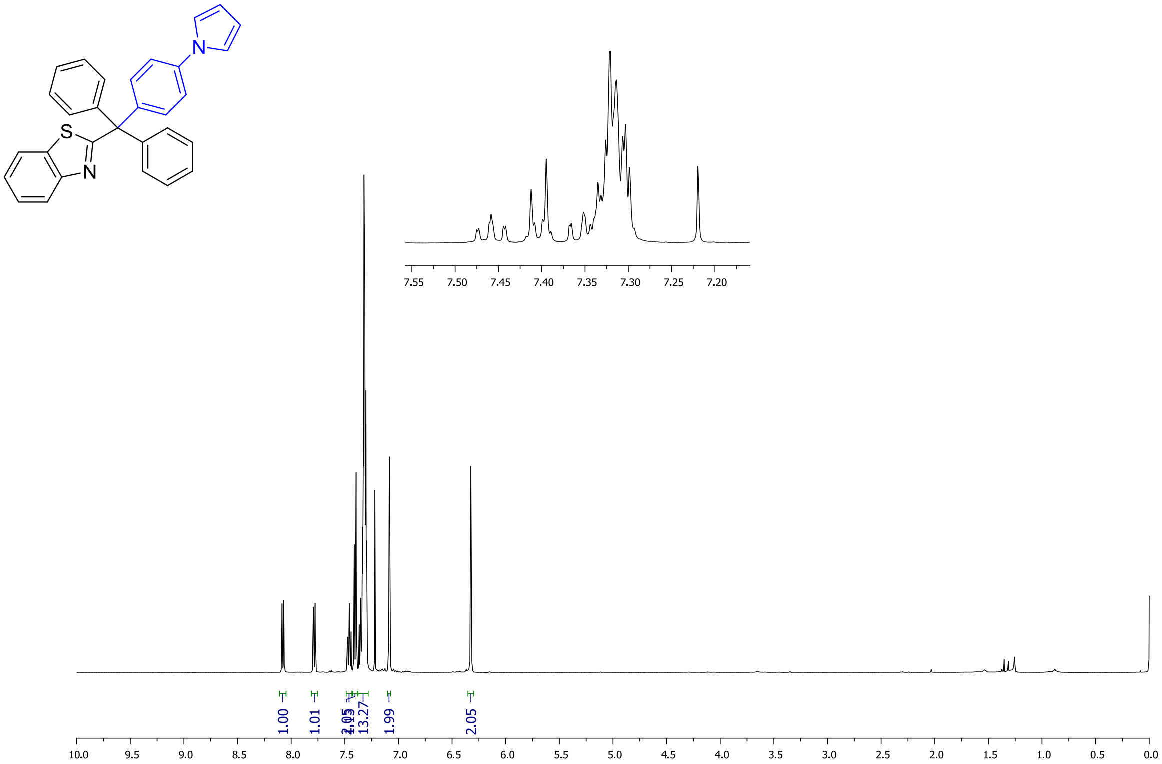

Supplementary Figure 92. ^{13}C NMR Spectrum of 9d (125 MHz, $CDCl_3$)

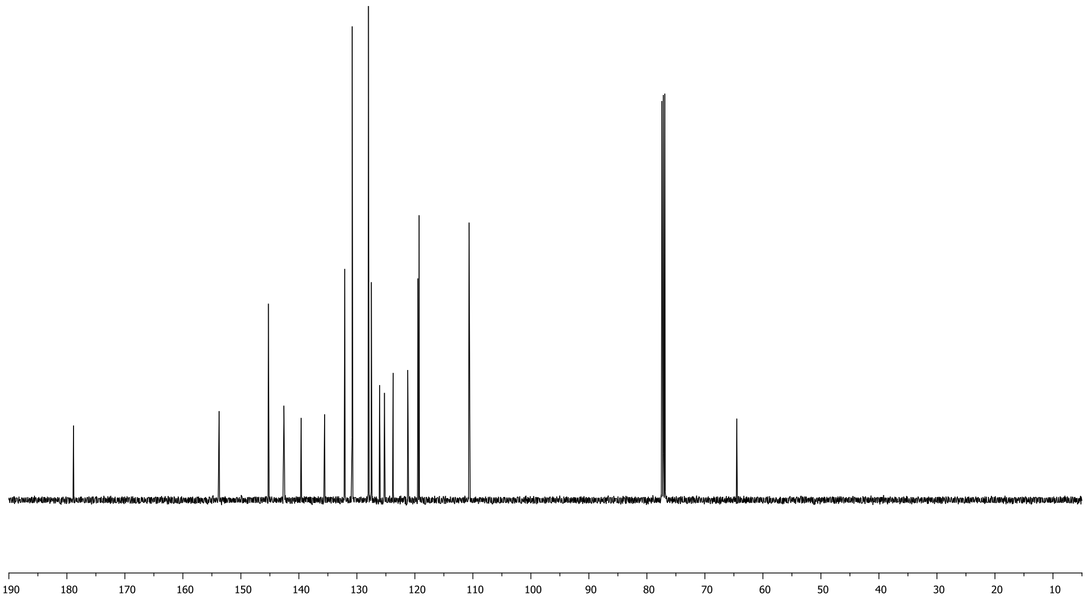

Supplementary Figure 93. ^{1}H NMR Spectrum of 9e (500 MHz, $CDCl_3$)

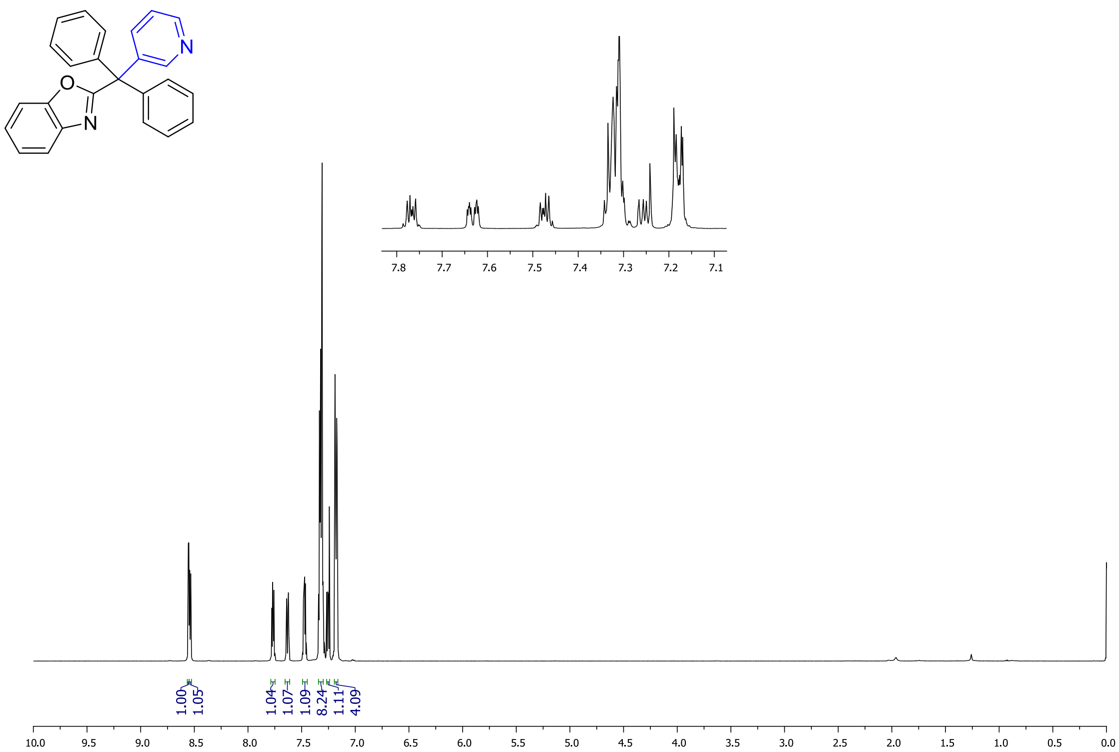

Supplementary Figure 94. ^{13}C NMR Spectrum of 9e (125 MHz, $CDCl_3$)

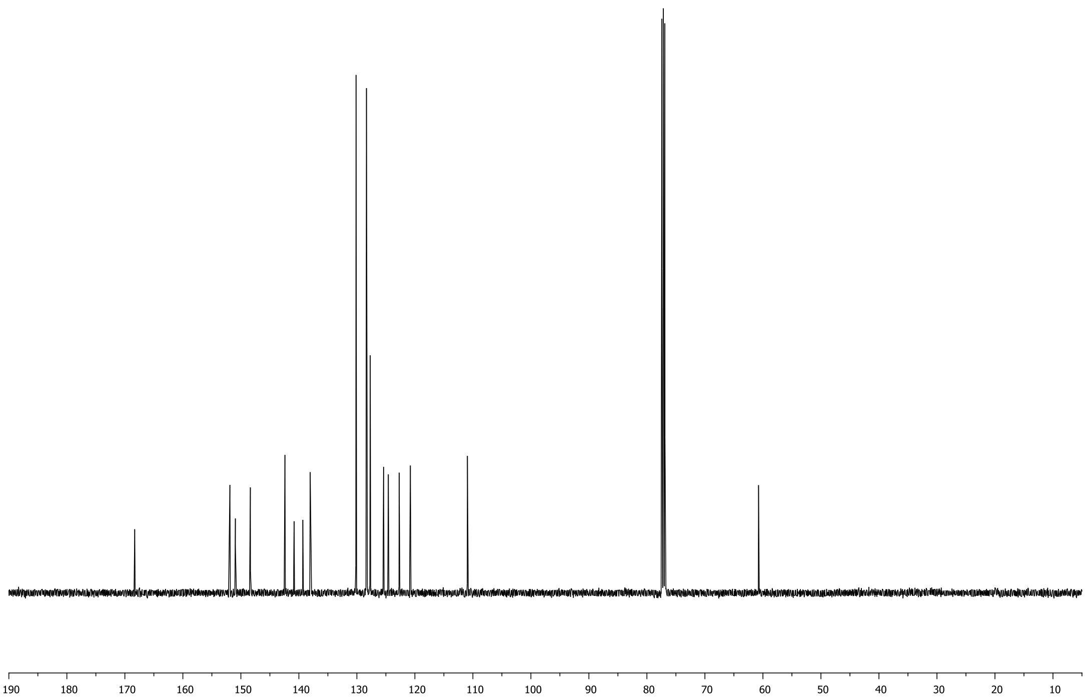

Supplementary Figure 95. ^{1}H NMR Spectrum of 9f (500 MHz, $CDCl_3$)

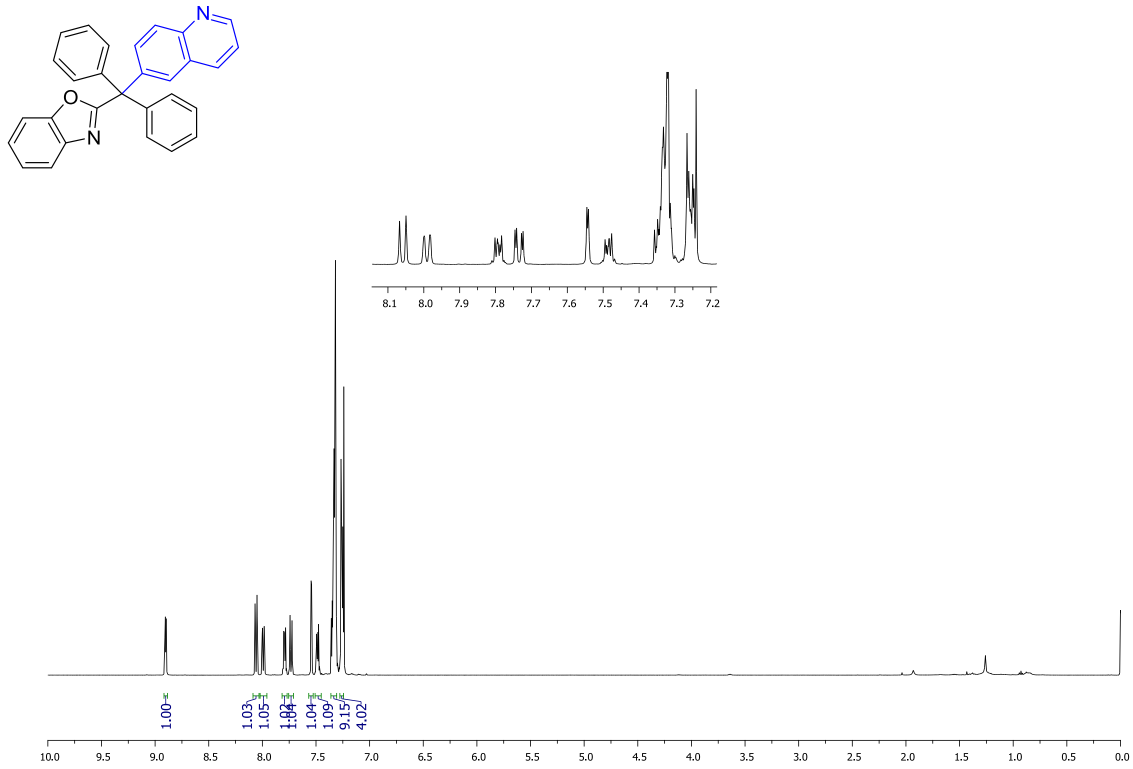

Supplementary Figure 96. ^{13}C NMR Spectrum of 9f (125 MHz, $CDCl_3$)

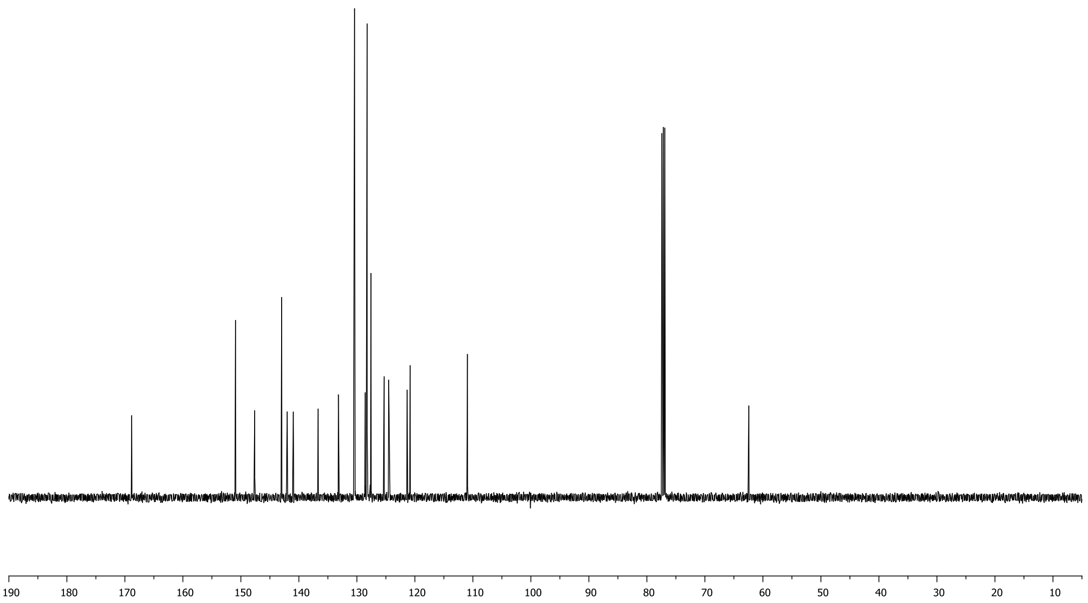

Supplementary Figure 97. ^{1}H NMR Spectrum of 9g (500 MHz, $CDCl_3$)

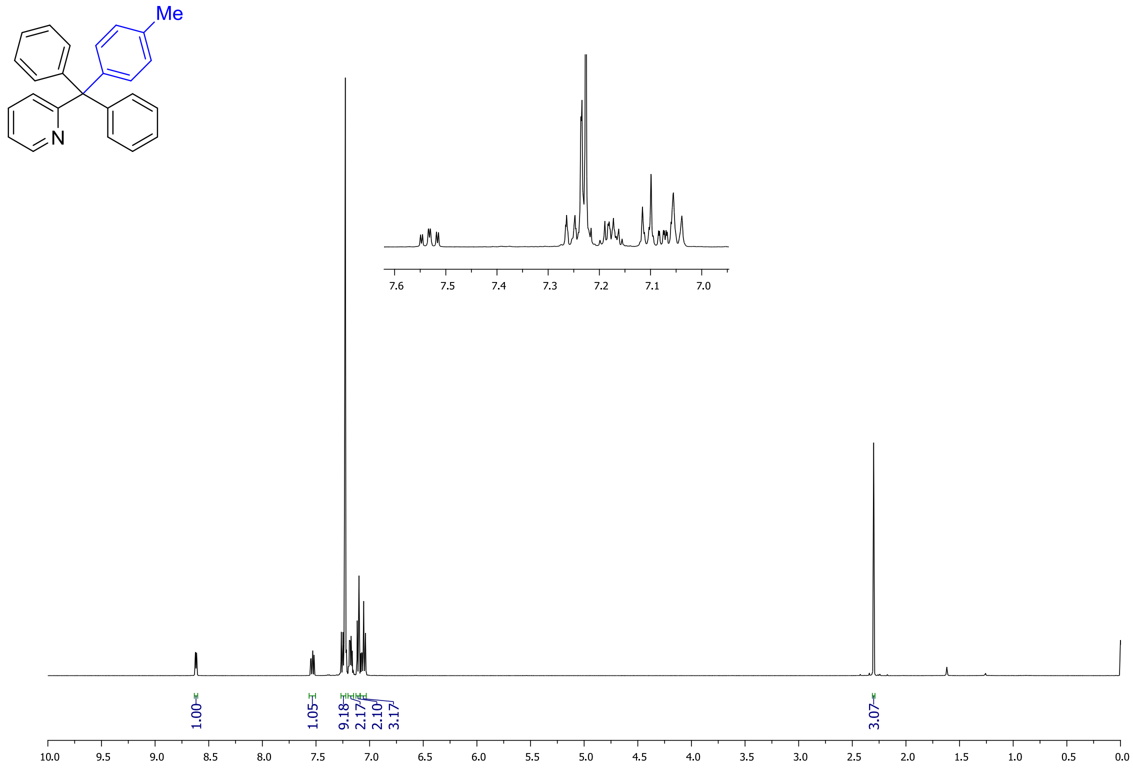

Supplementary Figure 98. ^{13}C NMR Spectrum of 9g (125 MHz, $CDCl_3$)

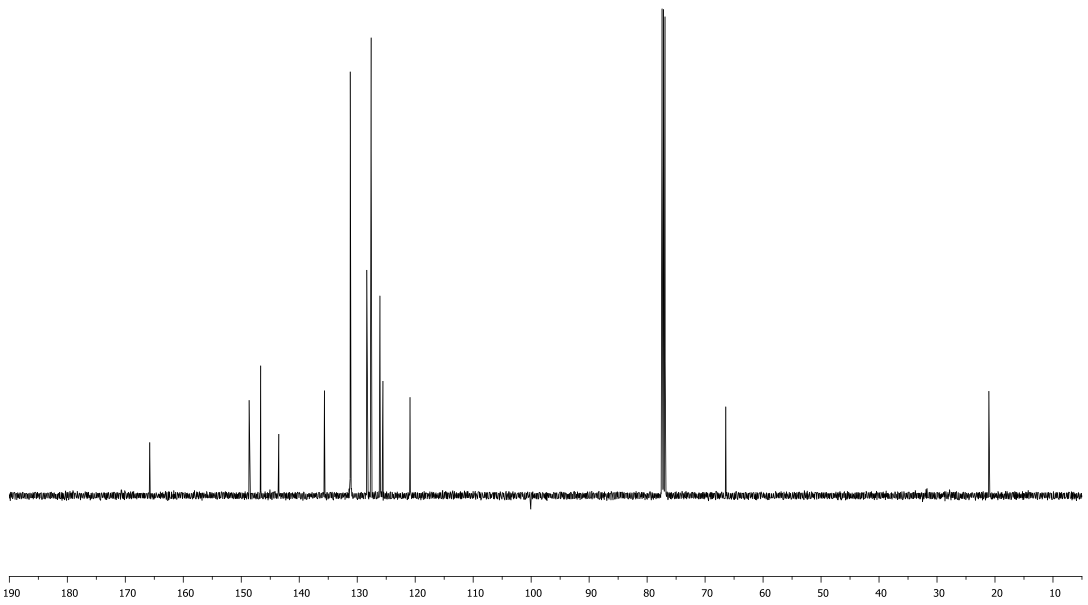

Supplementary Figure 99. ^{1}H NMR Spectrum of 10 (500 MHz, DMSO-d_6)

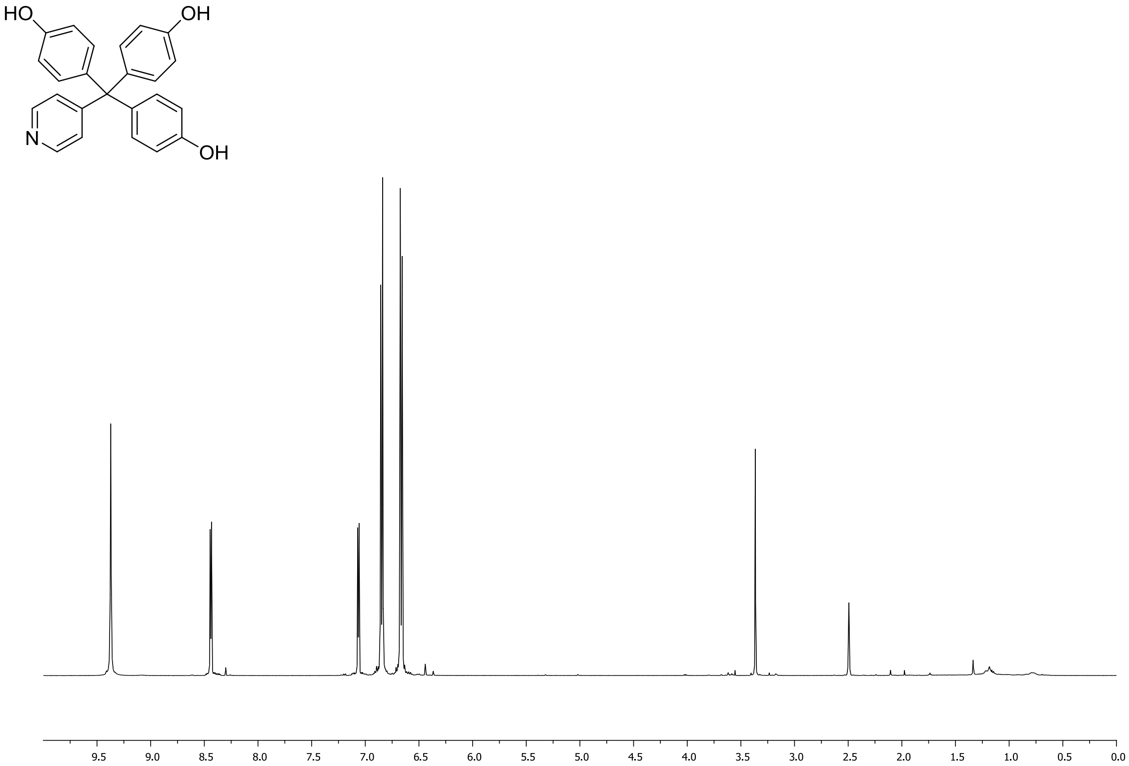

Supplementary Figure 100. ^{13}C NMR Spectrum of 10 (125 MHz, DMSO-d_6)

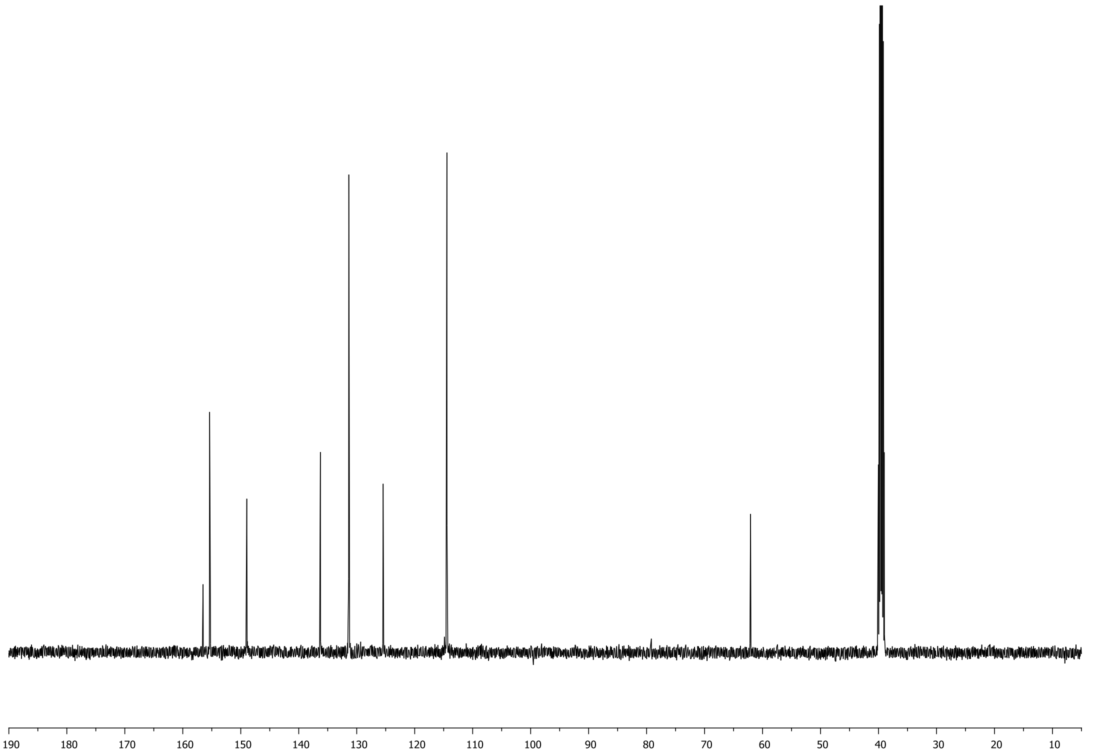

Supplementary Figure 101. ^{1}**H NMR Spectrum of 11 (500 MHz, CDCl$_3$)**

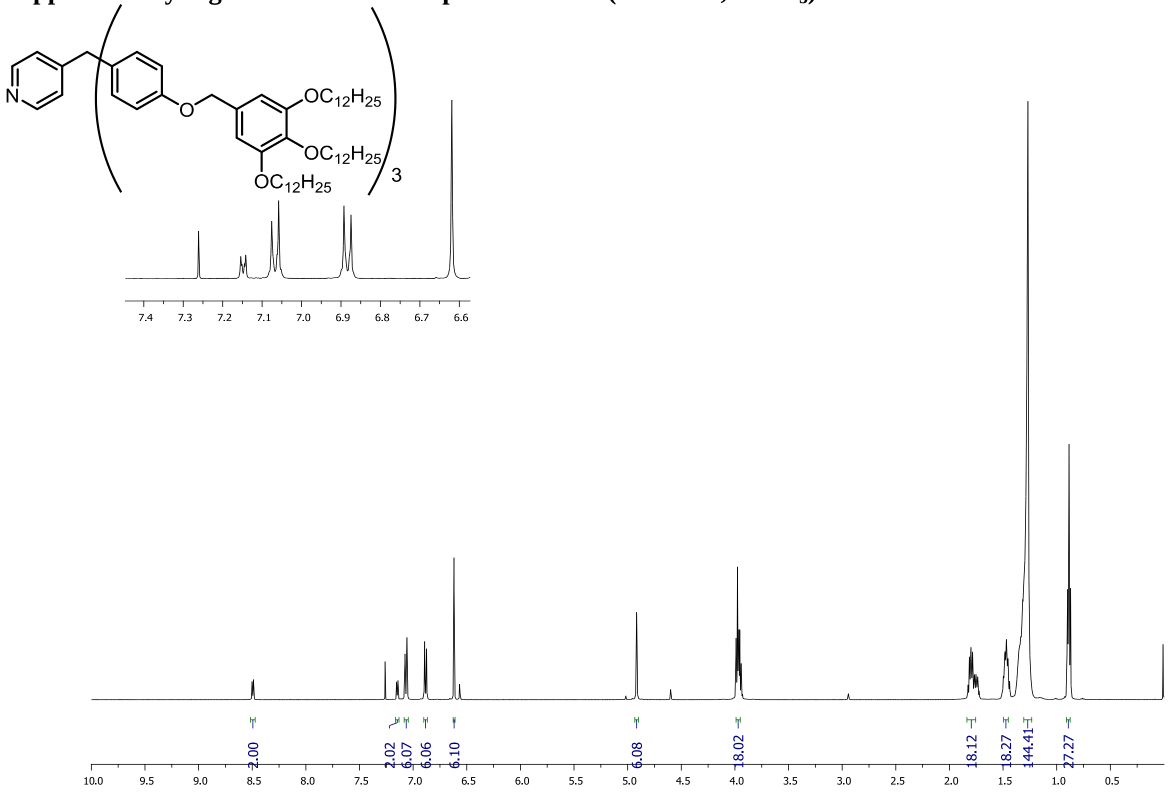

Supplementary Figure 102. ^{13}**C NMR Spectrum of 11 (125 MHz, CDCl$_3$)**

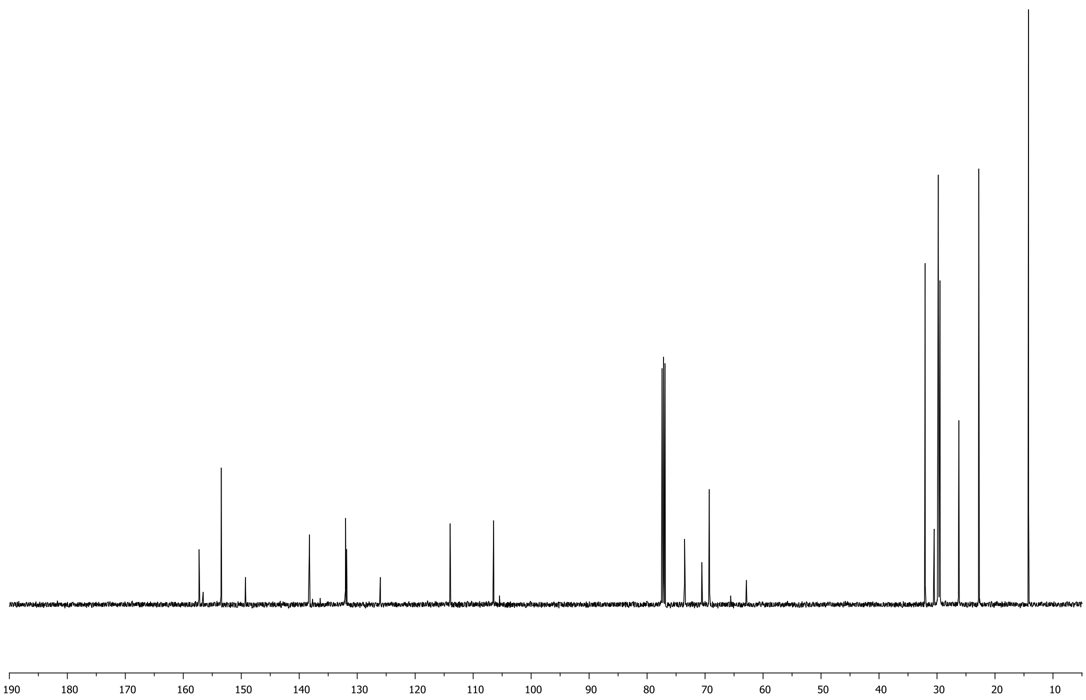

Supplementary Tables

Supplementary Table 1. High-throughput experimentation screenings for Pd-catalyzed diarylation of 4-benzylpyridine:

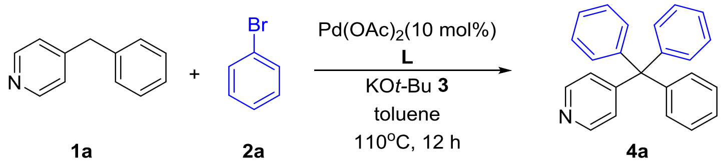

Ligands	Prod/IS
1–[2–[Bis(*t*–butyl)phosphino]phenyl]–3,5–diphenyl–1H–pyrazole (Trippyphos)	0.46
1,2,3,4,5–Pentaphenyl–1'–(di–*t*–butylphosphino)ferrocene (QPhos)	1.02
2–Dicyclohexylphosphino–2'–(*N*,*N*–dimethylamino)biphenyl (DavePhos)	0.31
2–Dicyclohexylphosphino–2',6'–dimethoxy–1,1'–biphenyl (SPhos)	**1.35**
N-(dicyclohexylphosphino)-2-(2'-methylphenyl)-1H-indole	0.23
5–(Di–*t*–butylphosphino)–1', 3', 5'–triphenyl–1'H–[1,4']bipyrazole (BippyPhos)	0.34
2–Di–*tert*-butylphosphino-3,4,5,6-tetramethyl-2′,4′,6′-triisopropyl-1,1′-biphenyl (Me-4–*t*–Bu XPhos)	0.69
N–phenyl–2–(dicyclohexylphosphino)pyrrole (CataCXium PCy)	0.18
Tri(furan-2-yl)phosphine	0.49
2-Di-*tert*-butylphosphino-1,1'-binaphthyl (TrixiePhos)	0.64
2-Dicyclohexylphosphino-2'-methylbiphenyl (MePhos)	0.31
2-(Dicyclohexylphosphino)-1-phenylindole (CataCXium PInCy)	0.22
2–Di–tert–butylphosphino–3–Methoxy–6–Methyl–2'–4'–6'–triisopropylbiphenyl (RockPhos)	0.50
1,1′-Bis(dicyclohexylphosphino)ferrocene (DPPF)	0.76
2-Di-tert-butylphosphino-2'-methylbiphenyl (^{t}Bu-MePhos)	0.26
2–(Di–*t*–butylphosphino)biphenyl (JohnPhos)	0.29
4,6–Bis(diphenylphosphino)phenoxazine (NIXANTPHOS)	0.37
2-Di-tert-butylphosphino-2′,4′,6′-triisopropylbiphenyl (tbu Xphos)	0.40

(1,1′-Binaphthalene-2,2′-diyl)bis(diphenylphosphine) (BINAP)	0.27
1,1′-Bis(di-*t*-butylphosphion)ferrocene (DTBPF)	1.08
1,1′-Bis(diisopropylphosphino)ferrocene (DiPPF)	0.62
Triphenylphosphine	0.57
9-[2-(Dicyclohexylphosphino)phenyl]-9H-carbazole (CyPhenCarPhos)	0.91
Tri(*o*-tolyl)phosphine	0.34
Di(1-adamantyl)-2-dimethylaminophenylphosphine (MeDalPhos)	0.32
2,2′-Bis(diphenylphosphino)-1,1′-biphenyl (BiPHEP)	0.43
Bis[(2–diphenylphosphino)phenyl] ether (DCEPhos)	0.28
Tricyclohexylphosphinetetrafluoroborate (PCy_3 HBF_4)	**2.50**
Bis(diphenylphosphinophenyl)ether (DPEPhos)	0.79
Di-*tert*-butylneopentylphosphoniumtetrafluoroborate	0.37
4,5-Bis(diphenylphosphino)-9,9-dimethylxanthene (XantPhos)	0.84
2-(Dicyclohexylphosphino)-3,6-dimethoxy-2′,4′,6′-triisopropyl-1,1′-biphenyl (BrettPhos)	0.49
Dicyclohexylphenylphospine	0.60
2-(Di-*tert*-butyl-phosphino)-1-phenyl-1*H*-pyrrole (CataCXium PtB)	0.49
Di(1-adamantyl)-*n*-butylphosphine (CataCXium A)	**4.74**
2-(Dicyclohexylphosphino)-1-(2,4,6-trimethyl-phenyl)-1*H*-imidazole (CataCXium PICy)	0.32
2-Dicyclohexylphosphino-2′,4′,6′-triisopropylbiphenyl (XPhos)	0.44
N,*N*'-dicyclohexyl-1-diphenylphosphanyl-formamidine (DCyF)	0.30
(2-Biphenyl)dicyclohexylphosphine (CyJohnPhos)	0.24
2-(Di-tert-butylphosphino) -2′,4′,6′-triisopropy-3,6-dimethoxy-1,1′-biphenyl (di*t*BuBreetPhos)	0.51
Sodium 2′-dicyclohexylphosphino-2,6-dimethoxy-1,1′-biphenyl-3-sulfonate hydrate (^{s}SPhos)	0.31
Di(1-adamantyl)benzylphosphine (CataCXium ABn)	0.25
2-Dicyclohexylphosphino-2′,6′-diisopropoxybiphenyl (RuPhos)	0.58
N,*N*-Dimethyl 4-(Di(*tert*-butyl)phosphino)aniline (A^{ta}Phos)	**1.46**
2′-(Dicyclohexylphosphino)acetophenone ethylene ketal (SymPhos)	0.45

Tri(3,5-dimethylphenyl)phosphine	**1.36**
(2-Biphenyl)di-*tert*-butylphosphine (JohnPhos)	0.29
N,*N*-Dimethyl 4-(Di(phenyl)phosphino)aniline (A^{pa}Phos)	0.84

Set up:

Experiments were set up inside a glovebox under a nitrogen atmosphere. Two 24-well aluminum blocks containing 1 mL glass vials were predosed with $Pd(OAc)_2$ (1.0 μmol) and the phosphine ligands (2 μmol for monodentate ligands and 1 μmol for bidentate ligands) in THF. The solvent was removed to dryness using a GeneVac and KO*t*-Bu (40 μmol) in THF was added to the ligand/catalyst mixture. The solvent was removed on the GeneVac and a parylene stir bar was then added to each reaction vial. 4-Benzylpyridine (**1a**, 10 μmol/reaction) and bromobenzene (**2a**, 40 μmol/reaction) were then dosed together into each reaction vial as a solution in toluene (100 μL, 0.1 M). The 24-well plates were then sealed and stirred for 12 h at 110 ^{o}C.

Work up:

Upon opening the plate to air, 500 μL of a solution of 4,4'-di-*tert*-butylbiphenyl (used as internal standard to measure HPLC yields) in acetonitrile (0.002 mol/L) was added into each vial. The plate was covered again and the vials stirred for 10 min to ensure good homogenization. Into a separate 96-well LC block was added 700 μL of acetonitrile, followed by 25 μL of the diluted reaction mixtures. The LC block was then sealed with a silicon-rubber storage mat and mounted on an automated UPLC instrument for analysis. The results are presented in Supplementary Table 1.

Supplementary Methods

General Information

All air- and moisture-sensitive solutions and chemicals were handled under nitrogen or in nitrogen filled glovebox and solutions were transferred via syringe. Dry THF, 1, 4-dioxane, DME, toluene and CPME were purchased from Sigma-Aldrich and used without further purification. Unless otherwise stated, reagents were commercially available and used as purchased. Chemicals were obtained from Sigma-Aldrich, Acros and Alfa-Aesar and solvents were purchased from Fisher Scientific. TLC was performed with Merck TLC Silicagel60 F_{254} plates with detection under

UV light at 254 nm. Silica gel (230–400 mesh, Silicycle) was used for flash chromatography. The ^{1}H NMR and $^{13}C\{^{1}H\}$ NMR spectra were obtained using a Brüker AM-500 Fourier-transform NMR spectrometer at 500 and 125 MHz, respectively. Chemical shifts are reported parts per million (ppm) referenced to tetramethylsilane (TMS, δ 0.00 ppm for) ^{1}H NMR, $CDCl_3$ (δ 77.16 ppm) for $^{13}C\{^{1}H\}$ NMR. Chemical shifts are reported in units of parts per million (ppm) downfield from tetramethylsilane (TMS) and all coupling constants are reported in hertz. The infrared spectra were obtained with KBr plates using a Perkin-Elmer Spectrum 100 Series FTIR spectrometer. High resolution mass spectrometry (HRMS) data were obtained on a Waters LC-TOF mass spectrometer (model LCT-XE Premier) using chemical ionization (CI) or electrospray ionization (ESI) in positive or negative mode, depending on the analyte. Melting points were determined on a Unimelt Thomas-Hoover melting point apparatus and were uncorrected.

Experimental Section

Preparation and characterization of substrates.

Preparation of Aryl(4-pyridyl)methanes:

Compounds **1b**,[1] **1c**,[2] **1d**[3] , **1e**[4] and **1f**[5] were prepared according to literature procedures.

General procedure: Into a dried sealed-tube was introduced, under argon, benzyl chloride (0.5 mmol, 1.0 equiv), Na_2CO_3 (1.05 mmol, 2.1 equiv), the corresponding boronic acid (0.6 mmol, 1.2 equiv), tetrakis(triphenylphosphane)palladium (0.01 mmol, 10 mol%), 1,2-dimethoxyethane (2 mL) and water (1 mL). The tube was sealed and heated at 100 °C for 4 h. After cooling to room temperature, the reaction mixture was opened to air, and filtered over a pad of $MgSO_4$ and silica. The pad was rinsed with ethyl acetate (3 × 5 mL), and the combined solutions were concentrated *in vacuo*. The crude material was loaded onto a deactivated silica gel column and purified by flash chromatography to afford desired products.[6]

Preparation of Diaryl(4-pyridyl)methanes:

Compounds **5a**[7], **5b**, **5c**, **5d** and **8c**[8] were prepared according to literature procedures.

General Procedure: An oven-dried 8 mL reaction vial equipped with a stir bar was charged with 4-benzylpyridine or 2-benzylpyridine (143.5 μL, 0.90 mmol, 1.2 equiv) and aryl bromide (0.75 mmol, 1.0 equiv) in a glove box under a nitrogen atmosphere at room temperature. A stock solution containing $Pd(OAc)_2$ (5 mol %) and NiXantphos (7.5 mol %) in 3.75 mL of dry CPME was taken up by syringe and added to the reaction vial under nitrogen. Next, KHMDS (448.8 mg, 2.25 mmol, 3.0 equiv) was added to the reaction mixture. The vial was capped, removed from the glove box, and stirred for 12 h at 110 °C. After cooling to room temperature, the reaction mixture was opened to air, quenched with 0.5 mL of H_2O, diluted with 5 mL of ethyl acetate, and filtered over a pad of $MgSO_4$ and silica. The pad was rinsed with ethyl acetate (3 × 5 mL), and the combined solutions were concentrated *in vacuo*. The crude material was loaded onto a deactivated silica gel column and purified by flash chromatography to afford desired products.[9]

Compounds **8a** and **8b** were prepared according to literature procedures.

General Procedure: An oven-dried 8 mL reaction vial equipped with a stir bar was charged with 2-methylbenzoxazole or 2-methylthiazole (0.5 mmol, 1.0 equiv) and chlorobenzene (1.5 mmol, 3.0 equiv) in a glove box under a nitrogen atmosphere at room temperature. A stock solution containing $Pd(OAc)_2$ (5 mol %) and PCy_3 (10 mol %) in 2.5 mL of dry o-xylene was taken up by syringe and added to the reaction vial under nitrogen. Next, NaO*t*-Bu (1.5 mmol, 3.0 equiv) was added to the reaction mixture. The vial was capped, removed from the glove box, and stirred for 12 h at 130 °C. The reaction mixture was quenched with 0.5 mL of H_2O, diluted with 10 mL of ethyl acetate, and filtered over a pad of $MgSO_4$ and silica. The pad was rinsed with ethyl acetate (10 × 2 mL) and the combined solutions were concentrated in vacuo. The crude material was loaded onto a deactivated silica gel column and purified by flash chromatography to afford the desired products **8a** and **8b** in 81 and 49% yield, respectively.[8]

Characterization of structurally new compounds

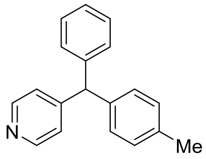

(4-Methylphenyl)(4-pyridyl)phenylmethane (**5c**): The reaction was performed following the literature procedure with 4-benzylpyridine (143.5 μL, 0.90 mmol) and 4-bromotoluene (92.3 μL, 0.75 mmol), KHMDS (448.8 mg, 2.25 mmol) in 3.75 mL of dry CPME at 110 °C. The crude material was purified by flash

chromatography on silica gel (eluted with hexanes:ethyl acetate, 4:1 to 3:1 v/v) to yield the product **5c** (156.7 mg, 81%) as a yellow oil; TLC (hexanes:ethyl acetate, 2:1 v/v): R_f = 0.44. ^{1}H NMR (500 MHz, $CDCl_3$): δ 8.50 (dd, *J* = 4.5, 1.6 Hz, 2H), 7.33 – 7.27 (m, 2H), 7.26 – 7.19 (m, 1H), 7.13 – 7.07 (m, 4H), 7.03 (dd, *J* = 4.5, 1.6 Hz, 2H), 7.00 – 6.96 (m, 2H), 5.45 (s, 1H), 2.33 (s, 3H); ^{13}C NMR (125 MHz, $CDCl_3$): δ 153.05, 149.84, 142.38, 139.20, 136.53, 129.35, 129.26, 128.61, 126.84, 124.66, 55.94, 21.07; IR (thin film): 3025, 1593, 1558, 1511, 1494, 1450, 1413, 1217, 1070, 1030, 994, 700, 605 cm^{-1}; HRMS (m/z): $[M + H]^+$ calcd. for $C_{19}H_{18}N$, 260.1439; found, 260.1448.

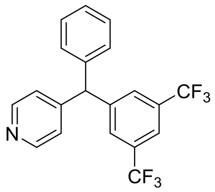

4-((3,5-Bis(trifluoromethyl)phenyl)(phenyl)methyl)pyridine (**5d**): The reaction was performed following the literature procedure with 4-benzylpyridine (143.5 μL, 0.90 mmol) and 3,5-bis(trifluoromethyl)bromobenzene (129.3 μL, 0.75 mmol), NaO*t*-Bu (216.2 mg, 2.25 mmol) in 3.75 mL of dry THF at 65 °C. The crude material was purified by flash chromatography on silica gel (eluted with hexanes:ethyl acetate, 5:1 to 4:1 v/v) to yield the product **5d** (168.6 mg, 59%) as a light yellow oil; TLC (hexanes:ethyl acetate, 2:1 v/v): R_f = 0.65. ^{1}H NMR (500 MHz, $CDCl_3$): δ 8.58 (dd, *J* = 4.5, 1.6 Hz, 2H), 7.80 (s, 1H), 7.57 (s, 2H), 7.39 – 7.35 (m, 2H), 7.33 – 7.29 (m, 1H), 7.10 – 7.06 (m, 2H), 7.02 (dd, *J* = 4.8, 1.4 Hz, 2H), 5.63 (s, 1H); ^{13}C NMR (125 MHz, $CDCl_3$): δ 150.86, 150.45, 144.93, 140.29, 132.13 (q, *J* = 33.2 Hz), 129.44 (q, *J* = 2.6 Hz), 129.25, 127.84, 124.39, 123.28 (q, *J* = 271.4 Hz), 121.27 (m), 55.95; IR (thin film): 2962, 1593, 1503, 1485, 1445, 1412, 1235, 1039, 936, 805, 704, 654 cm^{-1}; HRMS (m/z): $[M + H]^+$ calcd. for $C_{20}H_{14}NF_6$, 382.1030; found ,382.1035.

General Procedure and characterization of Pd-catalyzed DCCP of Aryl(4-pyridyl)methanes and Diaryl(4-pyridyl)methanes (Table 2 and 3).

General Procedure A: An oven-dried 8 mL reaction vial equipped with a stir bar was charged with aryl(4-pyridyl)methanes (**1**, 0.10 mmol, 1.0 equiv) or diaryl(4-pyridyl)methanes (**5**, 0.10 mmol, 1.0 equiv) and aryl chloride (**2**, 2.0 - 4.0 equiv) in a glove box under a nitrogen atmosphere at room temperature. A stock solution containing $Pd(OAc)_2$ (1.1 mg, 0.005 mmol, 5 mol %) and PCy_3 (2.8 mg, 0.010 mmol, 10 mol %) in 0.5 mL of dry THF was taken up by syringe and added to the reaction vial under nitrogen. Next, NaO*t*-Bu (2.0 - 4.0 equiv) was added to the reaction mixture. The vial was capped, removed from the glove box, and stirred for 12 h at 100 °C. After cooling to

room temperature, the reaction mixture was opened to air, quenched with three drops of H_2O, diluted with 3 mL of ethyl acetate, and filtered over a pad of $MgSO_4$ and silica. The pad was rinsed with ethyl acetate (3 × 2 mL), and the combined solutions were concentrated *in vacuo*. The crude material was loaded onto a deactivated silica gel column and purified by flash chromatography to afford desired products.

General Procedure B: An oven-dried 8 mL reaction vial equipped with a stir bar was charged with aryl(4-pyridyl)methanes (**1**, 0.10 mmol, 1.0 equiv) or diaryl(azaaryl)methanes (**5**, 0.10 mmol, or **8**, 0.2 mmol, 1.0 equiv) and aryl chloride (**2**, 2.0 - 4.0 equiv) in a glove box under a nitrogen atmosphere at room temperature. A stock solution containing $Pd(OAc)_2$ (1.1 mg, 0.005 mmol, 5 mol %) and cataCXium A (3.6 mg, 0.01 mmol, 10 mol %) in dry 1,4-dioxane was taken up by syringe and added to the reaction vial under nitrogen. Next, NaO*t*-Bu (2.0 - 4.0 equiv) was added to the reaction mixture. The vial was capped, removed from the glove box, and stirred for 12 h at 100 °C. After cooling to room temperature, the reaction mixture was opened to air, quenched with three drops of H_2O, diluted with 3 mL of ethyl acetate, and filtered over a pad of $MgSO_4$ and silica. The pad was rinsed with ethyl acetate (3 × 2 mL), and the combined solutions were concentrated *in vacuo*. The crude material was loaded onto a deactivated silica gel column and purified by flash chromatography to afford desired products.

Characterization of structurally new compounds

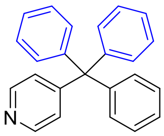

4-Tritylpyridine (**4a**): The reaction was performed following the General Procedure A with 4-benzylpyridine (**1a**, 15.9 μL, 0.10 mmol) and chlorobenzene (**2e**, 40.7 μL, 0.40 mmol, 4.0 equiv) with $Pd(OAc)_2$ (5.0 mol %), PCy_3 (10.0 mol %) and NaO*t*-Bu (38.4 mg, 0.40 mmol, 4.0 equiv) in 0.5 mL of dry THF for 12 h at 100 °C. The crude material was purified by flash chromatography on silica gel (eluted with hexanes:ethyl acetate, 4:1 to 3:1 v/v) to yield the product **4a** (22.1 mg, 69%) as a white solid; mp: 259 – 261 °C; TLC (hexanes:ethyl acetate, 2:1 v/v): R_f = 0.41. ^{1}H NMR (500 MHz, $CDCl_3$): δ 8.49 (dd, *J* = 4.6, 1.6 Hz, 2H), 7.29 – 7.28 (m, 1H), 7.27 – 7.25 (m, 5H), 7.24 – 7.20 (m, 3H), 7.19 – 7.15 (m, 8H); ^{13}C NMR (125 MHz, $CDCl_3$): δ 155.84, 149.42, 145.45, 131.07, 128.93, 126.56, 126.16, 64.86; IR (thin film): 3031, 1586, 1544, 1489, 1441, 1404, 1263, 1073, 813, 753, 703, 634 cm^{-1}; HRMS (m/z): $[M + H]^+$ calcd. for $C_{24}H_{20}N$, 322.1596; found, 322.1605.

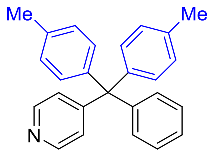

4-(Phenyldi-*p*-tolylmethyl)pyridine (**4b**): The reaction was performed following the General Procedure A with 4-benzylpyridine (**1a**, 39.9 μL, 0.25 mmol) and 4-chlorotoluene (**2d**, 88.7 μL, 0.75 mmol, 3.0 equiv) with $Pd(OAc)_2$ (5.0 mol %), PCy_3 (10.0 mol %) and NaO*t*-Bu (72.1 mg, 0.75 mmol, 3.0 equiv) in 1.25 mL of dry THF for 12 h at 100 °C. The crude material was purified by flash chromatography on silica gel (eluted with hexanes:ethyl acetate, 4:1 to 3:1 v/v) to yield the product **4b** (80.3 mg, 92%) as a light yellow solid; mp: 166 – 168 °C; TLC (hexanes:ethyl acetate, 2:1 v/v): R_f = 0.48. ^{1}H NMR (500 MHz, $CDCl_3$): δ 8.47 (dd, *J* = 4.6, 1.6 Hz, 2H), 7.27 – 7.22 (m, 2H), 7.21 – 7.15 (m, 5H), 7.08 – 7.03 (m, 8H), 2.31 (s, 6H); ^{13}C NMR (125 MHz, $CDCl_3$): δ 156.17, 149.33, 145.77, 142.65, 136.03, 130.98, 130.88, 128.57, 127.82, 126.38, 126.10, 64.17, 21.00; IR (thin film): 3022, 1590, 1547, 1508, 1492, 1445, 1409, 1192, 1119, 1073, 1021, 808, 757, 704, 630 cm^{-1}; HRMS (m/z): $[M + H]^+$ calcd. for $C_{26}H_{24}N$, 350.1909; found ,350.1896.

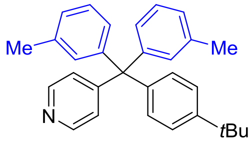

4-((4-(*tert*-Butyl)phenyl)di-*m*-tolylmethyl)pyridine (**4c**): The reaction was performed following the General Procedure A with 4-(4-*tert*-butylbenzyl)pyridine (**1b**, 22.5 mg, 0.10 mmol) and 3-chlorotoluene (**2f**, 47.2 μL, 0.40 mmol, 4.0 equiv) with $Pd(OAc)_2$ (5.0 mol %), PCy_3 (10.0 mol %) and NaO*t*-Bu (38.4 mg, 0.40 mmol, 4.0 equiv) in 0.5 mL of dry THF for 12 h at 100 °C. The crude material was purified by flash chromatography on silica gel (eluted with hexanes:ethyl acetate, 4:1 to 3:1 v/v) to yield the product **4c** (36.9 mg, 91%) as a colorless oil. TLC (hexanes:ethyl acetate, 2:1 v/v): R_f = 0.46. ^{1}H NMR (500 MHz, $CDCl_3$): δ 8.47 (dd, *J* = 4.6, 1.6 Hz, 2H), 7.27 – 7.24 (m, 2H), 7.18 (dd, *J* = 4.6, 1.6 Hz, 2H), 7.14 (t, *J* = 7.6 Hz, 2H), 7.10 – 7.06 (m, 2H), 7.03 – 6.96 (m, 6H), 2.26 (s, 6H), 1.30 (s, 9H); ^{13}C NMR (125 MHz, $CDCl_3$): δ 156.33, 149.24, 149.14, 145.64, 142.41, 137.23, 131.67, 130.68, 128.32, 127.62, 127.13, 126.25, 124.69, 64.37, 34.49, 31.46, 21.83; IR (thin film): 2962, 1590, 1558, 1542, 1507, 1489, 1457, 1417, 1362, 1269, 777, 708, 639 cm^{-1}; HRMS (m/z): $[M + H]^+$ calcd. for $C_{30}H_{32}N$, 406.2535; found, 406.2536.

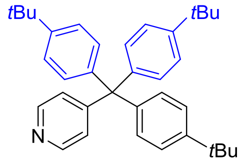

4-(Tris(4-(*tert*-butyl)phenyl)methyl)pyridine (**4d**): The reaction was performed following the General Procedure A with 4-(4-*tert*-butylbenzyl)pyridine (**1b**, 22.5 mg, 0.10 mmol) and 4-*tert*-butyl-chlorobenzene (**2g**, 50.1 μL, 0.30 mmol, 3.0 equiv) with $Pd(OAc)_2$ (5.0 mol %), PCy_3 (10.0 mol %) and NaO*t*-Bu (28.8 mg, 0.30 mmol, 3.0 equiv) in 0.5 mL of dry THF for 12 h at 100 °C. The crude material was purified by flash chromatography on silica gel (eluted with hexanes:ethyl acetate, 5:1 to 4:1 v/v) to yield the product **4d** (48.4 mg, 99%) as a white solid; mp: 254 – 255 °C; TLC (hexanes:ethyl acetate, 2:1 v/v): R_f = 0.60. ^{1}H NMR (500 MHz, $CDCl_3$): δ 8.47 (dd, *J* = 4.6, 1.6 Hz, 2H), 7.29 – 7.22 (m, 6H), 7.15 (dd, *J* = 4.6, 1.6 Hz, 2H), 7.10 – 7.03 (m, 6H), 1.30 (s, 27H); ^{13}C NMR (125 MHz, $CDCl_3$): δ 156.45, 149.18, 149.11, 142.58, 130.72, 126.31, 124.56, 63.66, 34.49, 31.49; IR (thin film): 2962, 2902, 2868, 1590, 1542, 1508, 1473, 1458, 1396, 1362, 1269, 1111, 1017, 824, 810, 748, 706, 652, 637 cm^{-1}; HRMS (m/z): $[M + H]^+$ calcd. for $C_{36}H_{44}N$, 490.3474; found ,490.3487.

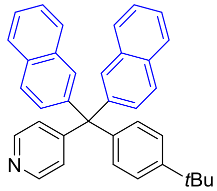

4-((4-(*tert*-Butyl)phenyl)di(naphthalen-2-yl)methyl)pyridine (**4e**): The reaction was performed following the General Procedure A with 4-(4-*tert*-butylbenzyl)pyridine (**1b**, 22.5 mg, 0.10 mmol) and 2-chloronaphthalene (**2h**, 65.1 mg, 0.40 mmol, 4.0 equiv) with $Pd(OAc)_2$ (5.0 mol %), PCy_3 (10.0 mol %) and NaO*t*-Bu (38.4 mg, 0.40 mmol, 4.0 equiv) in 0.5 mL of dry THF for 12 h at 100 °C. The crude material was purified by flash chromatography on silica gel (eluted with hexanes:ethyl acetate, 4:1 to 3:1 v/v) to yield the product **4e** (44.9 mg, 94%) as a white foam. TLC (hexanes:ethyl acetate, 2:1 v/v): R_f = 0.43. ^{1}H NMR (500 MHz, $CDCl_3$): δ 8.53 (dd, *J* = 4.6, 1.6 Hz, 2H), 7.80 (d, *J* = 7.8 Hz, 2H), 7.74 (d, *J* = 1.7 Hz, 2H), 7.71 (d, *J* = 8.8 Hz, 4H), 7.50 – 7.42 (m, 4H), 7.31 – 7.25 (m, 5H), 7.24 (d, *J* = 1.9 Hz, 1H), 7.19 – 7.15 (m, 2H), 1.32 (s, 9H); ^{13}C NMR (125 MHz, $CDCl_3$): δ 155.57, 149.54, 149.51, 142.88, 141.67, 133.05, 132.00, 130.84, 130.39, 128.57, 128.50, 127.48, 127.20, 126.42, 126.32, 126.24, 124.95, 64.56, 34.53, 31.45; IR (thin film): 3055, 2961, 1590, 1542, 1506, 1473, 1457, 1408, 1362, 1271, 1120, 1073, 1017, 857, 817, 790, 747, 675, 623 cm^{-1}; HRMS (m/z): $[M + H]^+$ calcd. for $C_{36}H_{32}N$, 478.2535; found, 478.2538.

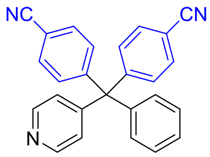

4,4'-(Phenyl(pyridin-4-yl)methylene)dibenzonitrile (**4f**): The reaction was performed following the General Procedure B with 4-benzylpyridine (**1a**, 15.9 µL, 0.10 mmol) and 4-chlorobenzonitrile (**2i**, 55.0 mg, 0.40 mmol, 4.0 equiv) with $Pd(OAc)_2$ (5.0 mol %), cataCXium A (10.0 mol %) and NaO*t*-Bu (38.4 mg, 0.40 mmol, 4.0 equiv) in 0.5 mL of dry 1,4-dioxane for 12 h at 100 °C. The crude material was purified by flash chromatography on silica gel (eluted with hexanes:ethyl acetate, 2:1 to 1:1 v/v) to yield the product **4f** (34.5 mg, 93%) as a light yellow solid; mp: 89 – 92 °C; TLC (hexanes:ethyl acetate, 1:2 v/v): R_f = 0.47. ^{1}H NMR (500 MHz, $CDCl_3$): δ 8.57 (dd, *J* = 4.7, 1.6 Hz, 2H), 7.63 – 7.60 (m, 4H), 7.36 – 7.28 (m, 7H), 7.12 – 7.09 (m, 4H); ^{13}C NMR (125 MHz, $CDCl_3$): δ 153.48, 150.05, 149.60, 143.12, 132.12, 131.44, 130.52, 128.73, 127.60, 125.48, 118.34, 111.23, 65.19; IR (thin film): 3057, 1591, 1500, 1445, 1410, 1269, 1072, 1019, 826, 813, 732, 704, 630 cm^{-1}; HRMS (m/z) : $[M + H]^+$ calcd. for $C_{26}H_{18}N$, 372.1501; found, 372.1499.

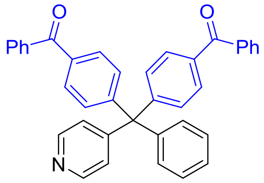

((Phenyl(pyridine-4-yl)methylene)bis(4,1-phenylene))bis(phenylmethanone) (**4g**): The reaction was performed following the General Procedure B with 4-benzylpyridine (**1a**, 15.9 µL, 0.10 mmol) and 4-chlorobenzophenone (**2j**, 86.7 mg, 0.40 mmol, 4.0 equiv) with $Pd(OAc)_2$ (5.0 mol %), cataCXium A (10.0 mol %) and NaO*t*-Bu (38.4 mg, 0.40 mmol, 4.0 equiv) in 0.5 mL of dry 1,4-dioxane for 12 h at 100 °C. The crude material was purified by flash chromatography on silica gel (eluted with hexanes:ethyl acetate, 2:1 to 1:1 v/v) to yield the product **4g** (44.4 mg, 84%) as a light yellow solid; mp: 238 – 241 °C; TLC (hexanes:ethyl acetate, 1:2 v/v): R_f = 0.57. ^{1}H NMR (500 MHz, $CDCl_3$): δ 8.56 (dd, *J* = 4.7, 1.6 Hz, 2H), 7.84 – 7.79 (m, 4H), 7.78 – 7.74 (m, 4H), 7.61 – 7.57 (m, 2H), 7.51 – 7.45 (m, 4H), 7.39 – 7.35 (m, 4H), 7.33 – 7.27 (m, 3H), 7.24 – 7.21 (m, 4H); ^{13}C NMR (125 MHz, $CDCl_3$): δ 196.04, 154.48, 149.79, 149.42, 144.18, 137.40, 135.96, 132.62, 130.79, 130.05, 129.89, 128.41, 128.36, 127.09, 125.78, 65.12; IR (thin film): 3056, 1656, 1597, 1490, 1446, 1408, 1316, 1279, 939, 841, 814, 793, 702, 649 cm^{-1}; HRMS (m/z): $[M + H]^+$ calcd. for $C_{38}H_{28}NO_2$,530.2120; found, 530.2114.

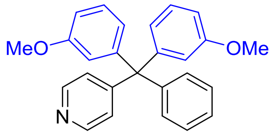

4-(Bis(3-methoxyphenyl)(phenyl)methyl)pyridine (**4h**): The reaction was performed following the General Procedure A with 4-benzylpyridine (**1a**, 15.9 μL, 0.10 mmol) and 3-chloroanisole (**2k**, 36.7 μL, 0.30 mmol, 3.0 equiv) with $Pd(OAc)_2$ (5.0 mol %), PCy_3 (10.0 mol %) and NaO*t*-Bu (28.8 mg, 0.30 mmol, 3.0 equiv) in 0.5 mL of dry THF for 12 h at 100 °C. The crude material was purified by flash chromatography on silica gel (eluted with hexanes:ethyl acetate, 3:1 to 2:1 v/v) to yield the product **4h** (36.6 mg, 96%) as a light yellow oil. TLC (hexanes:ethyl acetate, 2:1 v/v): R_f = 0.32. ^{1}H NMR (500 MHz, $CDCl_3$): δ 8.49 (dd, *J* = 4.6, 1.6 Hz, 2H), 7.28 – 7.23 (m, 2H), 7.22 – 7.16 (m, 7H), 6.79 – 6.73 (m, 6H), 3.69 (s, 6H); ^{13}C NMR (125 MHz, $CDCl_3$): δ 159.17, 155.58, 149.38, 146.92, 145.16, 131.00, 128.71, 127.88, 126.59, 126.10, 123.89, 117.76, 111.07, 64.84, 55.26; IR (thin film): 3027, 2938, 2833, 1591, 1488, 1430, 1317, 1292, 1251, 1053, 996, 882, 778, 741, 704, 639 cm^{-1}; HRMS (m/z): $[M + H]^+$ calcd. for $C_{26}H_{24}NO_2$, 382.1807; found, 382.1799.

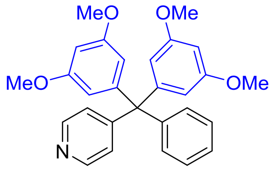

4-(Bis(3,5-dimethoxyphenyl)(phenyl)methyl)pyridine (**4i**): The reaction was performed following the General Procedure B with 4-benzylpyridine (**1a**, 15.9 μL, 0.10 mmol) and 5-chloro-1,3-dimethoxybenzene (**2l**, 69.0 mg, 0.40 mmol, 4.0 equiv) with $Pd(OAc)_2$ (5.0 mol %), cataCXium A (10.0 mol %) and NaO*t*-Bu (38.4 mg, 0.40 mmol, 4.0 equiv) in 0.5 mL of dry 1,4-dioxane for 12 h at 100 °C. The crude material was purified by flash chromatography on silica gel (eluted with hexanes:ethyl acetate, 3:1 to 2:1 v/v) to yield the product **4i** (40.1 mg, 91%) as a light yellow oil. TLC (hexanes:ethyl acetate, 1:1 v/v): R_f = 0.42. ^{1}H NMR (500 MHz, $CDCl_3$): δ 8.49 (dd, *J* = 4.8, 1.5 Hz, 2H), 7.31 – 7.15 (m, 8H), 6.36 (d, *J* = 2.2 Hz, 4H), 6.32 (t, *J* = 2.2 Hz, 2H), 3.66 (s, 12H); ^{13}C NMR (125 MHz, $CDCl_3$): δ 160.12, 155.45, 149.22, 147.46, 144.86, 130.94, 127.82, 126.60, 126.07, 110.17, 97.91, 65.13, 55.34; IR (thin film): 3056, 2936, 2835, 1592, 1456, 1422, 1330, 1304, 1285, 1204, 1158, 1070, 939, 834, 718, 704, 653 cm^{-1}; HRMS (m/z): $[M + H]^+$ calcd. for $C_{28}H_{28}NO_4$, 442.2018; found, 442.1997.

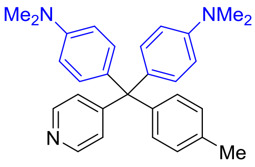 **4,4'-(Pyridin-4-yl(*p*-tolyl)methylene)bis(*N*,*N*-dimethylaniline)** (**4j**): The reaction was performed following the General Procedure B with 4-(4-methylbenzyl)pyridine (**1c**, 18.3 mg, 0.10 mmol) and 4-chloro-*N*,*N*-dimethylaniline (**2m**, 62.3 mg, 0.40 mmol, 4.0 equiv) with $Pd(OAc)_2$ (5.0 mol %), cataCXium A (10.0 mol %) and NaO*t*-Bu (38.4 mg, 0.40 mmol, 4.0 equiv) in 0.5 mL of dry 1,4-dioxane for 12 h at 100 °C. The crude material was purified by flash chromatography on silica gel (eluted with hexanes:ethyl acetate, 3:1 to 2:1 v/v) to yield the product **4j** (35.8 mg, 85%) as a light green solid; mp: 212 – 215 °C; TLC (hexanes:ethyl acetate, 1:1 v/v): R_f = 0.44. ^{1}H NMR (500 MHz, $CDCl_3$): δ 8.45 (dd, *J* = 4.7, 1.5 Hz, 2H), 7.20 (dd, *J* = 4.7, 1.6 Hz, 2H), 7.08 – 7.03 (m, 4H), 7.02 – 6.98 (m, 4H), 6.63 – 6.58 (m, 4H), 2.91 (s, 12H), 2.31 (s, 3H); ^{13}C NMR (125 MHz, $CDCl_3$): δ 157.88, 148.60, 148.55, 143.55, 135.57, 133.85, 131.64, 130.80, 128.36, 126.24, 111.57, 62.86, 40.52, 20.99; IR (thin film): 2884, 1609, 1591, 1517, 1444, 1409, 1352, 1209, 1162, 1129, 949, 810, 733, 655 cm^{-1}; HRMS (m/z): $[M + H]^+$ calcd. for $C_{29}H_{32}N_3$, 422.2596; found, 422.2583.

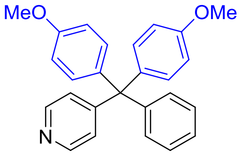 **4-(Bis(4-methoxyphenyl)(phenyl)methyl)pyridine** (**4k**): The reaction was performed following the General Procedure A with 4-benzylpyridine (**1a**, 15.9 μL, 0.10 mmol) and 4-chloromethoxybenzene (**2n**, 36.7 μL, 0.30 mmol, 3.0 equiv) with $Pd(OAc)_2$ (5.0 mol %), PCy_3 (10.0 mol %) and NaO*t*-Bu (28.8 mg, 0.30 mmol, 3.0 equiv) in 0.5 mL of dry THF for 12 h at 100 °C. The crude material was purified by flash chromatography on silica gel (eluted with hexanes:ethyl acetate, 3:1 to 2:1 v/v) to yield the product **4k** (31.6 mg, 83%) as a light yellow oil. TLC (hexanes:ethyl acetate, 1:1 v/v): R_f = 0.62. ^{1}H NMR (500 MHz, $CDCl_3$): δ 8.48 (dd, *J* = 4.7, 1.6 Hz, 2H), 7.28 – 7.23 (m, 2H), 7.22 – 7.19 (m, 1H), 7.17 – 7.14 (m, 4H), 7.08 – 7.04 (m, 4H), 6.82 – 6.77 (m, 4H), 3.78 (s, 6H); ^{13}C NMR (125 MHz, $CDCl_3$): δ 158.02, 156.52, 149.29, 145.98, 137.85, 132.09, 130.97, 127.90, 126.50, 126.14, 113.21, 63.55, 55.37; IR (thin film): 2953, 2835, 1606, 1590, 1508, 1463, 1412, 1298, 1252, 1183, 1035, 825, 814, 704, 670, 642, 627 cm^{-1}; HRMS (m/z): $[M + H]^+$ calcd. for $C_{26}H_{24}NO_2$, 382.1807; found, 382.1805.

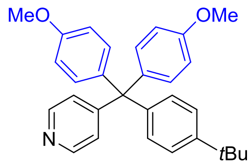

4-((4-(*tert*-Butyl)phenyl)bis(4-methoxyphenyl)methyl)pyridine (**4l**): The reaction was performed following the General Procedure A with 4-(4-*tert*-butylbenzyl)pyridine (**1b**, 22.5 mg, 0.10 mmol) and 4-chloromethoxybenzene (**2n**, 36.7 µL, 0.30 mmol, 3.0 equiv) with $Pd(OAc)_2$ (5.0 mol %), PCy_3 (10.0 mol %) and NaO*t*-Bu (28.8 mg, 0.30 mmol, 3.0 equiv) in 0.5 mL of dry THF for 12 h at 100 °C. The crude material was purified by flash chromatography on silica gel (eluted with hexanes:ethyl acetate, 3:1 to 2:1 v/v) to yield the product **4l** (35.9 mg, 82%) as a pale yellow solid; mp: 167 – 169 °C; TLC (hexanes:ethyl acetate, 2:1 v/v): R_f = 0.32. ^{1}H NMR (500 MHz, $CDCl_3$): δ 8.47 (dd, *J* = 4.6, 1.6 Hz, 2H), 7.28 – 7.23 (m, 2H), 7.15 (dd, *J* = 4.6, 1.6 Hz, 2H), 7.09 – 7.02 (m, 6H), 6.82 – 6.76 (m, 4H), 3.79 (s, 6H), 1.30 (s, 9H); ^{13}C NMR (125 MHz, $CDCl_3$): δ 157.92, 156.60, 149.27, 149.20, 142.73, 138.05, 132.06, 130.54, 126.10, 124.68, 113.07, 63.10, 55.33, 34.49, 31.46; IR (thin film): 2960, 2835, 1606, 1591, 1507, 1463, 1411, 1299, 1252, 1183, 1036, 825, 668, 655 cm^{-1}; HRMS (m/z): $[M + H]^+$ calcd. for $C_{30}H_{32}NO_2$, 438.2433; found, 438.2431.

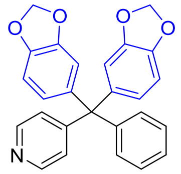

4-(Bis(benzo[*d*][1,3]dioxol-5-yl)(phenyl)methyl)pyridine (**4m**): The reaction was performed following the General Procedure B with 4-benzylpyridine (**1a**, 15.9 µL, 0.10 mmol) and 5-chloro-1,3-benzodioxole (**2o**, 46.7 µL, 0.40 mmol, 4.0 equiv) with $Pd(OAc)_2$ (5.0 mol %), cataCXium A (10.0 mol %) and NaO*t*-Bu (38.4 mg, 0.40 mmol, 4.0 equiv) in 1.0 mL of dry 1,4-dioxane for 12 h at 100 °C. The crude material was purified by flash chromatography on silica gel (eluted with hexanes:ethyl acetate, 3:1 to 2:1 v/v) to yield the product **4m** (38.4 mg, 94%) as a brown oil. TLC (hexanes:ethyl acetate, 1:1 v/v): R_f = 0.39. ^{1}H NMR (500 MHz, $CDCl_3$): δ 8.49 (dd, *J* = 4.6, 1.6 Hz, 2H), 7.31 – 7.24 (m, 2H), 7.23 – 7.15 (m, 5H), 6.73 – 6.59 (m, 6H), 5.92 (s, 4H); ^{13}C NMR (125 MHz, $CDCl_3$): δ 155.99, 149.30, 147.34, 146.11, 145.45, 139.44, 130.85, 127.92, 126.59, 125.98, 124.08, 111.87, 107.39, 101.26, 64.34; IR (thin film): 2893, 1591, 1501, 1484, 1434, 1235, 1038, 934, 801, 733, 705, 660 cm^{-1}; HRMS (m/z): $[M + H]^+$ calcd. for $C_{26}H_{20}NO_4$, 410.1392; found ,410.1398.

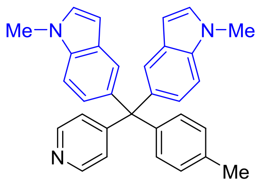 **5,5'-(Pyridin-4-yl(*p*-tolyl)methylene)bis(1-methyl-1*H*-indole)** (**4n**): The reaction was performed following the General Procedure B with 4-(4-methylbenzyl)pyridine (**1c**, 18.3 mg, 0.10 mmol) and 5-chloro-1-methyl-1*H*-indole (**2p**, 66.2 mg, 0.40 mmol, 4.0 equiv) with $Pd(OAc)_2$ (5.0 mol %), cataCXium A (10.0 mol %) and NaO*t*-Bu (38.4 mg, 0.40 mmol, 4.0 equiv) in 1.0 mL of dry 1,4-dioxane for 12 h at 100 °C. The crude material was purified by flash chromatography on silica gel (eluted with hexanes:ethyl acetate, 3:1 to 2:1 v/v) to yield the product **4n** (26.9 mg, 61%) as a yellow oil. TLC (hexanes:ethyl acetate, 1:1 v/v): R_f = 0.34. ^{1}H NMR (500 MHz, $CDCl_3$): δ 8.45 (d, *J* = 6.1 Hz, 2H), 7.50 (d, *J* = 1.7 Hz, 2H), 7.31 (dd, *J* = 4.8, 1.5 Hz, 2H), 7.19 – 7.12 (m, 4H), 7.05 (d, *J* = 8.1 Hz, 2H), 7.02 – 6.95 (m, 4H), 6.37 (d, *J* = 3.0 Hz, 2H), 3.74 (s, 6H), 2.32 (s, 3H); ^{13}C NMR (125 MHz, $CDCl_3$): δ 158.61, 148.21, 143.81, 137.33, 135.63, 135.00, 131.18, 129.03, 128.38, 127.70, 126.71, 126.46, 122.51, 108.51, 101.60, 64.52, 32.97, 21.03; IR (thin film): 2919, 1591, 1509, 1490, 1420, 1338, 1249, 807, 729, 668 cm^{-1}; HRMS (m/z): $[M + H]^+$ calcd. for $C_{31}H_{28}N_3$, 442.2283; found ,442.2284.

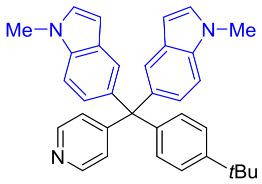 **5,5'-((4-(*tert*-Butyl)phenyl)(Pyridin-4-yl)methylene)bis(1-methyl-1*H*-indole)** (**4o**): The reaction was performed following the General Procedure B with 4-(4-*tert*-butylbenzyl)pyridine (**1b**, 22.5 mg, 0.10 mmol) and 5-chloro-1-methyl-1*H*-indole (**2p**, 66.2 mg, 0.40 mmol, 4.0 equiv) with $Pd(OAc)_2$ (5.0 mol %), cataCXium A (10.0 mol %) and NaO*t*-Bu (38.4 mg, 0.40 mmol, 4.0 equiv) in 1.0 mL of dry 1,4-dioxane for 12 h at 100 °C. The crude material was purified by flash chromatography on silica gel (eluted with hexanes:ethyl acetate, 3:1 to 2:1 v/v) to yield the product **4o** (27.5 mg, 57%) as a yellow oil. TLC (hexanes:ethyl acetate, 1:1 v/v): R_f = 0.38. ^{1}H NMR (500 MHz, $CDCl_3$): δ 8.46 (d, *J* = 6.0 Hz, 2H), 7.50 (d, *J* = 1.8 Hz, 2H), 7.34 (d, *J* = 6.3 Hz, 2H), 7.27 – 7.22 (m, 2H), 7.21 – 7.14 (m, 4H), 7.05 – 6.95 (m, 4H), 6.38 (d, *J* = 3.1 Hz, 2H), 3.76 (s, 6H), 1.30 (s, 9H); ^{13}C NMR (125 MHz, $CDCl_3$): δ 159.09, 148.83, 147.87, 143.50, 137.34, 135.04, 130.87, 129.05, 127.73, 126.84, 126.47, 124.56, 122.58, 108.51, 101.62, 64.53, 34.47, 33.00, 31.49; IR (thin film): 2959, 1591, 1509, 1489, 1421, 1362, 1337, 1291, 1249, 1163, 1073, 1017, 800, 724, 667 cm^{-1}; HRMS (m/z): $[M + H]^+$ calcd. for $C_{34}H_{34}N_3$, 484.2753; found, 484.2748.

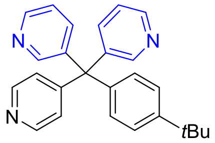

3,3'-((4-(*tert*-Butyl)phenyl)(pyridin-4-yl)methylene)dipyridine (**4p**): The reaction was performed following the General Procedure B with 4-(4-*tert*-butylbenzyl)pyridine (**1b**, 22.5 mg, 0.10 mmol) and 3-chloropyridine (**2q**, 38.0 μL, 0.40 mmol, 4.0 equiv) with $Pd(OAc)_2$ (5.0 mol %), cataCXium A (10.0 mol %) and NaO*t*-Bu (38.4 mg, 0.40 mmol, 4.0 equiv) in 1.0 mL of dry 1,4-dioxane for 12 h at 100 °C. The crude material was purified by flash chromatography on silica gel (eluted with dichloromethane:methanol, 20:1 to 10:1 v/v) to yield the product **4p** (31.9 mg, 84%) as a pale yellow solid; mp: 180 – 182 °C; TLC (dichloromethane:methanol, 12:1 v/v): R_f = 0.21. ^{1}H NMR (500 MHz, $CDCl_3$): δ 8.56 (dd, *J* = 4.7, 1.7 Hz, 2H), 8.51 (dd, *J* = 4.7, 1.5 Hz, 2H), 8.46 (d, *J* = 2.4 Hz, 2H), 7.51 (ddd, *J* = 8.1, 2.3, 1.7 Hz, 2H), 7.36 – 7.30 (m, 2H), 7.26 (dd, *J* = 8.1, 4.7 Hz, 2H), 7.13 (dd, *J* = 4.7, 1.6 Hz, 2H), 7.05 – 7.00 (m, 2H), 1.31 (s, 9H); ^{13}C NMR (125 MHz, $CDCl_3$): δ 153.84, 152.01, 150.34, 149.92, 147.99, 140.30, 140.05, 137.82, 130.16, 125.51, 125.45, 122.92, 61.14, 34.55, 31.33; IR (thin film): 3032, 2962, 1592, 1507, 1474, 1415, 1362, 1270, 1023, 804, 716, 668, 646 cm^{-1}; HRMS (m/z): $[M + H]^+$ calcd. for $C_{26}H_{26}N_3$, 380.2127; found, 380.2109.

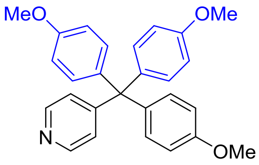

4-(Tris(4-methoxyphenyl)methyl)pyridine (**4q**): The reaction was performed following the General Procedure B with 4-(4-methoxybenzyl)pyridine (**1d**, 19.9 mg, 0.10 mmol) and 4-chloromethoxybenzene (**2n**, 49.0 μL, 0.40 mmol, 4.0 equiv) with $Pd(OAc)_2$ (5.0 mol %), cataCXium A (10.0 mol %) and NaO*t*-Bu (38,4 mg, 0.40 mmol, 4.0 equiv) in 0.5 mL of dry 1,4-dioxane for 12 h at 100 °C. The crude material was purified by flash chromatography on silica gel (eluted with hexanes:ethyl acetate, 3:1 to 2:1 v/v) to yield the product **4q** (40.1 mg, 91%) as a yellow foam. TLC (hexanes:ethyl acetate, 1:1 v/v): R_f = 0.39. ^{1}H NMR (500 MHz, $CDCl_3$): δ 8.47 (dd, *J* = 4.8, 1.5 Hz, 2H), 7.14 (dd, *J* = 4.7, 1.6 Hz, 2H), 7.10 – 7.01 (m, 6H), 6.85 – 6.73 (m, 6H), 3.78 (s, 9H); ^{13}C NMR (125 MHz, $CDCl_3$): δ 157.91, 156.64, 149.24, 138.04, 131.95, 125.99, 113.08, 62.78, 55.29; IR (thin film): 2952, 2835, 1606, 1591, 1507, 1459, 1412, 1298, 1251, 1182, 1034, 825, 735, 659, 634 cm^{-1}; HRMS (m/z) : $[M + H]^+$ calcd. for $C_{27}H_{26}NO_3$, 412.1913; found, 412.1911.

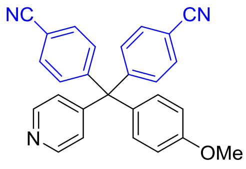

4,4'-((4-Methoxyphenyl)(pyridin-4-yl)methylene)dibenzonitrile (**4r**): The reaction was performed following the General Procedure B with 4-(4-methoxybenzyl)pyridine (**1d**, 19.9 mg, 0.10 mmol) and 4-chlorobenzonitrile (**2i**, 55.0 mg, 0.40 mmol, 4.0 equiv) with $Pd(OAc)_2$ (5.0 mol %), cataCXium A (10.0 mol %) and NaO*t*-Bu (38.4 mg, 0.40 mmol, 4.0 equiv) in 0.5 mL of dry 1,4-dioxane for 12 h at 100 °C. The crude material was purified by flash chromatography on silica gel (eluted with hexanes:ethyl acetate, 2:1 to 1:1 v/v) to yield the product **4r** (34.5 mg, 86%) as a yellow oil. TLC (hexanes:ethyl acetate, 1:2 v/v): R_f = 0.47. ^{1}H NMR (500 MHz, $CDCl_3$): δ 8.56 (dd, *J* = 4.6, 1.7 Hz, 2H), 7.67 – 7.57 (m, 4H), 7.33 – 7.29 (m, 4H), 7.09 (dd, *J* = 4.6, 1.7 Hz, 2H), 7.03 – 6.93 (m, 2H), 6.89 – 6.77 (m, 2H), 3.80 (s, 3H); ^{13}C NMR (125 MHz, $CDCl_3$): δ 158.68, 153.70, 150.05, 149.90, 135.07, 132.08, 131.68, 131.38, 125.41, 118.38, 113.99, 111.14, 64.53, 55.42; IR (thin film): 3030, 2228, 1603, 1591, 1508, 1457, 1409, 1299, 1254, 1185, 1072, 1032, 826, 734, 656, 641 cm^{-1}; HRMS (m/z) : $[M + H]^+$ calcd. for $C_{27}H_{20}N_3O$, 402.1606; found, 402.1602.

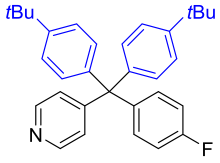

4-(Bis(4-(*tert*-butyl)phenyl)(4-fluorophenyl)methyl)pyridine (**4s**): The reaction was performed following the General Procedure A with 4-(4-fluorolbenzyl)pyridine (**1e**, 18.7 mg, 0.10 mmol) and 4-*tert*-butyl-chlorobenzene (**2g**, 50.1 μL, 0.30 mmol, 3.0 equiv) with $Pd(OAc)_2$ (5.0 mol %), PCy_3 (10.0 mol %) and NaO*t*-Bu (28.8 mg, 0.30 mmol, 3.0 equiv) in 0.5 mL of dry THF for 12 h at 100 °C. The crude material was purified by flash chromatography on silica gel (eluted with hexanes:ethyl acetate, 5:1 to 4:1 v/v) to yield the product **4s** (42.4 mg, 94%) as a white solid; m.p.= 193 – 195 °C. TLC (hexanes:ethyl acetate, 2:1 v/v): R_f = 0.54. ^{1}H NMR (500 MHz, $CDCl_3$): δ 8.49 (dd, *J* = 4.6, 1.6 Hz, 2H), 7.28 – 7.25 (m, 4H), 7.15 – 7.12 (m, 4H), 7.06 – 7.02 (m, 4H), 6.97 – 6.92 (m, 2H), 1.30 (s, 18H); ^{13}C NMR (125 MHz, $CDCl_3$): δ 161.23 (d, *J* = 245.1Hz), 156.05, 149.36, 149.34, 142.24, 141.61 (d, *J* = 3.3Hz), 132.63 (d, *J* = 7.7Hz), 130.56, 126.07, 124.75, 114.52 (d, *J* = 21.1Hz), 63.50, 34.48, 31.44; IR (thin film): 2962, 1591, 1542, 1507, 1473, 1457, 1396, 1362, 1269, 1229, 1164, 1016, 825, 749, 668, 653 cm^{-1}; HRMS (m/z) : $[M + H]^+$ calcd. for $C_{32}H_{35}NF$, 452.2754; found, 452.2752.

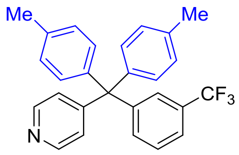

4-(Di-*p*-tolyl(3-(trifluoromethyl)phenyl)methyl)pyridine (**4t**): The reaction was performed following the General Procedure A with 4-(3-(trifluoromethyl)benzyl)pyridine (**1f**, 23.7 mg, 0.10 mmol) and 4-chlorotoluene (**2d**, 47.3 μL, 0.40 mmol, 3.0 equiv) with $Pd(OAc)_2$ (5.0 mol %), PCy_3 (10.0 mol %) and NaO*t*-Bu (38.4 mg, 0.40 mmol, 4.0 equiv) in 0.5 mL of dry THF for 12 h at 100 °C. The crude material was purified by flash chromatography on silica gel (eluted with hexanes:ethyl acetate, 4:1 to 3:1 v/v) to yield the product **4t** (36.3 mg, 87%) as a colorless oil. TLC (hexanes:ethyl acetate, 2:1 v/v): R_f = 0.46. ^{1}H NMR (500 MHz, $CDCl_3$): δ 8.50 (dd, *J* = 4.6, 1.6 Hz, 2H), 7.51 – 7.46 (m, 2H), 7.39 – 7.36 (m, 2H), 7.14 (dd, *J* = 4.6, 1.6 Hz, 2H), 7.09 (d, *J* = 8.2 Hz, 4H), 7.04 – 7.00 (m, 4H), 2.32 (s, 6H); ^{13}C NMR (125 MHz, $CDCl_3$): δ 155.46, 149.58, 146.96, 141.90, 136.49, 134.59, 130.69, 130.28 (q, *J* = 31.8 Hz), 128.87, 128.30, 127.17 (q, *J* = 3.5 Hz), 125.86, 124.19 (q, *J* = 270.9 Hz), 123.43 (q, *J* = 3.5 Hz), 64.16, 20.99; IR (thin film): 3024, 2924, 1590, 1543, 1508, 1489, 1457, 1410, 1328, 1284, 1166, 1126, 1076, 808, 786, 707, 660, 628 cm^{-1}; HRMS (m/z) : $[M + H]^+$ calcd. for $C_{27}H_{23}NF_3$, 418.1783; found, 418.1779.

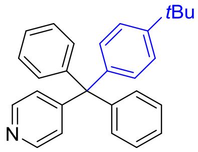

4-((4-(*tert*-Butyl)phenyl)diphenylmethyl)pyridine (**6a**): The reaction was performed following the General Procedure A with 4-benzhydrylpyridine (**5a**, 24.5 mg, 0.10 mmol) and 4-*tert*-butyl-chlorobenzene (**2g**, 33.4 μL, 0.20 mmol, 2.0 equiv) with $Pd(OAc)_2$ (5.0 mol %), PCy_3 (10.0 mol %) and NaO*t*-Bu (19.2 mg, 0.20 mmol, 2.0 equiv) in 0.5 mL of dry THF for 12 h at 100 °C. The crude material was purified by flash chromatography on silica gel (eluted with hexanes:ethyl acetate, 4:1 to 3:1 v/v) to yield the product **6a** (36.6 mg, 97%) as a white solid; m.p.= 206 – 208 °C. TLC (hexanes:ethyl acetate, 2:1 v/v): R_f = 0.48. ^{1}H NMR (500 MHz, $CDCl_3$): δ 8.49 (dd, *J* = 4.7, 1.6 Hz, 2H), 7.29 – 7.24 (m, 6H), 7.23 – 7.20 (m, 2H), 7.19 – 7.15 (m, 6H), 7.10 – 7.05 (m, 2H), 1.30 (s, 9H); ^{13}C NMR (125 MHz, $CDCl_3$): δ 156.06, 149.33, 149.30, 145.64, 142.22, 131.07, 130.66, 127.84, 126.45, 126.19, 124.76, 64.46, 34.49, 31.44; IR (thin film): 3029, 2962, 1590, 1508, 1490, 1445, 1406, 1362, 1269, 830, 811, 762, 703, 632 cm^{-1}; HRMS (m/z) : $[M + H]^+$ calcd. for $C_{28}H_{28}N$, 378.2222; found, 378.2230.

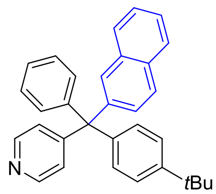

4-((4-(*tert*-Butyl)phenyl)(naphthalen-2-yl)(phenyl)methyl)pyridine (**6b**): The reaction was performed following the General Procedure A with (4-*tert*-butylphenyl)(4-pyridyl)phenylmethane (**5b**, 30.1 mg, 0.10 mmol) and 2-chloronaphthalene (**2h**, 32.6 mg, 0.20 mmol, 2.0 equiv) with $Pd(OAc)_2$ (5.0 mol %), PCy_3 (10.0 mol %) and NaO*t*-Bu (19.2 mg, 0.20 mmol, 2.0 equiv) in 0.5 mL of dry THF for 12 h at 100 °C. The crude material was purified by flash chromatography on silica gel (eluted with hexanes:ethyl acetate, 4:1 to 3:1 v/v) to yield the product **6b** (41.1 mg, 96%) as a yellow solid; m.p.= 197 – 199 °C. TLC (hexanes:ethyl acetate, 2:1 v/v): R_f = 0.44. ^{1}H NMR (500 MHz, $CDCl_3$): δ 8.51 (dd, *J* = 4.7, 1.5 Hz, 2H), 7.82 – 7.75 (m, 1H), 7.73 – 7.65 (m, 3H), 7.48 – 7.41 (m, 2H), 7.29 – 7.27 (m, 3H), 7.25 – 7.18 (m, 7H), 7.15 – 7.10 (m, 2H), 1.31 (s, 9H); ^{13}C NMR (125 MHz, $CDCl_3$): δ 155.85, 149.43, 145.37, 143.17, 141.94, 133.01, 131.95, 131.16, 130.76, 130.39, 128.49, 128.46, 127.94, 127.46, 127.09, 126.57, 126.38, 126.26, 126.21, 124.87, 64.51, 34.53, 31.46; IR (thin film): 3055, 2962, 1590, 1542, 1507, 1408, 1362, 1270, 818, 742, 718, 704, 669, 629 cm^{-1}; HRMS (m/z) : [M + H]$^+$ calcd. for $C_{32}H_{30}N$, 428.2378; found, 428.2379.

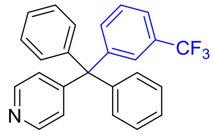

4-(Diphenyl(3-(trifluoromethyl)phenyl)methyl)pyridine (**6c**): The reaction was performed following the General Procedure B with 4-benzhydrylpyridine (**5a**, 24.5 mg, 0.10 mmol) and 3-chlorobenzotrifluoride (**2r**, 27.0 μL, 0.20 mmol, 2.0 equiv) with $Pd(OAc)_2$ (5.0 mol %), cataCXium A (10.0 mol %) and NaO*t*-Bu (19.2 mg, 0.20 mmol, 2.0 equiv) in 0.5 mL of dry 1,4-dioxane for 12 h at 100 °C. The crude material was purified by flash chromatography on silica gel (eluted with hexanes:ethyl acetate, 4:1 to 3:1 v/v) to yield the product **6c** (35.8 mg, 92%) as a yellow solid; m.p.= 117 – 119 °C. TLC (hexanes:ethyl acetate, 2:1 v/v): R_f = 0.32. ^{1}H NMR (500 MHz, $CDCl_3$): δ 8.52 (dd, *J* = 4.6, 1.6 Hz, 2H), 7.55 – 7.48 (m, 2H), 7.43 – 7.36 (m, 2H), 7.32 – 7.26 (m, 4H), 7.25 – 7.21 (m, 2H), 7.19 – 7.11 (m, 6H); ^{13}C NMR (125 MHz, $CDCl_3$): δ 155.08, 149.66, 146.61, 144.68, 134.64, 130.85, 130.39 (q, *J* = 31.9 Hz), 128.41, 128.21, 127.22 (q, *J* = 3.8 Hz), 126.93, 125.90, 124.14 (q, *J* = 270.9 Hz), 123.59 (q, *J* = 3.8 Hz), 64.81; IR (thin film): 3031, 1590, 1550, 1507, 1492, 1446, 1409, 1328, 1167, 1125, 1077, 802, 744, 703, 637 cm^{-1}; HRMS (m/z) : [M + H]$^+$ calcd. for $C_{25}H_{19}NF_3$, 390.1470; found, 390.1478.

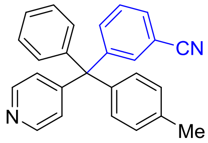

3-(Diphenyl(pyridine-4-yl)(*p*-tolyl)methyl)benzonitrile (**6d**): The reaction was performed following the General Procedure B with (4-methylphenyl)(4-pyridyl)phenylmethane (**5c**, 25.9 mg, 0.10 mmol) and 3-chlorobenzonitrile (**2s**, 27.5 mg, 0.20 mmol, 2.0 equiv) with $Pd(OAc)_2$ (5.0 mol %), cataCXium A (10.0 mol %) and NaO*t*-Bu (19.2 mg, 0.20 mmol, 2.0 equiv) in 0.5 mL of dry 1,4-dioxane for 12 h at 100 °C. The crude material was purified by flash chromatography on silica gel (eluted with hexanes:ethyl acetate, 3:1 to 2:1 v/v) to yield the product **6d** (34.9 mg, 97%) as a yellow oil. TLC (hexanes:ethyl acetate, 1:1 v/v): R_f = 0.64. ^{1}H NMR (500 MHz, $CDCl_3$): δ 8.51 (dd, *J* = 4.7, 1.6 Hz, 2H), 7.54 – 7.50 (m, 2H), 7.47 – 7.42 (m, 1H), 7.41 – 7.35 (m, 1H), 7.32 – 7.27 (m, 2H), 7.26 – 7.22 (m, 1H), 7.15 – 7.08 (m, 6H), 7.02 – 6.98 (m, 2H), 2.33 (s, 3H); ^{13}C NMR (125 MHz, $CDCl_3$): δ 154.81, 149.65, 147.35, 144.37, 141.24, 136.79, 135.60, 134.01, 130.71, 130.61, 130.34, 129.00, 128.70, 128.27, 127.00, 125.75, 118.91, 112.19, 64.29, 20.97; IR (thin film): 3026, 2230, 1590, 1548, 1509, 1491, 1445, 1415, 1319, 1191, 1121, 1073, 796, 739, 699, 636 cm^{-1}; HRMS (m/z) : $[M + H]^+$ calcd. for $C_{26}H_{21}N_2$, 361.1705; found, 361.1711.

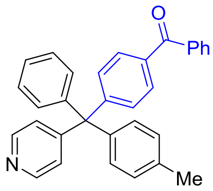

Phenyl(4-(phenyl(pyridine-4-yl)(*p*-tolyl)methyl)phenyl)methanone (**6e**): The reaction was performed following the General Procedure B with (4-methylphenyl)(4-pyridyl)phenylmethane (**5c**, 25.9 mg, 0.10 mmol) and 4-chlorobenzophenone (**2j**, 43.4 mg, 0.20 mmol, 2.0 equiv) with $Pd(OAc)_2$ (5.0 mol %), cataCXium A (10.0 mol %) and NaO*t*-Bu (19.2 mg, 0.20 mmol, 2.0 equiv) in 0.5 mL of dry 1,4-dioxane for 12 h at 100 °C. The crude material was purified by flash chromatography on silica gel (eluted with hexanes:ethyl acetate, 3:1 to 2:1 v/v) to yield the product **6e** (42.1 mg, 96%) as a yellow oil. TLC (hexanes:ethyl acetate, 1:1 v/v): R_f = 0.62. ^{1}H NMR (500 MHz, $CDCl_3$): δ 8.52 (dd, *J* = 4.7, 1.6 Hz, 2H), 7.83 – 7.78 (m, 2H), 7.75 – 7.70 (m, 2H), 7.60 – 7.54 (m, 1H), 7.49 – 7.42 (m, 2H), 7.37 – 7.31 (m, 2H), 7.30 – 7.26 (m, 2H), 7.25 – 6.19 (m, 5H), 7.12 – 7.06 (m, 4H), 2.32 (s, 3H); ^{13}C NMR (125 MHz, $CDCl_3$): δ 196.19, 155.43, 150.34, 149.43, 144.92, 141.80, 137.55, 136.47, 135.61, 132.51, 130.87, 130.77, 130.05, 129.69, 128.82, 128.36, 128.08, 126.74, 125.96, 64.66, 20.98; IR (thin film): 3029, 1656, 1591, 1542, 1508, 1489, 1446, 1408, 1316, 1278, 1193, 1072, 808, 729, 702, 669, 648 cm^{-1}; HRMS (m/z) : $[M + H]^+$ calcd. for $C_{32}H_{26}NO$, 440.2014; found, 440.2017.

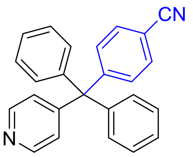

4-(Diphenyl(pyridin-4-yl)methyl)benzonitrile (**6f**): The reaction was performed following the General Procedure B with 4-benzhydrylpyridine (**5a**, 24.5 mg, 0.10 mmol) and 4-chlorobenzonitrile (**2i**, 27.5 mg, 0.20 mmol, 2.0 equiv) with $Pd(OAc)_2$ (5.0 mol %), cataCXium A (10.0 mol %) and NaO*t*-Bu (19.2 mg, 0.20 mmol, 2.0 equiv) in 0.5 mL of dry 1,4-dioxane for 12 h at 100 °C. The crude material was purified by flash chromatography on silica gel (eluted with hexanes:ethyl acetate, 3:1 to 2:1 v/v) to yield the product **6f** (31.5 mg, 91%) as a light yellow solid; m.p.= 209 – 211 °C. TLC (hexanes:ethyl acetate, 1:1 v/v): R_f = 0.36. ^{1}H NMR (500 MHz, $CDCl_3$): δ 8.53 (dd, *J* = 4.6, 1.6 Hz, 2H), 7.60 – 7.53 (m, 2H), 7.37 – 7.33 (m, 2H), 7.32 – 7.28 (m, 4H), 7.27 – 7.22 (m, 2H), 7.17 – 7.11 (m, 6H); ^{13}C NMR (125 MHz, $CDCl_3$): δ 154.64, 150.92, 149.72, 144.27, 131.78, 131.67, 130.80, 128.32, 127.08, 125.81, 118.68, 110.61, 65.05; IR (thin film): 3056, 1590, 1491, 1445, 1409, 1073, 1034, 834, 812, 732, 703, 631 cm^{-1}; HRMS (m/z) : $[M + H]^+$ calcd. for $C_{25}H_{19}N_2$, 347.1548; found, 347.1545.

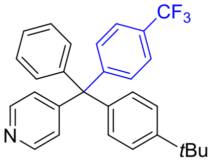

4-((4-(*tert*-Butyl)phenyl)(phenyl)(4-(trifluoromethyl)phenyl)methyl)pyridine (**6g**): The reaction was performed following the General Procedure A with (4-*tert*-butylphenyl)(4-pyridyl)phenylmethane (**5b**, 30.1 mg, 0.10 mmol) and 4-chlorobenzotrifluoride (**2t**, 26.7 µL, 0.20 mmol, 2.0 equiv) with $Pd(OAc)_2$ (5.0 mol %), PCy_3 (10.0 mol %) and NaO*t*-Bu (19.2 mg, 0.20 mmol, 2.0 equiv) in 0.5 mL of dry THF for 12 h at 100 °C. The crude material was purified by flash chromatography on silica gel (eluted with hexanes:ethyl acetate, 5:1 to 4:1 v/v) to yield the product **6g** (39.6 mg, 89%) as a colorless oil. TLC (hexanes:ethyl acetate, 2:1 v/v): R_f = 0.52. ^{1}H NMR (500 MHz, $CDCl_3$): δ 8.51 (dd, *J* = 4.7, 1.6 Hz, 2H), 7.53 (d, *J* = 8.4 Hz, 2H), 7.33 (d, *J* = 8.3 Hz, 2H), 7.31 – 7.27 (m, 4H), 7.25 – 7.22 (m, 1H), 7.19 – 7.14 (m, 4H), 7.08 – 7.04 (m, 2H), 1.31 (s, 9H); ^{13}C NMR (125 MHz, $CDCl_3$): δ 155.28, 149.74, 149.57, 144.89, 141.48, 131.32, 130.91, 130.53, 128.70 (q, *J* = 32.4 Hz), 128.11, 126.81, 125.96, 125.04, 124.83 (q, *J* = 3.9 Hz), 124.22 (q, *J* = 270.4 Hz), 64.47, 34.53, 31.41; IR (thin film): 3031, 2964, 1590, 1542, 1507, 1490, 1445, 1410, 1328, 1167, 1125, 1070, 1017, 827, 811, 761, 740, 704, 632 cm^{-1}; HRMS (m/z) : $[M + H]^+$ calcd. for $C_{29}H_{27}NF_3$, 446.2096; found, 446.2100.

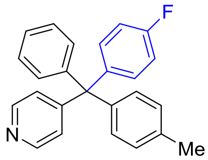

4-((4-Fluorophenyl)(phenyl)(*p*-tolyl)methyl)pyridine (**6h**): The reaction was performed following the General Procedure B with (4-methylphenyl)(4-pyridyl)phenylmethane (**5c**, 25.9 mg, 0.10 mmol) and 1-chloro-4-fluorobenzene (**2u**, 21.3 μL, 0.20 mmol, 2.0 equiv) with $Pd(OAc)_2$ (5.0 mol %), cataCXium A (10.0 mol %) and NaO*t*-Bu (19.2 mg, 0.20 mmol, 2.0 equiv) in 0.5 mL of dry 1,4-dioxane for 12 h at 100 °C. The crude material was purified by flash chromatography on silica gel (eluted with hexanes:ethyl acetate, 4:1 to 3:1 v/v) to yield the product **6h** (32.5 mg, 97%) as a light yellow solid; m.p.= 148 – 151 °C. TLC (hexanes:ethyl acetate, 2:1 v/v): R_f = 0.38. ^{1}H NMR (500 MHz, $CDCl_3$): δ 8.49 (dd, *J* = 4.6, 1.6 Hz, 2H), 7.29 – 7.19 (m, 3H), 7.17 – 7.12 (m, 6H), 7.09 – 7.01 (m, 4H), 6.97 – 6.91 (m, 2H), 2.32 (s, 3H); ^{13}C NMR (125 MHz, $CDCl_3$): δ 161.26 (d, *J* = 245.4Hz), 155.83, 149.44, 145.43, 142.31, 141.41 (d, *J* = 3.4Hz), 136.32, 132.58 (d, *J* = 7.8Hz), 130.88, 130.79, 128.71, 127.96, 126.62, 125.98, 114.68 (d, *J* = 21.0Hz), 63.96, 20.99; IR (thin film): 3025, 1590, 1542, 1506, 1457, 1409, 1230, 1164, 1016, 835, 818, 704, 628 cm^{-1}; HRMS (m/z) : $[M + H]^+$ calcd. for $C_{25}H_{21}NF$, 354.1658; found, 354.1662.

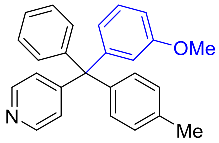

4-((3-Methoxyphenyl)(phenyl)(*p*-tolyl)methyl)pyridine (**6i**): The reaction was performed following the General Procedure A with (4-methylphenyl)(4-pyridyl)phenylmethane (**5c**, 25.9 mg, 0.10 mmol) and 3-chloroanisole (**2k**, 24.5 μL, 0.20 mmol, 2.0 equiv) with $Pd(OAc)_2$ (5.0 mol %), PCy_3 (10.0 mol %) and NaO*t*-Bu (19.2 mg, 0.20 mmol, 2.0 equiv) in 0.5 mL of dry THF for 12 h at 100 °C. The crude material was purified by flash chromatography on silica gel (eluted with hexanes:ethyl acetate, 3:1 to 2:1 v/v) to yield the product **6i** (35.4 mg, 97%) as a colorless oil. TLC (hexanes:ethyl acetate, 2:1 v/v): R_f = 0.36. ^{1}H NMR (500 MHz, $CDCl_3$): δ 8.48 (dd, *J* = 4.6, 1.6 Hz, 2H), 7.28 – 7.24 (m, 2H), 7.22 – 7.16 (m, 6H), 7.06 (s, 4H), 6.80 – 6.72 (m, 3H), 3.69 (s, 3H), 2.32 (s, 3H); ^{13}C NMR (125 MHz, $CDCl_3$): δ 159.17, 155.92, 149.36, 147.27, 145.48, 142.35, 136.17, 131.02, 130.92, 128.69, 128.62, 127.87, 126.51, 126.13, 123.91, 117.78, 110.98, 64.54, 55.28, 21.03; IR (thin film): 3023, 1590, 1542, 1508, 1489, 1446, 1291, 1253, 1053, 784, 756, 743, 703, 634 cm^{-1}; HRMS (m/z) : $[M + H]^+$ calcd. for $C_{26}H_{24}NO$, 366.1858; found, 366.1849.

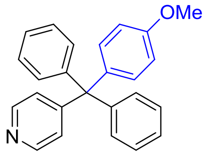

4-((4-Methoxyphenyl)diphenylmethyl)pyridine (**6j**): The reaction was performed following the General Procedure A with 4-benzhydrylpyridine (**5a**, 24.5 mg, 0.10 mmol) and 4-chloromethoxybenzene (**2n**, 24.5 µL, 0.20 mmol, 2.0 equiv) with $Pd(OAc)_2$ (5.0 mol %), PCy_3 (10.0 mol %) and NaO*t*-Bu (19.2 mg, 0.20 mmol, 2.0 equiv) in 0.5 mL of dry THF for 12 h at 100 °C. The crude material was purified by flash chromatography on silica gel (eluted with hexanes:ethyl acetate, 3:1 to 2:1 v/v) to yield the product **6j** (27.4 mg, 78%) as a white solid; m.p.= 165 – 167 °C. TLC (hexanes:ethyl acetate, 2:1 v/v): R_f = 0.32. ^{1}H NMR (500 MHz, $CDCl_3$): δ 8.49 (dd, *J* = 4.6, 1.6 Hz, 2H), 7.28 – 7.24 (m, 4H), 7.23 – 7.19 (m, 2H), 7.18 – 7.15 (m, 6H), 7.09 – 7.05 (m, 2H), 6.82 – 6.78 (m, 2H), 3.78 (s, 3H); ^{13}C NMR (125 MHz, $CDCl_3$): δ 158.01, 156.11, 149.39, 145.70, 137.57, 132.13, 131.00, 127.89, 126.50, 126.12, 113.20, 64.18, 55.33; IR (thin film): 3056, 3030, 2953, 2835, 1590, 1509, 1493, 1463, 1443, 1410, 1299, 1252, 1184, 1035, 995, 830, 812, 744, 703, 630 cm^{-1}; HRMS (m/z) : $[M + H]^+$ calcd. for $C_{25}H_{22}NO$, 352.1701; found, 352.1701.

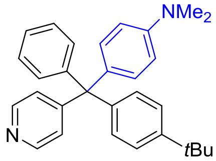

4-((4-(*tert*-Butyl)phenyl)(phenyl)(pyridine-4-yl)methyl)-*N,N*-dimethylaniline (**6k**): The reaction was performed following the General Procedure B with (4-*tert*-butylphenyl)(4-pyridyl)phenylmethane (**5b**, 30.1 mg, 0.10 mmol)and 4-chloro-*N,N*-dimethylaniline (**2m**, 31.2 mg, 0.20 mmol, 2.0 equiv) with $Pd(OAc)_2$ (5.0 mol %), cataCXium A (10.0 mol %) and NaO*t*-Bu (19.2 mg, 0.20 mmol, 2.0 equiv) in 0.5 mL of dry 1,4-dioxane for 12 h at 100 °C. The crude material was purified by flash chromatography on silica gel (eluted with hexanes:ethyl acetate, 3:1 to 2:1 v/v) to yield the product **6k** (38.6 mg, 92%) as a light green solid; m.p.= 198 – 201 °C. TLC (hexanes:ethyl acetate, 2:1 v/v): R_f = 0.41. ^{1}H NMR (500 MHz, $CDCl_3$): δ 8.47 (dd, *J* = 4.8, 1.5 Hz, 2H), 7.27 – 7.23 (m, 4H), 7.22 – 7.16 (m, 5H), 7.10 – 7.06 (m, 2H), 7.02 – 6.96 (m, 2H), 6.63 – 6.58 (m, 2H), 2.92 (s, 6H), 1.30 (s, 9H); ^{13}C NMR (125 MHz, $CDCl_3$): δ 157.38, 149.05, 148.70, 148.58, 146.10, 142.68, 133.19, 131.73, 130.98, 130.57, 127.72, 126.32, 126.25, 124.64, 111.60, 63.67, 40.99, 34.45, 31.45; IR (thin film): 2961, 1647, 1609, 1590, 1519, 1457, 1397, 1362, 1269, 1165, 1128, 1072, 1018, 949, 810, 703, 630 cm^{-1}; HRMS (m/z) : $[M + H]^+$ calcd. for $C_{30}H_{33}N_2$, 421.2644; found, 421.2630.

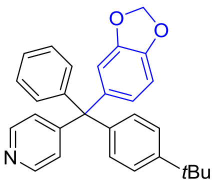

4-(Benzo[*d*][1,3]dioxol-5-yl(4-(*tert*-butyl)phenyl)(phenyl)methyl)pyridine (**6l**): The reaction was performed following the General Procedure B with (4-*tert*-butylphenyl)(4-pyridyl)phenylmethane (**5b**, 30.1 mg, 0.10 mmol)and 5-chloro-1,3-benzodioxole (**2o**, 23.4 μL, 0.20 mmol, 2.0 equiv) with $Pd(OAc)_2$ (5.0 mol %), cataCXium A (10.0 mol %) and NaO*t*-Bu (19.2 mg, 0.20 mmol, 2.0 equiv) in 1.0 mL of dry 1,4-dioxane for 12 h at 100 °C. The crude material was purified by flash chromatography on silica gel (eluted with hexanes:ethyl acetate, 4:1 to 3:1 v/v) to yield the product **6l** (37.1 mg, 88%) as a yellow solid; m.p.= 194 – 196 °C. TLC (hexanes:ethyl acetate, 2:1 v/v): R_f = 0.55. ^{1}H NMR (500 MHz, $CDCl_3$): δ 8.48 (dd, *J* = 4.6, 1.6 Hz, 2H), 7.29 – 7.24 (m, 4H), 7.22 – 7.15 (m, 5H), 7.10 – 7.05 (m, 2H), 6.72 – 6.59 (m, 3H), 5.92 (s, 2H), 1.30 (s, 9H); ^{13}C NMR (125 MHz, $CDCl_3$): δ 156.13, 149.34, 149.29, 147.28, 146.02, 145.65, 142.23, 139.68, 130.98, 130.58, 127.85, 126.48, 126.11, 124.78, 124.22, 112.01, 107.36, 101.22, 64.22, 34.49, 31.44; IR (thin film): 3031, 1596, 1496, 1452, 1415, 1373, 1279, 1167, 1134, 1072, 1032, 903, 703, 682, 622 cm^{-1}; HRMS (m/z) : $[M + H]^+$ calcd. for $C_{29}H_{28}NO_2$, 422.2120; found, 422.2126.

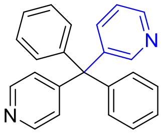

3-(Diphenyl(pyridin-4-yl)methyl)pyridine (**6m**): The reaction was performed following the General Procedure B with 4-benzhydrylpyridine (**5a**, 24.5 mg, 0.10 mmol) and 3-chloropyridine (**2q**, 19.0 μL, 0.20 mmol, 2.0 equiv) with $Pd(OAc)_2$ (5.0 mol %), cataCXium A (10.0 mol %) and NaO*t*-Bu (19.2 mg, 0.20 mmol, 2.0 equiv) in 1.0 mL of dry 1,4-dioxane for 12 h at 100 °C. The crude material was purified by flash chromatography on silica gel (eluted with hexanes:ethyl acetate, 1:1 to 0:1 v/v) to yield the product **6m** (30.3 mg, 94%) as a white solid; m.p.= 245 – 246 °C. TLC (ethyl acetate): R_f = 0.35. ^{1}H NMR (500 MHz, $CDCl_3$): δ 8.53 (dd, *J* = 4.7, 1.6 Hz, 2H), 8.48 (dd, *J* = 2.8, 2.1 Hz, 2H), 7.53 – 7.51 (m, 1H), 7.33 – 7.27 (m, 4H), 7.26 – 7.20 (m, 3H), 7.17 – 7.13 (m, 6H); ^{13}C NMR (125 MHz, $CDCl_3$): δ 154.76, 152.30, 149.70, 147.66, 144.35, 141.13, 138.04, 130.78, 128.26, 126.98, 125.82, 122.73, 63.18; IR (thin film): 3032, 1588, 1542, 1507, 1490, 1445, 1412, 1263, 1074, 1023, 805, 759, 703, 654, 635 cm^{-1}; HRMS (m/z) : $[M + H]^+$ calcd. for $C_{23}H_{19}N_2$, 323.1548; found, 323.1558.

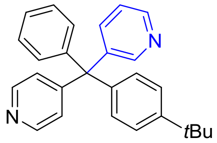 **3-((4-(*tert*-Butyl)phenyl)(phenyl)(pyridin-4-yl)methyl)pyridine** (**6n**): The reaction was performed following the General Procedure B with (4-*tert*-butylphenyl)(4-pyridyl)phenylmethane (**5b**, 30.1 mg, 0.10 mmol) and 3-chloropyridine (**2q**, 19.0 μL, 0.20 mmol, 2.0 equiv) with $Pd(OAc)_2$ (5.0 mol %), cataCXium A (10.0 mol %) and NaO*t*-Bu (19.2 mg, 0.20 mmol, 2.0 equiv) in 1.0 mL of dry 1,4-dioxane for 12 h at 100 °C. The crude material was purified by flash chromatography on silica gel (eluted with hexanes:ethyl acetate, 1:1 to 0:1 v/v) to yield the product **6n** (36.3 mg, 96%) as a light yellow solid; m.p.= 186 – 188 °C. TLC (ethyl acetate): R_f = 0.47. ^{1}H NMR (500 MHz, $CDCl_3$): δ 8.52 (dd, *J* = 4.6, 1.6 Hz, 2H), 8.49 – 8.45 (m, 2H), 7.52 (ddd, *J* = 8.1, 2.5, 1.6 Hz, 1H), 7.32 – 7.27 (m, 4H), 7.25 – 7.20 (m, 2H), 7.17 – 7.14 (m, 4H), 7.07 – 7.04 (m, 2H), 1.30 (s, 9H); ^{13}C NMR (125 MHz, $CDCl_3$): δ 154.95, 152.33, 149.79, 149.60, 147.52, 144.52, 141.31, 141.12, 137.99, 130.78, 130.40, 128.15, 126.86, 125.83, 125.08, 122.66, 62.77, 34.50, 31.37; IR (thin film): 3030, 2961, 1591, 1542, 1507, 1490, 1445, 1413, 1362, 1270, 1108, 1073, 1023, 832, 813, 762, 718, 705, 634 cm^{-1}; HRMS (m/z) : $[M + H]^+$ calcd. for $C_{27}H_{27}N_2$, 379.2174; found, 379.2166.

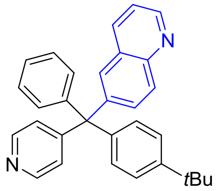 **6-((4-(*tert*-Butyl)phenyl)(phenyl)(pyridin-4-yl)methyl)quinoline** (**6o**): The reaction was performed following the General Procedure B with (4-*tert*-butylphenyl)(4-pyridyl)phenylmethane (**5b**, 30.1 mg, 0.10 mmol) and 6-chloroquinoline (**2v**, 32.7 mg, 0.20 mmol, 2.0 equiv) with $Pd(OAc)_2$ (5.0 mol %), cataCXium A (10.0 mol %) and NaO*t*-Bu (19.2 mg, 0.20 mmol, 2.0 equiv) in 1.0 mL of dry 1,4-dioxane for 12 h at 100 °C. The crude material was purified by flash chromatography on silica gel (eluted with hexanes:ethyl acetate, 1:1 to 0:1 v/v) to yield the product **6o** (32.9 mg, 77%) as a white foam. TLC (ethyl acetate): R_f = 0.53. ^{1}H NMR (500 MHz, $CDCl_3$): δ 8.89 (dd, *J* = 4.3, 1.7 Hz, 1H), 8.53 (d, *J* = 5.7 Hz, 2H), 8.03 (dd, *J* = 8.4, 1.0 Hz, 1H), 7.97 (d, *J* = 9.0 Hz, 1H), 7.67 (d, *J* = 2.2 Hz, 1H), 7.46 (dd, *J* = 9.0, 2.2 Hz, 1H), 7.37 (dd, *J* = 8.3, 4.3 Hz, 1H), 7.32 – 7.28 (m, 4H), 7.27 – 7.24 (m, 1H), 7.23 – 7.20 (m, 4H), 7.13 – 7.10 (m, 2H), 1.31 (s, 9H); ^{13}C NMR (125 MHz, $CDCl_3$): δ 155.50, 150.76, 149.65, 149.51, 147.14, 145.00, 144.04, 141.57, 136.52, 133.98, 131.06, 130.67, 128.52, 128.18, 128.05, 127.73, 126.75, 126.13, 124.98, 121.39, 64.40, 34.52, 31.41; IR (thin film): 3031, 2962, 2867, 1590, 1542, 1507,

1492, 1445, 1410, 1362, 1268, 1116, 1073, 1018, 887, 827, 803, 738, 719, 704, 621 cm^{-1}; HRMS (m/z) : $[M + H]^+$ calcd. for $C_{31}H_{29}N_2$, 429.2331; found, 429.2332.

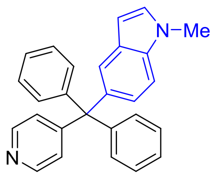

5-(Diphenyl(pyridine-4-yl)methyl)-1-methyl-1*H*-indole (**6p**): The reaction was performed following the General Procedure B with 4-benzhydrylpyridine (**5a**, 24.5 mg, 0.10 mmol) and 5-chloro-1-methyl-1*H*-indole (**2p**, 33.1 mg, 0.20 mmol, 2.0 equiv) with $Pd(OAc)_2$ (5.0 mol %), cataCXium A (10.0 mol %) and NaO*t*-Bu (19.2 mg, 0.20 mmol, 2.0 equiv) in 1.0 mL of dry 1,4-dioxane for 12 h at 100 °C. The crude material was purified by flash chromatography on silica gel (eluted with hexanes:ethyl acetate, 2:1 to 1:1 v/v) to yield the product **6p** (27.3 mg, 73%) as a brown solid; m.p.= 188 – 191 °C. TLC (hexanes:ethyl acetate, 1:1 v/v): R_f = 0.59. ^{1}H NMR (500 MHz, $CDCl_3$): δ 8.48 (d, *J* = 6.0 Hz, 2H), 7.45 (d, *J* = 1.8 Hz, 1H), 7.28 – 7.25 (m, 6H), 7.24 – 7.19 (m, 7H), 7.03 (d, *J* = 3.1 Hz, 1H), 6.94 (dd, *J* = 8.7, 1.9 Hz, 1H), 6.39 (d, *J* = 3.0 Hz, 1H), 3.77 (s, 3H); ^{13}C NMR (125 MHz, $CDCl_3$): δ 157.36, 148.68, 146.09, 136.46, 135.09, 131.20, 129.26, 127.82, 127.80, 126.51, 126.37, 126.20, 122.54, 108.72, 101.63, 64.90, 33.02; IR (thin film): 3055, 1590, 1490, 1445, 1420, 1338, 1249, 1091, 1034, 801, 760, 728, 703, 659, 638 cm^{-1}; HRMS (m/z) : $[M + H]^+$ calcd. for $C_{27}H_{23}N_2$, 375.1861; found, 375.1868.

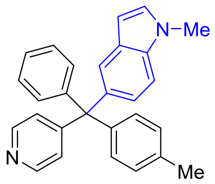

1-Methyl-5-(phenyl(pyridin-4-yl)(*p*-tolyl)methyl)-1*H*-indole (**6q**): The reaction was performed following the General Procedure B with (4-methylphenyl)(4-pyridyl)phenylmethane (**5c**, 25.9 mg, 0.10 mmol) and 5-chloro-1-methyl-1*H*-indole (**2p**, 33.1 mg, 0.20 mmol, 2.0 equiv) with $Pd(OAc)_2$ (5.0 mol %), cataCXium A (10.0 mol %) and NaO*t*-Bu (19.2 mg, 0.20 mmol, 2.0 equiv) in 1.0 mL of dry 1,4-dioxane for 12 h at 100 °C. The crude material was purified by flash chromatography on silica gel (eluted with hexanes:ethyl acetate, 2:1 to 1:1 v/v) to yield the product **6q** (30.3 mg, 78%) as a brown solid; m.p.= 224 – 226 °C. TLC (hexanes:ethyl acetate, 1:1 v/v): R_f = 0.55. ^{1}H NMR (500 MHz, $CDCl_3$): δ 8.46 (d, *J* = 6.2 Hz, 2H), 7.45 (d, *J* = 1.8 Hz, 1H), 7.26 – 7.21 (m, 6H), 7.20 – 7.16 (m, 2H), 7.11 (d, *J* = 8.3 Hz, 2H), 7.05 (d, *J* = 8.4 Hz, 2H), 7.00 (d, *J* = 3.1 Hz, 1H), 6.94 (dd, *J* = 8.7, 1.9 Hz, 1H), 6.38 (d, *J* = 3.1 Hz, 1H), 3.73 (s, 3H), 2.31 (s, 3H); ^{13}C NMR (125 MHz, $CDCl_3$): δ 157.12,

148.90, 146.28, 143.16, 136.64, 135.84, 135.01, 131.12, 131.03, 129.15, 128.47, 127.73, 127.71, 126.37, 126.22, 126.16, 122.44, 108.61, 101.57, 64.48, 32.95, 21.00; IR (thin film): 3022, 1590, 1509, 1491, 1444, 1421, 1338, 1249, 1100, 1035, 808, 759, 729, 704, 650 cm^{-1}; HRMS (m/z) : $[M + Na]^+$ calcd. for $C_{28}H_{24}N_2Na$, 411.1837; found, 411.1833.

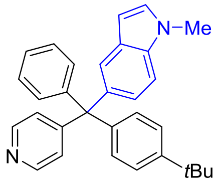

5-((4-(*tert*-Butyl)phenyl(phenyl)(pyridine-4-yl)methyl)-1-methyl-1*H*-indole (**6r**): The reaction was performed following the General Procedure B with (4-*tert*-butylphenyl)(4-pyridyl)phenylmethane (**5b**, 30.1 mg, 0.10 mmol) and 5-chloro-1-methyl-1*H*-indole (**2p**, 33.1 mg, 0.20 mmol, 2.0 equiv) with $Pd(OAc)_2$ (5.0 mol %), cataCXium A (10.0 mol %) and NaO*t*-Bu (19.2 mg, 0.20 mmol, 2.0 equiv) in 1.0 mL of dry 1,4-dioxane for 12 h at 100 °C. The crude material was purified by flash chromatography on silica gel (eluted with hexanes:ethyl acetate, 2:1 to 1:1 v/v) to yield the product **6r** (32.7 mg, 76%) as a brown solid; m.p.= 179 – 181 °C. TLC (hexanes:ethyl acetate, 1:1 v/v): R_f = 0.56. ^{1}H NMR (500 MHz, $CDCl_3$): δ 8.46 (dd, *J* = 4.7, 1.6 Hz, 2H), 7.46 (d, *J* = 1.8 Hz, 1H), 7.25 – 7.21 (m, 8H), 7.20 – 7.16 (m, 2H), 7.15 – 7.11 (m, 2H), 6.99 (d, *J* = 3.1 Hz, 1H), 6.95 (dd, *J* = 8.7, 1.9Hz, 1H), 6.38 (dd, *J* = 3.0, 0.6 Hz, 1H), 3.73 (s, 3H), 1.30 (s, 9H); ^{13}C NMR (125 MHz, $CDCl_3$): δ 157.05, 148.96, 146.34, 142.92, 136.71, 135.02, 131.17, 130.75, 129.11, 127.72, 127.67, 126.38, 126.20, 126.19, 124.60, 122.49, 108.54, 101.57, 64.41, 34.44, 32.95, 31.45; IR (thin film): 3030, 1590, 1508, 1490, 1444, 1411, 1338, 1249, 1073, 1017, 813, 800, 762, 734, 711, 648 cm^{-1}; HRMS (m/z) : $[M + H]^+$ calcd. for $C_{31}H_{31}N_2$, 431.2487; found, 431.2504.

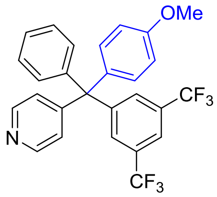

4-((3,5-Bis(trifluoromethyl)phenyl)(4-methoxyphenyl)(phenyl)methyl)pyridine (**6s**): The reaction was performed following the General Procedure B with bis(trifluoromethyl)phenyl)(phenyl)methyl)pyridine (**5d**, 38.1 mg, 0.10 mmol) and 4-chloromethoxybenzene (**2n**, 24.5 μL, 0.20 mmol, 2.0 equiv) with $Pd(OAc)_2$ (5.0 mol %), cataCXium A (10.0 mol %) and NaO*t*-Bu (19.2 mg, 0.20 mmol, 2.0 equiv) in 1.0 mL of dry 1,4-dioxane for 12 h at 100 °C. The crude material was purified by flash chromatography on silica gel (eluted with hexanes:ethyl

acetate, 4:1 to 3:1 v/v) to yield the product **6s** (37.5 mg, 77%) as a yellow oil. TLC (hexanes:ethyl acetate, 2:1 v/v): R_f = 0.42. ^{1}H NMR (500 MHz, $CDCl_3$): δ 8.56 (dd, *J* = 4.6, 1.6 Hz, 2H), 7.77 (s, 1H), 7.69 (s, 2H), 7.34 – 7.30 (m, 2H), 7.29 – 7.25 (m, 1H), 7.15 – 7.11 (m, 4H), 7.05 – 7.01 (m, 2H), 6.87 – 6.83 (m, 2H), 3.80 (s, 3H); ^{13}C NMR (125 MHz, $CDCl_3$): δ 158.32, 154.59, 149.92, 148.67, 144.19, 135.93, 131.80, 131.29 (q, *J* = 33.2 Hz), 130.77 (d, *J* = 2.7 Hz), 130.60, 128.50, 127.29, 125.57, 123.34(q, *J* = 271.3 Hz), 120.75 (m), 113.82, 64.21, 55.38; IR (thin film): 3033, 2839, 1607, 1590, 1509, 1365, 1279, 1255, 1185, 1134, 1036, 832, 705, 682, 645 cm^{-1}; HRMS (m/z) : [M + H]$^+$ calcd. for $C_{27}H_{20}NOF_6$, 488.1449; found, 488.1444.

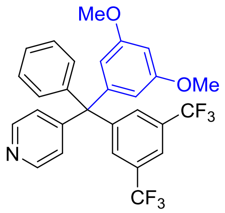

4-((3,5-Bis(trifluoromethyl)phenyl)(3,5-dimethoxyphenyl)(phenyl)methyl)pyridine (**6t**): The reaction was performed following the General Procedure B with bis(trifluoromethyl)phenyl)(phenyl)-methyl)pyridine (**5d**, 38.1 mg, 0.10 mmol) and 5-chloro-1,3-dimethoxybenzene (**2l**, 34.5 mg, 0.20 mmol, 2.0 equiv) with $Pd(OAc)_2$ (5.0 mol %), cataCXium A (10.0 mol %) and NaO*t*-Bu (19.2 mg, 0.20 mmol, 2.0 equiv) in 1.0 mL of dry 1,4-dioxane for 12 h at 100 °C. The crude material was purified by flash chromatography on silica gel (eluted with hexanes:ethyl acetate, 4:1 to 3:1 v/v) to yield the product **6t** (47.1 mg, 91%) as a yellow oil. TLC (hexanes:ethyl acetate, 2:1 v/v): R_f = 0.39. ^{1}H NMR (500 MHz, $CDCl_3$): δ 8.56 (dd, *J* = 4.7, 1.6 Hz, 2H), 7.77 (s, 1H), 7.73 (s, 2H), 7.34 – 7.30 (m, 2H), 7.29 – 7.25 (m, 1H), 7.15 – 7.12 (m, 4H), 6.37 (t, *J* = 2.2 Hz, 1H), 6.25 (d, *J* = 2.2 Hz, 2H), 3.67 (s, 6H); ^{13}C NMR (125 MHz, $CDCl_3$): δ 160.69, 154.08, 149.94, 148.13, 146.18, 143.67, 131.28 (q, *J* = 33.1 Hz), 130.88 (d, *J* = 2.1 Hz), 130.63, 128.52, 127.41, 125.60, 123.34(q, *J* = 271.2 Hz), 120.86 (m), 109.83, 98.52, 64.97, 55.45; IR (thin film): 2936, 1591, 1507, 1457, 1364, 1206, 1159, 1069, 897, 842, 704, 682, 668 cm^{-1}; HRMS (m/z) : [M + H]$^+$ calcd. for $C_{28}H_{22}NO_2F_6$, 518.1555; found, 518.1548.

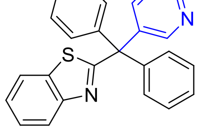

2-(Diphenyl(pyridine-3-yl)methyl)benzo[*d*]thiazole (**9a**): The reaction was performed following the General Procedure B with 2-benzhydrylbenzo[*d*]thiazole (**8a**, 60.2 mg, 0.20 mmol) and 3-chloropyridine (**2q**, 38.0 μL, 0.40 mmol, 2.0 equiv) with $Pd(OAc)_2$ (5.0 mol %), cataCXium A (10.0 mol %) and

NaO*t*-Bu (38.4 mg, 0.40 mmol, 2.0 equiv) in 2.0 mL of dry 1,4-dioxane for 12 h at 100 °C. The crude material was purified by flash chromatography on silica gel (eluted with hexanes:ethyl acetate, 4:1 to 3:1 v/v) to yield the product **9a** (67.3 mg, 89%) as a white solid; m.p.= 128 – 130 °C. TLC (hexanes:ethyl acetate, 2:1 v/v): R_f = 0.35. ^{1}H NMR (500 MHz, $CDCl_3$): δ 8.63 (dd, *J* = 2.5, 0.7 Hz, 1H), 8.53 (dd, *J* = 4.7, 1.6 Hz, 1H), 8.09 – 8.03 (m, 1H), 7.82 – 7.76 (m, 2H), 7.47 (ddd, *J* = 8.3, 7.2, 1.2 Hz, 1H), 7.36 (ddd, *J* = 8.2, 7.3, 1.1 Hz, 1H), 7.34 – 7.30 (m, 6H), 7.29 – 7.21 (m, 5H); ^{13}C NMR (125 MHz, $CDCl_3$): δ 117.90, 153.57, 152.22, 148.18, 144.44, 140.94, 138.06, 135.54, 130.65, 128.18, 127.76, 126.19, 125.40, 123.77, 122.68, 121.25, 63.23; IR (thin film): 3057, 1571, 1490, 1434, 1414, 1314, 1276, 1118, 1024, 878, 796, 759, 702 cm^{-1}; HRMS (m/z) : [M + H]$^+$ calcd. for $C_{25}H_{19}N_2S$, 379.1269; found, 379.1290.

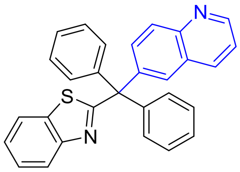

2-(Diphenyl(quinolin-6-yl)methyl)benzo[*d*]thiazole (**9b**): The reaction was performed following the General Procedure B with 2-benzhydrylbenzo[*d*]thiazole (**8a**, 60.2 mg, 0.20 mmol) and 6-chloroquinoline (**2v**, 65.4 mg, 0.40 mmol, 2.0 equiv) with $Pd(OAc)_2$ (5.0 mol %), cataCXium A (10.0 mol %) and NaO*t*-Bu (38.4 mg, 0.40 mmol, 2.0 equiv) in 2.0 mL of dry 1,4-dioxane for 12 h at 100 °C. The crude material was purified by flash chromatography on silica gel (eluted with hexanes:ethyl acetate, 4:1 to 3:1 v/v) to yield the product **9b** (78.8 mg, 92%) as a light yellow oil. TLC (hexanes:ethyl acetate, 2:1 v/v): R_f = 0.37. ^{1}H NMR (500 MHz, $CDCl_3$): δ 8.89 (dd, *J* = 4.2, 1.7 Hz, 1H), 8.08 (d, *J* = 8.2 Hz, 1H), 8.03 (d, *J* = 9.0 Hz, 1H), 8.00 (dd, *J* = 8.3, 0.9 Hz, 1H), 7.83 – 7.76 (m, 2H), 7.72 (dd, *J* = 9.0, 2.3 Hz, 1H), 7.50 – 7.43 (m, 1H), 7.40 – 7.30 (m, 12H); ^{13}C NMR (125 MHz, $CDCl_3$): δ 178.41, 153.65, 150.93, 147.55, 144.95, 143.56, 136.81, 135.55, 133.34, 130.84, 128.95, 128.51, 128.08, 127.69, 127.60, 126.11, 125.31, 123.77, 121.36, 121.25, 64.87; IR (thin film): 3058, 1593, 1572, 1493, 1435, 1314, 1264, 1122, 1035, 880, 834, 798, 760, 739, 701 cm^{-1}; HRMS (m/z) : [2M + H]$^+$ calcd. for $C_{58}H_{41}N_4S_2$, 857.2773; found, 857.2768.

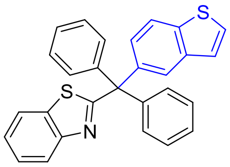

2-(Benzo[*b*]thiophen-5-yldiphenylmethyl)benzo[*d*]thiazole (**9c**): The reaction was performed following the General Procedure B with 2-benzhydrylbenzo[*d*]thiazole (**8a**, 60.2 mg, 0.20 mmol) and 5-

chlorobenzothiophene (**2w**, 67.5 mg, 0.40 mmol, 2.0 equiv) with $Pd(OAc)_2$ (5.0 mol %), cataCXium A (10.0 mol %) and NaO*t*-Bu (38.4 mg, 0.40 mmol, 2.0 equiv) in 2.0 mL of dry 1,4-dioxane for 12 h at 100 °C. The crude material was purified by flash chromatography on silica gel (eluted with hexanes:ethyl acetate, 8:1 to 6:1 v/v) to yield the product **9c** (52.8 mg, 61%) as a yellow oil. TLC (hexanes:ethyl acetate, 5:1 v/v): R_f = 0.73. ^{1}H NMR (500 MHz, $CDCl_3$): δ 8.10 – 8.08 (m, 1H), 7.80 – 7.76 (m, 2H), 7.75 (d, *J* = 1.8 Hz, 1H), 7.46 (ddd, *J* = 8.3, 7.2, 1.2 Hz, 1H), 7.39 (d, *J* = 5.4 Hz, 1H), 7.37 – 7.33 (m, 6H), 7.31 – 7.28 (m, 6H), 7.21 (dd, *J* = 5.4, 0.5 Hz, 1H); ^{13}C NMR (125 MHz, $CDCl_3$): δ 179.25, 153.76, 145.50, 141.80, 139.44, 138.73, 135.61, 130.94, 127.94, 127.38, 126.83, 126.01, 125.49, 125.17, 124.61, 123.77, 121.67, 121.22, 64.93; IR (thin film): 3059, 2923, 1596, 1557, 1491, 1435, 1314, 1264, 1112, 1049, 887, 806, 758, 700 cm^{-1}; HRMS (m/z) : $[M + H]^+$ calcd. for $C_{28}H_{20}NS_2$, 434.1037; found, 434.1041.

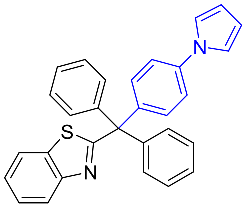

2-((4-(1*H*-Pyrrol-1-yl)phenyl)diphenylmethyl)benzo[*d*]thiazole (**9d**): The reaction was performed following the General Procedure B with 2-benzhydrylbenzo[*d*]thiazole (**8a**, 60.2 mg, 0.20 mmol) and 1-(4-chlorophenyl)-1*H*-pyrrole (**2x**, 71.1 mg, 0.40 mmol, 2.0 equiv) with $Pd(OAc)_2$ (5.0 mol %), cataCXium A (10.0 mol %) and NaO*t*-Bu (38.4 mg, 0.40 mmol, 2.0 equiv) in 2.0 mL of dry 1,4-dioxane for 12 h at 100 °C. The crude material was purified by flash chromatography on silica gel (eluted with hexanes:ethyl acetate, 8:1 to 6:1 v/v) to yield the product **9d** (55.7 mg, 63%) as a yellow oil. TLC (hexanes:ethyl acetate, 5:1 v/v): R_f = 0.72. ^{1}H NMR (500 MHz, $CDCl_3$): δ 8.08 (d, *J* = 8.2 Hz, 1H), 7.81 – 7.74 (m, 1H), 7.48 – 7.44 (m, 1H), 7.42 – 7.38 (m, 2H), 7.37 – 7.28 (m, 13H), 7.09 (t, *J* = 2.2 Hz, 2H), 6.33 (t, *J* = 2.2 Hz, 2H); ^{13}C NMR (125 MHz, $CDCl_3$): δ 178.85, 153.75, 145.25, 142.59, 139.62, 135.56, 132.08, 130.81, 128.01, 127.51, 126.09, 125.25, 123.76, 121.23, 119.49, 119.29, 110.67, 64.52; IR (thin film): 3057, 1608, 1557, 1517, 1489, 1434, 1330, 1314, 1264, 1111, 1069, 1021, 878, 759, 727, 704 cm^{-1}; HRMS (m/z) : $[M + H]^+$ calcd. for $C_{30}H_{23}N_2S$, 443.1582; found, 443.1595.

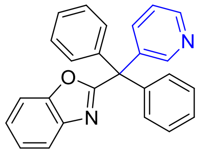

2-(Diphenyl(pyridine-3-yl)methyl)benzo[*d*]oxazole (**9e**): The reaction was performed following the General Procedure B with 2-benzhydrylbenzo[*d*]oxazole (**8b**, 57.0 mg, 0.20 mmol) and 3-chloropyridine (**2q**, 38.0 μL, 0.40 mmol, 2.0 equiv) with $Pd(OAc)_2$ (5.0 mol %), cataCXium A (10.0 mol %) and NaO*t*-Bu (38.4 mg, 0.40 mmol, 2.0 equiv) in 2.0 mL of dry 1,4-dioxane for 12 h at 100 °C. The crude material was purified by flash chromatography on silica gel (eluted with hexanes:ethyl acetate, 4:1 to 3:1 v/v) to yield the product **9e** (62.2 mg, 86%) as a white solid; m.p.= 167 – 169 °C. TLC (hexanes:ethyl acetate, 2:1 v/v): R_f = 0.33. ^{1}H NMR (500 MHz, $CDCl_3$): δ 8.56 – 8.55 (m, 1H), 8.54 (dd, *J* = 4.8, 1.5 Hz, 1H), 7.79 – 7.74 (m, 1H), 7.63 (ddd, *J* = 8.1, 2.5, 1.6 Hz, 1H), 7.50 – 7.45 (m, 1H), 7.35 – 7.29 (m, 8H), 7.28 – 7.24 (m, 1H), 7.21 – 7.15 (m, 4H); ^{13}C NMR (125 MHz, $CDCl_3$): δ 168.30, 151.89, 150.94, 148.36, 142.41, 140.83, 139.32, 138.05, 130.15, 128.35, 127.70, 125.40, 124.58, 122.71, 120.78, 110.95, 60.75; IR (thin film): 3057, 1550, 1493, 1453, 1415, 1241, 1146, 1025, 899, 807, 748, 700 cm^{-1}; HRMS (m/z) : [M + H]$^+$ calcd. for $C_{25}H_{19}$N2O, 363.1497; found, 363.1474.

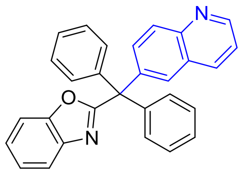

2-(Diphenyl(quinolin-6-yl)methyl)benzo[*d*]oxazole (**9f**): The reaction was performed following the General Procedure B with 2-benzhydrylbenzo[*d*]oxazole (**8b**, 57.0 mg, 0.20 mmol) and 6-chloroquinoline (**2v**, 65.4 mg, 0.40 mmol, 2.0 equiv) with $Pd(OAc)_2$ (5.0 mol %), cataCXium A (10.0 mol %) and NaO*t*-Bu (38.4 mg, 0.40 mmol, 2.0 equiv) in 2.0 mL of dry 1,4-dioxane for 12 h at 100 °C. The crude material was purified by flash chromatography on silica gel (eluted with hexanes:ethyl acetate, 4:1 to 3:1 v/v) to yield the product **9f** (77.5 mg, 94%) as a yellow oil. TLC (hexanes:ethyl acetate, 2:1 v/v): R_f = 0.31. ^{1}H NMR (500 MHz, $CDCl_3$): δ 8.90 (dd, *J* = 4.2, 1.7 Hz, 1H), 8.06 (d, *J* = 9.0 Hz, 1H), 8.02 – 7.96 (m, 1H), 7.82 – 7.78 (m, 1H), 7.73 (dd, *J* = 9.0, 2.2 Hz, 1H), 7.54 (d, *J* = 2.2 Hz, 1H), 7.51 – 7.46 (m, 1H), 7.37 – 7.30 (m, 9H), 7.28 – 7.24 (m, 4H); ^{13}C NMR (125 MHz, $CDCl_3$): δ 168.82, 150.93, 147.61, 142.96, 142.02, 140.96, 136.70, 133.18, 130.41, 128.71, 128.58, 128.24, 127.69, 127.58, 125.31, 124.52, 121.35, 120.83, 110.94, 62.43; IR (thin film): 3056, 1593, 1542, 1493, 1454, 1240, 1156, 1034, 880, 835, 793, 743, 699 cm^{-1}; HRMS (m/z) : [M + H]$^+$ calcd. for $C_{29}H_{21}N_2O$, 413.1654; found, 413.1661.

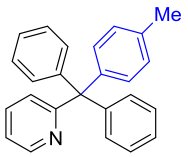

2-(Diphenyl(*p*-tolyl)methyl)pyridine (**9g**): The reaction was performed following the General Procedure B with 2-benzhydrylpyridine (**8c**, 49.0 mg, 0.20 mmol) and 4-chlorotoluene (**2d**, 60.0 µL, 0.40 mmol, 2.0 equiv) with $Pd(OAc)_2$ (5.0 mol %), cataCXium A (10.0 mol %) and NaO*t*-Bu (38.4 mg, 0.40 mmol, 2.0 equiv) in 2.0 mL of dry 1,4-dioxane for 12 h at 100 °C. The crude material was purified by flash chromatography on silica gel (eluted with hexanes:ethyl acetate, 6:1 to 5:1 v/v) to yield the product **9g** (62.3 mg, 93%) as a white solid; m.p.= 158 – 160 °C. TLC (hexanes:ethyl acetate, 4:1 v/v): R_f = 0.64. ^{1}H NMR (500 MHz, $CDCl_3$): δ 8.62 (ddd, *J* = 4.8, 1.9, 0.9 Hz, 1H), 7.53 (ddd, *J* = 8.0, 7.6, 1.9 Hz, 1H), 7.28 – 7.21 (m, 9H), 7.20 – 7.15 (m, 2H), 7.12 – 7.09 (m, 2H), 7.08 – 7.03 (m, 3H), 2.30 (s, 3H); ^{13}C NMR (125 MHz, $CDCl_3$): δ 165.79, 148.63, 146.65, 143.53, 135.66, 135.63, 131.20, 131.10, 128.36, 127.59, 126.09, 125.57, 120.89, 66.44, 21.06; IR (thin film): 3025, 1583, 1509, 1492, 1443, 1425, 1191, 1035, 812, 749, 701, 633 cm^{-1}; HRMS (m/z) : $[M + H]^+$ calcd. for $C_{25}H_{22}N$, 336.1752; found, 336.1751.

Transformation of reaction product. Synthesis of liquid crystals **11**.

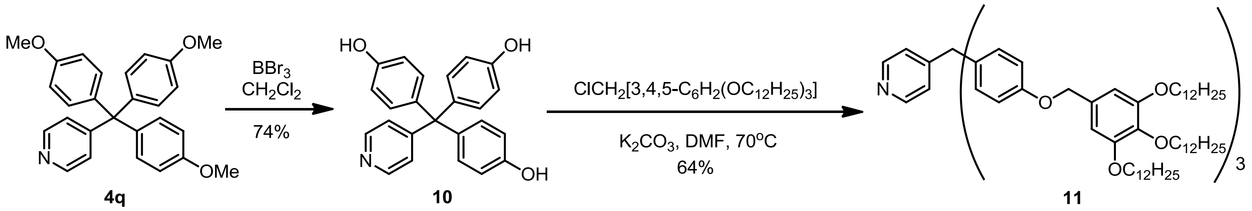

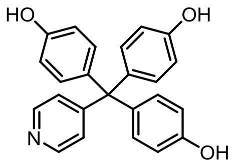

Tris(4-hydroxyphenyl)-4-pyridylmethane (**10**): An oven-dried 50 mL round bottom flask equipped with a stir bar was charged with 4-(Tris(4-methoxyphenyl)methyl)pyridine (**4q**, 123.3 mg, 0.30 mmol) and dry CH_2Cl_2 (10 mL)under nitrogen system. And then the mixture was kept at -78 °C. BBr_3 in methylene chloride (1.0 M, 1.8 mL, 1.8 mmol) was added slowly. The temperature was slowly rasied to 25 °C and the mixture was stirred overnight. The reaction was quenched with water at 0°C and extracted with ethyl actate. The collected organic fraction was washed with brine, dried over $MgSO_4$, and filtered over a pad of celite. The pad was rinsed with ethyl acetate, and the combined solutions were concentrated *in vacuo*. The crude material was purified by flash

chromatography on silica gel (eluted with hexanes:ethyl acetate, 1:1 to 0:1 v/v) to yield the product **10** (81.9 mg, 74%) as a white solid. The NMR spectral data match the previously published data.[10]

3,4,5-tridodecyloxybenzyl chloride was prepared according to literature procedures.[11]

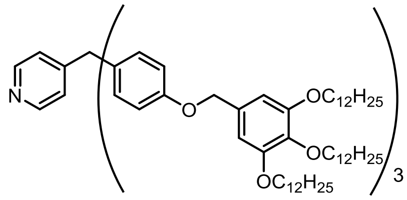

Tris(4-(3,4,5-tridodecyloxybenzyloxy)phenyl)-4-pyridylmethane (**11**): An oven-dried 50 mL round bottom flask equipped with a stir bar was charged with tris(4-hydroxyphenyl)-4-pyridylmethane (**10**, 50.0 mg, 0.14 mmol), 3,4,5-tridodecyloxybenzyl chloride (280 mg, 0.41 mmol), K_2CO_3 (190 mg, 1.4 mmol) and dry DMF (10 mL) in a glove box under a nitrogen atmosphere at room temperature. The flask was capped with a rubber septum, removed from the glove box, and stirred for 12 h at 70°C. After cooling to room temperature the reaction was uncapped and the reaction mixture quenched with of H_2O (100 mL), diluted with 50 mL of ethyl acetate, and filtered over a pad of celite. The collected organic fraction was washed with brine, dried over $MgSO_4$, and filtered over a pad of celite. The pad was rinsed with ethyl acetate, and the combined solutions were concentrated *in vacuo*. The crude material was purified by flash chromatography on silica gel (eluted with hexanes:ethyl acetate, 6:1 to 4:1 v/v) to yield the product **11** (205.9 mg, 64%) as a light yellow oil. The NMR spectral data match the previously published data.[10]

Gram Scale Preparation of ((Phenyl(pyridine-4-yl)methylene)bis(4,1-phenylene))bis(phenylmethanone) (**4g**):

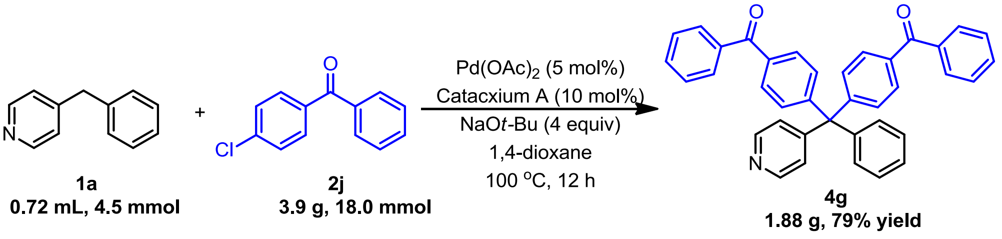

An oven-dried 100 mL round bottom flask equipped with a stir bar was charged with 4-benzylpyridine (**1a**, 0.72 mL, 4.5 mmol, 1.0 equiv) and 4-chlorobenzophenone (**2j**, 3.90 g, 18.0 mmol, 4.0 equiv) in a glove box under a nitrogen atmosphere at room temperature. A stock solution of $Pd(OAc)_2$ (50.4 mg, 0.23 mmol, 5 mol %) and cataCXium A (161.3 mg, 0.45 mmol, 10 mol %) in 22.5 mL of dry 1,4-dioxane was taken up by syringe and added to the reaction

flask under nitrogen. Next, NaO*t*-Bu (1.73 g, 18.0 mmol, 4.0 equiv) was added to the reaction mixture. The flask was capped with a rubber septum, removed from the glove box, and stirred for 12 h at 100°C. After cooling to room temperature the reaction was uncapped and the reaction mixture quenched with of H_2O (300 μL), diluted with 50 mL of ethyl acetate, and filtered over a pad of $MgSO_4$ and silica. The pad was rinsed with ethyl acetate (3 × 20 mL), and the combined solutions were concentrated *in vacuo*. The crude material was purified by flash chromatography on silica gel (eluted with hexanes : ethyl acetate = 2:1 to 1:1) to yield the product **4g** (1.88 g, 79%) as a light yellow solid.

Supplementary References

1. Efange, S. M. N., Michelson, R. H., Remmel, R. P., Boudreau, R. J., Dutta, A. K. & Freshler, A. Flexible N-methyl-4-phenyl-1,2,3,6-tetrahydropyridine analogues: synthesis and monamine oxidase catalyzed bioactivation. *J. Med. Chem*. **33**, 3133-3138 (1990).
2. Shiao, M.-J. & Chia, W.-L. A facile synthesis of 4-benzylpyridines by regiospecific addition of substituted benzylic Griganard reagents to pyridinium salts. *Synthetic. Commun*. **21**, 401-406 (1991).
3. Duez, S., Steib, A. K., Manolikakes, S. M. & Knochel, P. Lewis acid promoted benzylic cross-coupling of pyridines with aryl bromides. *Angew. Chem. Int. Ed*. **50**, 7686-7690 (2011).
4. Agai, B., Proszenyak, A., Tarkanyi, G., Vida, L. & Faigl, F. Convenient, benign and scalable synthesis of 2- and 4-substituted benzylpiperidines. *Eur. J. Org. Chem*. **2004**, 3623-3632 (2004).
5. Quinio, P., Roman, D. S., Leon, T., William, S., Karaghiosoff, K. & Knochel, P. Transition-metal-free cross-coupling of aryl and N-heteroaryl cyanides with benzylic zinc reagents. *Org. Lett*. **17**, 4396-4399 (2015).
6. Henry, N., Enguehard-Gueiffier, C., Thery, I. & Gueiffier, A. One-pot dual substitutions of bromobenzyl chloride, 2-chloromethyl-6-halogenoimidazo[1,2-*a*]pyridine and –[1,2-*b*]pyridazine by Suzuki-Miyaura cross-coupling reactions. *Eur. J. Org. Chem*. **2008**, 4824-4827 (2008).
7. Quinio, P., Roman, D. S., Leon, T., William, S., Karaghiosoff, K. & Knochel, P. Transition-metal-free cross-coupling of aryl and N-heteroaryl cyanides with benzylic Zinc reagents. *Org. Lett*. **17**, 4396-4399 (2015).
8. Song, G.-Y., Su, Y., Gong, X., Han, K. –L. & Li, X. –W. Pd(0)-catalyzed diarylation of sp^3 C-H bond in (2-azaaryl)methanes. *Org. Lett*. **13**, 1968-1971 (2013).
9. Zhang, J.-D., Bellomo, A., Creamer, A. D., Dreher, S. D. & Walsh, P. J. Palladium-catalyzed $C(sp^3)$-H arylation of diarylmethanes at room temperature: synthesis of triarylmethanes via deprotonative-cross-coupling processes. *J. Am. Chem. Soc*. **134**, 13765-13772 (2012).
10. Hatano, T. & Kato. T. Nanostructured columnar and cubic liquid-crystalline assemblies consisting of unconventional rigid mesogens based on triphenylmethanes. *Tetrahedron*. **64**, 8386-8380 (2008).

11. Kuritani, M., Tashiro, S. & Shionoya, M. Organic and organometallic nanofibers forms by supramolecular assembly of diamond-shaped macrocyclic ligands and Pd^{II} complexes. *Chem. Asian. J*. **8**, 1368-1371 (2013).
